# Predicting bond dissociation energies of cyclic hypervalent halogen reagents using DFT calculations and graph attention network model

**DOI:** 10.3762/bjoc.20.127

**Published:** 2024-06-28

**Authors:** Yingbo Shao, Zhiyuan Ren, Zhihui Han, Li Chen, Yao Li, Xiao-Song Xue

**Affiliations:** 1 State Key Laboratory of Elemento-Organic Chemistry, College of Chemistry, Nankai University, Tianjin 300071, P. R. China; 2 Key Laboratory of Fluorine and Nitrogen Chemistry and Advanced Materials, Shanghai Institute of Organic Chemistry, University of Chinese Academy of Sciences, Shanghai 200032, P. R. China; 3 School of Chemistry and Material Sciences, Hangzhou Institute for Advanced Study, University of Chinese Academy of Sciences, Hangzhou 310024, P. R. China

**Keywords:** BDE, cyclic hypervalent halogen reagents, DFT calculation, graph attention network, machine learning

## Abstract

Although hypervalent iodine(III) reagents have become staples in organic chemistry, the exploration of their isoelectronic counterparts, namely hypervalent bromine(III) and chlorine(III) reagents, has been relatively limited, partly due to challenges in synthesizing and stabilizing these compounds. In this study, we conduct a thorough examination of both homolytic and heterolytic bond dissociation energies (BDEs) critical for assessing the chemical stability and functional group transfer capability of cyclic hypervalent halogen compounds using density functional theory (DFT) analysis. A moderate linear correlation was observed between the homolytic BDEs across different halogen centers, while a strong linear correlation was noted among the heterolytic BDEs across these centers. Furthermore, we developed a predictive model for both homolytic and heterolytic BDEs of cyclic hypervalent halogen compounds using machine learning algorithms. The results of this study could aid in estimating the chemical stability and functional group transfer capabilities of hypervalent bromine(III) and chlorine(III) reagents, thereby facilitating their development.

## Introduction

**Figure 1:**
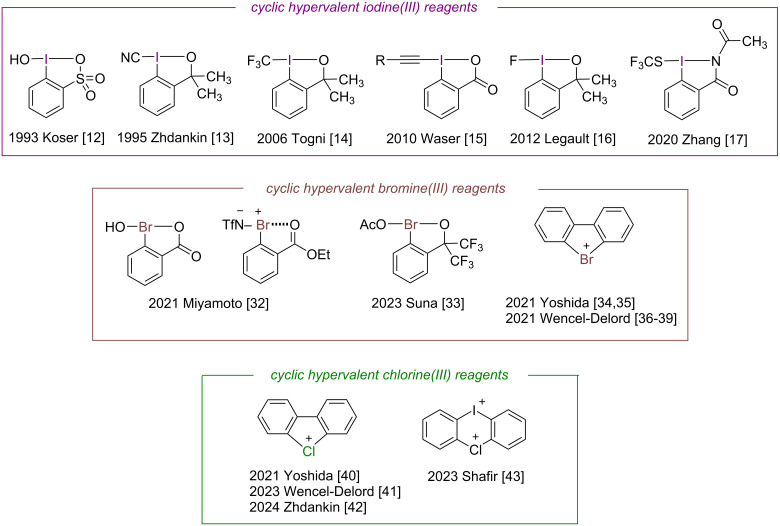
Examples of cyclic hypervalent halogen reagents.

Hypervalent iodine reagents are increasingly gaining attention in the fields of organic synthesis and catalysis due to their environmental benefits, accessibility, and cost-efficiency [1–11]. Over the last three decades, a series of cyclic hypervalent iodine(III) reagents has been developed [12–17] (Figure 1), including the well-known Zhdankin reagents [13] and Togni reagents [14]. These reagents are popularly used as electrophilic group transfer reagents [18–19] in a variety of reactions, such as C–H functionalization [20–22], unsaturated alkane addition [23–24], and cyclization [25–26].

Despite the rapid development of hypervalent iodine(III) reagents, the exploration of isoelectronic hypervalent bromine(III) and chlorine(III) reagents has been comparatively limited despite their demonstrated potential for unique applications [27–30]. For example, hypervalent bromine(III) reagents enable C–H amination and alkene aziridination reactions without the need for additional Lewis acid activation [31–33]. However, challenges in the synthesis and stabilization of cyclic hypervalent bromine and chlorine reagents have impeded their development relative to their iodine(III) analogs [27–30]. Cyclic hypervalent bromine(III) reagents were pioneered by Miyamoto [32] and have since been developed to a certain extent [33]. Biphenyl hypervalent bromine(III) reagents [34–39] have been synthesized by Yoshida and Wencel-Delord (Figure 1). Cyclic hypervalent chlorine(III) reagents with similar skeletal structures have not been reported yet, and only biphenyl hypervalent chlorine(III) reagents [40–42] and cyclic diaryliodonium salts [43] have been synthesized.

Previous investigations [44–47] have highlighted the critical role of bond dissociation energy (BDE) in understanding the group transfer capabilities and chemical stability of hypervalent iodine(III) reagents. In this context, detailed knowledge of the BDE of hypervalent bromine(III) and chlorine(III) reagents is especially crucial for designing novel reagents. Yet, the BDE values of hypervalent bromine(III) and chlorine(III) reagents remain largely elusive, hampering the design and synthesis of novel reagents.

In recent years, machine learning has emerged as a promising and cost-effective alternative to traditional DFT calculations for predicting key properties of organic molecules such as BDE, nucleophilicity, and electrophilicity [48–60]. Recently, applications of the Elastic Net model with Avalon fingerprints [55] and the deployment of artificial neural network (ANN) models [57] with the Mordred cheminformatics package have demonstrated considerable success in predicting the BDEs of hypervalent iodine(III) reagents. However, previous studies have been limited to the prediction of hypervalent iodine(III) reagents. Driven by their proven effectiveness and our ongoing interest in hypervalent halogen chemistry [61–72], we are motivated to develop a machine learning model for a broader array of cyclic hypervalent halogen reagents, thereby integrating different halogen centers and making it easier to predict the group transfer capacity and chemical stability of different cyclic hypervalent halogen reagents.

## Results and Discussion

**Figure 2:**
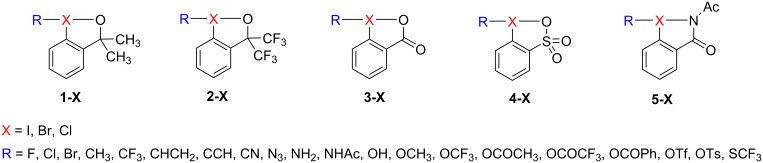
Common cyclic hypervalent halogen skeletons and transfer groups.

We selected five different skeletons and twenty common transfer groups for combination (Figure 2) and calculated their BDEs. Referring to the previous computational studies of hypervalent iodine [61–76] and the computational database of organic species by Paton and co-workers [77], geometry optimizations and single point energy calculations for homolytic BDEs are both performed using M06-2X/def2-TZVPP [78–80] in the gas phase at 298.15 K by Gaussian 16 [81]. Frequency calculations confirmed that optimized structures are minima (no imaginary frequency). The accuracy of computational BDEs of halides using M06-2X/def2-TZVPP is also evaluated and compared to experimental BDEs, demonstrating the reliability of the method (see Supporting Information File 1).

The computational homolytic BDEs are presented in Table 1. From the perspective of halogen centers, hypervalent iodine(III) reagents exhibit the highest homolytic BDEs, followed by hypervalent bromine(III) reagents, while hypervalent chlorine(III) reagents have the lowest. Generally, the homolytic BDEs of cyclic hypervalent iodine(III) reagents are above 30.0 kcal/mol, consistent with their good chemical stability. The homolytic BDEs of some cyclic hypervalent bromine(III) and most cyclic hypervalent chlorine(III) reagents are below 20 kcal/mol, implying these reagents should be too reactive to be isolated. From the perspective of transfer groups, the homolytic BDEs of groups with strong trans effects [82–84] such as -F, -CCH, -CN, -OCF_3_, -OTf, -OTs are elevated, while those of -N_3_, -NH_2_, -SCF_3_, etc. are smaller. These results are consistent with our previous studies on the group transfer ability of hypervalent iodine(III) reagents [44]. According to the calculation results, skeleton 5 may be a better candidate for synthesizing cyclic hypervalent bromine(III) and chlorine(III) reagents. The groups with strong trans effects, such as -F, -CCH, -CN, -OTf, can help stabilize cyclic hypervalent bromine(III) and chlorine(III) reagents.

**Table 1:** Computational homolytic BDEs (kcal/mol) of cyclic hypervalent halogen reagents.

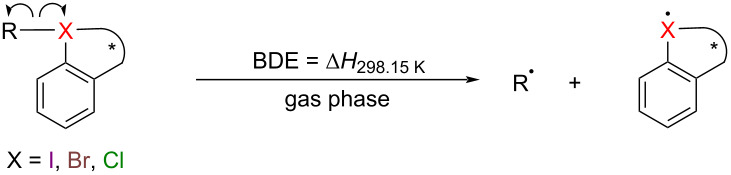																

		**1-X**			**2-X**			**3-X**			**4-X**			**5-X**		

R		X = I	X = Br	X = Cl	X = I	X = Br	X = Cl	X = I	X = Br	X = Cl	X = I	X = Br	X = Cl	X = I	X = Br	X = Cl

a	F	78.6	50.5	30.5	81.9	56.6	35.5	80.8	54.3	33.3	80.6	61.4	29.4	84.0	62.0	42.9
b	Cl	55.3	29.4	10.1	58.1	35.6	14.0	57.4	33.1	12.1	57.1	40.9	10.7	60.1	40.3	21.3
c	Br	44.7	19.8	0.2	47.3	24.9	4.3	46.7	23.0	2.4	46.5	31.2	2.0	49.3	30.2	11.4
d	CH_3_	33.2	13.4	−0.3	42.4	28.7	16.5	42.8	29.6	20.0	49.1	49.3	40.0	39.9	28.9	22.2
e	CF_3_	33.5	13.5	−0.8	39.0	24.5	11.8	38.5	23.6	12.5	41.2	39.0	25.1	37.2	24.5	12.6
f	CHCH_2_	40.4	21.4	8.7	49.6	36.5	25.8	49.6	36.8	28.1	56.0	58.2	47.1	46.8	36.1	27.0
g	CCH	66.1	42.0	24.5	73.1	53.9	38.2	72.6	53.0	39.2	76.3	68.5	48.5	71.1	53.8	39.0
h	CN	68.8	43.6	24.2	72.6	51.6	33.4	71.6	49.7	32.7	72.8	61.6	37.9	71.9	53.0	34.8
i	N_3_	32.1	7.6	−11.2	35.8	14.6	−4.5	34.8	12.3	−6.3	36.3	23.3	−4.8	36.5	17.9	0.1
j	NH_2_	37.7	12.6	−4.3	45.5	25.1	9.6	45.0	24.1	8.3	49.4	40.2	16.7	44.5	27.5	11.7
k	NHAc	47.6	22.0	2.6	53.4	31.6	13.0	52.7	30.0	12.2	55.8	43.4	18.8	53.0	33.7	15.7
l	OH	53.0	26.4	6.5	58.7	35.7	15.9	57.7	33.5	14.1	60.1	44.9	16.3	58.7	38.3	19.5
m	OCH_3_	40.7	15.3	−3.5	46.2	24.6	6.1	45.2	22.4	4.3	47.9	34.5	7.7	46.2	26.9	9.1
n	OCF_3_	62.9	37.0	18.4	64.4	40.5	19.8	63.5	38.1	17.4	62.7	45.0	13.3	67.0	46.6	28.6
o	OCOCH_3_	54.6	27.4	7.5	58.1	33.7	12.3	57.5	31.1	9.9	57.6	39.3	8.6	59.1	37.1	17.4
p	OCOCF_3_	62.3	36.1	17.4	63.5	39.0	18.0	62.7	36.7	15.8	61.2	43.4	11.3	65.9	45.1	27.0
q	OCOPh	55.8	28.5	8.7	58.9	33.8	12.4	58.4	31.8	10.4	58.4	40.5	9.0	60.1	38.0	19.1
r	OTf	66.3	41.9	25.8	65.1	41.3	21.3	64.3	39.0	18.7	62.0	43.6	11.1	69.8	50.8	35.4
s	OTs	61.8	36.5	18.4	62.4	38.4	18.3	62.3	36.2	15.5	61.0	43.0	12.9	65.8	45.8	28.9
t	SCF_3_	40.1	16.1	−3.1	44.6	23.3	4.0	43.2	21.6	3.0	44.9	33.7	14.0	44.2	26.2	7.9

In addition, we also calculated the heterolytic BDEs of cyclic hypervalent halogen reagents [46–47] to comprehensively examine the strength of chemical bonds (Table 2). Geometry optimizations and single point energy calculations for heterolytic BDEs are performed using M06-2X/def2-TZVPP in the SMD (acetonitrile) Implicit solvent model at 298.15 K. Due to the instability of some transfer group cations, such as ^+^OCH_3_, ^+^OCF_3_, ^+^OCOCF_3_, ^+^OCOPh, ^+^OTf and ^+^SCF_3_, it is difficult for us to investigate their heterolytic BDEs. From Table 2, it can be seen that, except for CF_3_ and CHCH_2_, all other transfer groups exhibit high heterolytic BDEs with hypervalent halogen centers.

**Table 2:** Computational heterolytic BDEs (kcal/mol) of cyclic hypervalent halogen reagents.

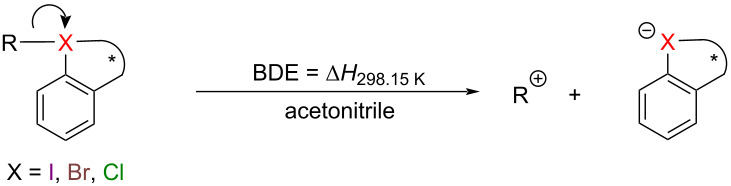																

		**1-X**			**2-X**			**3-X**			**4-X**			**5-X**		

R		X = I	X = Br	X = Cl	X = I	X = Br	X = Cl	X = I	X = Br	X = Cl	X = I	X = Br	X = Cl	X = I	X = Br	X = Cl

a	F	375.2	346.9	327.5	354.4	322.1	301.1	345.0	309.7	290.0	324.1	286.4	263.3	353.0	321.5	303.1
b	Cl	239.5	214.1	195.2	217.7	188.5	166.9	208.4	176.3	156.5	187.6	154.8	134.6	216.4	188.0	170.2
c	Br	212.7	188.4	169.0	190.8	163.3	142.0	181.6	151.3	131.9	160.7	130.9	111.9	189.5	162.2	143.8
d	CH_3_	83.8	66.1	55.6	74.0	57.4	47.9	67.9	51.4	47.8	58.6	44.5	43.0	66.2	48.7	43.4
e	CF_3_	77.5	56.5	42.1	61.7	40.4	29.3	53.7	30.9	24.2	37.8	19.8	18.9	55.2	32.0	20.9
f	CHCH_2_	71.2	54.2	43.9	60.3	44.9	37.7	54.2	38.4	34.9	44.0	28.8	29.3	53.0	35.9	30.7
g	CCH	231.7	207.2	189.8	216.5	192.5	178.7	208.7	183.3	174.1	194.4	171.4	163.5	210.1	183.8	171.0
h	CN	254.7	227.8	207.7	235.6	206.1	189.3	226.6	194.4	181.2	207.8	177.1	166.7	231.0	200.5	181.2
i	N_3_	159.6	134.1	115.4	139.5	111.3	92.2	130.4	99.5	83.4	111.6	81.9	68.2	136.6	107.7	89.5
j	NH_2_	145.5	121.4	103.8	133.0	108.4	94.0	124.9	99.2	89.9	111.7	87.7	79.7	125.5	99.9	86.9
k	NHAc	113.6	87.8	68.3	97.6	71.5	55.2	89.3	61.4	49.0	73.5	47.9	37.6	92.2	64.2	47.2
l	OH	244.1	217.6	197.1	227.3	199.1	179.6	218.4	187.7	170.9	200.9	170.3	154.0	222.9	193.6	174.0
m	OCH_3_	–	–	–	–	–	–	–	–	–	–	–	–	–	–	–
n	OCF_3_	–	–	–	–	–	–	–	–	–	–	–	–	–	–	–
o	OCOCH_3_	149.1	122.7	102.9	128.7	100.4	78.1	119.6	87.4	67.7	99.4	67.8	47.0	127.0	97.5	77.2
p	OCOCF_3_	–	–	–	–	–	–	–	–	–	–	–	–	–	–	–
q	OCOPh	–	–	–	–	–	–	–	–	–	–	–	–	–	–	–
r	OTf	–	–	–	–	–	–	–	–	–	–	–	–	–	–	–
s	OTs	104.8	79.7	62.4	80.2	50.0	29.1	70.9	37.0	18.5	47.8	13.2	-7.6	/	52.5	37.9
t	SCF_3_	–	–	–	–	–	–	–	–	–	–	–	–	–	–	–

**Figure 3:**
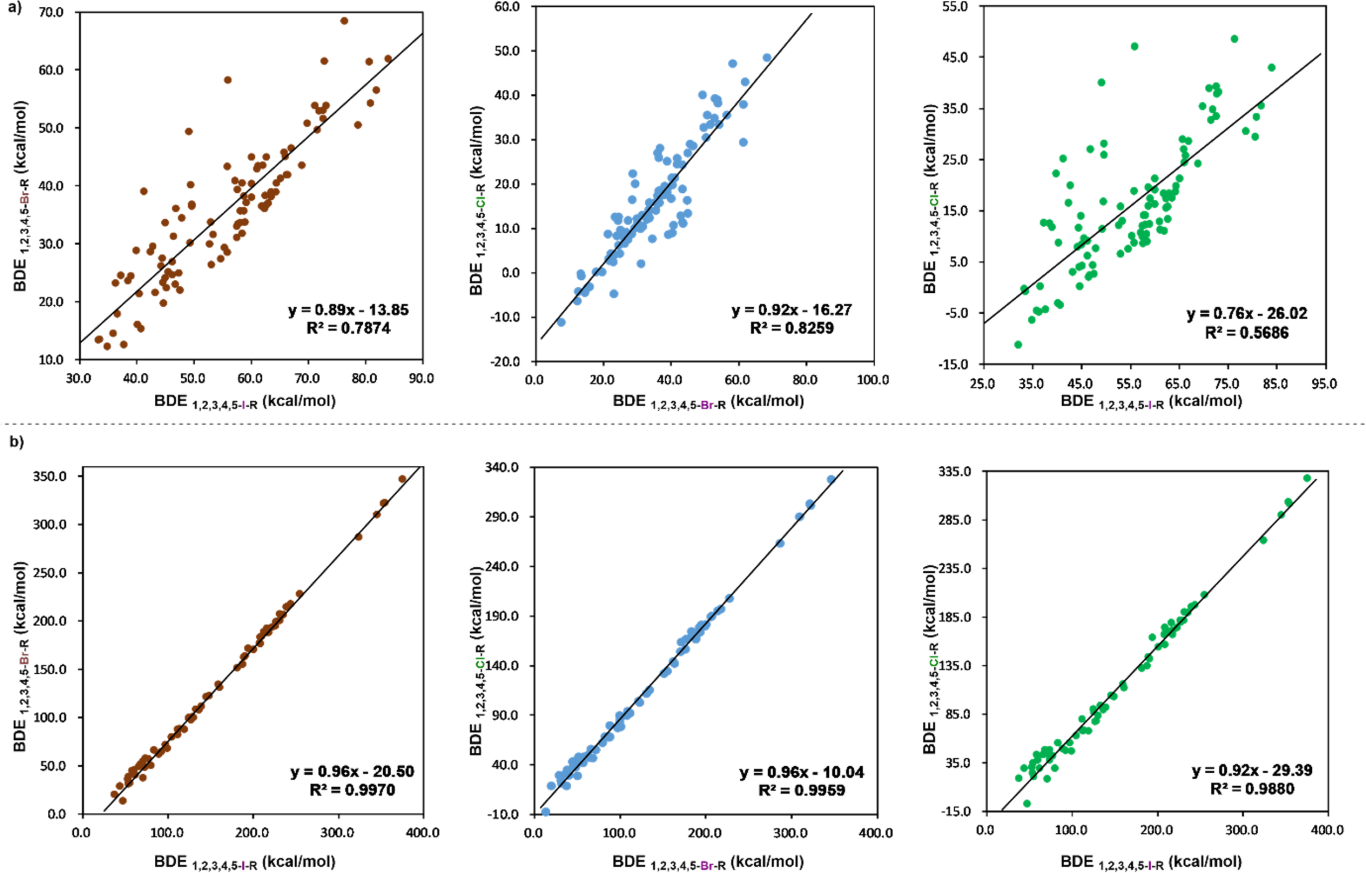
a) Linear dependence between the homolytic BDEs of cyclic hypervalent halogen reagents; b) linear dependence between the heterolytic BDEs of cyclic hypervalent halogen reagents.

To elucidate the relationships between halogen centers and their corresponding homolytic BDEs, the homolytic BDEs of cyclic hypervalent halogen reagents were plotted against those of reagents with different halogen centers, giving moderate linear relationships (Figure 3a). For heterolytic BDEs, we found a strong linear relationship between different halogen centers, as illustrated in Figure 3b. This indicates that based on any kind of cyclic hypervalent halogen reagents, we can obtain a rough estimation of the BDEs for others with different halogen centers.

**Figure 4:**
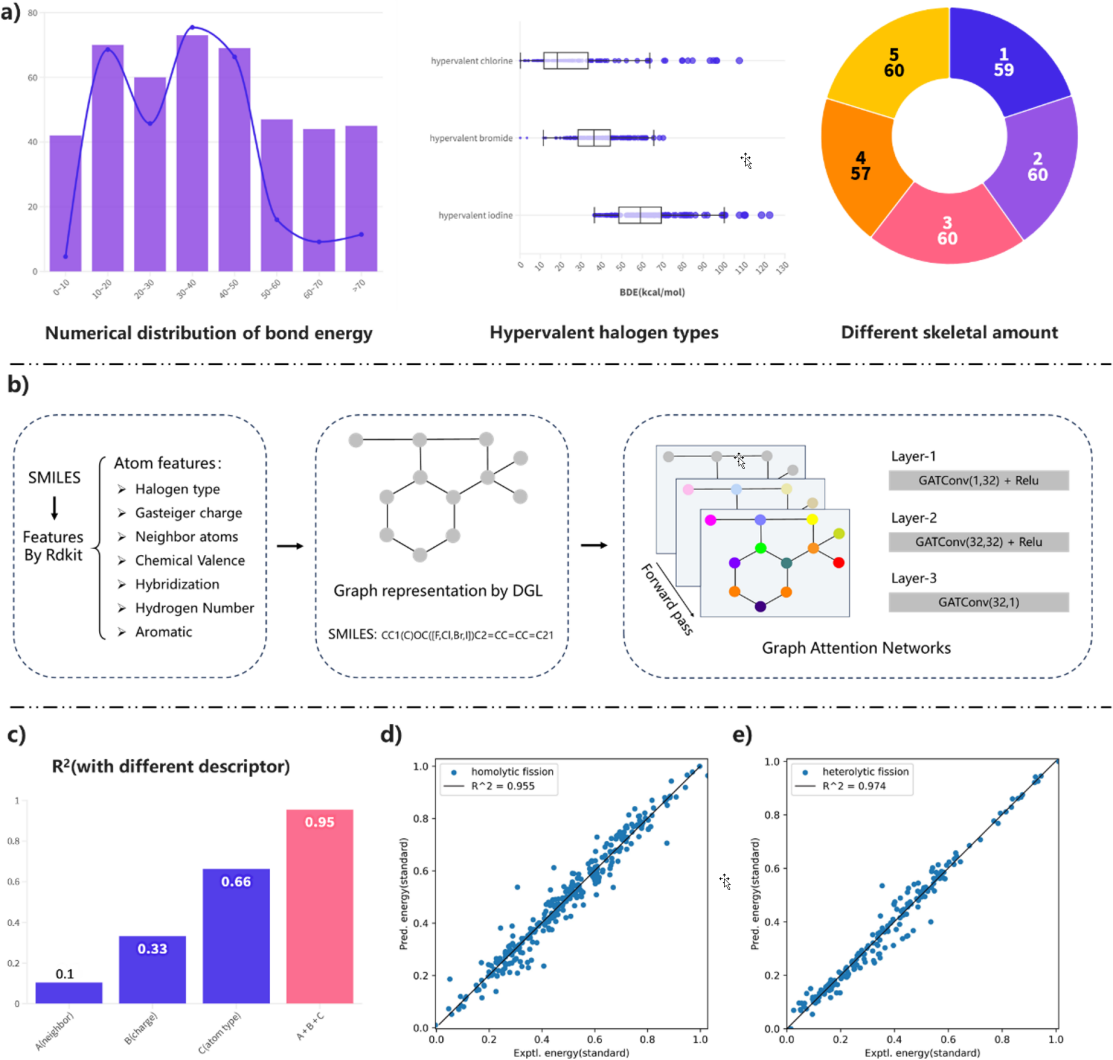
a) Composition and distribution of homolytic BDE dataset; b) graph attention network (GAT) model architecture and workflow; c) comparison of prediction performance on the training set using different descriptors (see more details in Supporting Information File 1); d) prediction performance for homolytic BDEs on the test set; e) prediction performance for heterolytic BDEs on the test set.

With these homolytic and heterolytic BDEs in hand, we next attempted to develop a predictive model for BDEs of hypervalent halogen compounds using machine learning algorithms. Graph attention network (GAT) [85] embeds local chemical environment information into the graph network by taking atomic information as node inputs, thus achieving higher predictive capabilities [86]. Building upon the computational studies, we constructed two compound datasets separately, consisting of 296 homolytic BDE data points and 209 heterolytic BDE data points. Taking homolytic BDE datasets as an example (Figure 4a), the distribution of this dataset is illustrated with key bond energy values normalized using min–max scaling. This approach ensures both data consistency and improves training efficiency.

We used the GAT model as the core framework, incorporating ten selected atomic descriptors as local information within the graph structure. Effective molecular transformations into molecular graphs (Figure 4b) were achieved using the RDKit and Deep Graph Library [87]. The dataset was randomly divided into training and testing sets in a 9:1 ratio. Notably, our analysis of descriptor testing revealed that individual inputs, such as neighboring atomic information, atomic charge, and atomic species, did not yield satisfactory results. However, combining all three inputs resulted in highly effective predictions (Figure 4c, see Supporting Information File 1 for detail). The R^2^, MAE, and RMSE metrics exhibited outstanding performance. The final predictive results yielded excellent performance with an R^2^ value of 0.955 for homolytic BDEs (Figure 4d) and an R^2^ value of 0.974 for heterolytic BDEs (Figure 4e). Furthermore, we achieved superior predictive results by not distinguishing between halogen categories in the dataset. This approach is reliable and efficient in assisting chemists in estimating the bond energy ranges of novel cyclic hypervalent halogen reagents.

We conducted additional tests with cyclic hypervalent halogen reagents beyond the training set, employing linear dependence equations and the GAT model for predictions (Table 3). The comparison of the two methods reveals that the GAT model is more reliable, as indicated by the lower root mean square error (RMSE). Moreover, the linear dependence method requires the BDEs of known cyclic hypervalent iodine(III) reagents to deduce the BDEs of the cyclic hypervalent bromine(III) and chlorine(III) reagents. In contrast, the GAT model is more straightforward, relying solely on structural information. Therefore, the GAT model is a superior method to predict the BDEs of cyclic hypervalent halogen reagents.

**Table 3:** Predictional BDEs of cyclic hypervalent halogen reagents.

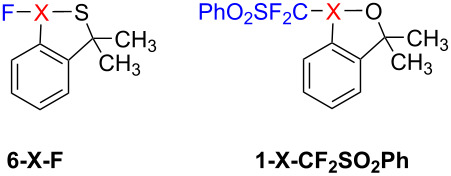															

homolytic BDEs								heterolytic BDEs							

methods	**6-X-F**			**1-X-CF** **_2_** **SO** **_2_** **Ph**			RMSE	methods	**6-X-F**			**1-X-CF** **_2_** **SO** **_2_** **Ph**			RMSE
	X = I	X = Br	X = Cl	X = I	X = Br	X = Cl			X = I	X = Br	X = Cl	X = I	X = Br	X = Cl	

DFT^a^	68.3	44.1	26.2	32.6	10	−6.2	–	DFT^b^	367.4	342.7	328.8	79.9	57.6	41.7	–
LE^c^	–	47	26.1	–	15.2	−1.2	3.9	LE^d^	–	332.2	308.6	–	56.2	44.1	11.4
ML^e^	72.7	45.2	27.2	38.1	13.3	−4.5	3.3	ML^e^	375.8	336.8	329.1	86.3	56.6	48.9	5.7

^a^DFT calculations: M06-2X/def2-TZVPP in gas phase; ^b^DFT calculations: M06-2X/def2-TZVPP in SMD (acetonitrile); ^c^linear dependence equations: these predicted BDEs for hypervalent bromine and hypervalent chlorine are obtained by inserting the calculated hypervalent iodine BDEs into the linear dependence equations: y = 0.89x−13.85 and y = 0.76x−26.02; ^d^linear dependence equations: y = 0.96x−20.50 and y = 0.92x−29.39; ^e^machine learning.

## Conclusion

We have undertaken an extensive computational investigation into the BDEs of cyclic hypervalent halogen reagents. Leveraging this dataset, we have developed a predictive model for both homolytic and heterolytic BDEs of hypervalent halogen compounds employing a graph attention network. We anticipate that the findings from our research will aid the design and development of new hypervalent bromine(III) and chlorine(III) reagents, an area that remains largely underexplored.

## Supporting Information

File 1 Machine learning details and calculation data.

## Data Availability

The data that supports the findings of this study is available from the corresponding author upon reasonable request.
