# Supplementary material for: Predicting bond dissociation energies of cyclic hypervalent halogen reagents using DFT calculations and graph attention network model
Source: Beilstein J Org Chem. 2024 Jun 28;20:1444–52. doi: 10.3762/bjoc.20.127 (PMC11216094; doi:10.3762/bjoc.20.127)
Supplement: File 1 — Machine learning details and calculation data. [file Beilstein_J_Org_Chem-20-1444-s001.pdf]

BEILSTEIN JOURNAL OF ORGANIC CHEMISTRY

Supporting Information

for

Predicting bond dissociation energies of cyclic hypervalent halogen reagents using DFT calculations and graph attention network model

Yingbo Shao, Zhiyuan Ren, Zhihui Han, Li Chen, Yao Li and Xiao-Song Xue

Beilstein J. Org. Chem. **2024,** *20,* 1444–1452. doi:10.3762/bjoc.20.127

Machine learning details and calculation data

License and Terms: This is a supporting information file under the terms of the Creative Commons Attribution License (https://creativecommons.org/licenses/by/4.0). Please note that the reuse, redistribution and reproduction in particular requires that the author(s) and source are credited and that individual graphics may be subject to special legal provisions.
The license is subject to the *Beilstein Journal of Organic Chemistry* terms and conditions: (https://www.beilstein-journals.org/bjoc/terms)

Table of contents

Testing of different computational methods S2

ML model details S3

Descriptor selection and comparison S4

Numerical comparison of model predictions with calculated values

and assessment results S6

Cartesian coordinates and energies of optimized compounds S13

Testing of different computational methods

Table S1: Computational BDEs (kcal/mol) of halides using different methods

expt.	Compounds	M06-2X/def2-TZVPP	M06-2X/def2-QZVPP	DLPNO-CCSD(T)/def2-QZVPP	wB97X-2/def2-QZVPP
104.1	Cl-CCH	112.2	111.9	109.7	114.0
88.7	Cl-CF3	87.1	86.7	86.4	85.7
94.2	Cl-CHCH2	95.5	95.3	93.9	95.5
101.0	Cl-CN	107.7	107.5	102.1	107.6
84.2	Cl-Et	85.4	85.2	83.9	84.5
83.7	Cl-Me	85.1	84.9	82.8	83.5
55.8	Cl-OH	57.1	57.4	54.4	55.8
97.1	Cl-Ph	97.3	97.1	96.4	99.0
98.1	Br-CCH	97.6	97.1	98.9	102.9
70.8	Br-CF3	70.3	69.7	73.0	72.4
79.6	Br-CHCH2	80.6	80.1	82.4	83.9
87.2	Br-CN	93.7	93.2	91.7	96.9
70.2	Br-Et	71.6	71.0	73.5	73.8
70.3	Br-Me	71.7	71.2	72.7	73.2
50.1	Br-OH	50.5	50.6	52.2	53.2
83.5	Br-Ph	82.2	81.7	85.0	87.6
78.8	I-CCH	83.9	83.8	90.9	94.7
54.3	I-CF3	52.7	52.4	60.0	59.3
64.8	I-CHCH2	64.2	64.0	71.2	72.5
76.5	I-CN	80.7	80.7	84.1	89.2
56.3	I-Et	55.6	55.5	62.7	62.8
56.9	I-Me	56.6	56.4	62.6	62.9
51.0	I-OH	46.1	47.2	53.2	54.3
67.4	I-Ph	65.5	65.4	73.9	76.3
0.0	MAE	2.3	2.2	3.5	5.1

The BDEs of hypervalent halogen reagents lack experimental values, so we used experimental BDEs of halides to test computational methods, and the experimental BDEs are retrieved from *i*BonD. All geometry optimizations were performed at M06-2X/def2-TZVPP. We tested single point energies with different functionals and basis sets, and the results are shown in the Table S1. The M06-2X functional has good accuracy, and the results of def2-TZVPP and def2-QZVPP are similar. Although the MAE of M06-2X/def2-QZVPP is slightly smaller, we chose the M06-2X/def2-TZVPP method to complete the calculation considering the computational cost.

ML model details

All structural information is converted from .mol files to SMILES using RDKit. Bond energy data is transformed through min-max normalization, with a conversion range of [0, 1]. After being recognized as valid molecular structures by RDKit, a total of 296 valid data entries are input into the model. The training set and test set are randomly divided from the database in a (9:1) ratio. To evaluate the performances of the proposed methods, we applied four evaluation metrics for evaluation, mean absolute error (MAE), the root mean squared error (RMSE), R^2 value (where R is the Pearson correlation coefficient), and the Spearman's coefficient of correlation (CC) .

The computational code pertaining to machine learning models is scripted in the Python programming language. The detailed information about the software packages involved in the computational process is as follows.

- Python 3.9.18
- Scikit-learn 1.2.1
- RDKit 2022.09.5
- PyTorch 2.1.1+cu118
- DGL 1.1.2-cu118
- Pandas 2.1.3

Descriptor selection and comparison

A descriptor stands for:

atom_features += [len(atom.GetNeighbors())]
atom_features += [atom.GetTotalNumHs()]
TPSAs = Chem.rdMolDescriptors._CalcTPSAContribs(mol)
Training result for 500 epoch:

BestEpoch	TrainMAE	TrainRMSE	Train R^2	TrainCC	TestMAE	TestRMSE	Test R^2	TestCC
500	0.164	0.202	**0.104**	0.308	0.161	0.193	0.115	0.476

B descriptor stands for:

atom_features += [float(atom.GetProp('_GasteigerCharge'))]
atom_features += one_of_k_encoding(atom.GetDegree(), [0, 1, 2, 3, 4, 5, 6])
atom_features += one_of_k_encoding_unk(atom.GetFormalCharge(), [-1, 0, 1])
atom_features += [atom.GetIsAromatic()]
Training result for 500 epoch:

BestEpoch	TrainMAE	TrainRMSE	Train R^2	TrainCC	TestMAE	TestRMSE	Test R^2	TestCC
500	0.146	0.174	**0.332**	0.537	0.159	0.185	0.182	0.426

C descriptor stands for:

possible_atoms = ['Cl', 'Br', 'I']
atom_features += one_of_k_encoding_unk(atom.GetSymbol(), possible_atoms)
atom_features += one_of_k_encoding_unk(atom.GetHybridization(),
[Chem.rdchem.HybridizationType.SP, Chem.rdchem.HybridizationType.SP2,
Chem.rdchem.HybridizationType.SP3, Chem.rdchem.HybridizationType.SP3D])
Training result for 500 epoch:

BestEpoch	TrainMAE	TrainRMSE	Train R^2	TrainCC	TestMAE	TestRMSE	TestR^2	TestCC
496	0.085	0.124	**0.663**	0.842	0.091	0.116	0.678	0.871

A + B + C training result for 500 epoch:

BestEpoch	TrainMAE	TrainRMSE	Train R^2	TrainCC	TestMAE	TestRMSE	TestR2	TestCC
500	0.030	0.043	**0.959**	0.977	0.040	0.054	0.930	0.956

Numerical comparison of model predictions with calculated values and assessment results

Homolytic BDEs:

Serial	Calculated value(normalized)	Predicted value(normalized)
0	0.943	0.89
1	0.699	0.661
2	0.587	0.539
3	0.466	0.523
4	0.469	0.557
5	0.542	0.511
6	0.812	0.817
7	0.84	0.823
8	0.514	0.549
9	0.618	0.609
10	0.674	0.603
11	0.545	0.523
12	0.778	0.74
13	0.691	0.639
14	0.772	0.72
15	0.704	0.645
16	0.814	0.729
17	0.767	0.716
18	0.539	0.54
19	0.978	0.97
20	0.728	0.724
21	0.615	0.601
22	0.563	0.6
23	0.527	0.537
24	0.639	0.588
25	0.885	0.885
26	0.88	0.885
27	0.494	0.506
28	0.596	0.629
29	0.679	0.679
30	0.734	0.683
31	0.603	0.603
32	0.794	0.802
33	0.728	0.701
34	0.785	0.782
35	0.736	0.708
36	0.801	0.791
37	0.773	0.778
38	0.586	0.603
39	0.966	0.95
41	0.608	0.58
42	0.567	0.614
43	0.522	0.551
44	0.639	0.602
45	0.88	0.865
46	0.87	0.817
47	0.483	0.438
48	0.59	0.609
49	0.671	0.659
50	0.724	0.663
51	0.592	0.583
52	0.785	0.781
53	0.722	0.68
54	0.776	0.761
55	0.731	0.687
56	0.793	0.77
57	0.772	0.757
58	0.571	0.534
59	0.964	1.028
60	0.717	0.707
61	0.606	0.584
62	0.633	0.665
63	0.55	0.602
64	0.706	0.873
65	0.919	0.943
66	0.882	0.908
67	0.637	0.688
68	0.704	0.738
69	0.749	0.741
70	0.621	0.661
71	0.776	0.776
72	0.723	0.717

73	0.76	0.803	117	0.287	0.326
74	0.731	0.767	118	0.712	0.719
75	0.769	0.812	119	0.492	0.478
76	0.758	0.803	120	0.379	0.356
77	0.589	0.586	121	0.419	0.437
78	1	0.998	122	0.375	0.374
79	0.749	0.711	123	0.501	0.55
80	0.635	0.589	124	0.684	0.693
81	0.537	0.67	125	0.66	0.64
82	0.508	0.606	126	0.271	0.261
83	0.609	0.658	127	0.381	0.425
84	0.865	0.867	128	0.45	0.48
85	0.873	0.872	129	0.493	0.462
86	0.501	0.493	130	0.376	0.366
87	0.585	0.657	131	0.543	0.525
88	0.674	0.658	132	0.472	0.456
89	0.734	0.711	133	0.527	0.505
90	0.603	0.63	134	0.473	0.463
91	0.821	0.79	135	0.551	0.514
92	0.738	0.688	136	0.521	0.501
93	0.81	0.77	137	0.362	0.357
94	0.749	0.695	138	0.688	0.699
95	0.851	0.875	139	0.465	0.477
96	0.809	0.766	140	0.359	0.354
97	0.582	0.59	141	0.429	0.436
98	0.648	0.665	142	0.365	0.372
99	0.426	0.447	143	0.504	0.53
100	0.326	0.325	144	0.674	0.673
101	0.258	0.36	145	0.64	0.644
102	0.26	0.296	146	0.247	0.259
103	0.342	0.348	147	0.371	0.405
104	0.559	0.614	148	0.433	0.46
105	0.576	0.609	149	0.469	0.442
106	0.198	0.23	150	0.353	0.346
107	0.25	0.345	151	0.518	0.556
108	0.349	0.348	152	0.444	0.454
109	0.395	0.382	153	0.503	0.536
110	0.278	0.286	154	0.452	0.461
111	0.506	0.526	155	0.527	0.545
112	0.406	0.425	156	0.498	0.532
113	0.497	0.506	157	0.344	0.356
114	0.417	0.431	158	0.763	0.777
115	0.558	0.515	159	0.547	0.537
116	0.501	0.502	160	0.445	0.415

161	0.635	0.585	205	0.01	0.004
162	0.527	0.432	206	0.072	0.046
163	0.729	0.608	207	0.145	0.161
164	0.837	0.751	208	0.186	0.051
165	0.765	0.722	209	0.081	0.103
166	0.54	0.483	210	0.311	0.298
167	0.574	0.538	211	0.196	0.197
168	0.589	0.52	212	0.3	0.278
169	0.48	0.425	213	0.209	0.203
170	0.59	0.515	214	0.389	0.287
171	0.53	0.514	215	0.311	0.274
172	0.574	0.596	216	0.085	0.093
173	0.543	0.541	217	0.49	0.451
174	0.576	0.617	218	0.265	0.302
175	0.569	0.603	219	0.163	0.18
176	0.472	0.416	220	0.291	0.261
177	0.769	0.747	221	0.242	0.197
178	0.541	0.532	222	0.389	0.387
179	0.435	0.409	223	0.519	0.517
180	0.421	0.491	224	0.469	0.469
181	0.375	0.427	225	0.07	0.085
182	0.497	0.478	226	0.218	0.235
183	0.683	0.721	227	0.254	0.253
184	0.674	0.693	228	0.285	0.261
185	0.306	0.314	229	0.182	0.171
186	0.406	0.453	230	0.326	0.381
187	0.472	0.479	231	0.247	0.28
188	0.52	0.49	232	0.307	0.315
189	0.4	0.394	233	0.248	0.286
190	0.607	0.61	234	0.341	0.324
191	0.507	0.509	235	0.31	0.31
192	0.591	0.591	236	0.16	0.181
193	0.517	0.516	237	0.467	0.431
194	0.651	0.628	238	0.245	0.275
195	0.599	0.587	239	0.143	0.153
196	0.393	0.411	240	0.328	0.364
197	0.438	0.436	241	0.249	0.17
198	0.224	0.219	242	0.413	0.367
199	0.12	0.091	243	0.529	0.497
200	0.115	0.173	244	0.461	0.449
201	0.109	0.109	245	0.051	0.058
202	0.209	0.307	246	0.205	0.215
203	0.375	0.438	247	0.246	0.233
204	0.372	0.375	248	0.266	0.241

249	0.163	0.151	274	0.253	0.317
250	0.3	0.354			
251	0.222	0.253	275	0.265	0.227
252	0.284	0.269	276	0.568	0.548
253	0.227	0.259	277	0.341	0.33
254	0.314	0.278	278	0.237	0.208
255	0.281	0.265	279	0.351	0.289
256	0.149	0.154	280	0.25	0.226
257	0.426	0.509	281	0.401	0.414
258	0.23	0.348	282	0.527	0.545
259	0.139	0.226	283	0.483	0.492
260	0.538	0.307	284	0.119	0.113
261	0.381	0.243	285	0.24	0.263
262	0.612	0.445	286	0.283	0.278
263	0.627	0.576	287	0.323	0.289
264	0.516	0.527	288	0.213	0.199
265	0.293	0.293	289	0.418	0.409
266	0.315	0.311	290	0.3	0.308
267	0.289	0.319	291	0.401	0.389
268	0.199	0.23	292	0.318	0.314
269	0.257	0.238	293	0.489	0.441
270	0.208	0.251	294	0.421	0.428
271	0.236	0.407	295	0.201	0.209
272	0.212	0.278			
273	0.234	0.324			

TestMSE	TestMAE	TestRMSE	Test R^2	TestCC
0.002	0.031	0.044	0.955	0.976

Heterolytic BDEs:

Serial	Calculated value(normalized)	Predicted value(normalized)	Serial	Calculated value(normalized)	Predicted value(normalized)
0	1.000	1.010	10	0.658	0.627
1	0.645	0.630	11	0.409	0.395
2	0.576	0.562	12	0.294	0.278
3	0.239	0.260	13	0.946	0.944
4	0.222	0.249	14	0.589	0.574
5	0.206	0.206	15	0.518	0.506
6	0.625	0.645	16	0.213	0.225
7	0.685	0.678	17	0.181	0.177
8	0.400	0.535	18	0.177	0.172
9	0.317	0.334	19	0.586	0.583
			20	0.635	0.622
			21	0.384	0.374
			22	0.367	0.470
			23	0.275	0.268

24	0.614	0.562	68	0.579	0.573
25	0.356	0.339	69	0.512	0.505
26	0.230	0.222	70	0.192	0.205
27	0.921	0.920	71	0.168	0.157
28	0.564	0.581	72	0.162	0.151
29	0.494	0.513	73	0.561	0.588
30	0.197	0.213	74	0.615	0.621
31	0.160	0.165	75	0.370	0.373
32	0.161	0.159	76	0.337	0.430
33	0.565	0.559	77	0.249	0.242
34	0.612	0.593	78	0.588	0.538
35	0.360	0.345	79	0.340	0.337
36	0.346	0.446	80	0.228	0.221
37	0.253	0.244	81	0.862	0.859
38	0.591	0.538	82	0.512	0.502
39	0.332	0.345	83	0.447	0.434
40	0.205	0.229	84	0.170	0.170
41	0.867	0.870	85	0.125	0.121
42	0.510	0.494	86	0.137	0.140
43	0.440	0.425	87	0.523	0.522
44	0.173	0.161	88	0.558	0.550
45	0.119	0.113	89	0.311	0.302
46	0.135	0.198	90	0.303	0.365
47	0.528	0.509	91	0.207	0.201
48	0.563	0.546	92	0.540	0.473
49	0.312	0.396	93	0.282	0.267
50	0.212	0.194	94	0.150	0.089
51	0.545	0.488	95	0.829	0.834
52	0.280	0.281	96	0.480	0.489
53	0.145	0.160	97	0.415	0.420
54	0.942	0.924	98	0.154	0.156
55	0.585	0.555	99	0.101	0.108
56	0.515	0.486	100	0.120	0.116
57	0.193	0.222	101	0.499	0.498
58	0.164	0.174	102	0.528	0.538
59	0.158	0.168	103	0.280	0.288
60	0.569	0.569	104	0.279	0.340
61	0.624	0.603	105	0.180	0.177
62	0.377	0.355	106	0.510	0.448
63	0.348	0.449	107	0.248	0.253
64	0.261	0.259	108	0.117	0.163
65	0.602	0.542	109	0.768	0.785
66	0.352	0.319	110	0.424	0.432
67	0.926	0.932	111	0.362	0.363

112	0.136	0.138	156	0.487	0.482
113	0.072	0.051	157	0.514	0.523
114	0.095	0.066	158	0.261	0.287
115	0.468	0.448	159	0.265	0.346
116	0.482	0.488	160	0.164	0.170
117	0.249	0.291	161	0.489	0.422
118	0.145	0.127	162	0.224	0.252
119	0.465	0.399	163	0.096	0.055
120	0.197	0.227	164	0.777	0.769
121	0.054	0.095	165	0.429	0.445
122	0.860	0.838	166	0.364	0.376
123	0.511	0.498	167	0.145	0.141
124	0.444	0.429	168	0.083	0.064
125	0.147	0.165	169	0.111	0.095
126	0.103	0.117	170	0.475	0.458
127	0.114	0.111	171	0.493	0.499
128	0.500	0.501	172	0.238	0.244
129	0.544	0.545	173	0.255	0.322
130	0.301	0.297	174	0.148	0.146
131	0.281	0.344	175	0.466	0.398
132	0.187	0.202	176	0.197	0.209
133	0.526	0.452	177	0.068	0.053
134	0.275	0.262	178	0.708	0.719
135	0.157	0.146	179	0.371	0.406
136	0.876	0.875	180	0.312	0.338
137	0.530	0.516	181	0.132	0.074
138	0.461	0.452	182	0.069	0.025
139	0.165	0.188	183	0.096	0.046
140	0.130	0.140	184	0.447	0.408
141	0.135	0.185	185	0.455	0.449
142	0.516	0.548	186	0.228	0.273
143	0.562	0.569	187	0.118	0.096
144	0.321	0.321	188	0.422	0.348
145	0.291	0.319	189	0.143	0.138
146	0.198	0.225	190	0.000	0.015
147	0.535	0.354	191	0.812	0.814
148	0.289	0.280	192	0.465	0.454
149	0.183	0.164	193	0.396	0.386
150	0.806	0.793	194	0.133	0.121
151	0.456	0.487	195	0.074	0.073
152	0.391	0.419	196	0.100	0.099
153	0.145	0.155	197	0.467	0.462
154	0.096	0.106	198	0.493	0.502
155	0.118	0.120	199	0.254	0.254

200	0.247	0.326	203	0.222	0.218
201	0.143	0.158	204	0.119	0.106
202	0.474	0.401			

TestMSE	TestMAE	TestRMSE	Test R^2	TestCC
0.001	0.022	0.034	**0.974**	0.985

Composition and distribution of heterolytic BDE dataset:

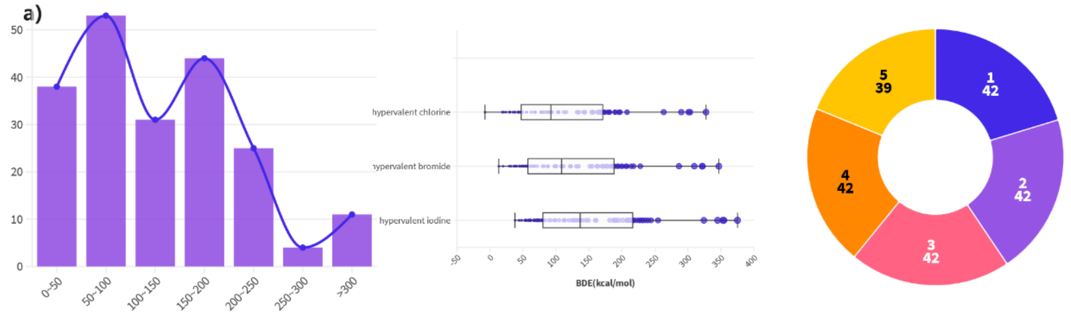

Numerical distribution of bond energy Hypervalent halogen types Different skeletal amount

Cartesian coordinates and energies of optimized compounds

geometry optimizations and single point energy calculations are both performed using M06-2X/def2-TZVPP in gas phase at 298.15 K by Gaussian 16 Rev. A.03. Frequency calculations confirmed that optimized structures are minima (no imaginary frequency).

1-BrIII-Br

C	-2.93446	-1.10859	0.00015
C	-2.52243	0.21846	0.00015
C	-1.16116	0.50593	0.00000
C	-0.29395	-0.55644	-0.00016
C	-0.62935	-1.88835	-0.00026
C	-2.00137	-2.14559	-0.00009
H	-3.99148	-1.34315	0.0003
H	-3.22289	1.04544	0.00026
H	0.12474	-2.66268	-0.00066
H	-2.33707	-3.17535	-0.00015
Br	1.57797	0.06966	0.00005
O	0.73007	1.89152	-0.00025
C	-0.59322	1.89656	-0.00007
C	-1.01634	2.67844	1.2574
C	-1.01649	2.67857	-1.2574
H	-0.94281	2.04221	2.11455
H	-2.02689	3.01224	1.14662
H	-2.04465	2.9622	-1.17173
H	-0.37331	3.52413	1.38474
H	-0.41123	3.5558	-1.35241
H	-0.88769	2.06105	-2.12168
Br	2.14787	-1.78352	0.00019

Sum of electronic and zero-point Energies=-5572.316279
Sum of electronic and thermal Energies= -5572.304189
Sum of electronic and thermal Enthalpies= -5572.303245
Sum of electronic and thermal Free Energies= -5572.355610

1-BrIII-CCH

C	-2.04035	2.84698	0.05489
C	-2.5659	1.56311	0.11017
C	-1.72726	0.46123	0.01266
C	-0.37785	0.7091	-0.1241
C	0.19157	1.95846	-0.17863
C	-0.67465	3.04314	-0.09188
H	-2.70165	3.69963	0.12404
H	-3.63161	1.41448	0.22532
H	1.25822	2.08618	-0.28069
H	-0.26946	4.04403	-0.14095
Br	0.61073	-0.9639	-0.22502
O	-1.11851	-1.7315	-0.54841
C	-2.14837	-0.99464	0.08134
C	-3.42086	-1.26546	-0.71001
C	-2.3016	-1.41942	1.54222
H	-3.3038	-0.92901	-1.73841
H	-4.27435	-0.75844	-0.25949
H	-3.08641	-0.84221	2.03169
H	-3.61736	-2.33653	-0.70891
H	-2.55741	-2.47759	1.5843
H	-1.37045	-1.26233	2.08775
C	3.50893	0.60328	-0.42291
H	4.44663	1.11586	-0.47663
C	2.45626	0.02786	-0.36262

Sum of electronic and zero-point Energies=-3074.794451
Sum of electronic and thermal Energies= -3074.781512
Sum of electronic and thermal Enthalpies= -3074.780568
Sum of electronic and thermal Free Energies= -3074.834032

1-BrIII-CF3

C	-2.69011	2.28973	0.00005
C	-2.95457	0.92757	0.0001
C	-1.91235	-0.00045	0.00001
C	-0.64991	0.52412	-0.00006
C	-0.30538	1.85846	-0.00013
C	-1.37667	2.75141	-0.00009
H	-3.50769	2.99983	0.00013
H	-3.96181	0.52596	0.00018
H	0.70804	2.22727	-0.00024
H	-1.16527	3.81354	-0.00016
Br	0.77117	-0.94889	0.00007
O	-1.13277	-2.20926	-0.00037
C	2.40402	0.26982	0.00001
F	3.4394	-0.54682	0.00069
F	2.44547	1.03228	1.07774
F	2.44614	1.03133	-1.07837
C	-2.19866	-1.51874	-0.00005
C	-2.95724	-1.98293	-1.2573
C	-2.95672	-1.98296	1.25751
H	-2.97973	-3.05231	-1.28578
H	-3.95818	-1.60575	-1.22951
H	-2.46089	-1.61324	-2.13015
H	-2.99293	-3.05215	1.27777
H	-3.9528	-1.59266	1.23792
H	-2.45112	-1.62661	2.1306

Sum of electronic and zero-point Energies=-3335.743687
Sum of electronic and thermal Energies= -3335.729276
Sum of electronic and thermal Enthalpies= -3335.728332
Sum of electronic and thermal Free Energies= -3335.785414

1-BrIII-CH3

C	-2.69011	2.28973	0.00005
C	-2.95457	0.92757	0.0001
C	-1.91235	-0.00045	0.00001
C	-0.64991	0.52412	-0.00006
C	-0.30538	1.85846	-0.00013
C	-1.37667	2.75141	-0.00009
H	-3.50769	2.99983	0.00013
H	-3.96181	0.52596	0.00018
H	0.70804	2.22727	-0.00024
H	-1.16527	3.81354	-0.00016
Br	0.77117	-0.94889	0.00007
O	-1.13277	-2.20926	-0.00037
C	2.40402	0.26982	0.00001
C	-2.19866	-1.51874	-0.00005
C	-2.95724	-1.98293	-1.2573
C	-2.95672	-1.98296	1.25751
H	-2.97973	-3.05231	-1.28578
H	-3.95818	-1.60575	-1.22951
H	-2.46089	-1.61324	-2.13015
H	-2.99293	-3.05215	1.27777
H	-3.9528	-1.59266	1.23792
H	-2.45112	-1.62661	2.1306
H	2.4376	0.8875	0.87308
H	3.24415	-0.39281	0.00056
H	2.43814	0.88672	-0.87359

Sum of electronic and zero-point Energies=-3037.949644
Sum of electronic and thermal Energies= -3037.937343
Sum of electronic and thermal Enthalpies= -3037.936399
Sum of electronic and thermal Free Energies= -3037.988264

1-BrIII-CHCH2

C	-1.47784	2.86145	0.03034
C	-2.0225	1.58679	0.05887
C	-1.20342	0.46278	-0.00179
C	0.14941	0.69073	-0.073
C	0.74099	1.93408	-0.09943
C	-0.10271	3.03613	-0.05279
H	-2.12833	3.72443	0.0711
H	-3.09468	1.45695	0.12705
H	1.81297	2.04725	-0.15525
H	0.32231	4.02966	-0.08167
Br	1.18041	-0.97072	-0.11678
O	-0.63703	-1.78431	-0.38264
C	-1.69058	-0.9845	0.03468

C	-2.86618	-1.18298	-0.92188
C	-2.10141	-1.32814	1.47242
H	-2.57499	-0.89886	-1.93205
H	-3.74087	-0.60407	-0.62299
H	-2.91611	-0.69238	1.82282
H	-3.13356	-2.23898	-0.92145
H	-2.42141	-2.36932	1.50635
H	-1.25084	-1.20578	2.14422
C	2.87922	-0.03555	0.10832
H	3.0106	0.65322	0.91656
C	4.3036	-0.48069	0.241
H	4.39568	-1.13578	1.082
H	4.60003	-0.99749	-0.64779

Sum of electronic and zero-point Energies=-3076.020584
Sum of electronic and thermal Energies= -3076.007360
Sum of electronic and thermal Enthalpies= -3076.006416
Sum of electronic and thermal Free Energies= -3076.060558

1-BrIII-Cl

C	2.52236	2.11339	-0.00003
C	2.66085	0.73134	-0.00002
C	1.52519	-0.07221	-0.00001
C	0.30752	0.56194	0.00001
C	0.09823	1.91869	0.00008
C	1.25733	2.69731	0.00004
H	3.40253	2.74383	-0.00006
H	3.62846	0.24255	-0.00002
H	-0.89074	2.35437	0.00019
H	1.15606	3.77559	0.00007
Br	-1.17132	-0.78467	0.00008
O	0.38663	-2.12239	-0.00002
Cl	-2.94445	0.85661	-0.00013
C	1.58381	-1.57491	-0.00003
C	2.2915	-2.1132	1.25736
C	2.29144	-2.11318	-1.25745
H	3.34533	-1.94952	-1.17123
H	1.92071	-1.60247	-2.12153
H	2.09996	-3.16154	-1.35326
H	2.05234	-3.1482	1.3857
H	3.34992	-2.00288	1.1457
H	1.96393	-1.56251	2.1143

Sum of electronic and zero-point Energies=-3458.312891
Sum of electronic and thermal Energies= -3458.301805
Sum of electronic and thermal Enthalpies= -3458.300861
Sum of electronic and thermal Free Energies= -3458.349932

1-BrIII-CN

C	2.38355	2.16507	-0.00011
C	2.54579	0.78572	-0.00018
C	1.42977	-0.04976	-0.00014
C	0.21531	0.57735	0.00019
C	-0.03278	1.92761	0.00009
C	1.11008	2.72995	0.00012
H	3.25324	2.81011	-0.00022
H	3.52047	0.31084	-0.00021
H	-1.02602	2.35531	0.00032
H	0.98659	3.8058	0.00018
Br	-1.29133	-0.79808	-0.00016
O	0.38892	-2.14584	0.00036
C	-2.68934	0.59238	0.00006
N	-3.53721	1.37008	0.00019
C	1.54327	-1.57372	0.00002
C	2.25451	-2.10752	1.25731
C	2.25411	-2.10754	-1.2575
H	1.80196	-1.68452	2.12977
H	3.28884	-1.83553	1.22454
H	3.28438	-1.8196	-1.23413
H	1.78933	-1.69904	-2.13043
H	2.18253	-3.17486	-1.28166
H	2.16689	-3.17338	1.29121

Sum of electronic and zero-point Energies=-3090.910144
Sum of electronic and thermal Energies= -3090.897422
Sum of electronic and thermal Enthalpies= -3090.896478
Sum of electronic and thermal Free Energies= -3090.949376

1-BrIII-F

C	-2.93446	-1.10859	0.00015
C	-2.52243	0.21846	0.00015
C	-1.16116	0.50593	0.
C	-0.29395	-0.55644	-0.00016
C	-0.62935	-1.88835	-0.00026
C	-2.00137	-2.14559	-0.00009
H	-3.99148	-1.34315	0.0003
H	-3.22289	1.04544	0.00026
H	0.12474	-2.66268	-0.00066
H	-2.33707	-3.17535	-0.00015
Br	1.57797	0.06966	0.00005
O	0.73007	1.89152	-0.00025
F	2.14787	-1.78352	0.00019
C	-0.59322	1.89656	-0.00007
C	-1.01634	2.67844	1.2574
C	-1.01649	2.67857	-1.2574
H	-0.94281	2.04221	2.11455
H	-2.02689	3.01224	1.14662
H	-2.04465	2.9622	-1.17173
H	-0.37331	3.52413	1.38474
H	-0.41123	3.5558	-1.35241
H	-0.88769	2.06105	-2.12168

Sum of electronic and zero-point Energies=-3097.947196
Sum of electronic and thermal Energies= -3097.936636
Sum of electronic and thermal Enthalpies= -3097.935692
Sum of electronic and thermal Free Energies= -3097.983191

1-BrIII-N3

C	-2.93446	-1.10859	0.00015
C	-2.52243	0.21846	0.00015
C	-1.16116	0.50593	0.
C	-0.29395	-0.55644	-0.00016
C	-0.62935	-1.88835	-0.00026
C	-2.00137	-2.14559	-0.00009
H	-3.99148	-1.34315	0.0003
H	-3.22289	1.04544	0.00026
H	0.12474	-2.66268	-0.00066
H	-2.33707	-3.17535	-0.00015
Br	1.57797	0.06966	0.00005
O	0.73007	1.89152	-0.00025
C	-0.59322	1.89656	-0.00007
C	-1.01634	2.67844	1.2574
C	-1.01649	2.67857	-1.2574
H	-0.94281	2.04221	2.11455
H	-2.02689	3.01224	1.14662
H	-2.04465	2.9622	-1.17173
H	-0.37331	3.52413	1.38474
H	-0.41123	3.5558	-1.35241
H	-0.88769	2.06105	-2.12168
N	2.83867	-4.1374	0.02604
N	2.56344	-3.08239	0.08667
N	2.11882	-1.68906	0.00018

Sum of electronic and zero-point Energies=-3162.271158
Sum of electronic and thermal Energies= -3162.257755
Sum of electronic and thermal Enthalpies= -3162.256811
Sum of electronic and thermal Free Energies= -3162.312011

1-BrIII-NH2

C	-2.04035	2.84698	0.05489
C	-2.5659	1.56311	0.11017
C	-1.72726	0.46123	0.01266
C	-0.37785	0.7091	-0.1241
C	0.19157	1.95846	-0.17863
C	-0.67465	3.04314	-0.09188
H	-2.70165	3.69963	0.12404
H	-3.63161	1.41448	0.22532
H	1.25822	2.08618	-0.28069
H	-0.26946	4.04403	-0.14095
Br	0.61073	-0.9639	-0.22502
O	-1.11851	-1.7315	-0.54841
C	-2.14837	-0.99464	0.08134
C	-3.42086	-1.26546	-0.71001
C	-2.3016	-1.41942	1.54222
N	2.30521	0.27775	0.07282
H	-2.55284	-2.45868	1.58355
H	-3.07183	-0.85294	2.02259
H	-1.388	-1.26529	2.07747
H	-3.61394	-2.31789	-0.70893
H	-4.25856	-0.76783	-0.26783
H	-3.30578	-0.93469	-1.72107
H	2.63855	0.74915	0.88932
H	2.63855	0.74915	-0.74368

Sum of electronic and zero-point Energies=-3054.004873
Sum of electronic and thermal Energies= -3053.992859
Sum of electronic and thermal Enthalpies= -3053.991915
Sum of electronic and thermal Free Energies= -3054.042866

1-BrIII-NHAc

C	-2.48749	2.65494	0.07595
C	-2.82287	1.309	0.13098
C	-1.83276	0.34168	0.02766
C	-0.53154	0.77435	-0.1168
C	-0.15068	2.09807	-0.17026
C	-1.16408	3.04518	-0.0759
H	-3.26389	3.404	0.1492
H	-3.85566	1.00869	0.25032
H	0.88405	2.37614	-0.27841
H	-0.90926	4.09471	-0.12394
Br	0.66853	-0.73177	-0.22621
O	-0.90393	-1.73597	-0.5306
C	3.33499	-0.0176	0.10895
O	3.45194	-1.21954	0.00462
C	4.50791	0.91287	0.30858
H	4.56178	1.61088	-0.52588
H	4.36296	1.49441	1.21765
H	5.42474	0.33562	0.37207
C	-2.0357	-1.16154	0.09642
C	-3.25395	-1.61798	-0.69284
C	-2.12385	-1.60413	1.55773
H	-3.18826	-1.26973	-1.72199

H	-4.17329	-1.24228	-0.24339
H	-3.28946	-2.70645	-0.69055
H	-2.22146	-2.68838	1.60003
H	-2.98465	-1.14803	2.04745
H	-1.22512	-1.31276	2.10233
N	2.18186	0.61126	0.05843
H	2.67875	1.18833	-0.58971

Sum of electronic and zero-point Energies=-3206.632992
Sum of electronic and thermal Energies= -3206.617259
Sum of electronic and thermal Enthalpies= -3206.616314
Sum of electronic and thermal Free Energies= -3206.677192

1-BrIII-OCF3

C	2.67388	2.50582	-0.08055
C	3.07538	1.1861	-0.2436
C	2.13864	0.16777	-0.09614
C	0.85091	0.5348	0.20224
C	0.38735	1.81389	0.38416
C	1.35008	2.81324	0.23105
H	3.39497	3.30541	-0.19349
H	4.09694	0.91345	-0.48175
H	-0.64249	2.02395	0.63
H	1.04878	3.8455	0.35987
Br	-0.29449	-1.08279	0.36657
O	1.40068	-2.06043	-0.03196
F	-3.25665	-0.89735	-0.63674
F	-2.19308	0.86999	-1.26105
F	-3.78459	1.02948	0.19587
C	-2.75499	0.26941	-0.18023
O	-1.90316	0.11538	0.81056
C	2.46298	-1.28967	-0.23514
C	3.51068	-1.74135	0.79923
C	2.97123	-1.60853	-1.65345
H	2.33942	-1.13273	-2.37409
H	3.97242	-1.2473	-1.76315
H	2.95499	-2.66711	-1.80855
H	4.48184	-1.41753	0.48797
H	3.49838	-2.80847	0.87672
H	3.27978	-1.31189	1.75167

Sum of electronic and zero-point Energies=-3411.003072
Sum of electronic and thermal Energies= -3410.987935
Sum of electronic and thermal Enthalpies= -3410.986991
Sum of electronic and thermal Free Energies= -3411.046608

1-BrIII-OCH3

C	2.67388	2.50582	-0.08055
C	3.07538	1.1861	-0.2436
C	2.13864	0.16777	-0.09614
C	0.85091	0.5348	0.20224
C	0.38735	1.81389	0.38416
C	1.35008	2.81324	0.23105
H	3.39497	3.30541	-0.19349
H	4.09694	0.91345	-0.48175
H	-0.64249	2.02395	0.63
H	1.04878	3.8455	0.35987
Br	-0.29449	-1.08279	0.36657
O	1.40068	-2.06043	-0.03196
C	-2.75499	0.26941	-0.18023
O	-1.90316	0.11538	0.81056
C	2.46298	-1.28967	-0.23514
C	3.51068	-1.74135	0.79923
C	2.97123	-1.60853	-1.65345
H	2.33942	-1.13273	-2.37409
H	3.97242	-1.2473	-1.76315
H	2.95499	-2.66711	-1.80855
H	4.48184	-1.41753	0.48797
H	3.49838	-2.80847	0.87672
H	3.27978	-1.31189	1.75167
H	-2.3123	0.74256	-1.03173
H	-3.5809	0.87912	0.12146
H	-3.15272	-0.65563	-0.54216

Sum of electronic and zero-point Energies=-3113.165407
Sum of electronic and thermal Energies= -3113.152109
Sum of electronic and thermal Enthalpies= -3113.151165
Sum of electronic and thermal Free Energies= -3113.204942

1-BrIII-OCOCF3

C	3.22289	2.62188	0.00859
C	3.68898	1.31361	0.00651
C	2.77394	0.26568	0.00105
C	1.43871	0.58809	-0.00237
C	0.91134	1.85792	-0.00002
C	1.85448	2.88734	0.0057
H	3.92673	3.44448	0.01292
H	4.74543	1.07154	0.00947
H	-0.15229	2.04153	-0.00164
H	1.50103	3.91113	0.00798
Br	0.34895	-1.0718	-0.00868
O	2.11919	-1.99141	-0.00185
C	-2.41859	-0.53165	0.0117
O	-2.5234	-1.72768	0.04512
O	-1.32746	0.16971	-0.02174
C	-3.65945	0.39701	0.00003
F	-4.78188	-0.30037	0.05516
F	-3.67375	1.13597	-1.11316
F	-3.62623	1.23022	1.0443
C	3.17219	-1.17944	0.00108
C	3.98348	-1.53747	1.26013
C	3.99077	-1.53249	-1.25466
H	3.51586	-1.11656	-2.11858
H	4.97784	-1.13005	-1.16176
H	4.04736	-2.59617	-1.35612
H	4.98789	-1.18568	1.14921
H	3.98986	-2.5995	1.39027
H	3.53637	-1.07597	2.11572

Sum of electronic and zero-point Energies=-3524.324308
Sum of electronic and thermal Energies= -3524.306999
Sum of electronic and thermal Enthalpies= -3524.306055
Sum of electronic and thermal Free Energies= -3524.372161

1-BrIII-OCOCH3

C	2.94595	2.24262	0.
C	3.14905	0.86891	-0.00023
C	2.05101	0.01452	-0.00014
C	0.80079	0.58421	0.00015
C	0.53351	1.93493	0.00041
C	1.65414	2.76631	0.00033
H	3.79558	2.9138	-0.00007
H	4.13833	0.42604	-0.00045
H	-0.47532	2.31781	0.00073
H	1.50439	3.83907	0.00055
Br	-0.60316	-0.8154	0.00013
O	1.00226	-2.08015	0.
C	-3.20495	0.1778	0.00021
O	-3.42588	-1.00956	0.00076
O	-1.9799	0.68529	0.00005
C	-4.26655	1.25034	-0.00099
H	-4.14463	1.88403	0.87785
H	-4.15011	1.8754	-0.88681
H	-5.24737	0.78154	0.00398
C	2.175	-1.48618	-0.00024
C	2.90387	-1.99583	1.25698
C	2.90344	-1.99553	-1.25783
H	2.55306	-1.46032	2.11434
H	3.95682	-1.84118	1.14612
H	2.70817	-3.04008	1.38401
H	2.75561	-3.05101	-1.35272
H	3.94968	-1.78816	-1.17256
H	2.51115	-1.50132	-2.12198

Sum of electronic and zero-point Energies=-3226.530989
Sum of electronic and thermal Energies= -3226.515572
Sum of electronic and thermal Enthalpies= -3226.514628
Sum of electronic and thermal Free Energies= -3226.574551

1-BrIII-OCOPh

C	-2.48749	2.65494	0.07595
C	-2.82287	1.309	0.13098
C	-1.83276	0.34168	0.02766
C	-0.53154	0.77435	-0.1168
C	-0.15068	2.09807	-0.17026
C	-1.16408	3.04518	-0.0759
H	-3.26389	3.404	0.1492
H	-3.85566	1.00869	0.25032
H	0.88405	2.37614	-0.27841
H	-0.90926	4.09471	-0.12394
Br	0.66853	-0.73177	-0.22621
O	-0.90393	-1.73597	-0.5306
C	3.33499	-0.0176	0.10895
O	3.45212	-1.21935	0.00267
O	2.18163	0.61103	0.06075
C	-2.0357	-1.16154	0.09642
C	-3.25395	-1.61798	-0.69284
C	-2.12385	-1.60413	1.55773
H	-3.18826	-1.26973	-1.72199
H	-4.17329	-1.24228	-0.24339
H	-3.28946	-2.70645	-0.69055
H	-2.22146	-2.68838	1.60003
H	-2.98465	-1.14803	2.04745
H	-1.22512	-1.31276	2.10233
C	4.53088	0.93109	0.31249
C	4.31964	2.3056	0.42461
C	5.82519	0.41625	0.38486
C	5.40258	3.16506	0.60838
H	3.29918	2.71126	0.36674
C	6.90848	1.27574	0.56968
H	5.99184	-0.66707	0.29669
C	6.69741	2.64998	0.68131
H	5.23622	4.24854	0.69614
H	7.92884	0.86948	0.62712
H	7.55112	3.32782	0.82627

Sum of electronic and zero-point Energies=-3418.209464
Sum of electronic and thermal Energies= -3418.191247
Sum of electronic and thermal Enthalpies= -3418.190303
Sum of electronic and thermal Free Energies= -3418.257941

1-BrIII-OH

C	2.85263	-1.32247	0.00438
C	2.53897	0.03132	0.02066
C	1.20519	0.42867	0.01343
C	0.26118	-0.56546	0.00447
C	0.49803	-1.91871	-0.02637
C	1.8446	-2.28523	-0.02237
H	3.88939	-1.63508	0.00637
H	3.29795	0.80511	0.03341
H	-0.31078	-2.63443	-0.06185
H	2.09873	-3.33788	-0.04592
Br	-1.58476	0.16966	-0.02049
O	-0.53232	1.99106	-0.02484
O	-2.35941	-1.6203	-0.02187
H	-2.69235	-1.77691	0.86934
C	0.76361	1.8772	0.00979
C	1.26116	2.64337	-1.22998
C	1.22455	2.60844	1.28432
H	0.69564	3.54465	-1.343
H	1.1371	2.033	-2.10002
H	2.29644	2.88479	-1.10834
H	2.27402	2.80736	1.2216
H	1.02831	1.99455	2.13845
H	0.69158	3.53147	1.37846

Sum of electronic and zero-point Energies=-3073.900149
Sum of electronic and thermal Energies= -3073.888367
Sum of electronic and thermal Enthalpies= -3073.887423
Sum of electronic and thermal Free Energies= -3073.937577

1-BrIII-OTf

C	-2.93446	-1.10859	0.00015
C	-2.52243	0.21846	0.00015
C	-1.16116	0.50593	0.
C	-0.29395	-0.55644	-0.00016
C	-0.62935	-1.88835	-0.00026
C	-2.00137	-2.14559	-0.00009
H	-3.99148	-1.34315	0.0003
H	-3.22289	1.04544	0.00026
H	0.12474	-2.66268	-0.00066
H	-2.33707	-3.17535	-0.00015
Br	1.57797	0.06966	0.00005
O	0.73007	1.89152	-0.00025
C	-0.59322	1.89656	-0.00007
C	-1.01634	2.67844	1.2574
C	-1.01649	2.67857	-1.2574
H	-0.94281	2.04221	2.11455
H	-2.02689	3.01224	1.14662
H	-2.04465	2.9622	-1.17173
H	-0.37331	3.52413	1.38474
H	-0.41123	3.5558	-1.35241
H	-0.88769	2.06105	-2.12168
O	2.10706	-1.65082	0.00018
S	3.59206	-1.98206	0.66643
O	3.32762	-2.88312	1.67648
O	4.05277	-0.70442	0.94415
C	4.38426	-2.66762	-0.56896
F	4.64947	-1.73381	-1.50712
F	5.54243	-3.19802	-0.12195
F	3.62112	-3.6424	-1.1074

Sum of electronic and zero-point Energies=-3959.610605
Sum of electronic and thermal Energies= -3959.592172
Sum of electronic and thermal Enthalpies= -3959.591228
Sum of electronic and thermal Free Energies= -3959.659134

1-BrIII-OTs

C	-2.93446	-1.10859	0.00015
C	-2.52243	0.21846	0.00015
C	-1.16116	0.50593	0.
C	-0.29395	-0.55644	-0.00016
C	-0.62935	-1.88835	-0.00026
C	-2.00137	-2.14559	-0.00009
H	-3.99148	-1.34315	0.0003
H	-3.22289	1.04544	0.00026
H	0.12474	-2.66268	-0.00066
H	-2.33707	-3.17535	-0.00015
Br	1.57797	0.06966	0.00005
O	0.73007	1.89152	-0.00025
C	-0.59322	1.89656	-0.00007
C	-1.01634	2.67844	1.2574
C	-1.01649	2.67857	-1.2574
H	-0.94281	2.04221	2.11455
H	-2.02689	3.01224	1.14662
H	-2.04465	2.9622	-1.17173
H	-0.37331	3.52413	1.38474
H	-0.41123	3.5558	-1.35241
H	-0.88769	2.06105	-2.12168
O	2.10706	-1.65082	0.00018
S	3.59206	-1.98206	0.66643
O	3.32762	-2.88312	1.67648
O	4.05277	-0.70442	0.94415
C	4.39898	-2.68036	-0.59191
C	4.29095	-4.05732	-0.83881
C	5.21583	-1.91719	-1.44026
C	4.97031	-4.65259	-1.89504
H	3.6592	-4.67373	-0.17344
C	5.89937	-2.50427	-2.49751
H	5.31971	-0.83289	-1.25251
C	5.78031	-3.88013	-2.73787
H	4.87315	-5.73479	-2.07074
H	6.53777	-1.88846	-3.14913
C	6.49103	-4.50754	-3.87454
H	7.41771	-3.93858	-4.1338
H	6.77579	-5.56208	-3.63595
H	5.82389	-4.51998	-4.77423

Sum of electronic and zero-point Energies=-3892.782271
Sum of electronic and thermal Energies= -3892.761813
Sum of electronic and thermal Enthalpies= -3892.760869
Sum of electronic and thermal Free Energies= -3892.833659

1-BrIII-radical

C	-2.93446	-1.10859	0.00015
C	-2.52243	0.21846	0.00015
C	-1.16116	0.50593	0.
C	-0.29395	-0.55644	-0.00016
C	-0.62935	-1.88835	-0.00026
C	-2.00137	-2.14559	-0.00009
H	-3.99148	-1.34315	0.0003
H	-3.22289	1.04544	0.00026
H	0.12474	-2.66268	-0.00066
H	-2.33707	-3.17535	-0.00015
Br	1.57797	0.06966	0.00005
O	0.73007	1.89152	-0.00025
C	-0.59322	1.89656	-0.00007
C	-1.01634	2.67844	1.2574
C	-1.01649	2.67857	-1.2574
H	-0.94281	2.04221	2.11455
H	-2.02689	3.01224	1.14662
H	-2.04465	2.9622	-1.17173
H	-0.37331	3.52413	1.38474
H	-0.41123	3.5558	-1.35241
H	-0.88769	2.06105	-2.12168

Sum of electronic and zero-point Energies=-2998.135254
Sum of electronic and thermal Energies= -2998.124755
Sum of electronic and thermal Enthalpies= -2998.123811
Sum of electronic and thermal Free Energies= -2998.172316

1-BrIII-SCF3

C	2.78872	2.53347	-0.0787
C	3.18535	1.22372	-0.31382
C	2.27986	0.18067	-0.13941
C	1.0132	0.52339	0.25857
C	0.55417	1.79211	0.51366
C	1.48729	2.81374	0.3311
H	3.49368	3.34408	-0.21405
H	4.18916	0.96587	-0.63234
H	-0.45947	1.99472	0.82838
H	1.17977	3.83591	0.514
O	1.66601	-2.0776	-0.15099
F	-3.93551	0.92128	-0.45801
F	-2.96359	-0.85487	-1.19914
F	-1.93008	1.02411	-1.25123
C	-2.77223	0.27822	-0.52401
S	-2.16147	-0.00054	1.15757
Br	-0.14178	-1.121	0.46359
C	2.65512	-1.26809	-0.37758
C	3.78298	-1.73615	0.56074
C	3.06597	-1.52096	-1.84007
H	3.60519	-1.36102	1.54693
H	2.36564	-1.04553	-2.49459
H	3.07479	-2.57372	-2.03113
H	4.04296	-1.11931	-2.01062
H	4.72122	-1.36702	0.20249
H	3.80616	-2.80563	0.58475

Sum of electronic and zero-point Energies=-3733.956326
Sum of electronic and thermal Energies= -3733.940393
Sum of electronic and thermal Enthalpies= -3733.939449
Sum of electronic and thermal Free Energies= -3734.001088

1-ClIII-Br

C	-2.93446	-1.10859	0.00015
C	-2.52243	0.21846	0.00015
C	-1.16116	0.50593	0.
C	-0.29395	-0.55644	-0.00016
C	-0.62935	-1.88835	-0.00026
C	-2.00137	-2.14559	-0.00009
H	-3.99148	-1.34315	0.0003
H	-3.22289	1.04544	0.00026
H	0.12474	-2.66268	-0.00066
H	-2.33707	-3.17535	-0.00015
O	0.73007	1.89152	-0.00025
C	-0.59322	1.89656	-0.00007
C	-1.01634	2.67844	1.2574
C	-1.01649	2.67857	-1.2574
H	-0.94281	2.04221	2.11455
H	-2.02689	3.01224	1.14662
H	-2.04465	2.9622	-1.17173
H	-0.37331	3.52413	1.38474

H	-0.41123	3.5558	-1.35241
H	-0.88769	2.06105	-2.12168
Br	2.14787	-1.78352	0.00019
Cl	1.57797	0.06966	0.00005

Sum of electronic and zero-point Energies=-3458.291169
Sum of electronic and thermal Energies= -3458.279409
Sum of electronic and thermal Enthalpies= -3458.278465
Sum of electronic and thermal Free Energies= -3458.329667

1-ClIII-CCH

C	-2.04035	2.84698	0.05489
C	-2.5659	1.56311	0.11017
C	-1.72726	0.46123	0.01266
C	-0.37785	0.7091	-0.1241
C	0.19157	1.95846	-0.17863
C	-0.67465	3.04314	-0.09188
H	-2.70165	3.69963	0.12404
H	-3.63161	1.41448	0.22532
H	1.25822	2.08618	-0.28069
H	-0.26946	4.04403	-0.14095
O	-1.11851	-1.7315	-0.54841
C	-2.14837	-0.99464	0.08134
C	-3.42086	-1.26546	-0.71001
C	-2.3016	-1.41942	1.54222
H	-3.3038	-0.92901	-1.73841
H	-4.27435	-0.75844	-0.25949
H	-3.08641	-0.84221	2.03169
H	-3.61736	-2.33653	-0.70891
H	-2.55741	-2.47759	1.5843
H	-1.37045	-1.26233	2.08775
C	3.50893	0.60328	-0.42291
H	4.44663	1.11586	-0.47663
C	2.45626	0.02786	-0.36262
Cl	0.68903	-1.09642	-0.23301

Sum of electronic and zero-point Energies= -960.772643
Sum of electronic and thermal Energies= -960.760009
Sum of electronic and thermal Enthalpies=-960.759065
Sum of electronic and thermal Free Energies= -960.811680

1-ClIII-CF3

C	-2.69011	2.28973	0.00005
C	-2.95457	0.92757	0.0001
C	-1.91235	-0.00045	0.00001
C	-0.64991	0.52412	-0.00006
C	-0.30538	1.85846	-0.00013
C	-1.37667	2.75141	-0.00009
H	-3.50769	2.99983	0.00013
H	-3.96181	0.52596	0.00018
H	0.70804	2.22727	-0.00024
H	-1.16527	3.81354	-0.00016
O	-1.13277	-2.20926	-0.00037
C	2.40402	0.26982	0.00001
F	3.4394	-0.54682	0.00069
F	2.44547	1.03228	1.07774
F	2.44614	1.03133	-1.07837
C	-2.19866	-1.51874	-0.00005
C	-2.95724	-1.98293	-1.2573
C	-2.95672	-1.98296	1.25751
H	-2.97973	-3.05231	-1.28578
H	-3.95818	-1.60575	-1.22951
H	-2.46089	-1.61324	-2.13015
H	-2.99293	-3.05215	1.27777
H	-3.9528	-1.59266	1.23792
H	-2.45112	-1.62661	2.1306
Cl	0.77117	-0.94889	0.00007

Sum of electronic and zero-point Energies=-1221.726817
Sum of electronic and thermal Energies= -1221.712829
Sum of electronic and thermal Enthalpies= -1221.711884
Sum of electronic and thermal Free Energies= -1221.767666

1-ClIII-CH3

C	-2.69011	2.28973	0.00005
C	-2.95457	0.92757	0.0001
C	-1.91235	-0.00045	0.00001
C	-0.64991	0.52412	-0.00006
C	-0.30538	1.85846	-0.00013
C	-1.37667	2.75141	-0.00009
H	-3.50769	2.99983	0.00013
H	-3.96181	0.52596	0.00018
H	0.70804	2.22727	-0.00024
H	-1.16527	3.81354	-0.00016
O	-1.13277	-2.20926	-0.00037
C	2.40402	0.26982	0.00001
C	-2.19866	-1.51874	-0.00005
C	-2.95724	-1.98293	-1.2573
C	-2.95672	-1.98296	1.25751
H	-2.97973	-3.05231	-1.28578
H	-3.95818	-1.60575	-1.22951
H	-2.46089	-1.61324	-2.13015
H	-2.99293	-3.05215	1.27777
H	-3.9528	-1.59266	1.23792
H	-2.45112	-1.62661	2.1306
H	2.4376	0.8875	0.87308
H	3.24415	-0.39281	0.00056
H	2.43814	0.88672	-0.87359
Cl	0.77117	-0.94889	0.00007

Sum of electronic and zero-point Energies= -923.933982
Sum of electronic and thermal Energies= -923.922014
Sum of electronic and thermal Enthalpies=-923.921070
Sum of electronic and thermal Free Energies= -923.971972

1-ClIII-CHCH2

C	-1.47784	2.86145	0.03034
C	-2.0225	1.58679	0.05887
C	-1.20342	0.46278	-0.00179
C	0.14941	0.69073	-0.073
C	0.74099	1.93408	-0.09943
C	-0.10271	3.03613	-0.05279
H	-2.12833	3.72443	0.0711
H	-3.09468	1.45695	0.12705
H	1.81297	2.04725	-0.15525
H	0.32231	4.02966	-0.08167
O	-0.63703	-1.78431	-0.38264
C	-1.69058	-0.9845	0.03468
C	-2.86618	-1.18298	-0.92188
C	-2.10141	-1.32814	1.47242
H	-2.57499	-0.89886	-1.93205
H	-3.74087	-0.60407	-0.62299
H	-2.91611	-0.69238	1.82282
H	-3.13356	-2.23898	-0.92145
H	-2.42141	-2.36932	1.50635
H	-1.25084	-1.20578	2.14422
C	2.87922	-0.03555	0.10832
H	3.0106	0.65322	0.91656
C	4.3036	-0.48069	0.241
H	4.39568	-1.13578	1.082
H	4.60003	-0.99749	-0.64779
Cl	1.18041	-0.97072	-0.11678

Sum of electronic and zero-point Energies= -962.006310
Sum of electronic and thermal Energies= -961.993429
Sum of electronic and thermal Enthalpies=-961.992485
Sum of electronic and thermal Free Energies= -962.045408

1-ClIII-Cl

C	2.52236	2.11339	-0.00003
C	2.66085	0.73134	-0.00002
C	1.52519	-0.07221	-0.00001
C	0.30752	0.56194	0.00001
C	0.09823	1.91869	0.00008
C	1.25733	2.69731	0.00004
H	3.40253	2.74383	-0.00006
H	3.62846	0.24255	-0.00002
H	-0.89074	2.35437	0.00019
H	1.15606	3.77559	0.00007
O	0.38663	-2.12239	-0.00002
Cl	-2.94445	0.85661	-0.00013
C	1.58381	-1.57491	-0.00003
C	2.2915	-2.1132	1.25736
C	2.29144	-2.11318	-1.25745
H	3.34533	-1.94952	-1.17123
H	1.92071	-1.60247	-2.12153
H	2.09996	-3.16154	-1.35326
H	2.05234	-3.1482	1.3857
H	3.34992	-2.00288	1.1457
H	1.96393	-1.56251	2.1143
Cl	-0.99381	-0.62303	0.00007

Sum of electronic and zero-point Energies=-1344.288970
Sum of electronic and thermal Energies= -1344.277444
Sum of electronic and thermal Enthalpies= -1344.276500
Sum of electronic and thermal Free Energies= -1344.326389

1-ClIII-CN

C	2.38355	2.16507	-0.00011
C	2.54579	0.78572	-0.00018
C	1.42977	-0.04976	-0.00014
C	0.21531	0.57735	0.00019
C	-0.03278	1.92761	0.00009
C	1.11008	2.72995	0.00012
H	3.25324	2.81011	-0.00022
H	3.52047	0.31084	-0.00021
H	-1.02602	2.35531	0.00032
H	0.98659	3.8058	0.00018
O	0.38892	-2.14584	0.00036
C	-2.68934	0.59238	0.00006
N	-3.53721	1.37008	0.00019
C	1.54327	-1.57372	0.00002
C	2.25451	-2.10752	1.25731
C	2.25411	-2.10754	-1.2575
H	1.80196	-1.68452	2.12977
H	3.28884	-1.83553	1.22454
H	3.28438	-1.8196	-1.23413
H	1.78933	-1.69904	-2.13043
H	2.18253	-3.17486	-1.28166
H	2.16689	-3.17338	1.29121
Cl	-1.44146	-0.64875	-0.00014

Sum of electronic and zero-point Energies= -976.885440

Sum of electronic and thermal Energies= -976.873014
Sum of electronic and thermal Enthalpies=-976.872070
Sum of electronic and thermal Free Energies= -976.923886

1-ClIII-F

C	-2.93446	-1.10859	0.00015
C	-2.52243	0.21846	0.00015
C	-1.16116	0.50593	0.
C	-0.29395	-0.55644	-0.00016
C	-0.62935	-1.88835	-0.00026
C	-2.00137	-2.14559	-0.00009
H	-3.99148	-1.34315	0.0003
H	-3.22289	1.04544	0.00026
H	0.12474	-2.66268	-0.00066
H	-2.33707	-3.17535	-0.00015
O	0.73007	1.89152	-0.00025
F	2.14787	-1.78352	0.00019
C	-0.59322	1.89656	-0.00007
C	-1.01634	2.67844	1.2574
C	-1.01649	2.67857	-1.2574
H	-0.94281	2.04221	2.11455
H	-2.02689	3.01224	1.14662
H	-2.04465	2.9622	-1.17173
H	-0.37331	3.52413	1.38474
H	-0.41123	3.5558	-1.35241
H	-0.88769	2.06105	-2.12168
Cl	1.37517	0.00183	0.00002

Sum of electronic and zero-point Energies= -983.921259
Sum of electronic and thermal Energies= -983.911033
Sum of electronic and thermal Enthalpies=-983.910089
Sum of electronic and thermal Free Energies= -983.956423

1-ClIII-N3

C	-2.93446	-1.10859	0.00015
C	-2.52243	0.21846	0.00015
C	-1.16116	0.50593	0.
C	-0.29395	-0.55644	-0.00016
C	-0.62935	-1.88835	-0.00026
C	-2.00137	-2.14559	-0.00009
H	-3.99148	-1.34315	0.0003
H	-3.22289	1.04544	0.00026
H	0.12474	-2.66268	-0.00066
H	-2.33707	-3.17535	-0.00015
O	0.73007	1.89152	-0.00025
C	-0.59322	1.89656	-0.00007
C	-1.01634	2.67844	1.2574
C	-1.01649	2.67857	-1.2574
H	-0.94281	2.04221	2.11455
H	-2.02689	3.01224	1.14662
H	-2.04465	2.9622	-1.17173
H	-0.37331	3.52413	1.38474
H	-0.41123	3.5558	-1.35241
H	-0.88769	2.06105	-2.12168
N	2.83867	-4.1374	0.02604
N	2.56344	-3.08239	0.08667
N	2.11882	-1.68906	0.00018
Cl	1.57797	0.06966	0.00005

Sum of electronic and zero-point Energies=-1048.247276
Sum of electronic and thermal Energies= -1048.234187
Sum of electronic and thermal Enthalpies= -1048.233242
Sum of electronic and thermal Free Energies= -1048.287377

1-ClIII-NH2

C	-2.04035	2.84698	0.05489
C	-2.5659	1.56311	0.11017
C	-1.72726	0.46123	0.01266
C	-0.37785	0.7091	-0.1241
C	0.19157	1.95846	-0.17863
C	-0.67465	3.04314	-0.09188
H	-2.70165	3.69963	0.12404
H	-3.63161	1.41448	0.22532
H	1.25822	2.08618	-0.28069
H	-0.26946	4.04403	-0.14095
O	-1.11851	-1.7315	-0.54841
C	-2.14837	-0.99464	0.08134
C	-3.42086	-1.26546	-0.71001
C	-2.3016	-1.41942	1.54222
N	2.30521	0.27775	0.07282
H	-2.55284	-2.45868	1.58355
H	-3.07183	-0.85294	2.02259
H	-1.388	-1.26529	2.07747
H	-3.61394	-2.31789	-0.70893
H	-4.25856	-0.76783	-0.26783
H	-3.30578	-0.93469	-1.72107
H	2.63855	0.74915	0.88932
H	2.63855	0.74915	-0.74368
Cl	0.68903	-1.09642	-0.23301

Sum of electronic and zero-point Energies= -939.984178
Sum of electronic and thermal Energies= -939.972253
Sum of electronic and thermal Enthalpies=-939.971309
Sum of electronic and thermal Free Energies= -940.021761

1-ClIII-NHAc

C	-2.48749	2.65494	0.07595
C	-2.82287	1.309	0.13098
C	-1.83276	0.34168	0.02766
C	-0.53154	0.77435	-0.1168
C	-0.15068	2.09807	-0.17026
C	-1.16408	3.04518	-0.0759
H	-3.26389	3.404	0.1492
H	-3.85566	1.00869	0.25032
H	0.88405	2.37614	-0.27841
H	-0.90926	4.09471	-0.12394
O	-0.90393	-1.73597	-0.5306
C	3.33499	-0.0176	0.10895
O	3.45194	-1.21954	0.00462
C	4.50791	0.91287	0.30858
H	4.56178	1.61088	-0.52588
H	4.36296	1.49441	1.21765
H	5.42474	0.33562	0.37207
C	-2.0357	-1.16154	0.09642
C	-3.25395	-1.61798	-0.69284
C	-2.12385	-1.60413	1.55773
H	-3.18826	-1.26973	-1.72199
H	-4.17329	-1.24228	-0.24339
H	-3.28946	-2.70645	-0.69055
H	-2.22146	-2.68838	1.60003
H	-2.98465	-1.14803	2.04745
H	-1.22512	-1.31276	2.10233
N	2.18186	0.61126	0.05843
H	2.67875	1.18833	-0.58971
Cl	0.66853	-0.73177	-0.22621

Sum of electronic and zero-point Energies=-1092.608096
Sum of electronic and thermal Energies= -1092.592799
Sum of electronic and thermal Enthalpies= -1092.591855
Sum of electronic and thermal Free Energies= -1092.651025

1-ClIII-OCF3

C	2.67388	2.50582	-0.08055
C	3.07538	1.1861	-0.2436
C	2.13864	0.16777	-0.09614
C	0.85091	0.5348	0.20224
C	0.38735	1.81389	0.38416
C	1.35008	2.81324	0.23105
H	3.39497	3.30541	-0.19349
H	4.09694	0.91345	-0.48175
H	-0.64249	2.02395	0.63
H	1.04878	3.8455	0.35987
O	1.40068	-2.06043	-0.03196
F	-3.25665	-0.89735	-0.63674
F	-2.19308	0.86999	-1.26105
F	-3.78459	1.02948	0.19587
C	-2.75499	0.26941	-0.18023
O	-1.90316	0.11538	0.81056
C	2.46298	-1.28967	-0.23514
C	3.51068	-1.74135	0.79923
C	2.97123	-1.60853	-1.65345
H	2.33942	-1.13273	-2.37409
H	3.97242	-1.2473	-1.76315
H	2.95499	-2.66711	-1.80855
H	4.48184	-1.41753	0.48797
H	3.49838	-2.80847	0.87672
H	3.27978	-1.31189	1.75167
Cl	-0.16269	-0.89665	0.34766

Sum of electronic and zero-point Energies=-1296.979435
Sum of electronic and thermal Energies= -1296.964611
Sum of electronic and thermal Enthalpies= -1296.963667
Sum of electronic and thermal Free Energies= -1297.022314

1-ClIII-OCH3

C	2.67388	2.50582	-0.08055
C	3.07538	1.1861	-0.2436
C	2.13864	0.16777	-0.09614
C	0.85091	0.5348	0.20224
C	0.38735	1.81389	0.38416
C	1.35008	2.81324	0.23105
H	3.39497	3.30541	-0.19349
H	4.09694	0.91345	-0.48175
H	-0.64249	2.02395	0.63
H	1.04878	3.8455	0.35987
O	1.40068	-2.06043	-0.03196
C	-2.75499	0.26941	-0.18023
O	-1.90316	0.11538	0.81056
C	2.46298	-1.28967	-0.23514
C	3.51068	-1.74135	0.79923
C	2.97123	-1.60853	-1.65345
H	2.33942	-1.13273	-2.37409
H	3.97242	-1.2473	-1.76315
H	2.95499	-2.66711	-1.80855
H	4.48184	-1.41753	0.48797
H	3.49838	-2.80847	0.87672
H	3.27978	-1.31189	1.75167
H	-2.3123	0.74256	-1.03173
H	-3.5809	0.87912	0.12146
H	-3.15272	-0.65563	-0.54216
Cl	-0.16269	-0.89665	0.34766

Sum of electronic and zero-point Energies= -999.141460
Sum of electronic and thermal Energies= -999.128476
Sum of electronic and thermal Enthalpies=-999.127532
Sum of electronic and thermal Free Energies= -999.180305

1-ClIII-OCOCF3

C	3.22289	2.62188	0.00859
C	3.68898	1.31361	0.00651
C	2.77394	0.26568	0.00105
C	1.43871	0.58809	-0.00237
C	0.91134	1.85792	-0.00002
C	1.85448	2.88734	0.0057
H	3.92673	3.44448	0.01292
H	4.74543	1.07154	0.00947
H	-0.15229	2.04153	-0.00164
H	1.50103	3.91113	0.00798
O	2.11919	-1.99141	-0.00185
C	-2.41859	-0.53165	0.0117
O	-2.5234	-1.72768	0.04512
O	-1.32746	0.16971	-0.02174
C	-3.65945	0.39701	0.00003
F	-4.78188	-0.30037	0.05516
F	-3.67375	1.13597	-1.11316
F	-3.62623	1.23022	1.0443
C	3.17219	-1.17944	0.00108
C	3.98348	-1.53747	1.26013
C	3.99077	-1.53249	-1.25466
H	3.51586	-1.11656	-2.11858
H	4.97784	-1.13005	-1.16176
H	4.04736	-2.59617	-1.35612
H	4.98789	-1.18568	1.14921
H	3.98986	-2.5995	1.39027
H	3.53637	-1.07597	2.11572
Cl	0.47279	-0.88316	-0.00796

Sum of electronic and zero-point Energies=-1410.300522
Sum of electronic and thermal Energies= -1410.283504
Sum of electronic and thermal Enthalpies= -1410.282560
Sum of electronic and thermal Free Energies= -1410.348224

1-ClIII-OCOCH3

C	2.70205	-0.90468	0.03268
C	2.36699	0.4492	0.11713
C	1.05019	0.83398	-0.00405
C	0.08991	-0.14034	-0.19627
C	0.37541	-1.48074	-0.28329
C	1.71022	-1.86092	-0.17075
H	3.15604	1.17344	0.26823
H	-0.40432	-2.2086	-0.42728
H	1.95557	-2.90889	-0.24485
O	-0.75123	2.14805	-0.61643
C	-3.60038	-1.0847	0.08543
O	-4.2044	-0.0377	-0.01172
O	-2.29571	-1.19558	0.00492
C	-4.29149	-2.41116	0.3128
H	-4.08022	-3.07783	-0.52237
H	-3.9009	-2.8778	1.21585
H	-5.36182	-2.25396	0.40242
C	0.49359	2.23856	0.0651
C	1.33712	3.24756	-0.69577
C	0.28467	2.65779	1.51895
H	1.48472	2.91877	-1.72259
H	2.30761	3.37242	-0.21513
H	0.8282	4.21035	-0.70082
H	-0.17663	3.64421	1.54807
H	1.23857	2.69299	2.04547
H	-0.36681	1.95131	2.03351
Cl	-1.52824	0.60049	-0.30451
H	3.74339	-1.1308	0.12949

Sum of electronic and zero-point Energies=-1112.505368
Sum of electronic and thermal Energies= -1112.490290
Sum of electronic and thermal Enthalpies= -1112.489346
Sum of electronic and thermal Free Energies= -1112.548229

1-ClIII-OCOPh

C	-2.48749	2.65494	0.07595
C	-2.82287	1.309	0.13098
C	-1.83276	0.34168	0.02766
C	-0.53154	0.77435	-0.1168
C	-0.15068	2.09807	-0.17026
C	-1.16408	3.04518	-0.0759
H	-3.26389	3.404	0.1492
H	-3.85566	1.00869	0.25032
H	0.88405	2.37614	-0.27841
H	-0.90926	4.09471	-0.12394
O	-0.90393	-1.73597	-0.5306
C	3.33499	-0.0176	0.10895
O	3.45212	-1.21935	0.00267
O	2.18163	0.61103	0.06075
C	-2.0357	-1.16154	0.09642
C	-3.25395	-1.61798	-0.69284
C	-2.12385	-1.60413	1.55773
H	-3.18826	-1.26973	-1.72199
H	-4.17329	-1.24228	-0.24339
H	-3.28946	-2.70645	-0.69055
H	-2.22146	-2.68838	1.60003
H	-2.98465	-1.14803	2.04745
H	-1.22512	-1.31276	2.10233
C	4.53088	0.93109	0.31249
C	4.31964	2.3056	0.42461
C	5.82519	0.41625	0.38486
C	5.40258	3.16506	0.60838
H	3.29918	2.71126	0.36674
C	6.90848	1.27574	0.56968
H	5.99184	-0.66707	0.29669
C	6.69741	2.64998	0.68131
H	5.23622	4.24854	0.69614
H	7.92884	0.86948	0.62712
H	7.55112	3.32782	0.82627
Cl	0.66853	-0.73177	-0.22621

Sum of electronic and zero-point Energies=-1304.184049
Sum of electronic and thermal Energies= -1304.166151
Sum of electronic and thermal Enthalpies= -1304.165207
Sum of electronic and thermal Free Energies= -1304.231965

1-ClIII-OH

C	2.85263	-1.32247	0.00438
C	2.53897	0.03132	0.02066
C	1.20519	0.42867	0.01343
C	0.26118	-0.56546	0.00447
C	0.49803	-1.91871	-0.02637
C	1.8446	-2.28523	-0.02237
H	3.88939	-1.63508	0.00637
H	3.29795	0.80511	0.03341
H	-0.31078	-2.63443	-0.06185
H	2.09873	-3.33788	-0.04592
O	-0.53232	1.99106	-0.02484
O	-2.35941	-1.6203	-0.02187
H	-2.69235	-1.77691	0.86934
C	0.76361	1.8772	0.00979
C	1.26116	2.64337	-1.22998
C	1.22455	2.60844	1.28432
H	0.69564	3.54465	-1.343
H	1.1371	2.033	-2.10002
H	2.29644	2.88479	-1.10834
H	2.27402	2.80736	1.2216
H	1.02831	1.99455	2.13845
H	0.69158	3.53147	1.37846
Cl	-1.3738	0.08565	-0.01763

Sum of electronic and zero-point Energies= -959.874640
Sum of electronic and thermal Energies= -959.863123
Sum of electronic and thermal Enthalpies=-959.862179
Sum of electronic and thermal Free Energies= -959.911316

1-ClIII-OTf

C	-2.93446	-1.10859	0.00015
C	-2.52243	0.21846	0.00015
C	-1.16116	0.50593	0.
C	-0.29395	-0.55644	-0.00016
C	-0.62935	-1.88835	-0.00026
C	-2.00137	-2.14559	-0.00009
H	-3.99148	-1.34315	0.0003
H	-3.22289	1.04544	0.00026
H	0.12474	-2.66268	-0.00066
H	-2.33707	-3.17535	-0.00015
O	0.73007	1.89152	-0.00025
C	-0.59322	1.89656	-0.00007
C	-1.01634	2.67844	1.2574
C	-1.01649	2.67857	-1.2574
H	-0.94281	2.04221	2.11455
H	-2.02689	3.01224	1.14662
H	-2.04465	2.9622	-1.17173
H	-0.37331	3.52413	1.38474
H	-0.41123	3.5558	-1.35241
H	-0.88769	2.06105	-2.12168
O	2.10706	-1.65082	0.00018
S	3.59206	-1.98206	0.66643
O	3.32762	-2.88312	1.67648
O	4.05277	-0.70442	0.94415
C	4.38426	-2.66762	-0.56896
F	4.64947	-1.73381	-1.50712
F	5.54243	-3.19802	-0.12195
F	3.62112	-3.6424	-1.1074
Cl	1.57797	0.06966	0.00005

Sum of electronic and zero-point Energies=-1845.590954
Sum of electronic and thermal Energies= -1845.572778
Sum of electronic and thermal Enthalpies= -1845.571834
Sum of electronic and thermal Free Energies= -1845.639334

1-ClIII-OTs

C	-2.93446	-1.10859	0.00015
C	-2.52243	0.21846	0.00015
C	-1.16116	0.50593	0.
C	-0.29395	-0.55644	-0.00016
C	-0.62935	-1.88835	-0.00026
C	-2.00137	-2.14559	-0.00009
H	-3.99148	-1.34315	0.0003

H	-3.22289	1.04544	0.00026
H	0.12474	-2.66268	-0.00066
H	-2.33707	-3.17535	-0.00015
O	0.73007	1.89152	-0.00025
C	-0.59322	1.89656	-0.00007
C	-1.01634	2.67844	1.2574
C	-1.01649	2.67857	-1.2574
H	-0.94281	2.04221	2.11455
H	-2.02689	3.01224	1.14662
H	-2.04465	2.9622	-1.17173
H	-0.37331	3.52413	1.38474
H	-0.41123	3.5558	-1.35241
H	-0.88769	2.06105	-2.12168
O	2.10706	-1.65082	0.00018
S	3.59206	-1.98206	0.66643
O	3.32762	-2.88312	1.67648
O	4.05277	-0.70442	0.94415
C	4.39898	-2.68036	-0.59191
C	4.29095	-4.05732	-0.83881
C	5.21583	-1.91719	-1.44026
C	4.97031	-4.65259	-1.89504
H	3.6592	-4.67373	-0.17344
C	5.89937	-2.50427	-2.49751
H	5.31971	-0.83289	-1.25251
C	5.78031	-3.88013	-2.73787
H	4.87315	-5.73479	-2.07074
H	6.53777	-1.88846	-3.14913
C	6.49103	-4.50754	-3.87454
H	7.41771	-3.93858	-4.1338
H	6.77579	-5.56208	-3.63595
H	5.82389	-4.51998	-4.77423
Cl	1.57797	0.06966	0.00005

Sum of electronic and zero-point Energies=-1778.760436
Sum of electronic and thermal Energies= -1778.739396
Sum of electronic and thermal Enthalpies= -1778.738452
Sum of electronic and thermal Free Energies= -1778.813401

1-ClIII-radical

C	-2.93446	-1.10859	0.00015
C	-2.52243	0.21846	0.00015
C	-1.16116	0.50593	0.
C	-0.29395	-0.55644	-0.00016
C	-0.62935	-1.88835	-0.00026
C	-2.00137	-2.14559	-0.00009
H	-3.99148	-1.34315	0.0003
H	-3.22289	1.04544	0.00026
H	0.12474	-2.66268	-0.00066
H	-2.33707	-3.17535	-0.00015
O	0.73007	1.89152	-0.00025
C	-0.59322	1.89656	-0.00007
C	-1.01634	2.67844	1.2574
C	-1.01649	2.67857	-1.2574
H	-0.94281	2.04221	2.11455
H	-2.02689	3.01224	1.14662
H	-2.04465	2.9622	-1.17173
H	-0.37331	3.52413	1.38474
H	-0.41123	3.5558	-1.35241
H	-0.88769	2.06105	-2.12168
Cl	1.37517	0.00183	0.00002

Sum of electronic and zero-point Energies= -884.141381
Sum of electronic and thermal Energies= -884.131126
Sum of electronic and thermal Enthalpies=-884.130182
Sum of electronic and thermal Free Energies= -884.177456

1-ClIII-SCF3

C	2.78872	2.53347	-0.0787
C	3.18535	1.22372	-0.31382
C	2.27986	0.18067	-0.13941
C	1.0132	0.52339	0.25857
C	0.55417	1.79211	0.51366
C	1.48729	2.81374	0.3311
H	3.49368	3.34408	-0.21405
H	4.18916	0.96587	-0.63234
H	-0.45947	1.99472	0.82838
H	1.17977	3.83591	0.514
O	1.66601	-2.0776	-0.15099
F	-3.93551	0.92128	-0.45801
F	-2.96359	-0.85487	-1.19914
F	-1.93008	1.02411	-1.25123
C	-2.77223	0.27822	-0.52401
S	-2.16147	-0.00054	1.15757
C	2.65512	-1.26809	-0.37758
C	3.78298	-1.73615	0.56074
C	3.06597	-1.52096	-1.84007
H	3.60519	-1.36102	1.54693
H	2.36564	-1.04553	-2.49459
H	3.07479	-2.57372	-2.03113
H	4.04296	-1.11931	-2.01062
H	4.72122	-1.36702	0.20249
H	3.80616	-2.80563	0.58475
Cl	0.00684	-0.90941	0.43721

Sum of electronic and zero-point Energies=-1619.931780
Sum of electronic and thermal Energies= -1619.916124
Sum of electronic and thermal Enthalpies= -1619.915180
Sum of electronic and thermal Free Energies= -1619.975984

1-IIII-Br

C	-2.93446	-1.10859	0.00015
C	-2.52243	0.21846	0.00015
C	-1.16116	0.50593	0.
C	-0.29395	-0.55644	-0.00016
C	-0.62935	-1.88835	-0.00026
C	-2.00137	-2.14559	-0.00009
H	-3.99148	-1.34315	0.0003
H	-3.22289	1.04544	0.00026
H	0.12474	-2.66268	-0.00066
H	-2.33707	-3.17535	-0.00015
O	0.73007	1.89152	-0.00025
C	-0.59322	1.89656	-0.00007
C	-1.01634	2.67844	1.2574
C	-1.01649	2.67857	-1.2574
H	-0.94281	2.04221	2.11455
H	-2.02689	3.01224	1.14662
H	-2.04465	2.9622	-1.17173
H	-0.37331	3.52413	1.38474
H	-0.41123	3.5558	-1.35241
H	-0.88769	2.06105	-2.12168
Br	2.14787	-1.78352	0.00019
I	1.57797	0.06966	0.00005

Sum of electronic and zero-point Energies=-3295.791410
Sum of electronic and thermal Energies= -3295.779163
Sum of electronic and thermal Enthalpies= -3295.778219
Sum of electronic and thermal Free Energies= -3295.831445

1-IIII-CCH

C	-2.04035	2.84698	0.05489
C	-2.5659	1.56311	0.11017
C	-1.72726	0.46123	0.01266
C	-0.37785	0.7091	-0.1241
C	0.19157	1.95846	-0.17863
C	-0.67465	3.04314	-0.09188
H	-2.70165	3.69963	0.12404
H	-3.63161	1.41448	0.22532
H	1.25822	2.08618	-0.28069
H	-0.26946	4.04403	-0.14095
O	-1.11851	-1.7315	-0.54841
C	-2.14837	-0.99464	0.08134
C	-3.42086	-1.26546	-0.71001
C	-2.3016	-1.41942	1.54222
H	-3.3038	-0.92901	-1.73841
H	-4.27435	-0.75844	-0.25949
H	-3.08641	-0.84221	2.03169
H	-3.61736	-2.33653	-0.70891
H	-2.55741	-2.47759	1.5843
H	-1.37045	-1.26233	2.08775
C	3.50893	0.60328	-0.42291
H	4.44663	1.11586	-0.47663
C	2.45626	0.02786	-0.36262
I	0.68903	-1.09642	-0.23301

Sum of electronic and zero-point Energies= -798.268480
Sum of electronic and thermal Energies= -798.255332
Sum of electronic and thermal Enthalpies=-798.254388
Sum of electronic and thermal Free Energies= -798.308818

1-IIII-CF3

C	-2.69011	2.28973	0.00005
C	-2.95457	0.92757	0.0001
C	-1.91235	-0.00045	0.00001
C	-0.64991	0.52412	-0.00006
C	-0.30538	1.85846	-0.00013
C	-1.37667	2.75141	-0.00009
H	-3.50769	2.99983	0.00013
H	-3.96181	0.52596	0.00018
H	0.70804	2.22727	-0.00024
H	-1.16527	3.81354	-0.00016
O	-1.13277	-2.20926	-0.00037
C	2.40402	0.26982	0.00001
F	3.4394	-0.54682	0.00069
F	2.44547	1.03228	1.07774
F	2.44614	1.03133	-1.07837
C	-2.19866	-1.51874	-0.00005
C	-2.95724	-1.98293	-1.2573
C	-2.95672	-1.98296	1.25751
H	-2.97973	-3.05231	-1.28578
H	-3.95818	-1.60575	-1.22951
H	-2.46089	-1.61324	-2.13015
H	-2.99293	-3.05215	1.27777
H	-3.9528	-1.59266	1.23792
H	-2.45112	-1.62661	2.1306
I	0.77117	-0.94889	0.00007

Sum of electronic and zero-point Energies=-1059.210302
Sum of electronic and thermal Energies= -1059.196431
Sum of electronic and thermal Enthalpies= -1059.195487
Sum of electronic and thermal Free Energies= -1059.251301

1-IIII-CH3

C	-2.69011	2.28973	0.00005
C	-2.95457	0.92757	0.0001
C	-1.91235	-0.00045	0.00001
C	-0.64991	0.52412	-0.00006
C	-0.30538	1.85846	-0.00013
C	-1.37667	2.75141	-0.00009
H	-3.50769	2.99983	0.00013
H	-3.96181	0.52596	0.00018
H	0.70804	2.22727	-0.00024
H	-1.16527	3.81354	-0.00016
O	-1.13277	-2.20926	-0.00037
C	2.40402	0.26982	0.00001
C	-2.19866	-1.51874	-0.00005
C	-2.95724	-1.98293	-1.2573
C	-2.95672	-1.98296	1.25751
H	-2.97973	-3.05231	-1.28578
H	-3.95818	-1.60575	-1.22951
H	-2.46089	-1.61324	-2.13015
H	-2.99293	-3.05215	1.27777
H	-3.9528	-1.59266	1.23792
H	-2.45112	-1.62661	2.1306
H	2.4376	0.8875	0.87308
H	3.24415	-0.39281	0.00056
H	2.43814	0.88672	-0.87359
I	0.77117	-0.94889	0.00007

Sum of electronic and zero-point Energies= -761.415947
Sum of electronic and thermal Energies= -761.404298
Sum of electronic and thermal Enthalpies=-761.403354
Sum of electronic and thermal Free Energies= -761.453401

1-IIII-CHCH2

C	-1.47784	2.86145	0.03034
C	-2.0225	1.58679	0.05887
C	-1.20342	0.46278	-0.00179
C	0.14941	0.69073	-0.073
C	0.74099	1.93408	-0.09943
C	-0.10271	3.03613	-0.05279
H	-2.12833	3.72443	0.0711
H	-3.09468	1.45695	0.12705
H	1.81297	2.04725	-0.15525
H	0.32231	4.02966	-0.08167
O	-0.63703	-1.78431	-0.38264
C	-1.69058	-0.9845	0.03468
C	-2.86618	-1.18298	-0.92188
C	-2.10141	-1.32814	1.47242
H	-2.57499	-0.89886	-1.93205
H	-3.74087	-0.60407	-0.62299
H	-2.91611	-0.69238	1.82282
H	-3.13356	-2.23898	-0.92145
H	-2.42141	-2.36932	1.50635
H	-1.25084	-1.20578	2.14422
C	2.87922	-0.03555	0.10832
H	3.18885	0.74836	-0.55087
C	4.3036	-0.48069	0.241
H	4.39568	-1.13578	1.082
H	4.93235	0.37326	0.38355
I	1.18041	-0.97072	-0.11678

Sum of electronic and zero-point Energies= -799.486264
Sum of electronic and thermal Energies= -799.472806
Sum of electronic and thermal Enthalpies=-799.471862
Sum of electronic and thermal Free Energies= -799.527081

1-IIII-Cl

C	2.52236	2.11339	-0.00003
C	2.66085	0.73134	-0.00002
C	1.52519	-0.07221	-0.00001
C	0.30752	0.56194	0.00001
C	0.09823	1.91869	0.00008
C	1.25733	2.69731	0.00004
H	3.40253	2.74383	-0.00006
H	3.62846	0.24255	-0.00002
H	-0.89074	2.35437	0.00019
H	1.15606	3.77559	0.00007
O	0.38663	-2.12239	-0.00002
Cl	-2.94445	0.85661	-0.00013
C	1.58381	-1.57491	-0.00003
C	2.2915	-2.1132	1.25736
C	2.29144	-2.11318	-1.25745
H	3.34533	-1.94952	-1.17123
H	1.92071	-1.60247	-2.12153
H	2.09996	-3.16154	-1.35326
H	2.05234	-3.1482	1.3857
H	3.34992	-2.00288	1.1457
H	1.96393	-1.56251	2.1143
I	-1.2452	-0.85194	0.00008

Sum of electronic and zero-point Energies=-1181.789683
Sum of electronic and thermal Energies= -1181.778447
Sum of electronic and thermal Enthalpies= -1181.777503
Sum of electronic and thermal Free Energies= -1181.827393

1-IIII-CN

C	2.38355	2.16507	-0.00011
C	2.54579	0.78572	-0.00018
C	1.42977	-0.04976	-0.00014
C	0.21531	0.57735	0.00019
C	-0.03278	1.92761	0.00009
C	1.11008	2.72995	0.00012
H	3.25324	2.81011	-0.00022
H	3.52047	0.31084	-0.00021
H	-1.02602	2.35531	0.00032
H	0.98659	3.8058	0.00018
O	0.38892	-2.14584	0.00036
C	-2.68934	0.59238	0.00006
N	-3.53721	1.37008	0.00019
C	1.54327	-1.57372	0.00002
C	2.25451	-2.10752	1.25731
C	2.25411	-2.10754	-1.2575
H	1.80196	-1.68452	2.12977
H	3.28884	-1.83553	1.22454
H	3.28438	-1.8196	-1.23413
H	1.78933	-1.69904	-2.13043
H	2.18253	-3.17486	-1.28166
H	2.16689	-3.17338	1.29121
I	-1.2004	-0.88851	-0.00018

Sum of electronic and zero-point Energies= -814.385115
Sum of electronic and thermal Energies= -814.373023
Sum of electronic and thermal Enthalpies=-814.372079
Sum of electronic and thermal Free Energies= -814.423606

1-IIII-F

C	-2.93446	-1.10859	0.00015
C	-2.52243	0.21846	0.00015
C	-1.16116	0.50593	0.
C	-0.29395	-0.55644	-0.00016
C	-0.62935	-1.88835	-0.00026
C	-2.00137	-2.14559	-0.00009
H	-3.99148	-1.34315	0.0003
H	-3.22289	1.04544	0.00026
H	0.12474	-2.66268	-0.00066
H	-2.33707	-3.17535	-0.00015
O	0.73007	1.89152	-0.00025
F	2.14787	-1.78352	0.00019
C	-0.59322	1.89656	-0.00007
C	-1.01634	2.67844	1.2574
C	-1.01649	2.67857	-1.2574
H	-0.94281	2.04221	2.11455
H	-2.02689	3.01224	1.14662
H	-2.04465	2.9622	-1.17173
H	-0.37331	3.52413	1.38474
H	-0.41123	3.5558	-1.35241
H	-0.88769	2.06105	-2.12168
I	1.69761	0.10968	0.00006

Sum of electronic and zero-point Energies= -821.427512
Sum of electronic and thermal Energies= -821.416720
Sum of electronic and thermal Enthalpies=-821.415776
Sum of electronic and thermal Free Energies= -821.464274

1-IIII-N3

C	-2.93446	-1.10859	0.00015
C	-2.52243	0.21846	0.00015
C	-1.16116	0.50593	0.
C	-0.29395	-0.55644	-0.00016
C	-0.62935	-1.88835	-0.00026
C	-2.00137	-2.14559	-0.00009
H	-3.99148	-1.34315	0.0003
H	-3.22289	1.04544	0.00026
H	0.12474	-2.66268	-0.00066
H	-2.33707	-3.17535	-0.00015
O	0.73007	1.89152	-0.00025
C	-0.59322	1.89656	-0.00007
C	-1.01634	2.67844	1.2574
C	-1.01649	2.67857	-1.2574
H	-0.94281	2.04221	2.11455
H	-2.02689	3.01224	1.14662
H	-2.04465	2.9622	-1.17173
H	-0.37331	3.52413	1.38474
H	-0.41123	3.5558	-1.35241
H	-0.88769	2.06105	-2.12168
N	2.83867	-4.1374	0.02604
N	2.56344	-3.08239	0.08667
N	2.11882	-1.68906	0.00018
I	1.57797	0.06966	0.00005

Sum of electronic and zero-point Energies= -885.745732
Sum of electronic and thermal Energies= -885.732107
Sum of electronic and thermal Enthalpies=-885.731163
Sum of electronic and thermal Free Energies= -885.787567

1-IIII-NH2

C	-2.04035	2.84698	0.05489
C	-2.5659	1.56311	0.11017
C	-1.72726	0.46123	0.01266
C	-0.37785	0.7091	-0.1241
C	0.19157	1.95846	-0.17863
C	-0.67465	3.04314	-0.09188
H	-2.70165	3.69963	0.12404
H	-3.63161	1.41448	0.22532

H	1.25822	2.08618	-0.28069
H	-0.26946	4.04403	-0.14095
O	-1.11851	-1.7315	-0.54841
C	-2.14837	-0.99464	0.08134
C	-3.42086	-1.26546	-0.71001
C	-2.3016	-1.41942	1.54222
N	2.30521	0.27775	0.07282
H	-2.55284	-2.45868	1.58355
H	-3.07183	-0.85294	2.02259
H	-1.388	-1.26529	2.07747
H	-3.61394	-2.31789	-0.70893
H	-4.25856	-0.76783	-0.26783
H	-3.30578	-0.93469	-1.72107
H	2.63855	0.74915	0.88932
H	2.63855	0.74915	-0.74368
I	0.68903	-1.09642	-0.23301

Sum of electronic and zero-point Energies= -777.480321
Sum of electronic and thermal Energies= -777.468059
Sum of electronic and thermal Enthalpies=-777.467115
Sum of electronic and thermal Free Energies= -777.519021

1-IIII-NHAc

C	-2.48749	2.65494	0.07595
C	-2.82287	1.309	0.13098
C	-1.83276	0.34168	0.02766
C	-0.53154	0.77435	-0.1168
C	-0.15068	2.09807	-0.17026
C	-1.16408	3.04518	-0.0759
H	-3.26389	3.404	0.1492
H	-3.85566	1.00869	0.25032
H	0.88405	2.37614	-0.27841
H	-0.90926	4.09471	-0.12394
O	-0.90393	-1.73597	-0.5306
C	3.33499	-0.0176	0.10895
O	3.45194	-1.21954	0.00462
C	4.50791	0.91287	0.30858
H	4.56178	1.61088	-0.52588
H	4.36296	1.49441	1.21765
H	5.42474	0.33562	0.37207
C	-2.0357	-1.16154	0.09642
C	-3.25395	-1.61798	-0.69284
C	-2.12385	-1.60413	1.55773
H	-3.18826	-1.26973	-1.72199
H	-4.17329	-1.24228	-0.24339
H	-3.28946	-2.70645	-0.69055
H	-2.22146	-2.68838	1.60003
H	-2.98465	-1.14803	2.04745
H	-1.22512	-1.31276	2.10233
N	2.18186	0.61126	0.05843
H	2.67875	1.18833	-0.58971
I	0.66853	-0.73177	-0.22621

Sum of electronic and zero-point Energies= -930.109311
Sum of electronic and thermal Energies= -930.093344
Sum of electronic and thermal Enthalpies=-930.092399
Sum of electronic and thermal Free Energies= -930.154351

1-IIII-OCF3

C	2.67388	2.50582	-0.08055
C	3.07538	1.1861	-0.2436
C	2.13864	0.16777	-0.09614
C	0.85091	0.5348	0.20224
C	0.38735	1.81389	0.38416
C	1.35008	2.81324	0.23105
H	3.39497	3.30541	-0.19349
H	4.09694	0.91345	-0.48175
H	-0.64249	2.02395	0.63
H	1.04878	3.8455	0.35987
O	1.40068	-2.06043	-0.03196
F	-3.25665	-0.89735	-0.63674
F	-2.19308	0.86999	-1.26105
F	-3.78459	1.02948	0.19587
C	-2.75499	0.26941	-0.18023
O	-1.90316	0.11538	0.81056
C	2.46298	-1.28967	-0.23514
C	3.51068	-1.74135	0.79923
C	2.97123	-1.60853	-1.65345
H	2.33942	-1.13273	-2.37409
H	3.97242	-1.2473	-1.76315
H	2.95499	-2.66711	-1.80855
H	4.48184	-1.41753	0.48797
H	3.49838	-2.80847	0.87672
H	3.27978	-1.31189	1.75167
I	-0.3585	-1.17318	0.37575

Sum of electronic and zero-point Energies=-1134.479919
Sum of electronic and thermal Energies= -1134.464516
Sum of electronic and thermal Enthalpies= -1134.463572
Sum of electronic and thermal Free Energies= -1134.524472

1-IIII-OCH3

C	2.67388	2.50582	-0.08055
C	3.07538	1.1861	-0.2436
C	2.13864	0.16777	-0.09614
C	0.85091	0.5348	0.20224
C	0.38735	1.81389	0.38416
C	1.35008	2.81324	0.23105
H	3.39497	3.30541	-0.19349
H	4.09694	0.91345	-0.48175
H	-0.64249	2.02395	0.63
H	1.04878	3.8455	0.35987
O	1.40068	-2.06043	-0.03196
C	-2.75499	0.26941	-0.18023
O	-1.90316	0.11538	0.81056
C	2.46298	-1.28967	-0.23514
C	3.51068	-1.74135	0.79923
C	2.97123	-1.60853	-1.65345
H	2.33942	-1.13273	-2.37409
H	3.97242	-1.2473	-1.76315
H	2.95499	-2.66711	-1.80855
H	4.48184	-1.41753	0.48797
H	3.49838	-2.80847	0.87672
H	3.27978	-1.31189	1.75167
H	-2.3123	0.74256	-1.03173
H	-3.5809	0.87912	0.12146
H	-3.15272	-0.65563	-0.54216
I	-0.3585	-1.17318	0.37575

Sum of electronic and zero-point Energies= -836.641386
Sum of electronic and thermal Energies= -836.627808
Sum of electronic and thermal Enthalpies=-836.626864
Sum of electronic and thermal Free Energies= -836.681859

1-IIII-OCOCF3

C	3.22289	2.62188	0.00859
C	3.68898	1.31361	0.00651
C	2.77394	0.26568	0.00105
C	1.43871	0.58809	-0.00237
C	0.91134	1.85792	-0.00002
C	1.85448	2.88734	0.0057
H	3.92673	3.44448	0.01292
H	4.74543	1.07154	0.00947
H	-0.15229	2.04153	-0.00164
H	1.50103	3.91113	0.00798
O	2.11919	-1.99141	-0.00185
C	-2.41859	-0.53165	0.0117
O	-2.5234	-1.72768	0.04512
O	-1.32746	0.16971	-0.02174
C	-3.65945	0.39701	0.00003
F	-4.78188	-0.30037	0.05516
F	-3.67375	1.13597	-1.11316
F	-3.62623	1.23022	1.0443
C	3.17219	-1.17944	0.00108
C	3.98348	-1.53747	1.26013
C	3.99077	-1.53249	-1.25466
H	3.51586	-1.11656	-2.11858
H	4.97784	-1.13005	-1.16176
H	4.04736	-2.59617	-1.35612
H	4.98789	-1.18568	1.14921
H	3.98986	-2.5995	1.39027
H	3.53637	-1.07597	2.11572
I	0.2862	-1.16738	-0.00904

Sum of electronic and zero-point Energies=-1247.801588
Sum of electronic and thermal Energies= -1247.784066
Sum of electronic and thermal Enthalpies= -1247.783122
Sum of electronic and thermal Free Energies= -1247.850135

1-IIII-OCOCH3

C	2.94595	2.24262	0.
C	3.14905	0.86891	-0.00023
C	2.05101	0.01452	-0.00014
C	0.80079	0.58421	0.00015
C	0.53351	1.93493	0.00041
C	1.65414	2.76631	0.00033
H	3.79558	2.9138	-0.00007
H	4.13833	0.42604	-0.00045
H	-0.47532	2.31781	0.00073
H	1.50439	3.83907	0.00055
O	1.00226	-2.08015	0.
C	-3.20495	0.1778	0.00021
O	-3.42588	-1.00956	0.00076
O	-1.9799	0.68529	0.00005
C	-4.26655	1.25034	-0.00099
H	-4.14463	1.88403	0.87785
H	-4.15011	1.8754	-0.88681
H	-5.24737	0.78154	0.00398
C	2.175	-1.48618	-0.00024
C	2.90387	-1.99583	1.25698
C	2.90344	-1.99553	-1.25783
H	2.55306	-1.46032	2.11434
H	3.95682	-1.84118	1.14612
H	2.70817	-3.04008	1.38401
H	2.75561	-3.05101	-1.35272
H	3.94968	-1.78816	-1.17256
H	2.51115	-1.50132	-2.12198
I	-0.68643	-0.89842	0.00013

Sum of electronic and zero-point Energies= -950.009197
Sum of electronic and thermal Energies= -949.994332
Sum of electronic and thermal Enthalpies=-949.993388

Sum of electronic and thermal Free Energies= -950.052113

1-IIII-OCOPh

C	-2.48749	2.65494	0.07595
C	-2.82287	1.309	0.13098
C	-1.83276	0.34168	0.02766
C	-0.53154	0.77435	-0.1168
C	-0.15068	2.09807	-0.17026
C	-1.16408	3.04518	-0.0759
H	-3.26389	3.404	0.1492
H	-3.85566	1.00869	0.25032
H	0.88405	2.37614	-0.27841
H	-0.90926	4.09471	-0.12394
O	-0.90393	-1.73597	-0.5306
C	3.33499	-0.0176	0.10895
O	3.45212	-1.21935	0.00267
O	2.18163	0.61103	0.06075
C	-2.0357	-1.16154	0.09642
C	-3.25395	-1.61798	-0.69284
C	-2.12385	-1.60413	1.55773
H	-3.18826	-1.26973	-1.72199
H	-4.17329	-1.24228	-0.24339
H	-3.28946	-2.70645	-0.69055
H	-2.22146	-2.68838	1.60003
H	-2.98465	-1.14803	2.04745
H	-1.22512	-1.31276	2.10233
C	4.53088	0.93109	0.31249
C	4.31964	2.3056	0.42461
C	5.82519	0.41625	0.38486
C	5.40258	3.16506	0.60838
H	3.29918	2.71126	0.36674
C	6.90848	1.27574	0.56968
H	5.99184	-0.66707	0.29669
C	6.69741	2.64998	0.68131
H	5.23622	4.24854	0.69614
H	7.92884	0.86948	0.62712
H	7.55112	3.32782	0.82627
I	0.66853	-0.73177	-0.22621

Sum of electronic and zero-point Energies=-1141.688453
Sum of electronic and thermal Energies= -1141.670039
Sum of electronic and thermal Enthalpies= -1141.669095
Sum of electronic and thermal Free Energies= -1141.737259

1-IIII-OH

C	2.85263	-1.32247	0.00438
C	2.53897	0.03132	0.02066
C	1.20519	0.42867	0.01343
C	0.26118	-0.56546	0.00447
C	0.49803	-1.91871	-0.02637
C	1.8446	-2.28523	-0.02237
H	3.88939	-1.63508	0.00637
H	3.29795	0.80511	0.03341
H	-0.31078	-2.63443	-0.06185
H	2.09873	-3.33788	-0.04592
O	-0.53232	1.99106	-0.02484
O	-2.35941	-1.6203	-0.02187
H	-2.69235	-1.77691	0.86934
C	0.76361	1.8772	0.00979
C	1.26116	2.64337	-1.22998
C	1.22455	2.60844	1.28432
H	0.69564	3.54465	-1.343
H	1.1371	2.033	-2.10002
H	2.29644	2.88479	-1.10834
H	2.27402	2.80736	1.2216
H	1.02831	1.99455	2.13845
H	0.69158	3.53147	1.37846
I	-1.68965	0.21143	-0.0219

Sum of electronic and zero-point Energies= -797.378136
Sum of electronic and thermal Energies= -797.366089
Sum of electronic and thermal Enthalpies=-797.365145
Sum of electronic and thermal Free Energies= -797.416495

1-IIII-OTf

C	-2.93446	-1.10859	0.00015
C	-2.52243	0.21846	0.00015
C	-1.16116	0.50593	0.
C	-0.29395	-0.55644	-0.00016
C	-0.62935	-1.88835	-0.00026
C	-2.00137	-2.14559	-0.00009
H	-3.99148	-1.34315	0.0003
H	-3.22289	1.04544	0.00026
H	0.12474	-2.66268	-0.00066
H	-2.33707	-3.17535	-0.00015
O	0.73007	1.89152	-0.00025
C	-0.59322	1.89656	-0.00007
C	-1.01634	2.67844	1.2574
C	-1.01649	2.67857	-1.2574
H	-0.94281	2.04221	2.11455
H	-2.02689	3.01224	1.14662
H	-2.04465	2.9622	-1.17173
H	-0.37331	3.52413	1.38474
H	-0.41123	3.5558	-1.35241
H	-0.88769	2.06105	-2.12168
O	2.10706	-1.65082	0.00018
S	3.59206	-1.98206	0.66643
O	3.32762	-2.88312	1.67648
O	4.05277	-0.70442	0.94415
C	4.38426	-2.66762	-0.56896
F	4.64947	-1.73381	-1.50712
F	5.54243	-3.19802	-0.12195
F	3.62112	-3.6424	-1.1074
I	1.57797	0.06966	0.00005

Sum of electronic and zero-point Energies=-1683.085001
Sum of electronic and thermal Energies= -1683.066326
Sum of electronic and thermal Enthalpies= -1683.065382
Sum of electronic and thermal Free Energies= -1683.134509

1-IIII-OTs

C	-2.93446	-1.10859	0.00015
C	-2.52243	0.21846	0.00015
C	-1.16116	0.50593	0.
C	-0.29395	-0.55644	-0.00016
C	-0.62935	-1.88835	-0.00026
C	-2.00137	-2.14559	-0.00009
H	-3.99148	-1.34315	0.0003
H	-3.22289	1.04544	0.00026
H	0.12474	-2.66268	-0.00066
H	-2.33707	-3.17535	-0.00015
O	0.73007	1.89152	-0.00025
C	-0.59322	1.89656	-0.00007
C	-1.01634	2.67844	1.2574
C	-1.01649	2.67857	-1.2574
H	-0.94281	2.04221	2.11455
H	-2.02689	3.01224	1.14662
H	-2.04465	2.9622	-1.17173
H	-0.37331	3.52413	1.38474
H	-0.41123	3.5558	-1.35241
H	-0.88769	2.06105	-2.12168
O	2.10706	-1.65082	0.00018
S	3.59206	-1.98206	0.66643
O	3.32762	-2.88312	1.67648
O	4.05277	-0.70442	0.94415
C	4.39898	-2.68036	-0.59191
C	4.29095	-4.05732	-0.83881
C	5.21583	-1.91719	-1.44026
C	4.97031	-4.65259	-1.89504
H	3.6592	-4.67373	-0.17344
C	5.89937	-2.50427	-2.49751
H	5.31971	-0.83289	-1.25251
C	5.78031	-3.88013	-2.73787
H	4.87315	-5.73479	-2.07074
H	6.53777	-1.88846	-3.14913
C	6.49103	-4.50754	-3.87454
H	7.41771	-3.93858	-4.1338
H	6.77579	-5.56208	-3.63595
H	5.82389	-4.51998	-4.77423
I	1.57797	0.06966	0.00005

Sum of electronic and zero-point Energies=-1616.258238
Sum of electronic and thermal Energies= -1616.237500
Sum of electronic and thermal Enthalpies= -1616.236556
Sum of electronic and thermal Free Energies= -1616.310859

1-IIII-radical

C	-2.93446	-1.10859	0.00015
C	-2.52243	0.21846	0.00015
C	-1.16116	0.50593	0.
C	-0.29395	-0.55644	-0.00016
C	-0.62935	-1.88835	-0.00026
C	-2.00137	-2.14559	-0.00009
H	-3.99148	-1.34315	0.0003
H	-3.22289	1.04544	0.00026
H	0.12474	-2.66268	-0.00066
H	-2.33707	-3.17535	-0.00015
O	0.73007	1.89152	-0.00025
C	-0.59322	1.89656	-0.00007
C	-1.01634	2.67844	1.2574
C	-1.01649	2.67857	-1.2574
H	-0.94281	2.04221	2.11455
H	-2.02689	3.01224	1.14662
H	-2.04465	2.9622	-1.17173
H	-0.37331	3.52413	1.38474
H	-0.41123	3.5558	-1.35241
H	-0.88769	2.06105	-2.12168
I	1.69761	0.10968	0.00006

Sum of electronic and zero-point Energies= -721.570728
Sum of electronic and thermal Energies= -721.560042
Sum of electronic and thermal Enthalpies=-721.559098
Sum of electronic and thermal Free Energies= -721.608626

1-IIII-SCF3

C	2.78872	2.53347	-0.0787
C	3.18535	1.22372	-0.31382
C	2.27986	0.18067	-0.13941
C	1.0132	0.52339	0.25857
C	0.55417	1.79211	0.51366
C	1.48729	2.81374	0.3311

H	3.49368	3.34408	-0.21405
H	4.18916	0.96587	-0.63234
H	-0.45947	1.99472	0.82838
H	1.17977	3.83591	0.514
O	1.66601	-2.0776	-0.15099
F	-3.93551	0.92128	-0.45801
F	-2.96359	-0.85487	-1.19914
F	-1.93008	1.02411	-1.25123
C	-2.77223	0.27822	-0.52401
S	-2.16147	-0.00054	1.15757
C	2.65512	-1.26809	-0.37758
C	3.78298	-1.73615	0.56074
C	3.06597	-1.52096	-1.84007
H	3.60519	-1.36102	1.54693
H	2.36564	-1.04553	-2.49459
H	3.07479	-2.57372	-2.03113
H	4.04296	-1.11931	-2.01062
H	4.72122	-1.36702	0.20249
H	3.80616	-2.80563	0.58475
I	-0.18758	-1.1862	0.47172

Sum of electronic and zero-point Energies=-1457.430136
Sum of electronic and thermal Energies= -1457.413990
Sum of electronic and thermal Enthalpies= -1457.413046
Sum of electronic and thermal Free Energies= -1457.475837

2-BrIII-Br

C	-2.93446	-1.10859	0.00015
C	-2.52243	0.21846	0.00015
C	-1.16116	0.50593	0.
C	-0.29395	-0.55644	-0.00016
C	-0.62935	-1.88835	-0.00026
C	-2.00137	-2.14559	-0.00009
H	-3.99148	-1.34315	0.0003
H	-3.22289	1.04544	0.00026
H	0.12474	-2.66268	-0.00066
H	-2.33707	-3.17535	-0.00015
Br	1.57797	0.06966	0.00005
O	0.73007	1.89152	-0.00025
C	-0.59322	1.89656	-0.00007
C	-1.01634	2.67844	1.2574
C	-1.01649	2.67857	-1.2574
F	-0.20504	3.74543	1.41806
F	-2.29133	3.09959	1.11763
F	-0.92357	1.87572	2.33885
F	-2.3137	3.03643	-1.14931
F	-0.25284	3.78535	-1.37727
F	-0.85398	1.89946	-2.34785
Br	2.14787	-1.78352	0.00019

Sum of electronic and zero-point Energies=-6167.912754
Sum of electronic and thermal Energies= -6167.896542
Sum of electronic and thermal Enthalpies= -6167.895598
Sum of electronic and thermal Free Energies= -6167.960259

2-BrIII-CCH

C	-2.04035	2.84698	0.05489
C	-2.5659	1.56311	0.11017
C	-1.72726	0.46123	0.01266
C	-0.37785	0.7091	-0.1241
C	0.19157	1.95846	-0.17863
C	-0.67465	3.04314	-0.09188
H	-2.70165	3.69963	0.12404
H	-3.63161	1.41448	0.22532
H	1.25822	2.08618	-0.28069
H	-0.26946	4.04403	-0.14095
Br	0.61073	-0.9639	-0.22502
O	-1.11851	-1.7315	-0.54841
C	-2.14837	-0.99464	0.08134
C	-3.42086	-1.26546	-0.71001
C	-2.3016	-1.41942	1.54222
C	3.50893	0.60328	-0.42291
H	4.44663	1.11586	-0.47663
C	2.45626	0.02786	-0.36262
F	-1.14893	-1.22495	2.21753
F	-2.61859	-2.73064	1.59436
F	-3.27338	-0.7047	2.14829
F	-3.66447	-2.5933	-0.70865
F	-4.47777	-0.6376	-0.15212
F	-3.27566	-0.84813	-1.98565

Sum of electronic and zero-point Energies=-3670.401642
Sum of electronic and thermal Energies= -3670.384692
Sum of electronic and thermal Enthalpies= -3670.383748
Sum of electronic and thermal Free Energies= -3670.448164

2-BrIII-CF3

C	-2.69011	2.28973	0.00005
C	-2.95457	0.92757	0.0001
C	-1.91235	-0.00045	0.00001
C	-0.64991	0.52412	-0.00006
C	-0.30538	1.85846	-0.00013
C	-1.37667	2.75141	-0.00009
H	-3.50769	2.99983	0.00013
H	-3.96181	0.52596	0.00018
H	0.70804	2.22727	-0.00024
H	-1.16527	3.81354	-0.00016
Br	0.77117	-0.94889	0.00007
O	-1.13277	-2.20926	-0.00037
C	2.40402	0.26982	0.00001
F	3.4394	-0.54682	0.00069
F	2.44547	1.03228	1.07774
F	2.44614	1.03133	-1.07837
C	-2.19866	-1.51874	-0.00005
C	-2.95724	-1.98293	-1.2573
C	-2.95672	-1.98296	1.25751
F	-4.21346	-1.49053	1.2328
F	-3.0024	-3.33194	1.28307
F	-2.31882	-1.53335	2.35907
F	-2.98561	-3.33215	-1.29324
F	-4.2201	-1.50705	-1.22224
F	-2.33101	-1.5165	-2.35856

Sum of electronic and zero-point Energies=-3931.349375
Sum of electronic and thermal Energies= -3931.330898
Sum of electronic and thermal Enthalpies= -3931.329953
Sum of electronic and thermal Free Energies= -3931.398011

2-BrIII-CH3

C	-2.69011	2.28973	0.00005
C	-2.95457	0.92757	0.0001
C	-1.91235	-0.00045	0.00001
C	-0.64991	0.52412	-0.00006
C	-0.30538	1.85846	-0.00013
C	-1.37667	2.75141	-0.00009
H	-3.50769	2.99983	0.00013
H	-3.96181	0.52596	0.00018
H	0.70804	2.22727	-0.00024
H	-1.16527	3.81354	-0.00016
Br	0.77117	-0.94889	0.00007
O	-1.13277	-2.20926	-0.00037
C	2.40402	0.26982	0.00001
C	-2.19866	-1.51874	-0.00005
C	-2.95724	-1.98293	-1.2573
C	-2.95672	-1.98296	1.25751
F	-4.21346	-1.49053	1.2328
F	-3.0024	-3.33194	1.28307
F	-2.31882	-1.53335	2.35907
F	-2.98561	-3.33215	-1.29324
F	-4.2201	-1.50705	-1.22224
F	-2.33101	-1.5165	-2.35856
H	2.4376	0.8875	0.87308
H	3.24415	-0.39281	0.00056
H	2.43814	0.88672	-0.87359

Sum of electronic and zero-point Energies=-3633.562198
Sum of electronic and thermal Energies= -3633.545872
Sum of electronic and thermal Enthalpies= -3633.544928
Sum of electronic and thermal Free Energies= -3633.607380

2-BrIII-CHCH2

C	-1.47784	2.86145	0.03034
C	-2.0225	1.58679	0.05887
C	-1.20342	0.46278	-0.00179
C	0.14941	0.69073	-0.073
C	0.74099	1.93408	-0.09943
C	-0.10271	3.03613	-0.05279
H	-2.12833	3.72443	0.0711
H	-3.09468	1.45695	0.12705
H	1.81297	2.04725	-0.15525
H	0.32231	4.02966	-0.08167
O	-0.63703	-1.78431	-0.38264
C	-1.69058	-0.9845	0.03468
C	-2.86618	-1.18298	-0.92188
C	-2.10141	-1.32814	1.47242
C	2.87922	-0.03555	0.10832
H	3.18885	0.74836	-0.55087
C	4.3036	-0.48069	0.241
H	4.39568	-1.13578	1.082
H	4.93235	0.37326	0.38355
F	-2.50521	-0.83077	-2.17413
F	-3.94885	-0.46642	-0.55192
F	-3.19754	-2.49169	-0.92134
F	-2.49782	-2.61795	1.51445
F	-1.04869	-1.1767	2.30389
F	-3.10934	-0.5416	1.90593
Br	1.18041	-0.97072	-0.11678

Sum of electronic and zero-point Energies=-3671.632788
Sum of electronic and thermal Energies= -3671.615527
Sum of electronic and thermal Enthalpies= -3671.614583
Sum of electronic and thermal Free Energies= -3671.679263

2-BrIII-Cl

C	2.52236	2.11339	-0.00003
C	2.66085	0.73134	-0.00002
C	1.52519	-0.07221	-0.00001
C	0.30752	0.56194	0.00001
C	0.09823	1.91869	0.00008
C	1.25733	2.69731	0.00004
H	3.40253	2.74383	-0.00006
H	3.62846	0.24255	-0.00002

H	-0.89074	2.35437	0.00019
H	1.15606	3.77559	0.00007
Br	-1.17132	-0.78467	0.00008
O	0.38663	-2.12239	-0.00002
Cl	-2.94445	0.85661	-0.00013
C	1.58381	-1.57491	-0.00003
C	2.2915	-2.1132	1.25736
C	2.29144	-2.11318	-1.25745
F	1.98976	-3.41904	1.41929
F	3.62689	-1.97401	1.11649
F	1.87822	-1.41841	2.33854
F	3.62112	-1.9067	-1.14867
F	2.04985	-3.43587	-1.37833
F	1.82369	-1.46882	-2.34765

Sum of electronic and zero-point Energies=-4053.910953
Sum of electronic and thermal Energies= -4053.895932
Sum of electronic and thermal Enthalpies= -4053.894987
Sum of electronic and thermal Free Energies= -4053.953943

2-BrIII-CN

C	2.38355	2.16507	-0.00011
C	2.54579	0.78572	-0.00018
C	1.42977	-0.04976	-0.00014
C	0.21531	0.57735	0.00019
C	-0.03278	1.92761	0.00009
C	1.11008	2.72995	0.00012
H	3.25324	2.81011	-0.00022
H	3.52047	0.31084	-0.00021
H	-1.02602	2.35531	0.00032
H	0.98659	3.8058	0.00018
Br	-1.29133	-0.79808	-0.00016
O	0.38892	-2.14584	0.00036
C	-2.68934	0.59238	0.00006
N	-3.53721	1.37008	0.00019
C	1.54327	-1.57372	0.00002
C	2.25451	-2.10752	1.25731
C	2.25411	-2.10754	-1.2575
F	3.55951	-1.76435	1.21596
F	2.14397	-3.4523	1.30008
F	1.68353	-1.57383	2.35807
F	3.55398	-1.74425	-1.22801
F	2.1638	-3.45417	-1.28798
F	1.6677	-1.59215	-2.35886

Sum of electronic and zero-point Energies=-3686.511142
Sum of electronic and thermal Energies= -3686.494414
Sum of electronic and thermal Enthalpies= -3686.493470
Sum of electronic and thermal Free Energies= -3686.557889

2-BrIII-F

C	-2.93446	-1.10859	0.00015
C	-2.52243	0.21846	0.00015
C	-1.16116	0.50593	0.
C	-0.29395	-0.55644	-0.00016
C	-0.62935	-1.88835	-0.00026
C	-2.00137	-2.14559	-0.00009
H	-3.99148	-1.34315	0.0003
H	-3.22289	1.04544	0.00026
H	0.12474	-2.66268	-0.00066
H	-2.33707	-3.17535	-0.00015
Br	1.57797	0.06966	0.00005
O	0.73007	1.89152	-0.00025
F	2.14787	-1.78352	0.00019
C	-0.59322	1.89656	-0.00007
C	-1.01634	2.67844	1.2574
C	-1.01649	2.67857	-1.2574
F	-0.20504	3.74543	1.41806
F	-2.29133	3.09959	1.11763
F	-0.92357	1.87572	2.33885
F	-2.3137	3.03643	-1.14931
F	-0.25284	3.78535	-1.37727
F	-0.85398	1.89946	-2.34785

Sum of electronic and zero-point Energies=-3693.545738
Sum of electronic and thermal Energies= -3693.530358
Sum of electronic and thermal Enthalpies= -3693.529414
Sum of electronic and thermal Free Energies= -3693.590052

2-BrIII-N3

C	-2.93446	-1.10859	0.00015
C	-2.52243	0.21846	0.00015
C	-1.16116	0.50593	0.
C	-0.29395	-0.55644	-0.00016
C	-0.62935	-1.88835	-0.00026
C	-2.00137	-2.14559	-0.00009
H	-3.99148	-1.34315	0.0003
H	-3.22289	1.04544	0.00026
H	0.12474	-2.66268	-0.00066
H	-2.33707	-3.17535	-0.00015
Br	1.57797	0.06966	0.00005
O	0.73007	1.89152	-0.00025
C	-0.59322	1.89656	-0.00007
C	-1.01634	2.67844	1.2574
C	-1.01649	2.67857	-1.2574
F	-0.20504	3.74543	1.41806
F	-2.29133	3.09959	1.11763
F	-0.92357	1.87572	2.33885
F	-2.3137	3.03643	-1.14931
F	-0.25284	3.78535	-1.37727
F	-0.85398	1.89946	-2.34785
N	2.11882	-1.68906	0.00018
N	3.3197	-1.96422	-0.00165
N	3.73122	-3.30237	-0.00011

Sum of electronic and zero-point Energies=-3757.870465
Sum of electronic and thermal Energies= -3757.852963
Sum of electronic and thermal Enthalpies= -3757.852019
Sum of electronic and thermal Free Energies= -3757.918402

2-BrIII-NH2

C	-2.04035	2.84698	0.05489
C	-2.5659	1.56311	0.11017
C	-1.72726	0.46123	0.01266
C	-0.37785	0.7091	-0.1241
C	0.19157	1.95846	-0.17863
C	-0.67465	3.04314	-0.09188
H	-2.70165	3.69963	0.12404
H	-3.63161	1.41448	0.22532
H	1.25822	2.08618	-0.28069
H	-0.26946	4.04403	-0.14095
Br	0.61073	-0.9639	-0.22502
O	-1.11851	-1.7315	-0.54841
C	-2.14837	-0.99464	0.08134
C	-3.42086	-1.26546	-0.71001
C	-2.3016	-1.41942	1.54222
F	-1.14893	-1.22495	2.21753
F	-2.61859	-2.73064	1.59436
F	-3.27338	-0.7047	2.14829
F	-3.66447	-2.5933	-0.70865
F	-4.47777	-0.6376	-0.15212
F	-3.27566	-0.84813	-1.98565
N	2.30521	0.27775	0.07282
H	2.63855	0.74915	0.88932
H	2.63855	0.74915	-0.74368

Sum of electronic and zero-point Energies=-3649.612931
Sum of electronic and thermal Energies= -3649.596887
Sum of electronic and thermal Enthalpies= -3649.595943
Sum of electronic and thermal Free Energies= -3649.658304

2-BrIII-NHAc

C	-0.85473	3.41472	0.00147
C	-1.53185	2.20509	0.00111
C	-0.81706	1.01329	0.00004
C	0.56125	1.0802	-0.00047
C	1.26756	2.26562	-0.00011
C	0.53151	3.44327	0.00083
H	-1.41506	4.33894	0.00226
H	-2.61108	2.18285	0.00168
H	2.34335	2.27341	-0.0005
H	1.05628	4.38841	0.00108
Br	1.39201	-0.65847	-0.00148
O	-0.43038	-1.3286	-0.00333
C	4.12689	-0.63449	-0.00053
O	3.94053	-1.82591	-0.00192
C	5.48582	0.01505	0.00106
H	5.58621	0.65234	-0.87609
H	5.5874	0.6443	0.88391
H	6.2516	-0.75352	-0.00275
C	-1.42375	-0.37925	-0.00049
C	-2.26756	-0.58778	-1.28126
C	-2.26336	-0.59025	1.28264
F	-2.74982	-1.81897	-1.35607
F	-3.29743	0.26172	-1.35504
F	-1.49524	-0.38027	-2.34723
F	-3.29196	0.26023	1.36229
F	-2.74673	-1.82109	1.35599
F	-1.48702	-0.38636	2.34643
N	3.13388	0.26183	0.00013
H	3.43394	1.09138	0.47109

Sum of electronic and zero-point Energies=-3802.236532
Sum of electronic and thermal Energies= -3802.216768
Sum of electronic and thermal Enthalpies= -3802.215824
Sum of electronic and thermal Free Energies= -3802.286977

2-BrIII-OCF3

C	2.67388	2.50582	-0.08055
C	3.07538	1.1861	-0.2436
C	2.13864	0.16777	-0.09614
C	0.85091	0.5348	0.20224
C	0.38735	1.81389	0.38416
C	1.35008	2.81324	0.23105
H	3.39497	3.30541	-0.19349
H	4.09694	0.91345	-0.48175
H	-0.64249	2.02395	0.63
H	1.04878	3.8455	0.35987
Br	-0.29449	-1.08279	0.36657
O	1.40068	-2.06043	-0.03196
F	-3.25665	-0.89735	-0.63674
F	-2.19308	0.86999	-1.26105

F	-3.78459	1.02948	0.19587
C	-2.75499	0.26941	-0.18023
O	-1.90316	0.11538	0.81056
C	2.46298	-1.28967	-0.23514
C	3.51068	-1.74135	0.79923
C	2.97123	-1.60853	-1.65345
F	3.49516	-3.08772	0.897
F	4.73598	-1.33279	0.40652
F	3.21935	-1.19951	2.0009
F	4.23441	-1.15277	-1.79186
F	2.95074	-2.94412	-1.84914
F	2.17408	-1.00823	-2.56267

Sum of electronic and zero-point Energies=-4006.596763
Sum of electronic and thermal Energies= -4006.577585
Sum of electronic and thermal Enthalpies= -4006.576641
Sum of electronic and thermal Free Energies= -4006.646961

2-BrIII-OCH3

C	2.67388	2.50582	-0.08055
C	3.07538	1.1861	-0.2436
C	2.13864	0.16777	-0.09614
C	0.85091	0.5348	0.20224
C	0.38735	1.81389	0.38416
C	1.35008	2.81324	0.23105
H	3.39497	3.30541	-0.19349
H	4.09694	0.91345	-0.48175
H	-0.64249	2.02395	0.63
H	1.04878	3.8455	0.35987
Br	-0.29449	-1.08279	0.36657
O	1.40068	-2.06043	-0.03196
C	-2.75499	0.26941	-0.18023
O	-1.90316	0.11538	0.81056
C	2.46298	-1.28967	-0.23514
C	3.51068	-1.74135	0.79923
C	2.97123	-1.60853	-1.65345
F	3.49516	-3.08772	0.897
F	4.73598	-1.33279	0.40652
F	3.21935	-1.19951	2.0009
F	4.23441	-1.15277	-1.79186
F	2.95074	-2.94412	-1.84914
F	2.17408	-1.00823	-2.56267
H	-2.3123	0.74256	-1.03173
H	-3.5809	0.87912	0.12146
H	-3.15272	-0.65563	-0.54216

Sum of electronic and zero-point Energies=-3708.768424
Sum of electronic and thermal Energies= -3708.751046
Sum of electronic and thermal Enthalpies= -3708.750102
Sum of electronic and thermal Free Energies= -3708.814828

2-BrIII-OCOCF3

C	3.22289	2.62188	0.00859
C	3.68898	1.31361	0.00651
C	2.77394	0.26568	0.00105
C	1.43871	0.58809	-0.00237
C	0.91134	1.85792	-0.00002
C	1.85448	2.88734	0.0057
H	3.92673	3.44448	0.01292
H	4.74543	1.07154	0.00947
H	-0.15229	2.04153	-0.00164
H	1.50103	3.91113	0.00798
Br	0.34895	-1.0718	-0.00868
O	2.11919	-1.99141	-0.00185
C	-2.41859	-0.53165	0.0117
O	-2.5234	-1.72768	0.04512
O	-1.32746	0.16971	-0.02174
C	-3.65945	0.39701	0.00003
F	-4.78188	-0.30037	0.05516
F	-3.67375	1.13597	-1.11316
F	-3.62623	1.23022	1.0443
C	3.17219	-1.17944	0.00108
C	3.98348	-1.53747	1.26013
C	3.99077	-1.53249	-1.25466
F	3.99153	-2.87742	1.42432
F	5.25073	-1.09363	1.12019
F	3.41937	-0.95521	2.33961
F	5.23614	-1.02474	-1.13746
F	4.06216	-2.87451	-1.38267
F	3.39159	-1.00772	-2.34466

Sum of electronic and zero-point Energies=-4119.917045
Sum of electronic and thermal Energies= -4119.895691
Sum of electronic and thermal Enthalpies= -4119.894747
Sum of electronic and thermal Free Energies= -4119.971331

2-BrIII-OCOCH3

C	2.94595	2.24262	0.
C	3.14905	0.86891	-0.00023
C	2.05101	0.01452	-0.00014
C	0.80079	0.58421	0.00015
C	0.53351	1.93493	0.00041
C	1.65414	2.76631	0.00033
H	3.79558	2.9138	-0.00007
H	4.13833	0.42604	-0.00045
H	-0.47532	2.31781	0.00073
H	1.50439	3.83907	0.00055
Br	-0.60316	-0.8154	0.00013
O	1.00226	-2.08015	0.
C	-3.20495	0.1778	0.00021
O	-3.42588	-1.00956	0.00076
O	-1.9799	0.68529	0.00005
C	-4.26655	1.25034	-0.00099
H	-4.14463	1.88403	0.87785
H	-4.15011	1.8754	-0.88681
H	-5.24737	0.78154	0.00398
C	2.175	-1.48618	-0.00024
C	2.90387	-1.99583	1.25698
C	2.90344	-1.99553	-1.25783
F	2.65696	-3.31334	1.41726
F	4.23236	-1.80072	1.1171
F	2.46125	-1.32019	2.3387
F	4.22347	-1.73389	-1.15024
F	2.71692	-3.32721	-1.37755
F	2.40849	-1.372	-2.34812

Sum of electronic and zero-point Energies=-3822.128331
Sum of electronic and thermal Energies= -3822.109721
Sum of electronic and thermal Enthalpies= -3822.108777
Sum of electronic and thermal Free Energies= -3822.176508

2-BrIII-OCOPh

C	-0.85473	3.41472	0.00147
C	-1.53185	2.20509	0.00111
C	-0.81706	1.01329	0.00004
C	0.56125	1.0802	-0.00047
C	1.26756	2.26562	-0.00011
C	0.53151	3.44327	0.00083
H	-1.41506	4.33894	0.00226
H	-2.61108	2.18285	0.00168
H	2.34335	2.27341	-0.0005
H	1.05628	4.38841	0.00108
Br	1.39201	-0.65847	-0.00148
O	-0.43038	-1.3286	-0.00333
C	4.1119	-0.78144	-0.00993
O	3.89449	-1.96759	-0.01207
O	3.14925	0.1342	-0.00582
C	-1.42375	-0.37925	-0.00049
C	-2.26756	-0.58778	-1.28126
C	-2.26336	-0.59025	1.28264
F	-2.74982	-1.81897	-1.35607
F	-3.29743	0.26172	-1.35504
F	-1.49524	-0.38027	-2.34723
F	-3.29196	0.26023	1.36229
F	-2.74673	-1.82109	1.35599
F	-1.48702	-0.38636	2.34643
C	5.5182	-0.15382	-0.01118
C	6.64759	-0.97293	-0.01539
C	5.66288	1.23348	-0.00823
C	7.92134	-0.40478	-0.01595
H	6.5332	-2.06662	-0.01694
C	6.93694	1.80197	-0.00979
H	4.77286	1.87921	-0.0051
C	8.06611	0.98312	-0.01351
H	8.81158	-1.05034	-0.01863
H	7.05071	2.89582	-0.00787
H	9.07051	1.43086	-0.01401

Sum of electronic and zero-point Energies=-4013.806084
Sum of electronic and thermal Energies= -4013.783781
Sum of electronic and thermal Enthalpies= -4013.782837
Sum of electronic and thermal Free Energies= -4013.861060

2-BrIII-OH

C	2.85263	-1.32247	0.00438
C	2.53897	0.03132	0.02066
C	1.20519	0.42867	0.01343
C	0.26118	-0.56546	0.00447
C	0.49803	-1.91871	-0.02637
C	1.8446	-2.28523	-0.02237
H	3.88939	-1.63508	0.00637
H	3.29795	0.80511	0.03341
H	-0.31078	-2.63443	-0.06185
H	2.09873	-3.33788	-0.04592
Br	-1.58476	0.16966	-0.02049
O	-0.53232	1.99106	-0.02484
O	-2.35941	-1.6203	-0.02187
H	-2.69235	-1.77691	0.86934
C	0.76361	1.8772	0.00979
C	1.26116	2.64337	-1.22998
C	1.22455	2.60844	1.28432
F	0.54765	3.7805	-1.37257
F	2.56736	2.94797	-1.0765
F	1.10463	1.87328	-2.32769
F	2.54865	2.85941	1.20518
F	0.55211	3.773	1.40309
F	0.97696	1.83391	2.36196

Sum of electronic and zero-point Energies=-3669.503150
Sum of electronic and thermal Energies= -3669.487285
Sum of electronic and thermal Enthalpies= -3669.486341
Sum of electronic and thermal Free Energies= -3669.547539

2-BrIII-OTf

C	-2.93446	-1.10859	0.00015
C	-2.52243	0.21846	0.00015
C	-1.16116	0.50593	0.
C	-0.29395	-0.55644	-0.00016
C	-0.62935	-1.88835	-0.00026
C	-2.00137	-2.14559	-0.00009
H	-3.99148	-1.34315	0.0003
H	-3.22289	1.04544	0.00026
H	0.12474	-2.66268	-0.00066
H	-2.33707	-3.17535	-0.00015
Br	1.57797	0.06966	0.00005
O	0.73007	1.89152	-0.00025
C	-0.59322	1.89656	-0.00007
C	-1.01634	2.67844	1.2574
C	-1.01649	2.67857	-1.2574
F	-0.20504	3.74543	1.41806
F	-2.29133	3.09959	1.11763
F	-0.92357	1.87572	2.33885
F	-2.3137	3.03643	-1.14931
F	-0.25284	3.78535	-1.37727
F	-0.85398	1.89946	-2.34785
O	2.10706	-1.65082	0.00018
S	3.58271	-1.98493	0.6855
O	3.30434	-2.89027	1.68795
O	4.03949	-0.7085	0.975
C	4.39195	-2.66525	-0.54171
F	4.69886	-1.72121	-1.45663
F	5.52792	-3.2263	-0.07556
F	3.62352	-3.61526	-1.11573

Sum of electronic and zero-point Energies=-4555.197793
Sum of electronic and thermal Energies= -4555.175232
Sum of electronic and thermal Enthalpies= -4555.174288
Sum of electronic and thermal Free Energies= -4555.253391

2-BrIII-OTs

C	-2.93446	-1.10859	0.00015
C	-2.52243	0.21846	0.00015
C	-1.16116	0.50593	0.
C	-0.29395	-0.55644	-0.00016
C	-0.62935	-1.88835	-0.00026
C	-2.00137	-2.14559	-0.00009
H	-3.99148	-1.34315	0.0003
H	-3.22289	1.04544	0.00026
H	0.12474	-2.66268	-0.00066
H	-2.33707	-3.17535	-0.00015
Br	1.57797	0.06966	0.00005
O	0.73007	1.89152	-0.00025
C	-0.59322	1.89656	-0.00007
C	-1.01634	2.67844	1.2574
C	-1.01649	2.67857	-1.2574
F	-0.20504	3.74543	1.41806
F	-2.29133	3.09959	1.11763
F	-0.92357	1.87572	2.33885
F	-2.3137	3.03643	-1.14931
F	-0.25284	3.78535	-1.37727
F	-0.85398	1.89946	-2.34785
O	2.10706	-1.65082	0.00018
S	3.58271	-1.98493	0.6855
O	3.30434	-2.89027	1.68795
O	4.03949	-0.7085	0.975
C	4.40699	-2.6779	-0.56451
C	4.30243	-4.05379	-0.81874
C	5.23548	-1.91115	-1.39822
C	4.99636	-4.64458	-1.868
H	3.66155	-4.67301	-0.16481
C	5.9336	-2.49374	-2.4484
H	5.33672	-0.82766	-1.20443
C	5.81792	-3.86856	-2.69624
H	4.90167	-5.72602	-2.04962
H	6.58094	-1.87518	-3.08849
C	6.54432	-4.49115	-3.82562
H	7.47449	-3.92111	-4.06961
H	6.82578	-5.5467	-3.58761
H	5.8897	-4.49974	-4.7345

Sum of electronic and zero-point Energies=-4488.374443
Sum of electronic and thermal Energies= -4488.349000
Sum of electronic and thermal Enthalpies= -4488.348056
Sum of electronic and thermal Free Energies= -4488.434443

2-BrIII-radical

C	-2.93446	-1.10859	0.00015
C	-2.52243	0.21846	0.00015
C	-1.16116	0.50593	0.
C	-0.29395	-0.55644	-0.00016
C	-0.62935	-1.88835	-0.00026
C	-2.00137	-2.14559	-0.00009
H	-3.99148	-1.34315	0.0003
H	-3.22289	1.04544	0.00026
H	0.12474	-2.66268	-0.00066
H	-2.33707	-3.17535	-0.00015
Br	1.57797	0.06966	0.00005
O	0.73007	1.89152	-0.00025
C	-0.59322	1.89656	-0.00007
C	-1.01634	2.67844	1.2574
C	-1.01649	2.67857	-1.2574
F	-0.20504	3.74543	1.41806
F	-2.29133	3.09959	1.11763
F	-0.92357	1.87572	2.33885
F	-2.3137	3.03643	-1.14931
F	-0.25284	3.78535	-1.37727
F	-0.85398	1.89946	-2.34785

Sum of electronic and zero-point Energies=-3593.722601
Sum of electronic and thermal Energies= -3593.708868
Sum of electronic and thermal Enthalpies= -3593.707924
Sum of electronic and thermal Free Energies= -3593.764756

2-BrIII-SCF3

C	2.78872	2.53347	-0.0787
C	3.18535	1.22372	-0.31382
C	2.27986	0.18067	-0.13941
C	1.0132	0.52339	0.25857
C	0.55417	1.79211	0.51366
C	1.48729	2.81374	0.3311
H	3.49368	3.34408	-0.21405
H	4.18916	0.96587	-0.63234
H	-0.45947	1.99472	0.82838
H	1.17977	3.83591	0.514
O	1.66601	-2.0776	-0.15099
F	-3.93551	0.92128	-0.45801
F	-2.96359	-0.85487	-1.19914
F	-1.93008	1.02411	-1.25123
C	-2.77223	0.27822	-0.52401
S	-2.16147	-0.00054	1.15757
Br	-0.14178	-1.121	0.46359
C	2.65512	-1.26809	-0.37758
C	3.78298	-1.73615	0.56074
C	3.06597	-1.52096	-1.84007
F	3.81223	-3.0855	0.59103
F	4.96674	-1.27042	0.10874
F	3.55867	-1.26286	1.805
F	4.29861	-1.01421	-2.05525
F	3.0771	-2.84921	-2.08113
F	2.18237	-0.92112	-2.66586

Sum of electronic and zero-point Energies=-4329.556036
Sum of electronic and thermal Energies= -4329.535984
Sum of electronic and thermal Enthalpies= -4329.535039
Sum of electronic and thermal Free Energies= -4329.607714

2-ClIII-Br

C	-2.93446	-1.10859	0.00015
C	-2.52243	0.21846	0.00015
C	-1.16116	0.50593	0.
C	-0.29395	-0.55644	-0.00016
C	-0.62935	-1.88835	-0.00026
C	-2.00137	-2.14559	-0.00009
H	-3.99148	-1.34315	0.0003
H	-3.22289	1.04544	0.00026
H	0.12474	-2.66268	-0.00066
H	-2.33707	-3.17535	-0.00015
O	0.73007	1.89152	-0.00025
C	-0.59322	1.89656	-0.00007
C	-1.01634	2.67844	1.2574
C	-1.01649	2.67857	-1.2574
F	-0.20504	3.74543	1.41806
F	-2.29133	3.09959	1.11763
F	-0.92357	1.87572	2.33885
F	-2.3137	3.03643	-1.14931
F	-0.25284	3.78535	-1.37727
F	-0.85398	1.89946	-2.34785
Br	2.14787	-1.78352	0.00019
Cl	1.57797	0.06966	0.00005

Sum of electronic and zero-point Energies=-4053.885833
Sum of electronic and thermal Energies= -4053.869949
Sum of electronic and thermal Enthalpies= -4053.869005
Sum of electronic and thermal Free Energies= -4053.931314

2-ClIII-CCH

C	-2.04035	2.84698	0.05489
C	-2.5659	1.56311	0.11017
C	-1.72726	0.46123	0.01266
C	-0.37785	0.7091	-0.1241
C	0.19157	1.95846	-0.17863
C	-0.67465	3.04314	-0.09188
H	-2.70165	3.69963	0.12404
H	-3.63161	1.41448	0.22532
H	1.25822	2.08618	-0.28069
H	-0.26946	4.04403	-0.14095
O	-1.11851	-1.7315	-0.54841
C	-2.14837	-0.99464	0.08134
C	-3.42086	-1.26546	-0.71001
C	-2.3016	-1.41942	1.54222
C	3.50893	0.60328	-0.42291
H	4.44663	1.11586	-0.47663
C	2.45626	0.02786	-0.36262

F -1.14893 -1.22495 2.21753
F -2.61859 -2.73064 1.59436
F -3.27338 -0.7047 2.14829
F -3.66447 -2.5933 -0.70865
F -4.47777 -0.6376 -0.15212
F -3.27566 -0.84813 -1.98565
Cl 0.68903 -1.09642 -0.23301
Sum of electronic and zero-point Energies=-1556.382531
Sum of electronic and thermal Energies= -1556.365919
Sum of electronic and thermal Enthalpies= -1556.364974
Sum of electronic and thermal Free Energies= -1556.428517

2-ClIII-CF3
C -2.69011 2.28973 0.00005
C -2.95457 0.92757 0.0001
C -1.91235 -0.00045 0.00001
C -0.64991 0.52412 -0.00006
C -0.30538 1.85846 -0.00013
C -1.37667 2.75141 -0.00009
H -3.50769 2.99983 0.00013
H -3.96181 0.52596 0.00018
H 0.70804 2.22727 -0.00024
H -1.16527 3.81354 -0.00016
O -1.13277 -2.20926 -0.00037
C 2.40402 0.26982 0.00001
F 3.4394 -0.54682 0.00069
F 2.44547 1.03228 1.07774
F 2.44614 1.03133 -1.07837
C -2.19866 -1.51874 -0.00005
C -2.95724 -1.98293 -1.2573
C -2.95672 -1.98296 1.25751
F -4.21346 -1.49053 1.2328
F -3.0024 -3.33194 1.28307
F -2.31882 -1.53335 2.35907
F -2.98561 -3.33215 -1.29324
F -4.2201 -1.50705 -1.22224
F -2.33101 -1.5165 -2.35856
Cl 0.77117 -0.94889 0.00007
Sum of electronic and zero-point Energies=-1817.334878
Sum of electronic and thermal Energies= -1817.316858
Sum of electronic and thermal Enthalpies= -1817.315914
Sum of electronic and thermal Free Energies= -1817.382371

2-ClIII-CH3
C -2.69011 2.28973 0.00005
C -2.95457 0.92757 0.0001
C -1.91235 -0.00045 0.00001
C -0.64991 0.52412 -0.00006
C -0.30538 1.85846 -0.00013
C -1.37667 2.75141 -0.00009
H -3.50769 2.99983 0.00013
H -3.96181 0.52596 0.00018
H 0.70804 2.22727 -0.00024
H -1.16527 3.81354 -0.00016
O -1.13277 -2.20926 -0.00037
C 2.40402 0.26982 0.00001
C -2.19866 -1.51874 -0.00005
C -2.95724 -1.98293 -1.2573
C -2.95672 -1.98296 1.25751
F -4.21346 -1.49053 1.2328
F -3.0024 -3.33194 1.28307
F -2.31882 -1.53335 2.35907
F -2.98561 -3.33215 -1.29324
F -4.2201 -1.50705 -1.22224
F -2.33101 -1.5165 -2.35856
H 2.4376 0.8875 0.87308
H 3.24415 -0.39281 0.00056
H 2.43814 0.88672 -0.87359
Cl 0.77117 -0.94889 0.00007
Sum of electronic and zero-point Energies=-1519.549085
Sum of electronic and thermal Energies= -1519.532637
Sum of electronic and thermal Enthalpies= -1519.531692
Sum of electronic and thermal Free Energies= -1519.593817

2-ClIII-CHCH2
C -1.47784 2.86145 0.03034
C -2.0225 1.58679 0.05887
C -1.20342 0.46278 -0.00179
C 0.14941 0.69073 -0.073
C 0.74099 1.93408 -0.09943
C -0.10271 3.03613 -0.05279
H -2.12833 3.72443 0.0711
H -3.09468 1.45695 0.12705
H 1.81297 2.04725 -0.15525
H 0.32231 4.02966 -0.08167
O -0.63703 -1.78431 -0.38264
C -1.69058 -0.9845 0.03468
C -2.86618 -1.18298 -0.92188
C -2.10141 -1.32814 1.47242
C 2.87922 -0.03555 0.10832
H 3.18885 0.74836 -0.55087
C 4.3036 -0.48069 0.241
H 4.39568 -1.13578 1.082
H 4.93235 0.37326 0.38355
F -2.50521 -0.83077 -2.17413
F -3.94885 -0.46642 -0.55192
F -3.19754 -2.49169 -0.92134
F -2.49782 -2.61795 1.51445
F -1.04869 -1.1767 2.30389
F -3.10934 -0.5416 1.90593
Cl 1.18041 -0.97072 -0.11678
Sum of electronic and zero-point Energies=-1557.621702
Sum of electronic and thermal Energies= -1557.604751
Sum of electronic and thermal Enthalpies= -1557.603807
Sum of electronic and thermal Free Energies= -1557.667175

2-ClIII-Cl
C 2.52236 2.11339 -0.00003
C 2.66085 0.73134 -0.00002
C 1.52519 -0.07221 -0.00001
C 0.30752 0.56194 0.00001
C 0.09823 1.91869 0.00008
C 1.25733 2.69731 0.00004
H 3.40253 2.74383 -0.00006
H 3.62846 0.24255 -0.00002
H -0.89074 2.35437 0.00019
H 1.15606 3.77559 0.00007
O 0.38663 -2.12239 -0.00002
Cl -2.94445 0.85661 -0.00013
C 1.58381 -1.57491 -0.00003
C 2.2915 -2.1132 1.25736
C 2.29144 -2.11318 -1.25745
F 1.98976 -3.41904 1.41929
F 3.62689 -1.97401 1.11649
F 1.87822 -1.41841 2.33854
F 3.62112 -1.9067 -1.14867
F 2.04985 -3.43587 -1.37833
F 1.82369 -1.46882 -2.34765
Cl -0.99381 -0.62303 0.00007
Sum of electronic and zero-point Energies=-1939.883319
Sum of electronic and thermal Energies= -1939.867725
Sum of electronic and thermal Enthalpies= -1939.866781
Sum of electronic and thermal Free Energies= -1939.927705

2-ClIII-CN
C 2.38355 2.16507 -0.00011
C 2.54579 0.78572 -0.00018
C 1.42977 -0.04976 -0.00014
C 0.21531 0.57735 0.00019
C -0.03278 1.92761 0.00009
C 1.11008 2.72995 0.00012
H 3.25324 2.81011 -0.00022
H 3.52047 0.31084 -0.00021
H -1.02602 2.35531 0.00032
H 0.98659 3.8058 0.00018
O 0.38892 -2.14584 0.00036
C -2.68934 0.59238 0.00006
N -3.53721 1.37008 0.00019
C 1.54327 -1.57372 0.00002
C 2.25451 -2.10752 1.25731
C 2.25411 -2.10754 -1.2575
F 3.55951 -1.76435 1.21596
F 2.14397 -3.4523 1.30008
F 1.68353 -1.57383 2.35807
F 3.55398 -1.74425 -1.22801
F 2.1638 -3.45417 -1.28798
F 1.6677 -1.59215 -2.35886
Cl -1.44146 -0.64875 -0.00014
Sum of electronic and zero-point Energies=-1572.488010
Sum of electronic and thermal Energies= -1572.471635
Sum of electronic and thermal Enthalpies= -1572.470690
Sum of electronic and thermal Free Energies= -1572.533761

2-ClIII-F
C -2.93446 -1.10859 0.00015
C -2.52243 0.21846 0.00015
C -1.16116 0.50593 0.
C -0.29395 -0.55644 -0.00016
C -0.62935 -1.88835 -0.00026
C -2.00137 -2.14559 -0.00009
H -3.99148 -1.34315 0.0003
H -3.22289 1.04544 0.00026
H 0.12474 -2.66268 -0.00066
H -2.33707 -3.17535 -0.00015
O 0.73007 1.89152 -0.00025
F 2.14787 -1.78352 0.00019
C -0.59322 1.89656 -0.00007
C -1.01634 2.67844 1.2574
C -1.01649 2.67857 -1.2574
F -0.20504 3.74543 1.41806
F -2.29133 3.09959 1.11763
F -0.92357 1.87572 2.33885
F -2.3137 3.03643 -1.14931
F -0.25284 3.78535 -1.37727
F -0.85398 1.89946 -2.34785
Cl 1.37517 0.00183 0.00002

Sum of electronic and zero-point Energies=-1579.518020
Sum of electronic and thermal Energies= -1579.502989
Sum of electronic and thermal Enthalpies= -1579.502045
Sum of electronic and thermal Free Energies= -1579.562318

2-ClIII-N3

C	0.93564	2.87637	0.20469
C	1.6766	1.71122	0.35449
C	1.10354	0.48608	0.0501
C	-0.20434	0.48266	-0.37768
C	-0.97891	1.60723	-0.5439
C	-0.37582	2.82461	-0.24631
H	1.38642	3.83168	0.43531
H	2.70141	1.75362	0.69952
H	-1.99597	1.54158	-0.89582
H	-0.94404	3.73535	-0.37218
O	0.97054	-1.68882	-0.73062
C	1.74098	-0.88482	0.13829
C	1.66932	-1.40652	1.57397
C	3.16901	-0.89411	-0.38741
N	-3.35043	-0.33499	1.5211
N	-3.03357	-0.50327	0.45013
N	-2.70518	-0.66932	-0.70709
Cl	-0.77753	-1.19318	-0.68837
F	3.62799	-2.16365	-0.3975
F	3.20727	-0.40445	-1.64489
F	3.97513	-0.1552	0.40423
F	2.37921	-0.61506	2.40592
F	2.17259	-2.65841	1.61858
F	0.39066	-1.43808	2.00587

Sum of electronic and zero-point Energies=-1643.845977
Sum of electronic and thermal Energies= -1643.828805
Sum of electronic and thermal Enthalpies= -1643.827861
Sum of electronic and thermal Free Energies= -1643.893230

2-ClIII-NH2

C	-2.04035	2.84698	0.05489
C	-2.5659	1.56311	0.11017
C	-1.72726	0.46123	0.01266
C	-0.37785	0.7091	-0.1241
C	0.19157	1.95846	-0.17863
C	-0.67465	3.04314	-0.09188
H	-2.70165	3.69963	0.12404
H	-3.63161	1.41448	0.22532
H	1.25822	2.08618	-0.28069
H	-0.26946	4.04403	-0.14095
O	-1.11851	-1.7315	-0.54841
C	-2.14837	-0.99464	0.08134
C	-3.42086	-1.26546	-0.71001
C	-2.3016	-1.41942	1.54222
F	-1.14893	-1.22495	2.21753
F	-2.61859	-2.73064	1.59436
F	-3.27338	-0.7047	2.14829
F	-3.66447	-2.5933	-0.70865
F	-4.47777	-0.6376	-0.15212
F	-3.27566	-0.84813	-1.98565
N	2.30521	0.27775	0.07282
H	2.63855	0.74915	0.88932
H	2.63855	0.74915	-0.74368
Cl	0.68903	-1.09642	-0.23301

Sum of electronic and zero-point Energies=-1535.594215
Sum of electronic and thermal Energies= -1535.578305
Sum of electronic and thermal Enthalpies= -1535.577361
Sum of electronic and thermal Free Energies= -1535.639074

2-ClIII-NHAc

C	-0.85473	3.41472	0.00147
C	-1.53185	2.20509	0.00111
C	-0.81706	1.01329	0.00004
C	0.56125	1.0802	-0.00047
C	1.26756	2.26562	-0.00011
C	0.53151	3.44327	0.00083
H	-1.41506	4.33894	0.00226
H	-2.61108	2.18285	0.00168
H	2.34335	2.27341	-0.0005
H	1.05628	4.38841	0.00108
O	-0.43038	-1.3286	-0.00333
C	4.12689	-0.63449	-0.00053
O	3.94053	-1.82591	-0.00192
C	5.48582	0.01505	0.00106
H	5.58621	0.65234	-0.87609
H	5.5874	0.6443	0.88391
H	6.2516	-0.75352	-0.00275
C	-1.42375	-0.37925	-0.00049
C	-2.26756	-0.58778	-1.28126
C	-2.26336	-0.59025	1.28264
F	-2.74982	-1.81897	-1.35607
F	-3.29743	0.26172	-1.35504
F	-1.49524	-0.38027	-2.34723
F	-3.29196	0.26023	1.36229
F	-2.74673	-1.82109	1.35599
F	-1.48702	-0.38636	2.34643
N	3.13388	0.26183	0.00013

H	3.43394	1.09138	0.47109
Cl	1.46662	-0.81461	-0.00157

Sum of electronic and zero-point Energies=-1688.212700
Sum of electronic and thermal Energies= -1688.193311
Sum of electronic and thermal Enthalpies= -1688.192367
Sum of electronic and thermal Free Energies= -1688.262386

2-ClIII-OCF3

C	2.67388	2.50582	-0.08055
C	3.07538	1.1861	-0.2436
C	2.13864	0.16777	-0.09614
C	0.85091	0.5348	0.20224
C	0.38735	1.81389	0.38416
C	1.35008	2.81324	0.23105
H	3.39497	3.30541	-0.19349
H	4.09694	0.91345	-0.48175
H	-0.64249	2.02395	0.63
H	1.04878	3.8455	0.35987
O	1.40068	-2.06043	-0.03196
F	-3.25665	-0.89735	-0.63674
F	-2.19308	0.86999	-1.26105
F	-3.78459	1.02948	0.19587
C	-2.75499	0.26941	-0.18023
O	-1.90316	0.11538	0.81056
C	2.46298	-1.28967	-0.23514
C	3.51068	-1.74135	0.79923
C	2.97123	-1.60853	-1.65345
F	3.49516	-3.08772	0.897
F	4.73598	-1.33279	0.40652
F	3.21935	-1.19951	2.0009
F	4.23441	-1.15277	-1.79186
F	2.95074	-2.94412	-1.84914
F	2.17408	-1.00823	-2.56267
Cl	-0.16269	-0.89665	0.34766

Sum of electronic and zero-point Energies=-1892.569630
Sum of electronic and thermal Energies= -1892.550814
Sum of electronic and thermal Enthalpies= -1892.549869
Sum of electronic and thermal Free Energies= -1892.618925

2-ClIII-OCH3

C	2.67388	2.50582	-0.08055
C	3.07538	1.1861	-0.2436
C	2.13864	0.16777	-0.09614
C	0.85091	0.5348	0.20224
C	0.38735	1.81389	0.38416
C	1.35008	2.81324	0.23105
H	3.39497	3.30541	-0.19349
H	4.09694	0.91345	-0.48175
H	-0.64249	2.02395	0.63
H	1.04878	3.8455	0.35987
O	1.40068	-2.06043	-0.03196
C	-2.75499	0.26941	-0.18023
O	-1.90316	0.11538	0.81056
C	2.46298	-1.28967	-0.23514
C	3.51068	-1.74135	0.79923
C	2.97123	-1.60853	-1.65345
F	3.49516	-3.08772	0.897
F	4.73598	-1.33279	0.40652
F	3.21935	-1.19951	2.0009
F	4.23441	-1.15277	-1.79186
F	2.95074	-2.94412	-1.84914
F	2.17408	-1.00823	-2.56267
H	-2.3123	0.74256	-1.03173
H	-3.5809	0.87912	0.12146
H	-3.15272	-0.65563	-0.54216
Cl	-0.16269	-0.89665	0.34766

Sum of electronic and zero-point Energies=-1594.744783
Sum of electronic and thermal Energies= -1594.727825
Sum of electronic and thermal Enthalpies= -1594.726881
Sum of electronic and thermal Free Energies= -1594.790264

2-ClIII-OCOCF3

C	3.22289	2.62188	0.00859
C	3.68898	1.31361	0.00651
C	2.77394	0.26568	0.00105
C	1.43871	0.58809	-0.00237
C	0.91134	1.85792	-0.00002
C	1.85448	2.88734	0.0057
H	3.92673	3.44448	0.01292
H	4.74543	1.07154	0.00947
H	-0.15229	2.04153	-0.00164
H	1.50103	3.91113	0.00798
O	2.11919	-1.99141	-0.00185
C	-2.41859	-0.53165	0.0117
O	-2.5234	-1.72768	0.04512
O	-1.32746	0.16971	-0.02174
C	-3.65945	0.39701	0.00003
F	-4.78188	-0.30037	0.05516
F	-3.67375	1.13597	-1.11316
F	-3.62623	1.23022	1.0443
C	3.17219	-1.17944	0.00108
C	3.98348	-1.53747	1.26013
C	3.99077	-1.53249	-1.25466

F	3.99153	-2.87742	1.42432
F	5.25073	-1.09363	1.12019
F	3.41937	-0.95521	2.33961
F	5.23614	-1.02474	-1.13746
F	4.06216	-2.87451	-1.38267
F	3.39159	-1.00772	-2.34466
Cl	0.47279	-0.88316	-0.00796

Sum of electronic and zero-point Energies=-2005.889617
Sum of electronic and thermal Energies= -2005.868546
Sum of electronic and thermal Enthalpies= -2005.867602
Sum of electronic and thermal Free Energies= -2005.944396

2-ClIII-OCOCH3

C	-1.19427	2.71033	0.00044
C	-1.59611	1.37371	0.00063
C	-0.64538	0.37548	-0.0006
C	0.69489	0.71632	-0.00175
C	1.12258	2.02427	-0.00201
C	0.16076	3.02786	-0.00097
H	-2.65097	1.14357	0.00174
H	2.16894	2.27021	-0.00293
H	0.49138	4.05488	-0.00123
O	0.28526	-1.77637	-0.00507
C	4.37703	-0.29803	-0.00098
O	4.38681	-1.50148	-0.00341
O	3.26291	0.43189	0.00007
C	5.61253	0.56548	0.0015
H	5.61031	1.20972	-0.87634
H	5.6112	1.20286	0.88435
H	6.4919	-0.0703	-0.00131
C	-0.91853	-1.11207	-0.00085
C	-1.68712	-1.51584	-1.28145
C	-1.67986	-1.51718	1.28367
F	-1.85769	-2.82722	-1.35773
F	-2.89076	-0.93898	-1.34941
F	-0.99016	-1.12597	-2.34739
F	-2.88191	-0.93819	1.36046
F	-1.85237	-2.82841	1.35838
F	-0.97536	-1.13074	2.34595
Cl	1.77497	-0.70281	-0.00252
H	-1.98372	3.4326	0.00141

Sum of electronic and zero-point Energies=-1708.100153
Sum of electronic and thermal Energies= -1708.081877
Sum of electronic and thermal Enthalpies= -1708.080932
Sum of electronic and thermal Free Energies= -1708.147801

2-ClIII-OCOPh

C	-0.85473	3.41472	0.00147
C	-1.53185	2.20509	0.00111
C	-0.81706	1.01329	0.00004
C	0.56125	1.0802	-0.00047
C	1.26756	2.26562	-0.00011
C	0.53151	3.44327	0.00083
H	-1.41506	4.33894	0.00226
H	-2.61108	2.18285	0.00168
H	2.34335	2.27341	-0.0005
H	1.05628	4.38841	0.00108
O	-0.43038	-1.3286	-0.00333
C	4.12689	-0.63449	-0.00053
O	3.94053	-1.8259	-0.00384
O	3.14066	0.25571	0.0025
C	-1.42375	-0.37925	-0.00049
C	-2.26756	-0.58778	-1.28126
C	-2.26336	-0.59025	1.28264
F	-2.74982	-1.81897	-1.35607
F	-3.29743	0.26172	-1.35504
F	-1.49524	-0.38027	-2.34723
F	-3.29196	0.26023	1.36229
F	-2.74673	-1.82109	1.35599
F	-1.48702	-0.38636	2.34643
C	5.51633	0.02963	0.0011
C	6.66672	-0.7597	-0.00168
C	5.62473	1.42024	0.00523
C	7.9252	-0.15849	0.00037
H	6.58092	-1.856	-0.00416
C	6.88351	2.0218	0.00628
H	4.71814	2.04251	0.00724
C	8.03368	1.23271	0.00399
H	8.832	-0.78058	-0.00119
H	6.96867	3.11825	0.00913
H	9.02604	1.70653	0.00554
Cl	1.32003	-0.50783	-0.00139

Sum of electronic and zero-point Energies=-1899.777889
Sum of electronic and thermal Energies= -1899.755940
Sum of electronic and thermal Enthalpies= -1899.754996
Sum of electronic and thermal Free Energies= -1899.832237

2-ClIII-OH

C	2.85263	-1.32247	0.00438
C	2.53897	0.03132	0.02066
C	1.20519	0.42867	0.01343
C	0.26118	-0.56546	0.00447
C	0.49803	-1.91871	-0.02637
C	1.8446	-2.28523	-0.02237
H	3.88939	-1.63508	0.00637
H	3.29795	0.80511	0.03341
H	-0.31078	-2.63443	-0.06185
H	2.09873	-3.33788	-0.04592
O	-0.53232	1.99106	-0.02484
O	-2.35941	-1.6203	-0.02187
H	-2.69235	-1.77691	0.86934
C	0.76361	1.8772	0.00979
C	1.26116	2.64337	-1.22998
C	1.22455	2.60844	1.28432
F	0.54765	3.7805	-1.37257
F	2.56736	2.94797	-1.0765
F	1.10463	1.87328	-2.32769
F	2.54865	2.85941	1.20518
F	0.55211	3.773	1.40309
F	0.97696	1.83391	2.36196
Cl	-1.3738	0.08565	-0.01763

Sum of electronic and zero-point Energies=-1555.477484
Sum of electronic and thermal Energies= -1555.461986
Sum of electronic and thermal Enthalpies= -1555.461042
Sum of electronic and thermal Free Energies= -1555.521011

2-ClIII-OTf

C	-2.93446	-1.10859	0.00015
C	-2.52243	0.21846	0.00015
C	-1.16116	0.50593	0.
C	-0.29395	-0.55644	-0.00016
C	-0.62935	-1.88835	-0.00026
C	-2.00137	-2.14559	-0.00009
H	-3.99148	-1.34315	0.0003
H	-3.22289	1.04544	0.00026
H	0.12474	-2.66268	-0.00066
H	-2.33707	-3.17535	-0.00015
O	0.73007	1.89152	-0.00025
C	-0.59322	1.89656	-0.00007
C	-1.01634	2.67844	1.2574
C	-1.01649	2.67857	-1.2574
F	-0.20504	3.74543	1.41806
F	-2.29133	3.09959	1.11763
F	-0.92357	1.87572	2.33885
F	-2.3137	3.03643	-1.14931
F	-0.25284	3.78535	-1.37727
F	-0.85398	1.89946	-2.34785
O	2.10706	-1.65082	0.00018
S	3.58271	-1.98493	0.6855
O	3.30434	-2.89027	1.68795
O	4.03949	-0.7085	0.975
C	4.39195	-2.66525	-0.54171
F	4.69886	-1.72121	-1.45663
F	5.52792	-3.2263	-0.07556
F	3.62352	-3.61526	-1.11573
Cl	1.57797	0.06966	0.00005

Sum of electronic and zero-point Energies=-2441.171890
Sum of electronic and thermal Energies= -2441.149686
Sum of electronic and thermal Enthalpies= -2441.148741
Sum of electronic and thermal Free Energies= -2441.226669

2-ClIII-OTs

C	-2.93446	-1.10859	0.00015
C	-2.52243	0.21846	0.00015
C	-1.16116	0.50593	0.
C	-0.29395	-0.55644	-0.00016
C	-0.62935	-1.88835	-0.00026
C	-2.00137	-2.14559	-0.00009
H	-3.99148	-1.34315	0.0003
H	-3.22289	1.04544	0.00026
H	0.12474	-2.66268	-0.00066
H	-2.33707	-3.17535	-0.00015
O	0.73007	1.89152	-0.00025
C	-0.59322	1.89656	-0.00007
C	-1.01634	2.67844	1.2574
C	-1.01649	2.67857	-1.2574
F	-0.20504	3.74543	1.41806
F	-2.29133	3.09959	1.11763
F	-0.92357	1.87572	2.33885
F	-2.3137	3.03643	-1.14931
F	-0.25284	3.78535	-1.37727
F	-0.85398	1.89946	-2.34785
O	2.10706	-1.65082	0.00018
S	3.58271	-1.98493	0.6855
O	3.30434	-2.89027	1.68795
O	4.03949	-0.7085	0.975
C	4.40699	-2.6779	-0.56451
C	4.30243	-4.05379	-0.81874
C	5.23548	-1.91115	-1.39822
C	4.99636	-4.64458	-1.868
H	3.66155	-4.67301	-0.16481
C	5.9336	-2.49374	-2.4484
H	5.33672	-0.82766	-1.20443
C	5.81792	-3.86856	-2.69624
H	4.90167	-5.72602	-2.04962
H	6.58094	-1.87518	-3.08849

C 6.54432 -4.49115 -3.82562
H 7.47449 -3.92111 -4.06961
H 6.82578 -5.5467 -3.58761
H 5.8897 -4.49974 -4.7345
Cl 1.57797 0.06966 0.00005
Sum of electronic and zero-point Energies=-2374.347379
Sum of electronic and thermal Energies= -2374.323178
Sum of electronic and thermal Enthalpies= -2374.322234
Sum of electronic and thermal Free Energies= -2374.404817

2-ClIII-radical
C -2.93446 -1.10859 0.00015
C -2.52243 0.21846 0.00015
C -1.16116 0.50593 0.
C -0.29395 -0.55644 -0.00016
C -0.62935 -1.88835 -0.00026
C -2.00137 -2.14559 -0.00009
H -3.99148 -1.34315 0.0003
H -3.22289 1.04544 0.00026
H 0.12474 -2.66268 -0.00066
H -2.33707 -3.17535 -0.00015
O 0.73007 1.89152 -0.00025
C -0.59322 1.89656 -0.00007
C -1.01634 2.67844 1.2574
C -1.01649 2.67857 -1.2574
F -0.20504 3.74543 1.41806
F -2.29133 3.09959 1.11763
F -0.92357 1.87572 2.33885
F -2.3137 3.03643 -1.14931
F -0.25284 3.78535 -1.37727
F -0.85398 1.89946 -2.34785
Cl 1.37517 0.00183 0.00002
Sum of electronic and zero-point Energies=-1479.729405
Sum of electronic and thermal Energies= -1479.715112
Sum of electronic and thermal Enthalpies= -1479.714168
Sum of electronic and thermal Free Energies= -1479.772036

2-ClIII-SCF3
C 2.78872 2.53347 -0.0787
C 3.18535 1.22372 -0.31382
C 2.27986 0.18067 -0.13941
C 1.0132 0.52339 0.25857
C 0.55417 1.79211 0.51366
C 1.48729 2.81374 0.3311
H 3.49368 3.34408 -0.21405
H 4.18916 0.96587 -0.63234
H -0.45947 1.99472 0.82838
H 1.17977 3.83591 0.514
O 1.66601 -2.0776 -0.15099
F -3.93551 0.92128 -0.45801
F -2.96359 -0.85487 -1.19914
F -1.93008 1.02411 -1.25123
C -2.77223 0.27822 -0.52401
S -2.16147 -0.00054 1.15757
C 2.65512 -1.26809 -0.37758
C 3.78298 -1.73615 0.56074
C 3.06597 -1.52096 -1.84007
F 3.81223 -3.0855 0.59103
F 4.96674 -1.27042 0.10874
F 3.55867 -1.26286 1.805
F 4.29861 -1.01421 -2.05525
F 3.0771 -2.84921 -2.08113
F 2.18237 -0.92112 -2.66586
Cl 0.00684 -0.90941 0.43721
Sum of electronic and zero-point Energies=-2215.531158
Sum of electronic and thermal Energies= -2215.511404
Sum of electronic and thermal Enthalpies= -2215.510460
Sum of electronic and thermal Free Energies= -2215.582134

2-IIII-Br
C -2.93446 -1.10859 0.00015
C -2.52243 0.21846 0.00015
C -1.16116 0.50593 0.
C -0.29395 -0.55644 -0.00016
C -0.62935 -1.88835 -0.00026
C -2.00137 -2.14559 -0.00009
H -3.99148 -1.34315 0.0003
H -3.22289 1.04544 0.00026
H 0.12474 -2.66268 -0.00066
H -2.33707 -3.17535 -0.00015
O 0.73007 1.89152 -0.00025
C -0.59322 1.89656 -0.00007
C -1.01634 2.67844 1.2574
C -1.01649 2.67857 -1.2574
F -0.20504 3.74543 1.41806
F -2.29133 3.09959 1.11763
F -0.92357 1.87572 2.33885
F -2.3137 3.03643 -1.14931
F -0.25284 3.78535 -1.37727
F -0.85398 1.89946 -2.34785
Br 2.14787 -1.78352 0.00019
I 1.57797 0.06966 0.00005
Sum of electronic and zero-point Energies=-3891.390438
Sum of electronic and thermal Energies= -3891.374043
Sum of electronic and thermal Enthalpies= -3891.373099
Sum of electronic and thermal Free Energies= -3891.438124

2-IIII-CCH
C -2.04035 2.84698 0.05489
C -2.5659 1.56311 0.11017
C -1.72726 0.46123 0.01266
C -0.37785 0.7091 -0.1241
C 0.19157 1.95846 -0.17863
C -0.67465 3.04314 -0.09188
H -2.70165 3.69963 0.12404
H -3.63161 1.41448 0.22532
H 1.25822 2.08618 -0.28069
H -0.26946 4.04403 -0.14095
O -1.11851 -1.7315 -0.54841
C -2.14837 -0.99464 0.08134
C -3.42086 -1.26546 -0.71001
C -2.3016 -1.41942 1.54222
C 3.50893 0.60328 -0.42291
H 4.44663 1.11586 -0.47663
C 2.45626 0.02786 -0.36262
F -1.14893 -1.22495 2.21753
F -2.61859 -2.73064 1.59436
F -3.27338 -0.7047 2.14829
F -3.66447 -2.5933 -0.70865
F -4.47777 -0.6376 -0.15212
F -3.27566 -0.84813 -1.98565
I 0.68903 -1.09642 -0.23301
Sum of electronic and zero-point Energies=-1393.874142
Sum of electronic and thermal Energies= -1393.857007
Sum of electronic and thermal Enthalpies= -1393.856063
Sum of electronic and thermal Free Energies= -1393.921040

2-IIII-CF3
C -2.69011 2.28973 0.00005
C -2.95457 0.92757 0.0001
C -1.91235 -0.00045 0.00001
C -0.64991 0.52412 -0.00006
C -0.30538 1.85846 -0.00013
C -1.37667 2.75141 -0.00009
H -3.50769 2.99983 0.00013
H -3.96181 0.52596 0.00018
H 0.70804 2.22727 -0.00024
H -1.16527 3.81354 -0.00016
O -1.13277 -2.20926 -0.00037
C 2.40402 0.26982 0.00001
F 3.4394 -0.54682 0.00069
F 2.44547 1.03228 1.07774
F 2.44614 1.03133 -1.07837
C -2.19866 -1.51874 -0.00005
C -2.95724 -1.98293 -1.2573
C -2.95672 -1.98296 1.25751
F -4.21346 -1.49053 1.2328
F -3.0024 -3.33194 1.28307
F -2.31882 -1.53335 2.35907
F -2.98561 -3.33215 -1.29324
F -4.2201 -1.50705 -1.22224
F -2.33101 -1.5165 -2.35856
I 0.77117 -0.94889 0.00007
Sum of electronic and zero-point Energies=-1654.814543
Sum of electronic and thermal Energies= -1654.795785
Sum of electronic and thermal Enthalpies= -1654.794841
Sum of electronic and thermal Free Energies= -1654.864249

2-IIII-CH3
C -2.69011 2.28973 0.00005
C -2.95457 0.92757 0.0001
C -1.91235 -0.00045 0.00001
C -0.64991 0.52412 -0.00006
C -0.30538 1.85846 -0.00013
C -1.37667 2.75141 -0.00009
H -3.50769 2.99983 0.00013
H -3.96181 0.52596 0.00018
H 0.70804 2.22727 -0.00024
H -1.16527 3.81354 -0.00016
O -1.13277 -2.20926 -0.00037
C 2.40402 0.26982 0.00001
C -2.19866 -1.51874 -0.00005
C -2.95724 -1.98293 -1.2573
C -2.95672 -1.98296 1.25751
F -4.21346 -1.49053 1.2328
F -3.0024 -3.33194 1.28307
F -2.31882 -1.53335 2.35907
F -2.98561 -3.33215 -1.29324
F -4.2201 -1.50705 -1.22224
F -2.33101 -1.5165 -2.35856
H 2.4376 0.8875 0.87308
H 3.24415 -0.39281 0.00056
H 2.43814 0.88672 -0.87359
I 0.77117 -0.94889 0.00007
Sum of electronic and zero-point Energies=-1357.025991
Sum of electronic and thermal Energies= -1357.009449

Sum of electronic and thermal Enthalpies= -1357.008505
Sum of electronic and thermal Free Energies= -1357.071880

2-IIII-CHCH2

C	-1.47784	2.86145	0.03034
C	-2.0225	1.58679	0.05887
C	-1.20342	0.46278	-0.00179
C	0.14941	0.69073	-0.073
C	0.74099	1.93408	-0.09943
C	-0.10271	3.03613	-0.05279
H	-2.12833	3.72443	0.0711
H	-3.09468	1.45695	0.12705
H	1.81297	2.04725	-0.15525
H	0.32231	4.02966	-0.08167
O	-0.63703	-1.78431	-0.38264
C	-1.69058	-0.9845	0.03468
C	-2.86618	-1.18298	-0.92188
C	-2.10141	-1.32814	1.47242
C	2.87922	-0.03555	0.10832
H	3.18885	0.74836	-0.55087
C	4.3036	-0.48069	0.241
H	4.39568	-1.13578	1.082
H	4.93235	0.37326	0.38355
I	1.18041	-0.97072	-0.11678
F	-2.50521	-0.83077	-2.17413
F	-3.94885	-0.46642	-0.55192
F	-3.19754	-2.49169	-0.92134
F	-2.49782	-2.61795	1.51445
F	-1.04869	-1.1767	2.30389
F	-3.10934	-0.5416	1.90593

Sum of electronic and zero-point Energies=-1395.095676
Sum of electronic and thermal Energies= -1395.078166
Sum of electronic and thermal Enthalpies= -1395.077222
Sum of electronic and thermal Free Energies= -1395.143181

2-IIII-Cl

C	2.52236	2.11339	-0.00003
C	2.66085	0.73134	-0.00002
C	1.52519	-0.07221	-0.00001
C	0.30752	0.56194	0.00001
C	0.09823	1.91869	0.00008
C	1.25733	2.69731	0.00004
H	3.40253	2.74383	-0.00006
H	3.62846	0.24255	-0.00002
H	-0.89074	2.35437	0.00019
H	1.15606	3.77559	0.00007
O	0.38663	-2.12239	-0.00002
Cl	-2.94445	0.85661	-0.00013
C	1.58381	-1.57491	-0.00003
C	2.2915	-2.1132	1.25736
C	2.29144	-2.11318	-1.25745
F	1.98976	-3.41904	1.41929
F	3.62689	-1.97401	1.11649
F	1.87822	-1.41841	2.33854
F	3.62112	-1.9067	-1.14867
F	2.04985	-3.43587	-1.37833
F	1.82369	-1.46882	-2.34765
I	-1.2452	-0.85194	0.00008

Sum of electronic and zero-point Energies=-1777.389565
Sum of electronic and thermal Energies= -1777.373490
Sum of electronic and thermal Enthalpies= -1777.372546
Sum of electronic and thermal Free Energies= -1777.435709

2-IIII-CN

C	2.38355	2.16507	-0.00011
C	2.54579	0.78572	-0.00018
C	1.42977	-0.04976	-0.00014
C	0.21531	0.57735	0.00019
C	-0.03278	1.92761	0.00009
C	1.11008	2.72995	0.00012
H	3.25324	2.81011	-0.00022
H	3.52047	0.31084	-0.00021
H	-1.02602	2.35531	0.00032
H	0.98659	3.8058	0.00018
O	0.38892	-2.14584	0.00036
C	-2.68934	0.59238	0.00006
N	-3.53721	1.37008	0.00019
C	1.54327	-1.57372	0.00002
C	2.25451	-2.10752	1.25731
C	2.25411	-2.10754	-1.2575
F	3.55951	-1.76435	1.21596
F	2.14397	-3.4523	1.30008
F	1.68353	-1.57383	2.35807
F	3.55398	-1.74425	-1.22801
F	2.1638	-3.45417	-1.28798
F	1.6677	-1.59215	-2.35886
I	-1.2004	-0.88851	-0.00018

Sum of electronic and zero-point Energies=-1409.986466
Sum of electronic and thermal Energies= -1409.969532
Sum of electronic and thermal Enthalpies= -1409.968588
Sum of electronic and thermal Free Energies= -1410.033454

2-IIII-F

C	-2.93446	-1.10859	0.00015
C	-2.52243	0.21846	0.00015
C	-1.16116	0.50593	0.
C	-0.29395	-0.55644	-0.00016
C	-0.62935	-1.88835	-0.00026
C	-2.00137	-2.14559	-0.00009
H	-3.99148	-1.34315	0.0003
H	-3.22289	1.04544	0.00026
H	0.12474	-2.66268	-0.00066
H	-2.33707	-3.17535	-0.00015
O	0.73007	1.89152	-0.00025
F	2.14787	-1.78352	0.00019
C	-0.59322	1.89656	-0.00007
C	-1.01634	2.67844	1.2574
C	-1.01649	2.67857	-1.2574
F	-0.20504	3.74543	1.41806
F	-2.29133	3.09959	1.11763
F	-0.92357	1.87572	2.33885
F	-2.3137	3.03643	-1.14931
F	-0.25284	3.78535	-1.37727
F	-0.85398	1.89946	-2.34785
I	1.69761	0.10968	0.00006

Sum of electronic and zero-point Energies=-1417.028064
Sum of electronic and thermal Energies= -1417.012449
Sum of electronic and thermal Enthalpies= -1417.011505
Sum of electronic and thermal Free Energies= -1417.073133

2-IIII-N3

C	-2.93446	-1.10859	0.00015
C	-2.52243	0.21846	0.00015
C	-1.16116	0.50593	0.
C	-0.29395	-0.55644	-0.00016
C	-0.62935	-1.88835	-0.00026
C	-2.00137	-2.14559	-0.00009
H	-3.99148	-1.34315	0.0003
H	-3.22289	1.04544	0.00026
H	0.12474	-2.66268	-0.00066
H	-2.33707	-3.17535	-0.00015
O	0.73007	1.89152	-0.00025
C	-0.59322	1.89656	-0.00007
C	-1.01634	2.67844	1.2574
C	-1.01649	2.67857	-1.2574
F	-0.20504	3.74543	1.41806
F	-2.29133	3.09959	1.11763
F	-0.92357	1.87572	2.33885
F	-2.3137	3.03643	-1.14931
F	-0.25284	3.78535	-1.37727
F	-0.85398	1.89946	-2.34785
N	2.11882	-1.68906	0.00018
N	3.3197	-1.96422	-0.00165
N	3.73122	-3.30237	-0.00011
I	1.57797	0.06966	0.00005

Sum of electronic and zero-point Energies=-1481.346347
Sum of electronic and thermal Energies= -1481.328612
Sum of electronic and thermal Enthalpies= -1481.327668
Sum of electronic and thermal Free Energies= -1481.395431

2-IIII-NH2

C	-2.04035	2.84698	0.05489
C	-2.5659	1.56311	0.11017
C	-1.72726	0.46123	0.01266
C	-0.37785	0.7091	-0.1241
C	0.19157	1.95846	-0.17863
C	-0.67465	3.04314	-0.09188
H	-2.70165	3.69963	0.12404
H	-3.63161	1.41448	0.22532
H	1.25822	2.08618	-0.28069
H	-0.26946	4.04403	-0.14095
O	-1.11851	-1.7315	-0.54841
C	-2.14837	-0.99464	0.08134
C	-3.42086	-1.26546	-0.71001
C	-2.3016	-1.41942	1.54222
F	-1.14893	-1.22495	2.21753
F	-2.61859	-2.73064	1.59436
F	-3.27338	-0.7047	2.14829
F	-3.66447	-2.5933	-0.70865
F	-4.47777	-0.6376	-0.15212
F	-3.27566	-0.84813	-1.98565
N	2.30521	0.27775	0.07282
H	2.63855	0.74915	0.88932
H	2.63855	0.74915	-0.74368
I	0.68903	-1.09642	-0.23301

Sum of electronic and zero-point Energies=-1373.087377
Sum of electronic and thermal Energies= -1373.071111
Sum of electronic and thermal Enthalpies= -1373.070167
Sum of electronic and thermal Free Energies= -1373.132857

2-IIII-NHAc

C	-0.85473	3.41472	0.00147
C	-1.53185	2.20509	0.00111
C	-0.81706	1.01329	0.00004
C	0.56125	1.0802	-0.00047
C	1.26756	2.26562	-0.00011

C	0.53151	3.44327	0.00083
H	-1.41506	4.33894	0.00226
H	-2.61108	2.18285	0.00168
H	2.34335	2.27341	-0.0005
H	1.05628	4.38841	0.00108
O	-0.43038	-1.3286	-0.00333
C	4.12689	-0.63449	-0.00053
O	3.94053	-1.82591	-0.00192
C	5.48582	0.01505	0.00106
H	5.58621	0.65234	-0.87609
H	5.5874	0.6443	0.88391
H	6.2516	-0.75352	-0.00275
C	-1.42375	-0.37925	-0.00049
C	-2.26756	-0.58778	-1.28126
C	-2.26336	-0.59025	1.28264
F	-2.74982	-1.81897	-1.35607
F	-3.29743	0.26172	-1.35504
F	-1.49524	-0.38027	-2.34723
F	-3.29196	0.26023	1.36229
F	-2.74673	-1.82109	1.35599
F	-1.48702	-0.38636	2.34643
N	3.13388	0.26183	0.00013
H	3.43394	1.09138	0.47109
I	1.46662	-0.81461	-0.00157

Sum of electronic and zero-point Energies=-1525.713291
Sum of electronic and thermal Energies= -1525.693196
Sum of electronic and thermal Enthalpies= -1525.692251
Sum of electronic and thermal Free Energies= -1525.765664

2-IIII-OCF3

C	2.67388	2.50582	-0.08055
C	3.07538	1.1861	-0.2436
C	2.13864	0.16777	-0.09614
C	0.85091	0.5348	0.20224
C	0.38735	1.81389	0.38416
C	1.35008	2.81324	0.23105
H	3.39497	3.30541	-0.19349
H	4.09694	0.91345	-0.48175
H	-0.64249	2.02395	0.63
H	1.04878	3.8455	0.35987
O	1.40068	-2.06043	-0.03196
F	-3.25665	-0.89735	-0.63674
F	-2.19308	0.86999	-1.26105
F	-3.78459	1.02948	0.19587
C	-2.75499	0.26941	-0.18023
O	-1.90316	0.11538	0.81056
C	2.46298	-1.28967	-0.23514
C	3.51068	-1.74135	0.79923
C	2.97123	-1.60853	-1.65345
F	3.49516	-3.08772	0.897
F	4.73598	-1.33279	0.40652
F	3.21935	-1.19951	2.0009
F	4.23441	-1.15277	-1.79186
F	2.95074	-2.94412	-1.84914
F	2.17408	-1.00823	-2.56267
I	-0.3585	-1.17318	0.37575

Sum of electronic and zero-point Energies=-1730.076934
Sum of electronic and thermal Energies= -1730.057494
Sum of electronic and thermal Enthalpies= -1730.056550
Sum of electronic and thermal Free Energies= -1730.128268

2-IIII-OCH3

C	2.67388	2.50582	-0.08055
C	3.07538	1.1861	-0.2436
C	2.13864	0.16777	-0.09614
C	0.85091	0.5348	0.20224
C	0.38735	1.81389	0.38416
C	1.35008	2.81324	0.23105
H	3.39497	3.30541	-0.19349
H	4.09694	0.91345	-0.48175
H	-0.64249	2.02395	0.63
H	1.04878	3.8455	0.35987
O	1.40068	-2.06043	-0.03196
C	-2.75499	0.26941	-0.18023
O	-1.90316	0.11538	0.81056
C	2.46298	-1.28967	-0.23514
C	3.51068	-1.74135	0.79923
C	2.97123	-1.60853	-1.65345
F	3.49516	-3.08772	0.897
F	4.73598	-1.33279	0.40652
F	3.21935	-1.19951	2.0009
F	4.23441	-1.15277	-1.79186
F	2.95074	-2.94412	-1.84914
F	2.17408	-1.00823	-2.56267
H	-2.3123	0.74256	-1.03173
H	-3.5809	0.87912	0.12146
H	-3.15272	-0.65563	-0.54216
I	-0.3585	-1.17318	0.37575

Sum of electronic and zero-point Energies=-1432.244911
Sum of electronic and thermal Energies= -1432.227266
Sum of electronic and thermal Enthalpies= -1432.226322
Sum of electronic and thermal Free Energies= -1432.292140

2-IIII-OCOCF3

C	3.22289	2.62188	0.00859
C	3.68898	1.31361	0.00651
C	2.77394	0.26568	0.00105
C	1.43871	0.58809	-0.00237
C	0.91134	1.85792	-0.00002
C	1.85448	2.88734	0.0057
H	3.92673	3.44448	0.01292
H	4.74543	1.07154	0.00947
H	-0.15229	2.04153	-0.00164
H	1.50103	3.91113	0.00798
O	2.11919	-1.99141	-0.00185
C	-2.41859	-0.53165	0.0117
O	-2.5234	-1.72768	0.04512
O	-1.32746	0.16971	-0.02174
C	-3.65945	0.39701	0.00003
F	-4.78188	-0.30037	0.05516
F	-3.67375	1.13597	-1.11316
F	-3.62623	1.23022	1.0443
C	3.17219	-1.17944	0.00108
C	3.98348	-1.53747	1.26013
C	3.99077	-1.53249	-1.25466
F	3.99153	-2.87742	1.42432
F	5.25073	-1.09363	1.12019
F	3.41937	-0.95521	2.33961
F	5.23614	-1.02474	-1.13746
F	4.06216	-2.87451	-1.38267
F	3.39159	-1.00772	-2.34466
I	0.2862	-1.16738	-0.00904

Sum of electronic and zero-point Energies=-1843.398106
Sum of electronic and thermal Energies= -1843.376484
Sum of electronic and thermal Enthalpies= -1843.375539
Sum of electronic and thermal Free Energies= -1843.453872

2-IIII-OCOCH3

C	2.94595	2.24262	0.
C	3.14905	0.86891	-0.00023
C	2.05101	0.01452	-0.00014
C	0.80079	0.58421	0.00015
C	0.53351	1.93493	0.00041
C	1.65414	2.76631	0.00033
H	3.79558	2.9138	-0.00007
H	4.13833	0.42604	-0.00045
H	-0.47532	2.31781	0.00073
H	1.50439	3.83907	0.00055
O	1.00226	-2.08015	0.
C	-3.20495	0.1778	0.00021
O	-3.42588	-1.00956	0.00076
O	-1.9799	0.68529	0.00005
C	-4.26655	1.25034	-0.00099
H	-4.14463	1.88403	0.87785
H	-4.15011	1.8754	-0.88681
H	-5.24737	0.78154	0.00398
C	2.175	-1.48618	-0.00024
C	2.90387	-1.99583	1.25698
C	2.90344	-1.99553	-1.25783
F	2.65696	-3.31334	1.41726
F	4.23236	-1.80072	1.1171
F	2.46125	-1.32019	2.3387
F	4.22347	-1.73389	-1.15024
F	2.71692	-3.32721	-1.37755
F	2.40849	-1.372	-2.34812
I	-0.68643	-0.89842	0.00013

Sum of electronic and zero-point Energies=-1545.610085
Sum of electronic and thermal Energies= -1545.590387
Sum of electronic and thermal Enthalpies= -1545.589443
Sum of electronic and thermal Free Energies= -1545.661266

2-IIII-OCOPh

C	-0.85473	3.41472	0.00147
C	-1.53185	2.20509	0.00111
C	-0.81706	1.01329	0.00004
C	0.56125	1.0802	-0.00047
C	1.26756	2.26562	-0.00011
C	0.53151	3.44327	0.00083
H	-1.41506	4.33894	0.00226
H	-2.61108	2.18285	0.00168
H	2.34335	2.27341	-0.0005
H	1.05628	4.38841	0.00108
O	-0.43038	-1.3286	-0.00333
C	4.12689	-0.63449	-0.00053
O	3.94053	-1.8259	-0.00384
O	3.14066	0.25571	0.0025
C	-1.42375	-0.37925	-0.00049
C	-2.26756	-0.58778	-1.28126
C	-2.26336	-0.59025	1.28264
F	-2.74982	-1.81897	-1.35607
F	-3.29743	0.26172	-1.35504
F	-1.49524	-0.38027	-2.34723
F	-3.29196	0.26023	1.36229
F	-2.74673	-1.82109	1.35599
F	-1.48702	-0.38636	2.34643
C	5.51633	0.02963	0.0011

C	6.66672	-0.7597	-0.00168
C	5.62473	1.42024	0.00523
C	7.9252	-0.15849	0.00037
H	6.58092	-1.856	-0.00416
C	6.88351	2.0218	0.00628
H	4.71814	2.04251	0.00724
C	8.03368	1.23271	0.00399
H	8.832	-0.78058	-0.00119
H	6.96867	3.11825	0.00913
H	9.02604	1.70653	0.00554
I	1.46662	-0.81461	-0.00157

Sum of electronic and zero-point Energies=-1737.288217
Sum of electronic and thermal Energies= -1737.265660
Sum of electronic and thermal Enthalpies= -1737.264716
Sum of electronic and thermal Free Energies= -1737.344630

2-IIII-OH

C	2.85263	-1.32247	0.00438
C	2.53897	0.03132	0.02066
C	1.20519	0.42867	0.01343
C	0.26118	-0.56546	0.00447
C	0.49803	-1.91871	-0.02637
C	1.8446	-2.28523	-0.02237
H	3.88939	-1.63508	0.00637
H	3.29795	0.80511	0.03341
H	-0.31078	-2.63443	-0.06185
H	2.09873	-3.33788	-0.04592
O	-0.53232	1.99106	-0.02484
O	-2.35941	-1.6203	-0.02187
H	-2.69235	-1.77691	0.86934
C	0.76361	1.8772	0.00979
C	1.26116	2.64337	-1.22998
C	1.22455	2.60844	1.28432
F	0.54765	3.7805	-1.37257
F	2.56736	2.94797	-1.0765
F	1.10463	1.87328	-2.32769
F	2.54865	2.85941	1.20518
F	0.55211	3.773	1.40309
F	0.97696	1.83391	2.36196
I	-1.68965	0.21143	-0.0219

Sum of electronic and zero-point Energies=-1392.981819
Sum of electronic and thermal Energies= -1392.965720
Sum of electronic and thermal Enthalpies= -1392.964776
Sum of electronic and thermal Free Energies= -1393.027147

2-IIII-OTf

C	-2.93446	-1.10859	0.00015
C	-2.52243	0.21846	0.00015
C	-1.16116	0.50593	0.
C	-0.29395	-0.55644	-0.00016
C	-0.62935	-1.88835	-0.00026
C	-2.00137	-2.14559	-0.00009
H	-3.99148	-1.34315	0.0003
H	-3.22289	1.04544	0.00026
H	0.12474	-2.66268	-0.00066
H	-2.33707	-3.17535	-0.00015
O	0.73007	1.89152	-0.00025
C	-0.59322	1.89656	-0.00007
C	-1.01634	2.67844	1.2574
C	-1.01649	2.67857	-1.2574
F	-0.20504	3.74543	1.41806
F	-2.29133	3.09959	1.11763
F	-0.92357	1.87572	2.33885
F	-2.3137	3.03643	-1.14931
F	-0.25284	3.78535	-1.37727
F	-0.85398	1.89946	-2.34785
O	2.10706	-1.65082	0.00018
S	3.58271	-1.98493	0.6855
O	3.30434	-2.89027	1.68795
O	4.03949	-0.7085	0.975
C	4.39195	-2.66525	-0.54171
F	4.69886	-1.72121	-1.45663
F	5.52792	-3.2263	-0.07556
F	3.62352	-3.61526	-1.11573
I	1.57797	0.06966	0.00005

Sum of electronic and zero-point Energies=-2278.677780
Sum of electronic and thermal Energies= -2278.654945
Sum of electronic and thermal Enthalpies= -2278.654001
Sum of electronic and thermal Free Energies= -2278.734728

2-IIII-OTs

C	-2.93446	-1.10859	0.00015
C	-2.52243	0.21846	0.00015
C	-1.16116	0.50593	0.
C	-0.29395	-0.55644	-0.00016
C	-0.62935	-1.88835	-0.00026
C	-2.00137	-2.14559	-0.00009
H	-3.99148	-1.34315	0.0003
H	-3.22289	1.04544	0.00026
H	0.12474	-2.66268	-0.00066
H	-2.33707	-3.17535	-0.00015
O	0.73007	1.89152	-0.00025
C	-0.59322	1.89656	-0.00007
C	-1.01634	2.67844	1.2574
C	-1.01649	2.67857	-1.2574
F	-0.20504	3.74543	1.41806
F	-2.29133	3.09959	1.11763
F	-0.92357	1.87572	2.33885
F	-2.3137	3.03643	-1.14931
F	-0.25284	3.78535	-1.37727
F	-0.85398	1.89946	-2.34785
O	2.10706	-1.65082	0.00018
S	3.58271	-1.98493	0.6855
O	3.30434	-2.89027	1.68795
O	4.03949	-0.7085	0.975
C	4.40699	-2.6779	-0.56451
C	4.30243	-4.05379	-0.81874
C	5.23548	-1.91115	-1.39822
C	4.99636	-4.64458	-1.868
H	3.66155	-4.67301	-0.16481
C	5.9336	-2.49374	-2.4484
H	5.33672	-0.82766	-1.20443
C	5.81792	-3.86856	-2.69624
H	4.90167	-5.72602	-2.04962
H	6.58094	-1.87518	-3.08849
C	6.54432	-4.49115	-3.82562
H	7.47449	-3.92111	-4.06961
H	6.82578	-5.5467	-3.58761
H	5.8897	-4.49974	-4.7345
I	1.57797	0.06966	0.00005

Sum of electronic and zero-point Energies=-2211.854815
Sum of electronic and thermal Energies= -2211.829115
Sum of electronic and thermal Enthalpies= -2211.828171
Sum of electronic and thermal Free Energies= -2211.916079

2-IIII-radical

C	-2.93446	-1.10859	0.00015
C	-2.52243	0.21846	0.00015
C	-1.16116	0.50593	0.
C	-0.29395	-0.55644	-0.00016
C	-0.62935	-1.88835	-0.00026
C	-2.00137	-2.14559	-0.00009
H	-3.99148	-1.34315	0.0003
H	-3.22289	1.04544	0.00026
H	0.12474	-2.66268	-0.00066
H	-2.33707	-3.17535	-0.00015
O	0.73007	1.89152	-0.00025
C	-0.59322	1.89656	-0.00007
C	-1.01634	2.67844	1.2574
C	-1.01649	2.67857	-1.2574
F	-0.20504	3.74543	1.41806
F	-2.29133	3.09959	1.11763
F	-0.92357	1.87572	2.33885
F	-2.3137	3.03643	-1.14931
F	-0.25284	3.78535	-1.37727
F	-0.85398	1.89946	-2.34785
I	1.69761	0.10968	0.00006

Sum of electronic and zero-point Energies=-1317.164382
Sum of electronic and thermal Energies= -1317.150644
Sum of electronic and thermal Enthalpies= -1317.149699
Sum of electronic and thermal Free Energies= -1317.206882

2-IIII-SCF3

C	2.78872	2.53347	-0.0787
C	3.18535	1.22372	-0.31382
C	2.27986	0.18067	-0.13941
C	1.0132	0.52339	0.25857
C	0.55417	1.79211	0.51366
C	1.48729	2.81374	0.3311
H	3.49368	3.34408	-0.21405
H	4.18916	0.96587	-0.63234
H	-0.45947	1.99472	0.82838
H	1.17977	3.83591	0.514
O	1.66601	-2.0776	-0.15099
F	-3.93551	0.92128	-0.45801
F	-2.96359	-0.85487	-1.19914
F	-1.93008	1.02411	-1.25123
C	-2.77223	0.27822	-0.52401
S	-2.16147	-0.00054	1.15757
C	2.65512	-1.26809	-0.37758
C	3.78298	-1.73615	0.56074
C	3.06597	-1.52096	-1.84007
F	3.81223	-3.0855	0.59103
F	4.96674	-1.27042	0.10874
F	3.55867	-1.26286	1.805
F	4.29861	-1.01421	-2.05525
F	3.0771	-2.84921	-2.08113
F	2.18237	-0.92112	-2.66586
I	-0.18758	-1.1862	0.47172

Sum of electronic and zero-point Energies=-2053.030968
Sum of electronic and thermal Energies= -2053.011642
Sum of electronic and thermal Enthalpies= -2053.010697
Sum of electronic and thermal Free Energies= -2053.080979

3-BrIII-Br

C	-2.93446	-1.10859	0.00015

C	-2.52243	0.21846	0.00015
C	-1.16116	0.50593	0.
C	-0.29395	-0.55644	-0.00016
C	-0.62935	-1.88835	-0.00026
C	-2.00137	-2.14559	-0.00009
H	-3.99148	-1.34315	0.0003
H	-3.22289	1.04544	0.00026
H	0.12474	-2.66268	-0.00066
H	-2.33707	-3.17535	-0.00015
Br	1.57797	0.06966	0.00005
C	-0.59322	1.89656	-0.00007
O	0.73007	1.89152	-0.00025
O	-1.26974	2.88566	0.00007
Br	2.14787	-1.78352	0.00019

Sum of electronic and zero-point Energies=-5567.819293
Sum of electronic and thermal Energies= -5567.809519
Sum of electronic and thermal Enthalpies= -5567.808575
Sum of electronic and thermal Free Energies= -5567.856683

3-BrIII-CCH

C	-2.63836	2.37145	-0.00017
C	-2.96026	1.02422	-0.00021
C	-1.94291	0.08138	-0.00015
C	-0.6403	0.52162	-0.00005
C	-0.27053	1.84604	-0.00002
C	-1.30744	2.77375	-0.00008
H	-3.42304	3.11482	-0.00022
H	-3.98224	0.66925	-0.00028
H	0.76272	2.15392	0.00006
H	-1.06056	3.82641	-0.00005
Br	0.59854	-0.97968	0.00003
C	-2.19376	-1.39221	-0.00019
O	-1.06845	-2.08072	-0.00012
O	-3.29097	-1.87622	-0.00027
C	3.41985	1.17907	0.23987
H	4.27376	1.81274	0.35904
C	2.46124	0.4677	0.10608

Sum of electronic and zero-point Energies=-3070.309849
Sum of electronic and thermal Energies= -3070.299303
Sum of electronic and thermal Enthalpies= -3070.298358
Sum of electronic and thermal Free Energies= -3070.346970

3-BrIII-CF3

C	-2.69011	2.28973	0.00005
C	-2.95457	0.92757	0.0001
C	-1.91235	-0.00045	0.00001
C	-0.64991	0.52412	-0.00006
C	-0.30538	1.85846	-0.00013
C	-1.37667	2.75141	-0.00009
H	-3.50769	2.99983	0.00013
H	-3.96181	0.52596	0.00018
H	0.70804	2.22727	-0.00024
H	-1.16527	3.81354	-0.00016
Br	0.77117	-0.94889	0.00007
C	-2.19866	-1.51874	-0.00005
O	-1.13277	-2.20926	-0.00037
O	-3.35993	-1.86798	0.00015
C	2.40402	0.26982	0.00001
F	3.4394	-0.54682	0.00069
F	2.44547	1.03228	1.07774
F	2.44614	1.03133	-1.07837

Sum of electronic and zero-point Energies=-3331.257576
Sum of electronic and thermal Energies= -3331.245512
Sum of electronic and thermal Enthalpies= -3331.244568
Sum of electronic and thermal Free Energies= -3331.297228

3-BrIII-CH3

C	-2.69011	2.28973	0.00005
C	-2.95457	0.92757	0.0001
C	-1.91235	-0.00045	0.00001
C	-0.64991	0.52412	-0.00006
C	-0.30538	1.85846	-0.00013
C	-1.37667	2.75141	-0.00009
H	-3.50769	2.99983	0.00013
H	-3.96181	0.52596	0.00018
H	0.70804	2.22727	-0.00024
H	-1.16527	3.81354	-0.00016
Br	0.77117	-0.94889	0.00007
C	-2.19866	-1.51874	-0.00005
O	-1.13277	-2.20926	-0.00037
O	-3.35993	-1.86798	0.00015
C	2.40402	0.26982	0.00001
H	2.4376	0.8875	0.87308
H	3.24415	-0.39281	0.00056
H	2.43814	0.88672	-0.87359

Sum of electronic and zero-point Energies=-3033.473338
Sum of electronic and thermal Energies= -3033.463282
Sum of electronic and thermal Enthalpies= -3033.462338
Sum of electronic and thermal Free Energies= -3033.509749

3-BrIII-CHCH2

C	1.84919	2.09564	-0.00026
C	2.03211	0.72259	-0.00036
C	0.94595	-0.1438	-0.0002
C	-0.303	0.43	0.00006
C	-0.54379	1.79014	0.00034
C	0.56625	2.62624	0.00012
H	2.70392	2.75794	-0.00043
H	3.01223	0.26343	-0.00053
H	-1.5312	2.22037	0.00075
H	0.41394	3.69656	0.00029
C	1.13934	-1.67296	-0.00004
O	0.03732	-2.28217	-0.00078
O	2.28626	-2.08358	0.00075
C	-3.1018	0.35593	-0.00026
H	-3.09138	0.95627	-0.90317
C	-4.29648	-0.61585	-0.00019
H	-5.20975	-0.05832	-0.00042
H	-4.25478	-1.23241	-0.8737
Br	-1.68034	-0.75405	0.00029

Sum of electronic and zero-point Energies=-3071.542995
Sum of electronic and thermal Energies= -3071.532009
Sum of electronic and thermal Enthalpies= -3071.531065
Sum of electronic and thermal Free Energies= -3071.581079

3-BrIII-Cl

C	2.52236	2.11339	-0.00003
C	2.66085	0.73134	-0.00002
C	1.52519	-0.07221	-0.00001
C	0.30752	0.56194	0.00001
C	0.09823	1.91869	0.00008
C	1.25733	2.69731	0.00004
H	3.40253	2.74383	-0.00006
H	3.62846	0.24255	-0.00002
H	-0.89074	2.35437	0.00019
H	1.15606	3.77559	0.00007
Br	-1.17132	-0.78467	0.00008
C	1.58381	-1.57491	-0.00003
O	0.38663	-2.12239	-0.00002
O	2.61636	-2.18619	-0.00011
Cl	-2.94445	0.85661	-0.00013

Sum of electronic and zero-point Energies=-3453.817288
Sum of electronic and thermal Energies= -3453.807829
Sum of electronic and thermal Enthalpies= -3453.806885
Sum of electronic and thermal Free Energies= -3453.853435

3-BrIII-CN

C	2.38355	2.16507	-0.00011
C	2.54579	0.78572	-0.00018
C	1.42977	-0.04976	-0.00014
C	0.21531	0.57735	0.00019
C	-0.03278	1.92761	0.00009
C	1.11008	2.72995	0.00012
H	3.25324	2.81011	-0.00022
H	3.52047	0.31084	-0.00021
H	-1.02602	2.35531	0.00032
H	0.98659	3.8058	0.00018
Br	-1.29133	-0.79808	-0.00016
C	1.54327	-1.57372	0.00002
O	0.38892	-2.14584	0.00036
O	2.63468	-2.0876	0.00015
C	-2.68934	0.59238	0.00006
N	-3.53721	1.37008	0.00019

Sum of electronic and zero-point Energies=-3086.417659
Sum of electronic and thermal Energies= -3086.407384
Sum of electronic and thermal Enthalpies= -3086.406439
Sum of electronic and thermal Free Energies= -3086.454520

3-BrIII-F

C	-2.93446	-1.10859	0.00015
C	-2.52243	0.21846	0.00015
C	-1.16116	0.50593	0.
C	-0.29395	-0.55644	-0.00016
C	-0.62935	-1.88835	-0.00026
C	-2.00137	-2.14559	-0.00009
H	-3.99148	-1.34315	0.0003
H	-3.22289	1.04544	0.00026
H	0.12474	-2.66268	-0.00066
H	-2.33707	-3.17535	-0.00015
Br	1.57797	0.06966	0.00005
C	-0.59322	1.89656	-0.00007
O	0.73007	1.89152	-0.00025
O	-1.26974	2.88566	0.00007
F	2.14787	-1.78352	0.00019

Sum of electronic and zero-point Energies=-3093.451612
Sum of electronic and thermal Energies= -3093.442694
Sum of electronic and thermal Enthalpies= -3093.441750
Sum of electronic and thermal Free Energies= -3093.486600

3-BrIII-N3

C	-2.93446	-1.10859	0.00015
C	-2.52243	0.21846	0.00015
C	-1.16116	0.50593	0.
C	-0.29395	-0.55644	-0.00016
C	-0.62935	-1.88835	-0.00026
C	-2.00137	-2.14559	-0.00009

H	-3.99148	-1.34315	0.0003
H	-3.22289	1.04544	0.00026
H	0.12474	-2.66268	-0.00066
H	-2.33707	-3.17535	-0.00015
Br	1.57797	0.06966	0.00005
C	-0.59322	1.89656	-0.00007
O	0.73007	1.89152	-0.00025
O	-1.26974	2.88566	0.00007
N	2.11882	-1.68906	0.00018
N	3.3197	-1.96422	0.00031
N	3.73122	-3.30238	0.00041

Sum of electronic and zero-point Energies=-3157.776464
Sum of electronic and thermal Energies= -3157.765400
Sum of electronic and thermal Enthalpies= -3157.764456
Sum of electronic and thermal Free Energies= -3157.815429

3-BrIII-NH2

C	-2.63836	2.37145	-0.00017
C	-2.96026	1.02422	-0.00021
C	-1.94291	0.08138	-0.00015
C	-0.6403	0.52162	-0.00005
C	-0.27053	1.84604	-0.00002
C	-1.30744	2.77375	-0.00008
H	-3.42304	3.11482	-0.00022
H	-3.98224	0.66925	-0.00028
H	0.76272	2.15392	0.00006
H	-1.06056	3.82641	-0.00005
Br	0.59854	-0.97968	0.00003
C	-2.19376	-1.39221	-0.00019
O	-1.06845	-2.08072	-0.00012
O	-3.29097	-1.87622	-0.00027
N	2.2254	0.26686	0.11023
H	2.63529	0.67481	0.92605
H	2.62113	0.68613	-0.70684

Sum of electronic and zero-point Energies=-3049.520876
Sum of electronic and thermal Energies= -3049.511245
Sum of electronic and thermal Enthalpies= -3049.510301
Sum of electronic and thermal Free Energies= -3049.556467

3-BrIII-NHAc

C	-2.96551	2.1948	0.00012
C	-3.14135	0.82115	0.00005
C	-2.02605	-0.00366	-0.00001
C	-0.77557	0.56842	0.00001
C	-0.55317	1.92978	0.00009
C	-1.68457	2.73807	0.00014
H	-3.82524	2.85	0.00016
H	-4.11978	0.3599	0.00003
H	0.44096	2.34251	0.00011
H	-1.55512	3.81166	0.0002
Br	0.5975	-0.7825	-0.00007
C	-2.1044	-1.49596	-0.00007
O	-0.90086	-2.04357	-0.00014
O	-3.13436	-2.1089	-0.00015
C	3.15326	0.18449	-0.00018
O	3.38959	-0.99771	-0.00038
C	4.2019	1.26499	0.0006
H	4.07476	1.89669	-0.87709
H	4.0784	1.89115	0.88282
H	5.18679	0.80999	-0.00262
N	1.91064	0.67979	-0.00004
H	1.90346	1.56189	0.47096

Sum of electronic and zero-point Energies=-3202.143543
Sum of electronic and thermal Energies= -3202.130136
Sum of electronic and thermal Enthalpies= -3202.129192
Sum of electronic and thermal Free Energies= -3202.185467

3-BrIII-OCF3

C	2.67388	2.50582	-0.08055
C	3.07538	1.1861	-0.2436
C	2.13864	0.16777	-0.09614
C	0.85091	0.5348	0.20224
C	0.38735	1.81389	0.38416
C	1.35008	2.81324	0.23105
H	3.39497	3.30541	-0.19349
H	4.09694	0.91345	-0.48175
H	-0.64249	2.02395	0.63
H	1.04878	3.8455	0.35987
Br	-0.29449	-1.08279	0.36657
C	2.46298	-1.28967	-0.23514
O	1.40068	-2.06043	-0.03196
O	3.55405	-1.70721	-0.49333
F	-3.25665	-0.89735	-0.63674
F	-2.19308	0.86999	-1.26105
F	-3.78459	1.02948	0.19587
C	-2.75499	0.26941	-0.18023
O	-1.90316	0.11538	0.81056

Sum of electronic and zero-point Energies=-3406.502563
Sum of electronic and thermal Energies= -3406.489845
Sum of electronic and thermal Enthalpies= -3406.488901
Sum of electronic and thermal Free Energies= -3406.543959

3-BrIII-OCH3

C	2.67388	2.50582	-0.08055
C	3.07538	1.1861	-0.2436
C	2.13864	0.16777	-0.09614
C	0.85091	0.5348	0.20224
C	0.38735	1.81389	0.38416
C	1.35008	2.81324	0.23105
H	3.39497	3.30541	-0.19349
H	4.09694	0.91345	-0.48175
H	-0.64249	2.02395	0.63
H	1.04878	3.8455	0.35987
Br	-0.29449	-1.08279	0.36657
C	2.46298	-1.28967	-0.23514
O	1.40068	-2.06043	-0.03196
O	3.55405	-1.70721	-0.49333
C	-2.75499	0.26941	-0.18023
O	-1.90316	0.11538	0.81056
H	-3.5809	0.87912	0.12146
H	-2.3123	0.74256	-1.03173
H	-3.15272	-0.65563	-0.54216

Sum of electronic and zero-point Energies=-3108.674433
Sum of electronic and thermal Energies= -3108.663496
Sum of electronic and thermal Enthalpies= -3108.662552
Sum of electronic and thermal Free Energies= -3108.711908

3-BrIII-OCOCF3

C	3.22289	2.62188	0.00859
C	3.68898	1.31361	0.00651
C	2.77394	0.26568	0.00105
C	1.43871	0.58809	-0.00237
C	0.91134	1.85792	-0.00002
C	1.85448	2.88734	0.0057
H	3.92673	3.44448	0.01292
H	4.74543	1.07154	0.00947
H	-0.15229	2.04153	-0.00164
H	1.50103	3.91113	0.00798
Br	0.34895	-1.0718	-0.00868
C	3.17219	-1.17944	0.00108
O	2.11919	-1.99141	-0.00185
O	4.30614	-1.55945	0.00406
C	-2.41859	-0.53165	0.0117
O	-2.5234	-1.72768	0.04512
O	-1.32746	0.16971	-0.02174
C	-3.65945	0.39701	0.00003
F	-4.78188	-0.30037	0.05516
F	-3.67375	1.13597	-1.11316
F	-3.62623	1.23022	1.0443

Sum of electronic and zero-point Energies=-3519.822982
Sum of electronic and thermal Energies= -3519.808104
Sum of electronic and thermal Enthalpies= -3519.807160
Sum of electronic and thermal Free Energies= -3519.868686

3-BrIII-OCOCH3

C	2.94595	2.24262	0.
C	3.14905	0.86891	-0.00023
C	2.05101	0.01452	-0.00014
C	0.80079	0.58421	0.00015
C	0.53351	1.93493	0.00041
C	1.65414	2.76631	0.00033
H	3.79558	2.9138	-0.00007
H	4.13833	0.42604	-0.00045
H	-0.47532	2.31781	0.00073
H	1.50439	3.83907	0.00055
Br	-0.60316	-0.8154	0.00013
C	2.175	-1.48618	-0.00024
O	1.00226	-2.08015	0.
O	3.23627	-2.04801	-0.00049
C	-3.20495	0.1778	0.00021
O	-3.42588	-1.00956	0.00076
O	-1.9799	0.68529	0.00005
C	-4.26655	1.25034	-0.00099
H	-4.14463	1.88403	0.87785
H	-4.15011	1.8754	-0.88681
H	-5.24737	0.78154	0.00398

Sum of electronic and zero-point Energies=-3222.034721
Sum of electronic and thermal Energies= -3222.021696
Sum of electronic and thermal Enthalpies= -3222.020752
Sum of electronic and thermal Free Energies= -3222.076214

3-BrIII-OCOPh

C	-2.96551	2.1948	0.00012
C	-3.14135	0.82115	0.00005
C	-2.02605	-0.00366	-0.00001
C	-0.77557	0.56842	0.00001
C	-0.55317	1.92978	0.00009
C	-1.68457	2.73807	0.00014
H	-3.82524	2.85	0.00016
H	-4.11978	0.3599	0.00003
H	0.44096	2.34251	0.00011
H	-1.55512	3.81166	0.0002
Br	0.5975	-0.7825	-0.00007
C	-2.1044	-1.49596	-0.00007
O	-0.90086	-2.04357	-0.00014
O	-3.13436	-2.1089	-0.00015

C	3.15326	0.18449	-0.00018
O	3.38959	-0.99771	-0.0023
O	1.91808	0.67682	0.00235
C	4.22579	1.2896	0.00062
C	5.57865	0.94865	-0.00127
C	3.84477	2.63138	0.00313
C	6.55019	1.94931	0.00003
H	5.87872	-0.10927	-0.00246
C	4.81648	3.63247	0.00343
H	2.77855	2.90028	0.00444
C	6.16903	3.29167	0.00202
H	7.61655	1.68065	-0.00082
H	4.51576	4.69032	0.00499
H	6.93524	4.08049	0.00299

Sum of electronic and zero-point Energies=-3413.712441
Sum of electronic and thermal Energies= -3413.696574
Sum of electronic and thermal Enthalpies= -3413.695630
Sum of electronic and thermal Free Energies= -3413.759196

3-BrIII-OH

C	2.85263	-1.32247	0.00438
C	2.53897	0.03132	0.02066
C	1.20519	0.42867	0.01343
C	0.26118	-0.56546	0.00447
C	0.49803	-1.91871	-0.02637
C	1.8446	-2.28523	-0.02237
H	3.88939	-1.63508	0.00637
H	3.29795	0.80511	0.03341
H	-0.31078	-2.63443	-0.06185
H	2.09873	-3.33788	-0.04592
Br	-1.58476	0.16966	-0.02049
C	0.76361	1.8772	0.00979
O	-0.53232	1.99106	-0.02484
O	1.56652	2.77539	0.03319
O	-2.35941	-1.6203	-0.02187
H	-2.69235	-1.77691	0.86934

Sum of electronic and zero-point Energies=-3069.409202
Sum of electronic and thermal Energies= -3069.399781
Sum of electronic and thermal Enthalpies= -3069.398837
Sum of electronic and thermal Free Energies= -3069.444552

3-BrIII-OTf

C	-2.93446	-1.10859	-0.00888
C	-2.52242	0.21846	-0.00736
C	-1.16116	0.50593	-0.00483
C	-0.29394	-0.55644	-0.00398
C	-0.62934	-1.88835	-0.00546
C	-2.00136	-2.14559	-0.00799
H	-3.99147	-1.34315	-0.01083
H	-3.22288	1.04544	-0.00808
H	0.12474	-2.66268	-0.00491
H	-2.33706	-3.17535	-0.00925
Br	1.57797	0.06966	0.00005
C	-0.59322	1.89656	-0.00305
O	0.73007	1.89152	-0.00078
O	-1.26973	2.88566	-0.00361
O	2.10706	-1.65082	0.00018
S	3.59307	-1.98174	0.66431
O	3.33018	-2.88233	1.67519
O	4.05421	-0.70398	0.94073
C	4.38339	-2.66789	-0.57196
F	4.63907	-1.73642	-1.51509
F	5.54654	-3.19005	-0.12819
F	3.62322	-3.64906	-1.10295

Sum of electronic and zero-point Energies=-3955.103667
Sum of electronic and thermal Energies= -3955.087592
Sum of electronic and thermal Enthalpies= -3955.086648
Sum of electronic and thermal Free Energies= -3955.150396

3-BrIII-OTs

C	-2.93446	-1.10859	-0.00888
C	-2.52242	0.21846	-0.00736
C	-1.16116	0.50593	-0.00483
C	-0.29394	-0.55644	-0.00398
C	-0.62934	-1.88835	-0.00546
C	-2.00136	-2.14559	-0.00799
H	-3.99147	-1.34315	-0.01083
H	-3.22288	1.04544	-0.00808
H	0.12474	-2.66268	-0.00491
H	-2.33706	-3.17535	-0.00925
Br	1.57797	0.06966	0.00005
C	-0.59322	1.89656	-0.00305
O	0.73007	1.89152	-0.00078
O	-1.26973	2.88566	-0.00361
O	2.10706	-1.65082	0.00018
S	3.59307	-1.98174	0.66431
O	3.33018	-2.88233	1.67519
O	4.05421	-0.70398	0.94073
C	4.39807	-2.68064	-0.59493
C	4.28967	-4.05772	-0.84102
C	5.21363	-1.91787	-1.44488
C	4.96741	-4.65349	-1.89801
H	3.65893	-4.67381	-0.17439
C	5.89555	-2.50544	-2.5029
H	5.3178	-0.83348	-1.2578
C	5.77613	-3.88142	-2.74243
H	4.86999	-5.73576	-2.07304
H	6.53296	-1.88994	-3.15578
C	6.4851	-4.50936	-3.87989
H	7.41139	-3.94052	-4.14083
H	6.77023	-5.56379	-3.64125
H	5.81659	-4.52222	-4.77855

Sum of electronic and zero-point Energies=-3888.280440
Sum of electronic and thermal Energies= -3888.261453
Sum of electronic and thermal Enthalpies= -3888.260508
Sum of electronic and thermal Free Energies= -3888.332186

3-BrIII-radical

C	-2.93446	-1.10859	0.00015
C	-2.52243	0.21846	0.00015
C	-1.16116	0.50593	0.
C	-0.29395	-0.55644	-0.00016
C	-0.62935	-1.88835	-0.00026
C	-2.00137	-2.14559	-0.00009
H	-3.99148	-1.34315	0.0003
H	-3.22289	1.04544	0.00026
H	0.12474	-2.66268	-0.00066
H	-2.33707	-3.17535	-0.00015
Br	1.57797	0.06966	0.00005
C	-0.59322	1.89656	-0.00007
O	0.73007	1.89152	-0.00025
O	-1.26974	2.88566	0.00007

Sum of electronic and zero-point Energies=-2993.633228
Sum of electronic and thermal Energies= -2993.624874
Sum of electronic and thermal Enthalpies= -2993.623930
Sum of electronic and thermal Free Energies= -2993.668716

3-BrIII-SCF3

C	2.78872	2.53347	-0.0787
C	3.18535	1.22372	-0.31382
C	2.27986	0.18067	-0.13941
C	1.0132	0.52339	0.25857
C	0.55417	1.79211	0.51366
C	1.48729	2.81374	0.3311
H	3.49368	3.34408	-0.21405
H	4.18916	0.96587	-0.63234
H	-0.45947	1.99472	0.82838
H	1.17977	3.83591	0.514
C	2.65512	-1.26809	-0.37758
O	1.66601	-2.0776	-0.15099
O	3.76604	-1.5693	-0.73478
F	-3.93551	0.92128	-0.45801
F	-2.96359	-0.85487	-1.19914
F	-1.93008	1.02411	-1.25123
C	-2.77223	0.27822	-0.52401
S	-2.16147	-0.00054	1.15757
Br	-0.14178	-1.121	0.46359

Sum of electronic and zero-point Energies=-3729.462802
Sum of electronic and thermal Energies= -3729.449202
Sum of electronic and thermal Enthalpies= -3729.448258
Sum of electronic and thermal Free Energies= -3729.505494

3-ClIII-Br

C	-2.93446	-1.10859	0.00015
C	-2.52243	0.21846	0.00015
C	-1.16116	0.50593	0.
C	-0.29395	-0.55644	-0.00016
C	-0.62935	-1.88835	-0.00026
C	-2.00137	-2.14559	-0.00009
H	-3.99148	-1.34315	0.0003
H	-3.22289	1.04544	0.00026
H	0.12474	-2.66268	-0.00066
H	-2.33707	-3.17535	-0.00015
C	-0.59322	1.89656	-0.00007
O	0.73007	1.89152	-0.00025
O	-1.26974	2.88566	0.00007
Br	2.14787	-1.78352	0.00019
Cl	1.57797	0.06966	0.00005

Sum of electronic and zero-point Energies=-3453.791825
Sum of electronic and thermal Energies= -3453.782353
Sum of electronic and thermal Enthalpies= -3453.781409
Sum of electronic and thermal Free Energies= -3453.828422

3-ClIII-CCH

C	-2.63836	2.37145	-0.00017
C	-2.96026	1.02422	-0.00021
C	-1.94291	0.08138	-0.00015
C	-0.6403	0.52162	-0.00005
C	-0.27053	1.84604	-0.00002
C	-1.30744	2.77375	-0.00008
H	-3.42304	3.11482	-0.00022
H	-3.98224	0.66925	-0.00028
H	0.76272	2.15392	0.00006
H	-1.06056	3.82641	-0.00005
C	-2.19376	-1.39221	-0.00019
O	-1.06845	-2.08072	-0.00012

O	-3.29097	-1.87622	-0.00027
C	3.41985	1.17907	0.23987
H	4.27376	1.81274	0.35904
C	2.46124	0.4677	0.10608
Cl	0.69628	-1.09812	0.00003

Sum of electronic and zero-point Energies= -956.293392
Sum of electronic and thermal Energies= -956.283054
Sum of electronic and thermal Enthalpies=-956.282110
Sum of electronic and thermal Free Energies= -956.329874

3-ClIII-CF3

C	-2.69011	2.28973	0.00005
C	-2.95457	0.92757	0.0001
C	-1.91235	-0.00045	0.00001
C	-0.64991	0.52412	-0.00006
C	-0.30538	1.85846	-0.00013
C	-1.37667	2.75141	-0.00009
H	-3.50769	2.99983	0.00013
H	-3.96181	0.52596	0.00018
H	0.70804	2.22727	-0.00024
H	-1.16527	3.81354	-0.00016
C	-2.19866	-1.51874	-0.00005
O	-1.13277	-2.20926	-0.00037
O	-3.35993	-1.86798	0.00015
C	2.40402	0.26982	0.00001
F	3.4394	-0.54682	0.00069
F	2.44547	1.03228	1.07774
F	2.44614	1.03133	-1.07837
Cl	0.77117	-0.94889	0.00007

Sum of electronic and zero-point Energies=-1217.245340
Sum of electronic and thermal Energies= -1217.233622
Sum of electronic and thermal Enthalpies= -1217.232678
Sum of electronic and thermal Free Energies= -1217.284251

3-ClIII-CH3

C	-2.69011	2.28973	0.00005
C	-2.95457	0.92757	0.0001
C	-1.91235	-0.00045	0.00001
C	-0.64991	0.52412	-0.00006
C	-0.30538	1.85846	-0.00013
C	-1.37667	2.75141	-0.00009
H	-3.50769	2.99983	0.00013
H	-3.96181	0.52596	0.00018
H	0.70804	2.22727	-0.00024
H	-1.16527	3.81354	-0.00016
C	-2.19866	-1.51874	-0.00005
O	-1.13277	-2.20926	-0.00037
O	-3.35993	-1.86798	0.00015
C	2.40402	0.26982	0.00001
H	2.4376	0.8875	0.87308
H	3.24415	-0.39281	0.00056
H	2.43814	0.88672	-0.87359
Cl	0.77117	-0.94889	0.00007

Sum of electronic and zero-point Energies= -919.463553
Sum of electronic and thermal Energies= -919.453729
Sum of electronic and thermal Enthalpies=-919.452785
Sum of electronic and thermal Free Energies= -919.499345

3-ClIII-CHCH2

C	1.84919	2.09564	-0.00026
C	2.03211	0.72259	-0.00036
C	0.94595	-0.1438	-0.0002
C	-0.303	0.43	0.00006
C	-0.54379	1.79014	0.00034
C	0.56625	2.62624	0.00012
H	2.70392	2.75794	-0.00043
H	3.01223	0.26343	-0.00053
H	-1.5312	2.22037	0.00075
H	0.41394	3.69656	0.00029
C	1.13934	-1.67296	-0.00004
O	0.03732	-2.28217	-0.00078
O	2.28626	-2.08358	0.00075
C	-3.1018	0.35593	-0.00026
H	-3.09138	0.95627	-0.90317
Cl	-1.68034	-0.75405	0.00029
C	-4.29648	-0.61585	-0.00019
H	-5.20975	-0.05832	-0.00042
H	-4.25478	-1.23241	-0.8737

Sum of electronic and zero-point Energies= -957.534569
Sum of electronic and thermal Energies= -957.523823
Sum of electronic and thermal Enthalpies=-957.522878
Sum of electronic and thermal Free Energies= -957.571934

3-ClIII-Cl

C	2.52236	2.11339	-0.00003
C	2.66085	0.73134	-0.00002
C	1.52519	-0.07221	-0.00001
C	0.30752	0.56194	0.00001
C	0.09823	1.91869	0.00008
C	1.25733	2.69731	0.00004
H	3.40253	2.74383	-0.00006
H	3.62846	0.24255	-0.00002
H	-0.89074	2.35437	0.00019
H	1.15606	3.77559	0.00007
C	1.58381	-1.57491	-0.00003
O	0.38663	-2.12239	-0.00002
O	2.61636	-2.18619	-0.00011
Cl	-2.94445	0.85661	-0.00013
Cl	-0.99381	-0.62303	0.00007

Sum of electronic and zero-point Energies=-1339.789322
Sum of electronic and thermal Energies= -1339.780130
Sum of electronic and thermal Enthalpies= -1339.779186
Sum of electronic and thermal Free Energies= -1339.824735

3-ClIII-CN

C	2.38355	2.16507	-0.00011
C	2.54579	0.78572	-0.00018
C	1.42977	-0.04976	-0.00014
C	0.21531	0.57735	0.00019
C	-0.03278	1.92761	0.00009
C	1.11008	2.72995	0.00012
H	3.25324	2.81011	-0.00022
H	3.52047	0.31084	-0.00021
H	-1.02602	2.35531	0.00032
H	0.98659	3.8058	0.00018
C	1.54327	-1.57372	0.00002
O	0.38892	-2.14584	0.00036
O	2.63468	-2.0876	0.00015
C	-2.68934	0.59238	0.00006
N	-3.53721	1.37008	0.00019
Cl	-1.44146	-0.64875	-0.00014

Sum of electronic and zero-point Energies= -972.396030
Sum of electronic and thermal Energies= -972.386004
Sum of electronic and thermal Enthalpies=-972.385060
Sum of electronic and thermal Free Energies= -972.432202

3-ClIII-F

C	-2.93446	-1.10859	0.00015
C	-2.52243	0.21846	0.00015
C	-1.16116	0.50593	0.
C	-0.29395	-0.55644	-0.00016
C	-0.62935	-1.88835	-0.00026
C	-2.00137	-2.14559	-0.00009
H	-3.99148	-1.34315	0.0003
H	-3.22289	1.04544	0.00026
H	0.12474	-2.66268	-0.00066
H	-2.33707	-3.17535	-0.00015
C	-0.59322	1.89656	-0.00007
O	0.73007	1.89152	-0.00025
O	-1.26974	2.88566	0.00007
F	2.14787	-1.78352	0.00019
Cl	1.37517	0.00183	0.00002

Sum of electronic and zero-point Energies= -979.423607
Sum of electronic and thermal Energies= -979.415082
Sum of electronic and thermal Enthalpies=-979.414138
Sum of electronic and thermal Free Energies= -979.457630

3-ClIII-N3

C	-2.93446	-1.10859	0.00015
C	-2.52243	0.21846	0.00015
C	-1.16116	0.50593	0.
C	-0.29395	-0.55644	-0.00016
C	-0.62935	-1.88835	-0.00026
C	-2.00137	-2.14559	-0.00009
H	-3.99148	-1.34315	0.0003
H	-3.22289	1.04544	0.00026
H	0.12474	-2.66268	-0.00066
H	-2.33707	-3.17535	-0.00015
C	-0.59322	1.89656	-0.00007
O	0.73007	1.89152	-0.00025
O	-1.26974	2.88566	0.00007
N	2.11882	-1.68906	0.00018
N	3.3197	-1.96422	0.00031
N	3.73122	-3.30238	0.00041
Cl	1.57797	0.06966	0.00005

Sum of electronic and zero-point Energies=-1043.752192
Sum of electronic and thermal Energies= -1043.741414
Sum of electronic and thermal Enthalpies= -1043.740470
Sum of electronic and thermal Free Energies= -1043.790460

3-ClIII-NH2

C	-2.63836	2.37145	-0.00017
C	-2.96026	1.02422	-0.00021
C	-1.94291	0.08138	-0.00015
C	-0.6403	0.52162	-0.00005
C	-0.27053	1.84604	-0.00002
C	-1.30744	2.77375	-0.00008
H	-3.42304	3.11482	-0.00022
H	-3.98224	0.66925	-0.00028
H	0.76272	2.15392	0.00006
H	-1.06056	3.82641	-0.00005
C	-2.19376	-1.39221	-0.00019
O	-1.06845	-2.08072	-0.00012
O	-3.29097	-1.87622	-0.00027
N	2.2254	0.26686	0.11023
H	2.63529	0.67481	0.92605

H	2.62113	0.68613	-0.70684
Cl	0.69628	-1.09812	0.00003

Sum of electronic and zero-point Energies= -935.501211
Sum of electronic and thermal Energies= -935.491837
Sum of electronic and thermal Enthalpies=-935.490893
Sum of electronic and thermal Free Energies= -935.536166

3-ClIII-NHAc

C	-2.96551	2.1948	0.00012
C	-3.14135	0.82115	0.00005
C	-2.02605	-0.00366	-0.00001
C	-0.77557	0.56842	0.00001
C	-0.55317	1.92978	0.00009
C	-1.68457	2.73807	0.00014
H	-3.82524	2.85	0.00016
H	-4.11978	0.3599	0.00003
H	0.44096	2.34251	0.00011
H	-1.55512	3.81166	0.0002
C	-2.1044	-1.49596	-0.00007
O	-0.90086	-2.04357	-0.00014
O	-3.13436	-2.1089	-0.00015
C	3.15326	0.18449	-0.00018
O	3.38959	-0.99771	-0.00038
C	4.2019	1.26499	0.0006
H	4.07476	1.89669	-0.87709
H	4.0784	1.89115	0.88282
H	5.18679	0.80999	-0.00262
N	1.91064	0.67979	-0.00004
H	1.90346	1.56189	0.47096
Cl	0.72137	-0.90438	-0.00008

Sum of electronic and zero-point Energies=-1088.120509
Sum of electronic and thermal Energies= -1088.107501
Sum of electronic and thermal Enthalpies= -1088.106557
Sum of electronic and thermal Free Energies= -1088.161270

3-ClIII-OCF3

C	2.67388	2.50582	-0.08055
C	3.07538	1.1861	-0.2436
C	2.13864	0.16777	-0.09614
C	0.85091	0.5348	0.20224
C	0.38735	1.81389	0.38416
C	1.35008	2.81324	0.23105
H	3.39497	3.30541	-0.19349
H	4.09694	0.91345	-0.48175
H	-0.64249	2.02395	0.63
H	1.04878	3.8455	0.35987
C	2.46298	-1.28967	-0.23514
O	1.40068	-2.06043	-0.03196
O	3.55405	-1.70721	-0.49333
F	-3.25665	-0.89735	-0.63674
F	-2.19308	0.86999	-1.26105
F	-3.78459	1.02948	0.19587
C	-2.75499	0.26941	-0.18023
O	-1.90316	0.11538	0.81056
Cl	-0.16269	-0.89665	0.34766

Sum of electronic and zero-point Energies=-1292.474997
Sum of electronic and thermal Energies= -1292.462622
Sum of electronic and thermal Enthalpies= -1292.461678
Sum of electronic and thermal Free Energies= -1292.515498

3-ClIII-OCH3

C	2.67388	2.50582	-0.08055
C	3.07538	1.1861	-0.2436
C	2.13864	0.16777	-0.09614
C	0.85091	0.5348	0.20224
C	0.38735	1.81389	0.38416
C	1.35008	2.81324	0.23105
H	3.39497	3.30541	-0.19349
H	4.09694	0.91345	-0.48175
H	-0.64249	2.02395	0.63
H	1.04878	3.8455	0.35987
C	2.46298	-1.28967	-0.23514
O	1.40068	-2.06043	-0.03196
O	3.55405	-1.70721	-0.49333
C	-2.75499	0.26941	-0.18023
O	-1.90316	0.11538	0.81056
H	-3.5809	0.87912	0.12146
H	-2.3123	0.74256	-1.03173
H	-3.15272	-0.65563	-0.54216
Cl	-0.16269	-0.89665	0.34766

Sum of electronic and zero-point Energies= -994.650923
Sum of electronic and thermal Energies= -994.640356
Sum of electronic and thermal Enthalpies=-994.639412
Sum of electronic and thermal Free Energies= -994.687521

3-ClIII-OCOCF3

C	3.22289	2.62188	0.00859
C	3.68898	1.31361	0.00651
C	2.77394	0.26568	0.00105
C	1.43871	0.58809	-0.00237
C	0.91134	1.85792	-0.00002
C	1.85448	2.88734	0.0057
H	3.92673	3.44448	0.01292
H	4.74543	1.07154	0.00947
H	-0.15229	2.04153	-0.00164
H	1.50103	3.91113	0.00798
C	3.17219	-1.17944	0.00108
O	2.11919	-1.99141	-0.00185
O	4.30614	-1.55945	0.00406
C	-2.41859	-0.53165	0.0117
O	-2.5234	-1.72768	0.04512
O	-1.32746	0.16971	-0.02174
C	-3.65945	0.39701	0.00003
F	-4.78188	-0.30037	0.05516
F	-3.67375	1.13597	-1.11316
F	-3.62623	1.23022	1.0443
Cl	0.47279	-0.88316	-0.00796

Sum of electronic and zero-point Energies=-1405.795067
Sum of electronic and thermal Energies= -1405.780490
Sum of electronic and thermal Enthalpies= -1405.779546
Sum of electronic and thermal Free Energies= -1405.840304

3-ClIII-OCOCH3

C	-2.77683	-0.58423	0.00036
C	-2.39776	0.75737	0.00014
C	-1.05787	1.07331	0.
C	-0.11297	0.07044	0.00005
C	-0.44469	-1.26455	0.00033
C	-1.79973	-1.58173	0.00051
H	-3.13782	1.5457	0.00012
H	0.30278	-2.03783	0.00043
H	-2.07752	-2.62454	0.00085
C	-0.52225	2.46371	-0.00019
O	0.8004	2.43757	-0.00032
O	-1.1913	3.45678	-0.00029
C	3.55808	-0.94997	0.00034
O	4.18941	0.07366	0.00147
O	2.22458	-0.9935	-0.0005
C	4.16192	-2.33025	-0.00075
H	3.82308	-2.87846	0.87685
H	3.82951	-2.87366	-0.88383
H	5.2437	-2.24584	0.0032
Cl	1.5424	0.73799	-0.00023
H	-3.82946	-0.77629	0.00053

Sum of electronic and zero-point Energies=-1108.006387
Sum of electronic and thermal Energies= -1107.993680
Sum of electronic and thermal Enthalpies= -1107.992736
Sum of electronic and thermal Free Energies= -1108.047178

3-ClIII-OCOPh

C	-2.96551	2.1948	0.00012
C	-3.14135	0.82115	0.00005
C	-2.02605	-0.00366	-0.00001
C	-0.77557	0.56842	0.00001
C	-0.55317	1.92978	0.00009
C	-1.68457	2.73807	0.00014
H	-3.82524	2.85	0.00016
H	-4.11978	0.3599	0.00003
H	0.44096	2.34251	0.00011
H	-1.55512	3.81166	0.0002
C	-2.1044	-1.49596	-0.00007
O	-0.90086	-2.04357	-0.00014
O	-3.13436	-2.1089	-0.00015
C	3.15326	0.18449	-0.00018
O	3.38959	-0.99771	-0.0023
O	1.91808	0.67682	0.00235
C	4.22579	1.2896	0.00062
C	5.57865	0.94865	-0.00127
C	3.84477	2.63138	0.00313
C	6.55019	1.94931	0.00003
H	5.87872	-0.10927	-0.00246
C	4.81648	3.63247	0.00343
H	2.77855	2.90028	0.00444
C	6.16903	3.29167	0.00202
H	7.61655	1.68065	-0.00082
H	4.51576	4.69032	0.00499
H	6.93524	4.08049	0.00299
Cl	0.47901	-0.66593	-0.00006

Sum of electronic and zero-point Energies=-1299.683848
Sum of electronic and thermal Energies= -1299.668300
Sum of electronic and thermal Enthalpies= -1299.667355
Sum of electronic and thermal Free Energies= -1299.729913

3-ClIII-OH

C	2.85263	-1.32247	0.00438
C	2.53897	0.03132	0.02066
C	1.20519	0.42867	0.01343
C	0.26118	-0.56546	0.00447
C	0.49803	-1.91871	-0.02637
C	1.8446	-2.28523	-0.02237
H	3.88939	-1.63508	0.00637
H	3.29795	0.80511	0.03341
H	-0.31078	-2.63443	-0.06185
H	2.09873	-3.33788	-0.04592
C	0.76361	1.8772	0.00979
O	-0.53232	1.99106	-0.02484

O	1.56652	2.77539	0.03319
O	-2.35941	-1.6203	-0.02187
H	-2.69235	-1.77691	0.86934
Cl	-1.3738	0.08565	-0.01763

Sum of electronic and zero-point Energies= -955.383830
Sum of electronic and thermal Energies= -955.374670
Sum of electronic and thermal Enthalpies=-955.373725
Sum of electronic and thermal Free Energies= -955.418397

3-ClIII-OTf

C	-2.93446	-1.10859	-0.00888
C	-2.52242	0.21846	-0.00736
C	-1.16116	0.50593	-0.00483
C	-0.29394	-0.55644	-0.00398
C	-0.62934	-1.88835	-0.00546
C	-2.00136	-2.14559	-0.00799
H	-3.99147	-1.34315	-0.01083
H	-3.22288	1.04544	-0.00808
H	0.12474	-2.66268	-0.00491
H	-2.33706	-3.17535	-0.00925
C	-0.59322	1.89656	-0.00305
O	0.73007	1.89152	-0.00078
O	-1.26973	2.88566	-0.00361
O	2.10706	-1.65082	0.00018
S	3.59307	-1.98174	0.66431
O	3.33018	-2.88233	1.67519
O	4.05421	-0.70398	0.94073
C	4.38339	-2.66789	-0.57196
F	4.63907	-1.73642	-1.51509
F	5.54654	-3.19005	-0.12819
F	3.62322	-3.64906	-1.10295
Cl	1.57797	0.06966	0.00005

Sum of electronic and zero-point Energies=-1841.076792
Sum of electronic and thermal Energies= -1841.061071
Sum of electronic and thermal Enthalpies= -1841.060126
Sum of electronic and thermal Free Energies= -1841.122290

3-ClIII-OTs

C	-2.93446	-1.10859	-0.00888
C	-2.52242	0.21846	-0.00736
C	-1.16116	0.50593	-0.00483
C	-0.29394	-0.55644	-0.00398
C	-0.62934	-1.88835	-0.00546
C	-2.00136	-2.14559	-0.00799
H	-3.99147	-1.34315	-0.01083
H	-3.22288	1.04544	-0.00808
H	0.12474	-2.66268	-0.00491
H	-2.33706	-3.17535	-0.00925
C	-0.59322	1.89656	-0.00305
O	0.73007	1.89152	-0.00078
O	-1.26973	2.88566	-0.00361
O	2.10706	-1.65082	0.00018
S	3.59307	-1.98174	0.66431
O	3.33018	-2.88233	1.67519
O	4.05421	-0.70398	0.94073
C	4.39807	-2.68064	-0.59493
C	4.28967	-4.05772	-0.84102
C	5.21363	-1.91787	-1.44488
C	4.96741	-4.65349	-1.89801
H	3.65893	-4.67381	-0.17439
C	5.89555	-2.50544	-2.5029
H	5.3178	-0.83348	-1.2578
C	5.77613	-3.88142	-2.74243
H	4.86999	-5.73576	-2.07304
H	6.53296	-1.88994	-3.15578
C	6.4851	-4.50936	-3.87989
H	7.41139	-3.94052	-4.14083
H	6.77023	-5.56379	-3.64125
H	5.81659	-4.52222	-4.77855
Cl	1.57797	0.06966	0.00005

Sum of electronic and zero-point Energies=-1774.252991
Sum of electronic and thermal Energies= -1774.234332
Sum of electronic and thermal Enthalpies= -1774.233388
Sum of electronic and thermal Free Energies= -1774.303902

3-ClIII-radical

C	-2.93446	-1.10859	0.00015
C	-2.52243	0.21846	0.00015
C	-1.16116	0.50593	0.
C	-0.29395	-0.55644	-0.00016
C	-0.62935	-1.88835	-0.00026
C	-2.00137	-2.14559	-0.00009
H	-3.99148	-1.34315	0.0003
H	-3.22289	1.04544	0.00026
H	0.12474	-2.66268	-0.00066
H	-2.33707	-3.17535	-0.00015
C	-0.59322	1.89656	-0.00007
O	0.73007	1.89152	-0.00025
O	-1.26974	2.88566	0.00007
Cl	1.37517	0.00183	0.00002

Sum of electronic and zero-point Energies= -879.638755
Sum of electronic and thermal Energies= -879.630626
Sum of electronic and thermal Enthalpies=-879.629682
Sum of electronic and thermal Free Energies= -879.673284

3-ClIII-SCF3

C	2.78872	2.53347	-0.0787
C	3.18535	1.22372	-0.31382
C	2.27986	0.18067	-0.13941
C	1.0132	0.52339	0.25857
C	0.55417	1.79211	0.51366
C	1.48729	2.81374	0.3311
H	3.49368	3.34408	-0.21405
H	4.18916	0.96587	-0.63234
H	-0.45947	1.99472	0.82838
H	1.17977	3.83591	0.514
C	2.65512	-1.26809	-0.37758
O	1.66601	-2.0776	-0.15099
O	3.76604	-1.5693	-0.73478
F	-3.93551	0.92128	-0.45801
F	-2.96359	-0.85487	-1.19914
F	-1.93008	1.02411	-1.25123
C	-2.77223	0.27822	-0.52401
S	-2.16147	-0.00054	1.15757
Cl	0.00684	-0.90941	0.43721

Sum of electronic and zero-point Energies=-1615.438795
Sum of electronic and thermal Energies= -1615.425425
Sum of electronic and thermal Enthalpies= -1615.424481
Sum of electronic and thermal Free Energies= -1615.480974

3-IIII-Br

C	-2.93446	-1.10859	0.00015
C	-2.52243	0.21846	0.00015
C	-1.16116	0.50593	0.
C	-0.29395	-0.55644	-0.00016
C	-0.62935	-1.88835	-0.00026
C	-2.00137	-2.14559	-0.00009
H	-3.99148	-1.34315	0.0003
H	-3.22289	1.04544	0.00026
H	0.12474	-2.66268	-0.00066
H	-2.33707	-3.17535	-0.00015
C	-0.59322	1.89656	-0.00007
O	0.73007	1.89152	-0.00025
O	-1.26974	2.88566	0.00007
Br	2.14787	-1.78352	0.00019
I	1.57797	0.06966	0.00005

Sum of electronic and zero-point Energies=-3291.296593
Sum of electronic and thermal Energies= -3291.286685
Sum of electronic and thermal Enthalpies= -3291.285741
Sum of electronic and thermal Free Energies= -3291.334589

3-IIII-CCH

C	-2.63836	2.37145	-0.00017
C	-2.96026	1.02422	-0.00021
C	-1.94291	0.08138	-0.00015
C	-0.6403	0.52162	-0.00005
C	-0.27053	1.84604	-0.00002
C	-1.30744	2.77375	-0.00008
H	-3.42304	3.11482	-0.00022
H	-3.98224	0.66925	-0.00028
H	0.76272	2.15392	0.00006
H	-1.06056	3.82641	-0.00005
C	-2.19376	-1.39221	-0.00019
O	-1.06845	-2.08072	-0.00012
O	-3.29097	-1.87622	-0.00027
C	3.41985	1.17907	0.23987
H	4.27376	1.81274	0.35904
C	2.46124	0.4677	0.10608
I	0.69628	-1.09812	0.00003

Sum of electronic and zero-point Energies= -793.780546
Sum of electronic and thermal Energies= -793.769816
Sum of electronic and thermal Enthalpies=-793.768872
Sum of electronic and thermal Free Energies= -793.818344

3-IIII-CF3

C	-2.69011	2.28973	0.00005
C	-2.95457	0.92757	0.0001
C	-1.91235	-0.00045	0.00001
C	-0.64991	0.52412	-0.00006
C	-0.30538	1.85846	-0.00013
C	-1.37667	2.75141	-0.00009
H	-3.50769	2.99983	0.00013
H	-3.96181	0.52596	0.00018
H	0.70804	2.22727	-0.00024
H	-1.16527	3.81354	-0.00016
C	-2.19866	-1.51874	-0.00005
O	-1.13277	-2.20926	-0.00037
O	-3.35993	-1.86798	0.00015
C	2.40402	0.26982	0.00001
F	3.4394	-0.54682	0.00069
F	2.44547	1.03228	1.07774
F	2.44614	1.03133	-1.07837
I	0.77117	-0.94889	0.00007

Sum of electronic and zero-point Energies=-1054.720873
Sum of electronic and thermal Energies= -1054.708540
Sum of electronic and thermal Enthalpies= -1054.707596

Sum of electronic and thermal Free Energies= -1054.761392

3-IIII-CH3

C	-2.69011	2.28973	0.00005
C	-2.95457	0.92757	0.0001
C	-1.91235	-0.00045	0.00001
C	-0.64991	0.52412	-0.00006
C	-0.30538	1.85846	-0.00013
C	-1.37667	2.75141	-0.00009
H	-3.50769	2.99983	0.00013
H	-3.96181	0.52596	0.00018
H	0.70804	2.22727	-0.00024
H	-1.16527	3.81354	-0.00016
C	-2.19866	-1.51874	-0.00005
O	-1.13277	-2.20926	-0.00037
O	-3.35993	-1.86798	0.00015
C	2.40402	0.26982	0.00001
H	2.4376	0.8875	0.87308
H	3.24415	-0.39281	0.00056
H	2.43814	0.88672	-0.87359
I	0.77117	-0.94889	0.00007

Sum of electronic and zero-point Energies= -756.933866
Sum of electronic and thermal Energies= -756.923698
Sum of electronic and thermal Enthalpies=-756.922754
Sum of electronic and thermal Free Energies= -756.970675

3-IIII-CHCH2

C	1.84919	2.09564	-0.00026
C	2.03211	0.72259	-0.00036
C	0.94595	-0.1438	-0.0002
C	-0.303	0.43	0.00006
C	-0.54379	1.79014	0.00034
C	0.56625	2.62624	0.00012
H	2.70392	2.75794	-0.00043
H	3.01223	0.26343	-0.00053
H	-1.5312	2.22037	0.00075
H	0.41394	3.69656	0.00029
C	1.13934	-1.67296	-0.00004
O	0.03732	-2.28217	-0.00078
O	2.28626	-2.08358	0.00075
C	-3.1018	0.35593	-0.00026
H	-3.09138	0.95627	-0.90317
C	-4.29648	-0.61585	-0.00019
H	-5.20975	-0.05832	-0.00042
H	-4.25478	-1.23241	-0.8737
I	-1.68034	-0.75405	0.00029

Sum of electronic and zero-point Energies= -795.002858
Sum of electronic and thermal Energies= -794.991741
Sum of electronic and thermal Enthalpies=-794.990797
Sum of electronic and thermal Free Energies= -795.041504

3-IIII-Cl

C	2.52236	2.11339	-0.00003
C	2.66085	0.73134	-0.00002
C	1.52519	-0.07221	-0.00001
C	0.30752	0.56194	0.00001
C	0.09823	1.91869	0.00008
C	1.25733	2.69731	0.00004
H	3.40253	2.74383	-0.00006
H	3.62846	0.24255	-0.00002
H	-0.89074	2.35437	0.00019
H	1.15606	3.77559	0.00007
C	1.58381	-1.57491	-0.00003
O	0.38663	-2.12239	-0.00002
O	2.61636	-2.18619	-0.00011
Cl	-2.94445	0.85661	-0.00013
I	-1.2452	-0.85194	0.00008

Sum of electronic and zero-point Energies=-1177.295720
Sum of electronic and thermal Energies= -1177.286063
Sum of electronic and thermal Enthalpies= -1177.285119
Sum of electronic and thermal Free Energies= -1177.332650

3-IIII-CN

C	2.38355	2.16507	-0.00011
C	2.54579	0.78572	-0.00018
C	1.42977	-0.04976	-0.00014
C	0.21531	0.57735	0.00019
C	-0.03278	1.92761	0.00009
C	1.11008	2.72995	0.00012
H	3.25324	2.81011	-0.00022
H	3.52047	0.31084	-0.00021
H	-1.02602	2.35531	0.00032
H	0.98659	3.8058	0.00018
C	1.54327	-1.57372	0.00002
O	0.38892	-2.14584	0.00036
O	2.63468	-2.0876	0.00015
C	-2.68934	0.59238	0.00006
N	-3.53721	1.37008	0.00019
I	-1.2004	-0.88851	-0.00018

Sum of electronic and zero-point Energies= -809.892050
Sum of electronic and thermal Energies= -809.881546
Sum of electronic and thermal Enthalpies=-809.880601
Sum of electronic and thermal Free Energies= -809.929731

3-IIII-F

C	-2.93446	-1.10859	0.00015
C	-2.52243	0.21846	0.00015
C	-1.16116	0.50593	0.
C	-0.29395	-0.55644	-0.00016
C	-0.62935	-1.88835	-0.00026
C	-2.00137	-2.14559	-0.00009
H	-3.99148	-1.34315	0.0003
H	-3.22289	1.04544	0.00026
H	0.12474	-2.66268	-0.00066
H	-2.33707	-3.17535	-0.00015
C	-0.59322	1.89656	-0.00007
O	0.73007	1.89152	-0.00025
O	-1.26974	2.88566	0.00007
F	2.14787	-1.78352	0.00019
I	1.69761	0.10968	0.00006

Sum of electronic and zero-point Energies= -816.933605
Sum of electronic and thermal Energies= -816.924437
Sum of electronic and thermal Enthalpies=-816.923493
Sum of electronic and thermal Free Energies= -816.969408

3-IIII-N3

C	-2.93446	-1.10859	0.00015
C	-2.52243	0.21846	0.00015
C	-1.16116	0.50593	0.
C	-0.29395	-0.55644	-0.00016
C	-0.62935	-1.88835	-0.00026
C	-2.00137	-2.14559	-0.00009
H	-3.99148	-1.34315	0.0003
H	-3.22289	1.04544	0.00026
H	0.12474	-2.66268	-0.00066
H	-2.33707	-3.17535	-0.00015
C	-0.59322	1.89656	-0.00007
O	0.73007	1.89152	-0.00025
O	-1.26974	2.88566	0.00007
N	2.11882	-1.68906	0.00018
N	3.3197	-1.96422	0.00031
N	3.73122	-3.30238	0.00041
I	1.57797	0.06966	0.00005

Sum of electronic and zero-point Energies= -881.251900
Sum of electronic and thermal Energies= -881.240646
Sum of electronic and thermal Enthalpies=-881.239702
Sum of electronic and thermal Free Energies= -881.291526

3-IIII-NH2

C	-2.63836	2.37145	-0.00017
C	-2.96026	1.02422	-0.00021
C	-1.94291	0.08138	-0.00015
C	-0.6403	0.52162	-0.00005
C	-0.27053	1.84604	-0.00002
C	-1.30744	2.77375	-0.00008
H	-3.42304	3.11482	-0.00022
H	-3.98224	0.66925	-0.00028
H	0.76272	2.15392	0.00006
H	-1.06056	3.82641	-0.00005
C	-2.19376	-1.39221	-0.00019
O	-1.06845	-2.08072	-0.00012
O	-3.29097	-1.87622	-0.00027
N	2.2254	0.26686	0.11023
H	2.63529	0.67481	0.92605
H	2.62113	0.68613	-0.70684
I	0.69628	-1.09812	0.00003

Sum of electronic and zero-point Energies= -772.993779
Sum of electronic and thermal Energies= -772.983910
Sum of electronic and thermal Enthalpies=-772.982966
Sum of electronic and thermal Free Energies= -773.030088

3-IIII-NHAc

C	-2.96551	2.1948	0.00012
C	-3.14135	0.82115	0.00005
C	-2.02605	-0.00366	-0.00001
C	-0.77557	0.56842	0.00001
C	-0.55317	1.92978	0.00009
C	-1.68457	2.73807	0.00014
H	-3.82524	2.85	0.00016
H	-4.11978	0.3599	0.00003
H	0.44096	2.34251	0.00011
H	-1.55512	3.81166	0.0002
C	-2.1044	-1.49596	-0.00007
O	-0.90086	-2.04357	-0.00014
O	-3.13436	-2.1089	-0.00015
C	3.15326	0.18449	-0.00018
O	3.38959	-0.99771	-0.00038
C	4.2019	1.26499	0.0006
H	4.07476	1.89669	-0.87709
H	4.0784	1.89115	0.88282
H	5.18679	0.80999	-0.00262
N	1.91064	0.67979	-0.00004
H	1.90346	1.56189	0.47096
I	0.72137	-0.90438	-0.00008

Sum of electronic and zero-point Energies= -925.619292
Sum of electronic and thermal Energies= -925.605707

Sum of electronic and thermal Enthalpies=-925.604763
Sum of electronic and thermal Free Energies= -925.662107

3-IIII-OCF3

C	2.67388	2.50582	-0.08055
C	3.07538	1.1861	-0.2436
C	2.13864	0.16777	-0.09614
C	0.85091	0.5348	0.20224
C	0.38735	1.81389	0.38416
C	1.35008	2.81324	0.23105
H	3.39497	3.30541	-0.19349
H	4.09694	0.91345	-0.48175
H	-0.64249	2.02395	0.63
H	1.04878	3.8455	0.35987
C	2.46298	-1.28967	-0.23514
O	1.40068	-2.06043	-0.03196
O	3.55405	-1.70721	-0.49333
F	-3.25665	-0.89735	-0.63674
F	-2.19308	0.86999	-1.26105
F	-3.78459	1.02948	0.19587
C	-2.75499	0.26941	-0.18023
O	-1.90316	0.11538	0.81056
I	-0.3585	-1.17318	0.37575

Sum of electronic and zero-point Energies=-1129.982632
Sum of electronic and thermal Energies= -1129.969662
Sum of electronic and thermal Enthalpies= -1129.968717
Sum of electronic and thermal Free Energies= -1130.024828

3-IIII-OCH3

C	2.67388	2.50582	-0.08055
C	3.07538	1.1861	-0.2436
C	2.13864	0.16777	-0.09614
C	0.85091	0.5348	0.20224
C	0.38735	1.81389	0.38416
C	1.35008	2.81324	0.23105
H	3.39497	3.30541	-0.19349
H	4.09694	0.91345	-0.48175
H	-0.64249	2.02395	0.63
H	1.04878	3.8455	0.35987
C	2.46298	-1.28967	-0.23514
O	1.40068	-2.06043	-0.03196
O	3.55405	-1.70721	-0.49333
C	-2.75499	0.26941	-0.18023
O	-1.90316	0.11538	0.81056
H	-3.5809	0.87912	0.12146
H	-2.3123	0.74256	-1.03173
H	-3.15272	-0.65563	-0.54216
I	-0.3585	-1.17318	0.37575

Sum of electronic and zero-point Energies= -832.150413
Sum of electronic and thermal Energies= -832.139201
Sum of electronic and thermal Enthalpies=-832.138257
Sum of electronic and thermal Free Energies= -832.188792

3-IIII-OCOCF3

C	3.22289	2.62188	0.00859
C	3.68898	1.31361	0.00651
C	2.77394	0.26568	0.00105
C	1.43871	0.58809	-0.00237
C	0.91134	1.85792	-0.00002
C	1.85448	2.88734	0.0057
H	3.92673	3.44448	0.01292
H	4.74543	1.07154	0.00947
H	-0.15229	2.04153	-0.00164
H	1.50103	3.91113	0.00798
C	3.17219	-1.17944	0.00108
O	2.11919	-1.99141	-0.00185
O	4.30614	-1.55945	0.00406
C	-2.41859	-0.53165	0.0117
O	-2.5234	-1.72768	0.04512
O	-1.32746	0.16971	-0.02174
C	-3.65945	0.39701	0.00003
F	-4.78188	-0.30037	0.05516
F	-3.67375	1.13597	-1.11316
F	-3.62623	1.23022	1.0443
I	0.2862	-1.16738	-0.00904

Sum of electronic and zero-point Energies=-1243.303932
Sum of electronic and thermal Energies= -1243.288818
Sum of electronic and thermal Enthalpies= -1243.287874
Sum of electronic and thermal Free Energies= -1243.350361

3-IIII-OCOCH3

C	2.94595	2.24262	0.
C	3.14905	0.86891	-0.00023
C	2.05101	0.01452	-0.00014
C	0.80079	0.58421	0.00015
C	0.53351	1.93493	0.00041
C	1.65414	2.76631	0.00033
H	3.79558	2.9138	-0.00007
H	4.13833	0.42604	-0.00045
H	-0.47532	2.31781	0.00073
H	1.50439	3.83907	0.00055
C	2.175	-1.48618	-0.00024
O	1.00226	-2.08015	0.
O	3.23627	-2.04801	-0.00049
C	-3.20495	0.1778	0.00021
O	-3.42588	-1.00956	0.00076
O	-1.9799	0.68529	0.00005
C	-4.26655	1.25034	-0.00099
H	-4.14463	1.88403	0.87785
H	-4.15011	1.8754	-0.88681
H	-5.24737	0.78154	0.00398
I	-0.68643	-0.89842	0.00013

Sum of electronic and zero-point Energies= -945.516278
Sum of electronic and thermal Energies= -945.503061
Sum of electronic and thermal Enthalpies=-945.502117
Sum of electronic and thermal Free Energies= -945.558323

3-IIII-OCOPh

C	-2.96551	2.1948	0.00012
C	-3.14135	0.82115	0.00005
C	-2.02605	-0.00366	-0.00001
C	-0.77557	0.56842	0.00001
C	-0.55317	1.92978	0.00009
C	-1.68457	2.73807	0.00014
H	-3.82524	2.85	0.00016
H	-4.11978	0.3599	0.00003
H	0.44096	2.34251	0.00011
H	-1.55512	3.81166	0.0002
C	-2.1044	-1.49596	-0.00007
O	-0.90086	-2.04357	-0.00014
O	-3.13436	-2.1089	-0.00015
C	3.15326	0.18449	-0.00018
O	3.38959	-0.99771	-0.0023
O	1.91808	0.67682	0.00235
C	4.22579	1.2896	0.00062
C	5.57865	0.94865	-0.00127
C	3.84477	2.63138	0.00313
C	6.55019	1.94931	0.00003
H	5.87872	-0.10927	-0.00246
C	4.81648	3.63247	0.00343
H	2.77855	2.90028	0.00444
C	6.16903	3.29167	0.00202
H	7.61655	1.68065	-0.00082
H	4.51576	4.69032	0.00499
H	6.93524	4.08049	0.00299
I	0.72137	-0.90438	-0.00008

Sum of electronic and zero-point Energies=-1137.194396
Sum of electronic and thermal Energies= -1137.178365
Sum of electronic and thermal Enthalpies= -1137.177421
Sum of electronic and thermal Free Energies= -1137.241196

3-IIII-OH

C	2.85263	-1.32247	0.00438
C	2.53897	0.03132	0.02066
C	1.20519	0.42867	0.01343
C	0.26118	-0.56546	0.00447
C	0.49803	-1.91871	-0.02637
C	1.8446	-2.28523	-0.02237
H	3.88939	-1.63508	0.00637
H	3.29795	0.80511	0.03341
H	-0.31078	-2.63443	-0.06185
H	2.09873	-3.33788	-0.04592
C	0.76361	1.8772	0.00979
O	-0.53232	1.99106	-0.02484
O	1.56652	2.77539	0.03319
O	-2.35941	-1.6203	-0.02187
H	-2.69235	-1.77691	0.86934
I	-1.68965	0.21143	-0.0219

Sum of electronic and zero-point Energies= -792.887433
Sum of electronic and thermal Energies= -792.877802
Sum of electronic and thermal Enthalpies=-792.876858
Sum of electronic and thermal Free Energies= -792.923492

3-IIII-OTf

C	-2.93446	-1.10859	-0.00888
C	-2.52242	0.21846	-0.00736
C	-1.16116	0.50593	-0.00483
C	-0.29394	-0.55644	-0.00398
C	-0.62934	-1.88835	-0.00546
C	-2.00136	-2.14559	-0.00799
H	-3.99147	-1.34315	-0.01083
H	-3.22288	1.04544	-0.00808
H	0.12474	-2.66268	-0.00491
H	-2.33706	-3.17535	-0.00925
C	-0.59322	1.89656	-0.00305
O	0.73007	1.89152	-0.00078
O	-1.26973	2.88566	-0.00361
O	2.10706	-1.65082	0.00018
S	3.59307	-1.98174	0.66431
O	3.33018	-2.88233	1.67519
O	4.05421	-0.70398	0.94073
C	4.38339	-2.66789	-0.57196
F	4.63907	-1.73642	-1.51509
F	5.54654	-3.19005	-0.12819
F	3.62322	-3.64906	-1.10295
I	1.57797	0.06966	0.00005

Sum of electronic and zero-point Energies=-1678.583631
Sum of electronic and thermal Energies= -1678.567340
Sum of electronic and thermal Enthalpies= -1678.566396
Sum of electronic and thermal Free Energies= -1678.631124

3-IIII-OTs

C	-2.93446	-1.10859	-0.00888
C	-2.52242	0.21846	-0.00736
C	-1.16116	0.50593	-0.00483
C	-0.29394	-0.55644	-0.00398
C	-0.62934	-1.88835	-0.00546
C	-2.00136	-2.14559	-0.00799
H	-3.99147	-1.34315	-0.01083
H	-3.22288	1.04544	-0.00808
H	0.12474	-2.66268	-0.00491
H	-2.33706	-3.17535	-0.00925
C	-0.59322	1.89656	-0.00305
O	0.73007	1.89152	-0.00078
O	-1.26973	2.88566	-0.00361
O	2.10706	-1.65082	0.00018
S	3.59307	-1.98174	0.66431
O	3.33018	-2.88233	1.67519
O	4.05421	-0.70398	0.94073
C	4.39807	-2.68064	-0.59493
C	4.28967	-4.05772	-0.84102
C	5.21363	-1.91787	-1.44488
C	4.96741	-4.65349	-1.89801
H	3.65893	-4.67381	-0.17439
C	5.89555	-2.50544	-2.5029
H	5.3178	-0.83348	-1.2578
C	5.77613	-3.88142	-2.74243
H	4.86999	-5.73576	-2.07304
H	6.53296	-1.88994	-3.15578
C	6.4851	-4.50936	-3.87989
H	7.41139	-3.94052	-4.14083
H	6.77023	-5.56379	-3.64125
H	5.81659	-4.52222	-4.77855
I	1.57797	0.06966	0.00005

Sum of electronic and zero-point Energies=-1611.760876
Sum of electronic and thermal Energies= -1611.742524
Sum of electronic and thermal Enthalpies= -1611.741580
Sum of electronic and thermal Free Energies= -1611.811500

3-IIII-radical

C	-2.93446	-1.10859	0.00015
C	-2.52243	0.21846	0.00015
C	-1.16116	0.50593	0.
C	-0.29395	-0.55644	-0.00016
C	-0.62935	-1.88835	-0.00026
C	-2.00137	-2.14559	-0.00009
H	-3.99148	-1.34315	0.0003
H	-3.22289	1.04544	0.00026
H	0.12474	-2.66268	-0.00066
H	-2.33707	-3.17535	-0.00015
C	-0.59322	1.89656	-0.00007
O	0.73007	1.89152	-0.00025
O	-1.26974	2.88566	0.00007
I	1.69761	0.10968	0.00006

Sum of electronic and zero-point Energies= -717.072517
Sum of electronic and thermal Energies= -717.064243
Sum of electronic and thermal Enthalpies=-717.063299
Sum of electronic and thermal Free Energies= -717.108193

3-IIII-SCF3

C	2.78872	2.53347	-0.0787
C	3.18535	1.22372	-0.31382
C	2.27986	0.18067	-0.13941
C	1.0132	0.52339	0.25857
C	0.55417	1.79211	0.51366
C	1.48729	2.81374	0.3311
H	3.49368	3.34408	-0.21405
H	4.18916	0.96587	-0.63234
H	-0.45947	1.99472	0.82838
H	1.17977	3.83591	0.514
C	2.65512	-1.26809	-0.37758
O	1.66601	-2.0776	-0.15099
O	3.76604	-1.5693	-0.73478
F	-3.93551	0.92128	-0.45801
F	-2.96359	-0.85487	-1.19914
F	-1.93008	1.02411	-1.25123
C	-2.77223	0.27822	-0.52401
S	-2.16147	-0.00054	1.15757
I	-0.18758	-1.1862	0.47172

Sum of electronic and zero-point Energies=-1452.936825
Sum of electronic and thermal Energies= -1452.923072
Sum of electronic and thermal Enthalpies= -1452.922127
Sum of electronic and thermal Free Energies= -1452.980196

4-BrIII-Br

C	-2.93446	-1.10859	0.00015
C	-2.52243	0.21846	0.00015
C	-1.16116	0.50593	0.
C	-0.29395	-0.55644	-0.00016
C	-0.62935	-1.88835	-0.00026
C	-2.00137	-2.14559	-0.00009
H	-3.99148	-1.34315	0.0003
H	-3.22289	1.04544	0.00026
H	0.12474	-2.66268	-0.00066
H	-2.33707	-3.17535	-0.00015
Br	1.57797	0.06966	0.00005
O	0.73007	1.89152	-0.00025
S	-0.59322	1.89656	-0.00007
O	-0.92751	2.51416	-1.20645
O	-0.92738	2.51405	1.20642
Br	2.14787	-1.78352	0.00019

Sum of electronic and zero-point Energies=-6003.092843
Sum of electronic and thermal Energies= -6003.082534
Sum of electronic and thermal Enthalpies= -6003.081590
Sum of electronic and thermal Free Energies= -6003.130621

4-BrIII-CCH

C	-1.73448	3.03455	-0.00008
C	-2.34105	1.79113	-0.0001
C	-1.55996	0.64271	-0.00004
C	-0.19406	0.7816	0.00004
C	0.45064	1.99943	0.00006
C	-0.34916	3.13487	-0.00001
H	-2.34077	3.92923	-0.00013
H	-3.41797	1.68502	-0.00015
H	1.52496	2.08236	0.00012
H	0.12713	4.10519	0.00001
Br	0.79291	-0.92176	0.00011
O	-1.08176	-1.82188	0.00026
S	-2.33535	-0.95715	-0.00008
O	-3.05502	-1.07363	1.22755
O	-3.05449	-1.0738	-1.228
C	2.56364	-0.20579	0.00002
C	3.67725	0.24449	-0.00003
H	4.66923	0.64558	-0.00008

Sum of electronic and zero-point Energies=-3505.595811
Sum of electronic and thermal Energies= -3505.583858
Sum of electronic and thermal Enthalpies= -3505.582913
Sum of electronic and thermal Free Energies= -3505.635075

4-BrIII-CF3

C	-2.69011	2.28973	0.00005
C	-2.95457	0.92757	0.0001
C	-1.91235	-0.00045	0.00001
C	-0.64991	0.52412	-0.00006
C	-0.30538	1.85846	-0.00013
C	-1.37667	2.75141	-0.00009
H	-3.50769	2.99983	0.00013
H	-3.96181	0.52596	0.00018
H	0.70804	2.22727	-0.00024
H	-1.16527	3.81354	-0.00016
Br	0.77117	-0.94889	0.00007
O	-1.13277	-2.20926	-0.00037
C	2.40402	0.26982	0.00001
F	3.4394	-0.54682	0.00069
F	2.44547	1.03228	1.07774
F	2.44614	1.03133	-1.07837
S	-2.19866	-1.51874	-0.00005
O	-2.79729	-1.88537	1.20651
O	-2.79782	-1.88533	-1.20636

Sum of electronic and zero-point Energies=-3766.543480
Sum of electronic and thermal Energies= -3766.529970
Sum of electronic and thermal Enthalpies= -3766.529026
Sum of electronic and thermal Free Energies= -3766.585303

4-BrIII-CH3

C	-2.69011	2.28973	0.00005
C	-2.95457	0.92757	0.0001
C	-1.91235	-0.00045	0.00001
C	-0.64991	0.52412	-0.00006
C	-0.30538	1.85846	-0.00013
C	-1.37667	2.75141	-0.00009
H	-3.50769	2.99983	0.00013
H	-3.96181	0.52596	0.00018
H	0.70804	2.22727	-0.00024
H	-1.16527	3.81354	-0.00016
Br	0.77117	-0.94889	0.00007
O	-1.13277	-2.20926	-0.00037
C	2.40402	0.26982	0.00001
S	-2.19866	-1.51874	-0.00005
O	-2.79729	-1.88537	1.20651
O	-2.79782	-1.88533	-1.20636
H	2.4376	0.8875	0.87308
H	3.24415	-0.39281	0.00056
H	2.43814	0.88672	-0.87359

Sum of electronic and zero-point Energies=-3468.766222
Sum of electronic and thermal Energies= -3468.754711
Sum of electronic and thermal Enthalpies= -3468.753766
Sum of electronic and thermal Free Energies= -3468.804764

4-BrIII-CHCH2

C	-1.06689	3.03816	0.03847

C	-1.68012	1.79376	0.06931
C	-0.91811	0.63558	0.01112
C	0.44442	0.78647	-0.08177
C	1.10103	1.99756	-0.12932
C	0.3136	3.14155	-0.06095
H	-1.66928	3.93489	0.08284
H	-2.75511	1.68537	0.12775
H	2.17244	2.07977	-0.22947
H	0.79033	4.11092	-0.09894
Br	1.47186	-0.87676	-0.18227
O	-0.82187	-1.69238	-0.93556
C	3.15698	-0.20687	0.53153
S	-1.65708	-1.01278	0.06058
O	-1.3946	-1.44641	1.41061
O	-3.02741	-0.82108	-0.31319
H	3.65477	0.38383	-0.22751
C	3.95896	-1.47933	0.86209
H	4.92873	-1.20618	1.22243
H	3.44406	-2.0402	1.6139

Sum of electronic and zero-point Energies=-3506.838271
Sum of electronic and thermal Energies= -3506.826077
Sum of electronic and thermal Enthalpies= -3506.825133
Sum of electronic and thermal Free Energies= -3506.877527

4-BrIII-Cl

C	2.52236	2.11339	-0.00003
C	2.66085	0.73134	-0.00002
C	1.52519	-0.07221	-0.00001
C	0.30752	0.56194	0.00001
C	0.09823	1.91869	0.00008
C	1.25733	2.69731	0.00004
H	3.40253	2.74383	-0.00006
H	3.62846	0.24255	-0.00002
H	-0.89074	2.35437	0.00019
H	1.15606	3.77559	0.00007
Br	-1.17132	-0.78467	0.00008
O	0.38663	-2.12239	-0.00002
Cl	-2.94445	0.85661	-0.00013
S	1.58381	-1.57491	-0.00003
O	2.14123	-2.00188	-1.20648
O	2.14415	-1.99815	1.20639

Sum of electronic and zero-point Energies=-3889.090319
Sum of electronic and thermal Energies= -3889.080284
Sum of electronic and thermal Enthalpies= -3889.079340
Sum of electronic and thermal Free Energies= -3889.127080

4-BrIII-CN

C	2.38355	2.16507	-0.00011
C	2.54579	0.78572	-0.00018
C	1.42977	-0.04976	-0.00014
C	0.21531	0.57735	0.00019
C	-0.03278	1.92761	0.00009
C	1.11008	2.72995	0.00012
H	3.25324	2.81011	-0.00022
H	3.52047	0.31084	-0.00021
H	-1.02602	2.35531	0.00032
H	0.98659	3.8058	0.00018
Br	-1.29133	-0.79808	-0.00016
O	0.38892	-2.14584	0.00036
C	-2.68934	0.59238	0.00006
N	-3.53721	1.37008	0.00019
S	1.54327	-1.57372	0.00002
O	2.10462	-1.99531	-1.20651
O	2.10502	-1.99529	1.20637

Sum of electronic and zero-point Energies=-3521.697812
Sum of electronic and thermal Energies= -3521.686163
Sum of electronic and thermal Enthalpies= -3521.685219
Sum of electronic and thermal Free Energies= -3521.736751

4-BrIII-F

C	-2.93446	-1.10859	0.00015
C	-2.52243	0.21846	0.00015
C	-1.16116	0.50593	0.
C	-0.29395	-0.55644	-0.00016
C	-0.62935	-1.88835	-0.00026
C	-2.00137	-2.14559	-0.00009
H	-3.99148	-1.34315	0.0003
H	-3.22289	1.04544	0.00026
H	0.12474	-2.66268	-0.00066
H	-2.33707	-3.17535	-0.00015
Br	1.57797	0.06966	0.00005
O	0.73007	1.89152	-0.00025
F	2.14787	-1.78352	0.00019
S	-0.59322	1.89656	-0.00007
O	-0.92751	2.51416	-1.20645
O	-0.92738	2.51405	1.20642

Sum of electronic and zero-point Energies=-3528.723548
Sum of electronic and thermal Energies= -3528.714075
Sum of electronic and thermal Enthalpies= -3528.713131
Sum of electronic and thermal Free Energies= -3528.759238

4-BrIII-N3

C	-2.93446	-1.10859	0.00015
C	-2.52243	0.21846	0.00015
C	-1.16116	0.50593	0.
C	-0.29395	-0.55644	-0.00016
C	-0.62935	-1.88835	-0.00026
C	-2.00137	-2.14559	-0.00009
H	-3.99148	-1.34315	0.0003
H	-3.22289	1.04544	0.00026
H	0.12474	-2.66268	-0.00066
H	-2.33707	-3.17535	-0.00015
Br	1.57797	0.06966	0.00005
O	0.73007	1.89152	-0.00025
S	-0.59322	1.89656	-0.00007
O	-0.92751	2.51416	-1.20645
O	-0.92738	2.51405	1.20642
N	2.11882	-1.68906	0.00018
N	2.60112	-3.93983	-0.89211
N	2.35359	-2.93701	-0.53779

Sum of electronic and zero-point Energies=-3593.055170
Sum of electronic and thermal Energies= -3593.042783
Sum of electronic and thermal Enthalpies= -3593.041839
Sum of electronic and thermal Free Energies= -3593.095853

4-BrIII-NH2

C	-1.73448	3.03455	-0.00008
C	-2.34105	1.79113	-0.0001
C	-1.55996	0.64271	-0.00004
C	-0.19406	0.7816	0.00004
C	0.45064	1.99943	0.00006
C	-0.34916	3.13487	-0.00001
H	-2.34077	3.92923	-0.00013
H	-3.41797	1.68502	-0.00015
H	1.52496	2.08236	0.00012
H	0.12713	4.10519	0.00001
Br	0.79291	-0.92176	0.00011
O	-1.08176	-1.82188	0.00026
S	-2.33535	-0.95715	-0.00008
O	-3.05502	-1.07363	1.22755
O	-3.05449	-1.0738	-1.228
N	2.49874	-0.23203	0.00003
H	2.98449	-0.54405	0.81654
H	2.98446	-0.54417	-0.81646

Sum of electronic and zero-point Energies=-3484.807901
Sum of electronic and thermal Energies= -3484.796754
Sum of electronic and thermal Enthalpies= -3484.795810
Sum of electronic and thermal Free Energies= -3484.845751

4-BrIII-NHAc

C	-2.17513	2.89633	-0.00006
C	-2.61484	1.58466	-0.00008
C	-1.68624	0.5528	-0.00008
C	-0.34787	0.86082	-0.00002
C	0.12958	2.15803	-0.00002
C	-0.81404	3.17627	-0.00003
H	-2.89259	3.70464	-0.00007
H	-3.66855	1.33932	-0.00009
H	1.18347	2.37431	-0.00002
H	-0.47135	4.20156	-0.00002
Br	0.83503	-0.6835	-0.0001
O	-0.84815	-1.80657	0.00013
C	3.48374	-0.17056	-0.00013
O	3.5246	-1.37015	-0.00022
C	4.67381	0.74661	0.00039
H	4.6399	1.38871	-0.87819
H	4.6425	1.38351	0.88288
H	5.58034	0.15093	-0.00251
S	-2.22312	-1.13898	0.00009
O	-2.91279	-1.37052	-1.22742
O	-2.91269	-1.37026	1.22771
N	2.33301	0.51156	-0.00017
H	2.46269	1.38405	0.47093

Sum of electronic and zero-point Energies=-3637.426202
Sum of electronic and thermal Energies= -3637.411456
Sum of electronic and thermal Enthalpies= -3637.410511
Sum of electronic and thermal Free Energies= -3637.469757

4-BrIII-OCF3

C	2.67388	2.50582	-0.08055
C	3.07538	1.1861	-0.2436
C	2.13864	0.16777	-0.09614
C	0.85091	0.5348	0.20224
C	0.38735	1.81389	0.38416
C	1.35008	2.81324	0.23105
H	3.39497	3.30541	-0.19349
H	4.09694	0.91345	-0.48175
H	-0.64249	2.02395	0.63
H	1.04878	3.8455	0.35987
Br	-0.29449	-1.08279	0.36657
O	1.40068	-2.06043	-0.03196
F	-3.25665	-0.89735	-0.63674
F	-2.19308	0.86999	-1.26105
F	-3.78459	1.02948	0.19587
C	-2.75499	0.26941	-0.18023
O	-1.90316	0.11538	0.81056

S	2.46298	-1.28967	-0.23514
O	3.33618	-1.65768	0.78988
O	2.81862	-1.53021	-1.56338

Sum of electronic and zero-point Energies=-3841.774713
Sum of electronic and thermal Energies= -3841.760677
Sum of electronic and thermal Enthalpies= -3841.759733
Sum of electronic and thermal Free Energies= -3841.817807

4-BrIII-OCH3

C	2.67388	2.50582	-0.08055
C	3.07538	1.1861	-0.2436
C	2.13864	0.16777	-0.09614
C	0.85091	0.5348	0.20224
C	0.38735	1.81389	0.38416
C	1.35008	2.81324	0.23105
H	3.39497	3.30541	-0.19349
H	4.09694	0.91345	-0.48175
H	-0.64249	2.02395	0.63
H	1.04878	3.8455	0.35987
Br	-0.29449	-1.08279	0.36657
O	1.40068	-2.06043	-0.03196
C	-2.75499	0.26941	-0.18023
O	-1.90316	0.11538	0.81056
S	2.46298	-1.28967	-0.23514
O	3.33618	-1.65768	0.78988
O	2.81862	-1.53021	-1.56338
H	-2.3123	0.74256	-1.03173
H	-3.5809	0.87912	0.12146
H	-3.15272	-0.65563	-0.54216

Sum of electronic and zero-point Energies=-3543.954954
Sum of electronic and thermal Energies= -3543.942735
Sum of electronic and thermal Enthalpies= -3543.941790
Sum of electronic and thermal Free Energies= -3543.994174

4-BrIII-OCOCF3

C	3.22289	2.62188	0.00859
C	3.68898	1.31361	0.00651
C	2.77394	0.26568	0.00105
C	1.43871	0.58809	-0.00237
C	0.91134	1.85792	-0.00002
C	1.85448	2.88734	0.0057
H	3.92673	3.44448	0.01292
H	4.74543	1.07154	0.00947
H	-0.15229	2.04153	-0.00164
H	1.50103	3.91113	0.00798
Br	0.34895	-1.0718	-0.00868
O	2.11919	-1.99141	-0.00185
C	-2.41859	-0.53165	0.0117
O	-2.5234	-1.72768	0.04512
O	-1.32746	0.16971	-0.02174
C	-3.65945	0.39701	0.00003
F	-4.78188	-0.30037	0.05516
F	-3.67375	1.13597	-1.11316
F	-3.62623	1.23022	1.0443
S	3.17219	-1.17944	0.00108
O	3.81228	-1.46262	1.20882
O	3.8193	-1.45784	-1.20404

Sum of electronic and zero-point Energies=-3955.094902
Sum of electronic and thermal Energies= -3955.078709
Sum of electronic and thermal Enthalpies= -3955.077765
Sum of electronic and thermal Free Energies= -3955.142201

4-BrIII-OCOCH3

C	2.94595	2.24262	0.
C	3.14905	0.86891	-0.00023
C	2.05101	0.01452	-0.00014
C	0.80079	0.58421	0.00015
C	0.53351	1.93493	0.00041
C	1.65414	2.76631	0.00033
H	3.79558	2.9138	-0.00007
H	4.13833	0.42604	-0.00045
H	-0.47532	2.31781	0.00073
H	1.50439	3.83907	0.00055
Br	-0.60316	-0.8154	0.00013
O	1.00226	-2.08015	0.
C	-3.20495	0.1778	0.00021
O	-3.42588	-1.00956	0.00076
O	-1.9799	0.68529	0.00005
C	-4.26655	1.25034	-0.00099
H	-4.14463	1.88403	0.87785
H	-4.15011	1.8754	-0.88681
H	-5.24737	0.78154	0.00398
S	2.175	-1.48618	-0.00024
O	2.75067	-1.8887	1.20606
O	2.75026	-1.88842	-1.20682

Sum of electronic and zero-point Energies=-3657.308276
Sum of electronic and thermal Energies= -3657.294683
Sum of electronic and thermal Enthalpies= -3657.293739
Sum of electronic and thermal Free Energies= -3657.350253

4-BrIII-OCOPh

C	-2.17513	2.89633	-0.00006
C	-2.61484	1.58466	-0.00008
C	-1.68624	0.5528	-0.00008
C	-0.34787	0.86082	-0.00002
C	0.12958	2.15803	-0.00002
C	-0.81404	3.17627	-0.00003
H	-2.89259	3.70464	-0.00007
H	-3.66855	1.33932	-0.00009
H	1.18347	2.37431	-0.00002
H	-0.47135	4.20156	-0.00002
Br	0.83503	-0.6835	-0.0001
O	-0.84815	-1.80657	0.00013
C	3.48374	-0.17056	-0.00013
O	3.5246	-1.37015	-0.00211
O	2.32856	0.5142	0.00225
S	-2.22312	-1.13898	0.00009
O	-2.91279	-1.37052	-1.22742
O	-2.91269	-1.37026	1.22771
C	4.70352	0.76951	0.0004
C	5.99358	0.23827	-0.00183
C	4.51865	2.15203	0.00301
C	7.09845	1.08942	-0.00078
H	6.13899	-0.85173	-0.00311
C	5.62375	3.00359	0.00307
H	3.50195	2.57091	0.00459
C	6.91353	2.47254	0.00131
H	8.11532	0.67076	-0.00191
H	5.47768	4.0936	0.00471
H	7.78484	3.14345	0.00208

Sum of electronic and zero-point Energies=-3848.987599
Sum of electronic and thermal Energies= -3848.970464
Sum of electronic and thermal Enthalpies= -3848.969519
Sum of electronic and thermal Free Energies= -3849.035461

4-BrIII-OH

C	2.85263	-1.32247	0.00438
C	2.53897	0.03132	0.02066
C	1.20519	0.42867	0.01343
C	0.26118	-0.56546	0.00447
C	0.49803	-1.91871	-0.02637
C	1.8446	-2.28523	-0.02237
H	3.88939	-1.63508	0.00637
H	3.29795	0.80511	0.03341
H	-0.31078	-2.63443	-0.06185
H	2.09873	-3.33788	-0.04592
Br	-1.58476	0.16966	-0.02049
O	-0.53232	1.99106	-0.02484
O	-2.35941	-1.6203	-0.02187
H	-2.69235	-1.77691	0.86934
S	0.76361	1.8772	0.00979
O	1.15963	2.48527	-1.18269
O	1.12457	2.45173	1.2297

Sum of electronic and zero-point Energies=-3504.688699
Sum of electronic and thermal Energies= -3504.677972
Sum of electronic and thermal Enthalpies= -3504.677028
Sum of electronic and thermal Free Energies= -3504.725908

4-BrIII-OTf

C	-2.93446	-1.10859	-0.00888
C	-2.52242	0.21846	-0.00736
C	-1.16116	0.50593	-0.00483
C	-0.29394	-0.55644	-0.00398
C	-0.62934	-1.88835	-0.00546
C	-2.00136	-2.14559	-0.00799
H	-3.99147	-1.34315	-0.01083
H	-3.22288	1.04544	-0.00808
H	0.12474	-2.66268	-0.00491
H	-2.33706	-3.17535	-0.00925
Br	1.57797	0.06966	0.00005
O	0.73007	1.89152	-0.00078
S	-0.59322	1.89656	-0.00305
O	-0.92527	2.51485	-1.2097
O	-0.92961	2.51337	1.20317
O	2.10706	-1.65082	0.00018
S	3.59172	-1.98216	0.66714
O	3.32676	-2.88338	1.67691
O	4.05228	-0.70457	0.94529
C	4.38455	-2.66753	-0.56795
F	4.65878	-1.73163	-1.50143
F	5.53789	-3.20648	-0.11868
F	3.61776	-3.6357	-1.11309

Sum of electronic and zero-point Energies=-4390.372201
Sum of electronic and thermal Energies= -4390.354823
Sum of electronic and thermal Enthalpies= -4390.353879
Sum of electronic and thermal Free Energies= -4390.420472

4-BrIII-OTs

C	-2.93446	-1.10859	-0.00888
C	-2.52242	0.21846	-0.00736
C	-1.16116	0.50593	-0.00483
C	-0.29394	-0.55644	-0.00398
C	-0.62934	-1.88835	-0.00546
C	-2.00136	-2.14559	-0.00799
H	-3.99147	-1.34315	-0.01083
H	-3.22288	1.04544	-0.00808

H	0.12474	-2.66268	-0.00491
H	-2.33706	-3.17535	-0.00925
Br	1.57797	0.06966	0.00005
O	0.73007	1.89152	-0.00078
S	-0.59322	1.89656	-0.00305
O	-0.92527	2.51485	-1.2097
O	-0.92961	2.51337	1.20317
O	2.10706	-1.65082	0.00018
S	3.59172	-1.98216	0.66714
O	3.32676	-2.88338	1.67691
O	4.05228	-0.70457	0.94529
C	4.39928	-2.68027	-0.5909
C	4.29138	-4.05719	-0.83807
C	5.21657	-1.91696	-1.4387
C	4.97128	-4.65229	-1.89405
H	3.65929	-4.6737	-0.17312
C	5.90065	-2.50387	-2.4957
H	5.32036	-0.83269	-1.25073
C	5.78172	-3.87969	-2.73634
H	4.87422	-5.73446	-2.06996
H	6.53939	-1.88796	-3.14689
C	6.49302	-4.50693	-3.87273
H	7.41984	-3.93793	-4.13143
H	6.77766	-5.5615	-3.63417
H	5.82634	-4.51922	-4.77277

Sum of electronic and zero-point Energies=-4323.552641
Sum of electronic and thermal Energies= -4323.532260
Sum of electronic and thermal Enthalpies= -4323.531316
Sum of electronic and thermal Free Energies= -4323.606800

4-BrIII-radical

C	-2.93446	-1.10859	0.00015
C	-2.52243	0.21846	0.00015
C	-1.16116	0.50593	0.
C	-0.29395	-0.55644	-0.00016
C	-0.62935	-1.88835	-0.00026
C	-2.00137	-2.14559	-0.00009
H	-3.99148	-1.34315	0.0003
H	-3.22289	1.04544	0.00026
H	0.12474	-2.66268	-0.00066
H	-2.33707	-3.17535	-0.00015
Br	1.57797	0.06966	0.00005
O	0.73007	1.89152	-0.00025
S	-0.59322	1.89656	-0.00007
O	-0.92751	2.51416	-1.20645
O	-0.92738	2.51405	1.20642

Sum of electronic and zero-point Energies=-3428.893471
Sum of electronic and thermal Energies= -3428.884804
Sum of electronic and thermal Enthalpies= -3428.883860
Sum of electronic and thermal Free Energies= -3428.929139

4-BrIII-SCF3

C	2.78872	2.53347	-0.0787
C	3.18535	1.22372	-0.31382
C	2.27986	0.18067	-0.13941
C	1.0132	0.52339	0.25857
C	0.55417	1.79211	0.51366
C	1.48729	2.81374	0.3311
H	3.49368	3.34408	-0.21405
H	4.18916	0.96587	-0.63234
H	-0.45947	1.99472	0.82838
H	1.17977	3.83591	0.514
O	1.66601	-2.0776	-0.15099
F	-3.93551	0.92128	-0.45801
F	-2.96359	-0.85487	-1.19914
F	-1.93008	1.02411	-1.25123
C	-2.77223	0.27822	-0.52401
S	-2.16147	-0.00054	1.15757
Br	-0.14178	-1.121	0.46359
S	2.65512	-1.26809	-0.37758
O	3.60669	-1.65601	0.56719
O	2.91876	-1.44953	-1.73631

Sum of electronic and zero-point Energies=-4164.743474
Sum of electronic and thermal Energies= -4164.728449
Sum of electronic and thermal Enthalpies= -4164.727505
Sum of electronic and thermal Free Energies= -4164.788182

4-ClIII-Br

C	-2.93446	-1.10859	0.00015
C	-2.52243	0.21846	0.00015
C	-1.16116	0.50593	0.
C	-0.29395	-0.55644	-0.00016
C	-0.62935	-1.88835	-0.00026
C	-2.00137	-2.14559	-0.00009
H	-3.99148	-1.34315	0.0003
H	-3.22289	1.04544	0.00026
H	0.12474	-2.66268	-0.00066
H	-2.33707	-3.17535	-0.00015
O	0.73007	1.89152	-0.00025
S	-0.59322	1.89656	-0.00007
O	-0.92751	2.51416	-1.20645
O	-0.92738	2.51405	1.20642
Br	2.14787	-1.78352	0.00019
Cl	1.57797	0.06966	0.00005

Sum of electronic and zero-point Energies=-3889.067576
Sum of electronic and thermal Energies= -3889.056761
Sum of electronic and thermal Enthalpies= -3889.055816
Sum of electronic and thermal Free Energies= -3889.105922

4-ClIII-CCH

C	-1.73448	3.03455	-0.00008
C	-2.34105	1.79113	-0.0001
C	-1.55996	0.64271	-0.00004
C	-0.19406	0.7816	0.00004
C	0.45064	1.99943	0.00006
C	-0.34916	3.13487	-0.00001
H	-2.34077	3.92923	-0.00013
H	-3.41797	1.68502	-0.00015
H	1.52496	2.08236	0.00012
H	0.12713	4.10519	0.00001
O	-1.08176	-1.82188	0.00026
S	-2.33535	-0.95715	-0.00008
O	-3.05502	-1.07363	1.22755
O	-3.05449	-1.0738	-1.228
C	2.56364	-0.20579	0.00002
C	3.67725	0.24449	-0.00003
H	4.66923	0.64558	-0.00008
Cl	0.79291	-0.92176	0.00011

Sum of electronic and zero-point Energies=-1391.584552
Sum of electronic and thermal Energies= -1391.572754
Sum of electronic and thermal Enthalpies= -1391.571810
Sum of electronic and thermal Free Energies= -1391.623380

4-ClIII-CF3

C	-2.69011	2.28973	0.00005
C	-2.95457	0.92757	0.0001
C	-1.91235	-0.00045	0.00001
C	-0.64991	0.52412	-0.00006
C	-0.30538	1.85846	-0.00013
C	-1.37667	2.75141	-0.00009
H	-3.50769	2.99983	0.00013
H	-3.96181	0.52596	0.00018
H	0.70804	2.22727	-0.00024
H	-1.16527	3.81354	-0.00016
O	-1.13277	-2.20926	-0.00037
C	2.40402	0.26982	0.00001
F	3.4394	-0.54682	0.00069
F	2.44547	1.03228	1.07774
F	2.44614	1.03133	-1.07837
S	-2.19866	-1.51874	-0.00005
O	-2.79729	-1.88537	1.20651
O	-2.79782	-1.88533	-1.20636
Cl	0.77117	-0.94889	0.00007

Sum of electronic and zero-point Energies=-1652.541757
Sum of electronic and thermal Energies= -1652.528609
Sum of electronic and thermal Enthalpies= -1652.527665
Sum of electronic and thermal Free Energies= -1652.582567

4-ClIII-CH3

C	-2.69011	2.28973	0.00005
C	-2.95457	0.92757	0.0001
C	-1.91235	-0.00045	0.00001
C	-0.64991	0.52412	-0.00006
C	-0.30538	1.85846	-0.00013
C	-1.37667	2.75141	-0.00009
H	-3.50769	2.99983	0.00013
H	-3.96181	0.52596	0.00018
H	0.70804	2.22727	-0.00024
H	-1.16527	3.81354	-0.00016
O	-1.13277	-2.20926	-0.00037
C	2.40402	0.26982	0.00001
S	-2.19866	-1.51874	-0.00005
O	-2.79729	-1.88537	1.20651
O	-2.79782	-1.88533	-1.20636
H	2.4376	0.8875	0.87308
H	3.24415	-0.39281	0.00056
H	2.43814	0.88672	-0.87359
Cl	0.77117	-0.94889	0.00007

Sum of electronic and zero-point Energies=-1354.771942
Sum of electronic and thermal Energies= -1354.760680
Sum of electronic and thermal Enthalpies= -1354.759735
Sum of electronic and thermal Free Energies= -1354.809608

4-ClIII-CHCH2

C	-1.06689	3.03816	0.03847
C	-1.68012	1.79376	0.06931
C	-0.91811	0.63558	0.01112
C	0.44442	0.78647	-0.08177
C	1.10103	1.99756	-0.12932
C	0.3136	3.14155	-0.06095
H	-1.66928	3.93489	0.08284
H	-2.75511	1.68537	0.12775
H	2.17244	2.07977	-0.22947
H	0.79033	4.11092	-0.09894
O	-0.82187	-1.69238	-0.93556
C	3.15698	-0.20687	0.53153

S -1.65708 -1.01278 0.06058
O -1.3946 -1.44641 1.41061
O -3.02741 -0.82108 -0.31319
H 3.65477 0.38383 -0.22751
C 3.95896 -1.47933 0.86209
H 4.92873 -1.20618 1.22243
H 3.44406 -2.0402 1.6139
Cl 1.47186 -0.87676 -0.18227
Sum of electronic and zero-point Energies=-1392.841160
Sum of electronic and thermal Energies= -1392.829104
Sum of electronic and thermal Enthalpies= -1392.828160
Sum of electronic and thermal Free Energies= -1392.880259

4-ClIII-Cl
C 2.52236 2.11339 -0.00003
C 2.66085 0.73134 -0.00002
C 1.52519 -0.07221 -0.00001
C 0.30752 0.56194 0.00001
C 0.09823 1.91869 0.00008
C 1.25733 2.69731 0.00004
H 3.40253 2.74383 -0.00006
H 3.62846 0.24255 -0.00002
H -0.89074 2.35437 0.00019
H 1.15606 3.77559 0.00007
O 0.38663 -2.12239 -0.00002
Cl -2.94445 0.85661 -0.00013
S 1.58381 -1.57491 -0.00003
O 2.14123 -2.00188 -1.20648
O 2.14415 -1.99815 1.20639
Cl -0.99381 -0.62303 0.00007
Sum of electronic and zero-point Energies=-1775.063362
Sum of electronic and thermal Energies= -1775.052836
Sum of electronic and thermal Enthalpies= -1775.051891
Sum of electronic and thermal Free Energies= -1775.100582

4-ClIII-CN
C 2.38355 2.16507 -0.00011
C 2.54579 0.78572 -0.00018
C 1.42977 -0.04976 -0.00014
C 0.21531 0.57735 0.00019
C -0.03278 1.92761 0.00009
C 1.11008 2.72995 0.00012
H 3.25324 2.81011 -0.00022
H 3.52047 0.31084 -0.00021
H -1.02602 2.35531 0.00032
H 0.98659 3.8058 0.00018
O 0.38892 -2.14584 0.00036
C -2.68934 0.59238 0.00006
N -3.53721 1.37008 0.00019
S 1.54327 -1.57372 0.00002
O 2.10462 -1.99531 -1.20651
O 2.10502 -1.99529 1.20637
Cl -1.44146 -0.64875 -0.00014
Sum of electronic and zero-point Energies=-1407.680665
Sum of electronic and thermal Energies= -1407.669171
Sum of electronic and thermal Enthalpies= -1407.668226
Sum of electronic and thermal Free Energies= -1407.719106

4-ClIII-F
C -2.93446 -1.10859 0.00015
C -2.52243 0.21846 0.00015
C -1.16116 0.50593 0.
C -0.29395 -0.55644 -0.00016
C -0.62935 -1.88835 -0.00026
C -2.00137 -2.14559 -0.00009
H -3.99148 -1.34315 0.0003
H -3.22289 1.04544 0.00026
H 0.12474 -2.66268 -0.00066
H -2.33707 -3.17535 -0.00015
O 0.73007 1.89152 -0.00025
F 2.14787 -1.78352 0.00019
S -0.59322 1.89656 -0.00007
O -0.92751 2.51416 -1.20645
O -0.92738 2.51405 1.20642
Cl 1.37517 0.00183 0.00002
Sum of electronic and zero-point Energies=-1414.692881
Sum of electronic and thermal Energies= -1414.683772
Sum of electronic and thermal Enthalpies= -1414.682828
Sum of electronic and thermal Free Energies= -1414.727714

4-ClIII-N3
C -2.93446 -1.10859 0.00015
C -2.52243 0.21846 0.00015
C -1.16116 0.50593 0.
C -0.29395 -0.55644 -0.00016
C -0.62935 -1.88835 -0.00026
C -2.00137 -2.14559 -0.00009
H -3.99148 -1.34315 0.0003
H -3.22289 1.04544 0.00026
H 0.12474 -2.66268 -0.00066
H -2.33707 -3.17535 -0.00015
O 0.73007 1.89152 -0.00025
S -0.59322 1.89656 -0.00007
O -0.92751 2.51416 -1.20645
O -0.92738 2.51405 1.20642
N 2.11882 -1.68906 0.00018
N 2.60112 -3.93983 -0.89211
N 2.35359 -2.93701 -0.53779
Cl 1.57797 0.06966 0.00005
Sum of electronic and zero-point Energies=-1479.030992
Sum of electronic and thermal Energies= -1479.018811
Sum of electronic and thermal Enthalpies= -1479.017867
Sum of electronic and thermal Free Energies= -1479.071314

4-ClIII-NH2
C -1.73448 3.03455 -0.00008
C -2.34105 1.79113 -0.0001
C -1.55996 0.64271 -0.00004
C -0.19406 0.7816 0.00004
C 0.45064 1.99943 0.00006
C -0.34916 3.13487 -0.00001
H -2.34077 3.92923 -0.00013
H -3.41797 1.68502 -0.00015
H 1.52496 2.08236 0.00012
H 0.12713 4.10519 0.00001
O -1.08176 -1.82188 0.00026
S -2.33535 -0.95715 -0.00008
O -3.05502 -1.07363 1.22755
O -3.05449 -1.0738 -1.228
N 2.49874 -0.23203 0.00003
H 2.98449 -0.54405 0.81654
H 2.98446 -0.54417 -0.81646
Cl 0.79291 -0.92176 0.00011
Sum of electronic and zero-point Energies=-1370.790334
Sum of electronic and thermal Energies= -1370.780224
Sum of electronic and thermal Enthalpies= -1370.779280
Sum of electronic and thermal Free Energies= -1370.826155

4-ClIII-NHAc
C -2.17513 2.89633 -0.00006
C -2.61484 1.58466 -0.00008
C -1.68624 0.5528 -0.00008
C -0.34787 0.86082 -0.00002
C 0.12958 2.15803 -0.00002
C -0.81404 3.17627 -0.00003
H -2.89259 3.70464 -0.00007
H -3.66855 1.33932 -0.00009
H 1.18347 2.37431 -0.00002
H -0.47135 4.20156 -0.00002
O -0.84815 -1.80657 0.00013
C 3.48374 -0.17056 -0.00013
O 3.5246 -1.37015 -0.00022
C 4.67381 0.74661 0.00039
H 4.6399 1.38871 -0.87819
H 4.6425 1.38351 0.88288
H 5.58034 0.15093 -0.00251
S -2.22312 -1.13898 0.00009
O -2.91279 -1.37052 -1.22742
O -2.91269 -1.37026 1.22771
N 2.33301 0.51156 -0.00017
H 2.46269 1.38405 0.47093
Cl 0.83503 -0.6835 -0.0001
Sum of electronic and zero-point Energies=-1523.407690
Sum of electronic and thermal Energies= -1523.393089
Sum of electronic and thermal Enthalpies= -1523.392145
Sum of electronic and thermal Free Energies= -1523.450901

4-ClIII-OCF3
C 2.67388 2.50582 -0.08055
C 3.07538 1.1861 -0.2436
C 2.13864 0.16777 -0.09614
C 0.85091 0.5348 0.20224
C 0.38735 1.81389 0.38416
C 1.35008 2.81324 0.23105
H 3.39497 3.30541 -0.19349
H 4.09694 0.91345 -0.48175
H -0.64249 2.02395 0.63
H 1.04878 3.8455 0.35987
O 1.40068 -2.06043 -0.03196
F -3.25665 -0.89735 -0.63674
F -2.19308 0.86999 -1.26105
F -3.78459 1.02948 0.19587
C -2.75499 0.26941 -0.18023
O -1.90316 0.11538 0.81056
S 2.46298 -1.28967 -0.23514
O 3.33618 -1.65768 0.78988
O 2.81862 -1.53021 -1.56338
Cl -0.16269 -0.89665 0.34766
Sum of electronic and zero-point Energies=-1727.744675
Sum of electronic and thermal Energies= -1727.730970
Sum of electronic and thermal Enthalpies= -1727.730025
Sum of electronic and thermal Free Energies= -1727.787012

4-ClIII-OCH3
C 2.67388 2.50582 -0.08055
C 3.07538 1.1861 -0.2436

C	2.13864	0.16777	-0.09614
C	0.85091	0.5348	0.20224
C	0.38735	1.81389	0.38416
C	1.35008	2.81324	0.23105
H	3.39497	3.30541	-0.19349
H	4.09694	0.91345	-0.48175
H	-0.64249	2.02395	0.63
H	1.04878	3.8455	0.35987
O	1.40068	-2.06043	-0.03196
C	-2.75499	0.26941	-0.18023
O	-1.90316	0.11538	0.81056
S	2.46298	-1.28967	-0.23514
O	3.33618	-1.65768	0.78988
O	2.81862	-1.53021	-1.56338
H	-2.3123	0.74256	-1.03173
H	-3.5809	0.87912	0.12146
H	-3.15272	-0.65563	-0.54216
Cl	-0.16269	-0.89665	0.34766

Sum of electronic and zero-point Energies=-1429.932692
Sum of electronic and thermal Energies= -1429.920759
Sum of electronic and thermal Enthalpies= -1429.919815
Sum of electronic and thermal Free Energies= -1429.971268

4-ClIII-OCOCF3

C	3.22289	2.62188	0.00859
C	3.68898	1.31361	0.00651
C	2.77394	0.26568	0.00105
C	1.43871	0.58809	-0.00237
C	0.91134	1.85792	-0.00002
C	1.85448	2.88734	0.0057
H	3.92673	3.44448	0.01292
H	4.74543	1.07154	0.00947
H	-0.15229	2.04153	-0.00164
H	1.50103	3.91113	0.00798
O	2.11919	-1.99141	-0.00185
C	-2.41859	-0.53165	0.0117
O	-2.5234	-1.72768	0.04512
O	-1.32746	0.16971	-0.02174
C	-3.65945	0.39701	0.00003
F	-4.78188	-0.30037	0.05516
F	-3.67375	1.13597	-1.11316
F	-3.62623	1.23022	1.0443
S	3.17219	-1.17944	0.00108
O	3.81228	-1.46262	1.20882
O	3.8193	-1.45784	-1.20404
Cl	0.47279	-0.88316	-0.00796

Sum of electronic and zero-point Energies=-1841.064133
Sum of electronic and thermal Energies= -1841.048273
Sum of electronic and thermal Enthalpies= -1841.047329
Sum of electronic and thermal Free Energies= -1841.110547

4-ClIII-OCOCH3

C	2.69653	-1.05749	0.02251
C	2.36246	0.29711	0.10383
C	1.04319	0.66795	0.01692
C	0.06952	-0.29413	-0.15272
C	0.35913	-1.63378	-0.24548
C	1.69558	-2.01166	-0.15321
H	3.1369	1.04354	0.21598
H	-0.40864	-2.37523	-0.3842
H	1.93696	-3.0604	-0.22903
O	-0.79243	2.11626	-0.71585
C	-3.6377	-1.19055	0.0653
O	-4.24674	-0.19471	-0.19054
O	-2.28459	-1.24122	0.08333
C	-4.22389	-2.53103	0.40566
H	-3.88351	-3.27418	-0.3135
H	-3.88446	-2.8375	1.39353
H	-5.30583	-2.45493	0.38459
S	0.47092	2.34667	0.10609
O	1.39208	3.19226	-0.57747
O	0.17172	2.60268	1.47965
Cl	-1.60441	0.36457	-0.28222
H	3.64541	-1.26577	0.08837

Sum of electronic and zero-point Energies=-1543.280519
Sum of electronic and thermal Energies= -1543.266454
Sum of electronic and thermal Enthalpies= -1543.265510
Sum of electronic and thermal Free Energies= -1543.323279

4-ClIII-OCOPh

C	-2.17513	2.89633	-0.00006
C	-2.61484	1.58466	-0.00008
C	-1.68624	0.5528	-0.00008
C	-0.34787	0.86082	-0.00002
C	0.12958	2.15803	-0.00002
C	-0.81404	3.17627	-0.00003
H	-2.89259	3.70464	-0.00007
H	-3.66855	1.33932	-0.00009
H	1.18347	2.37431	-0.00002
H	-0.47135	4.20156	-0.00002
O	-0.84815	-1.80657	0.00013
C	3.48374	-0.17056	-0.00013
O	3.5246	-1.37015	-0.00211
O	2.32856	0.5142	0.00225
S	-2.22312	-1.13898	0.00009
O	-2.91279	-1.37052	-1.22742
O	-2.91269	-1.37026	1.22771
C	4.70352	0.76951	0.0004
C	5.99358	0.23827	-0.00183
C	4.51865	2.15203	0.00301
C	7.09845	1.08942	-0.00078
H	6.13899	-0.85173	-0.00311
C	5.62375	3.00359	0.00307
H	3.50195	2.57091	0.00459
C	6.91353	2.47254	0.00131
H	8.11532	0.67076	-0.00191
H	5.47768	4.0936	0.00471
H	7.78484	3.14345	0.00208
Cl	0.72236	-0.5364	-0.00009

Sum of electronic and zero-point Energies=-1734.957808
Sum of electronic and thermal Energies= -1734.940981
Sum of electronic and thermal Enthalpies= -1734.940037
Sum of electronic and thermal Free Energies= -1735.004922

4-ClIII-OH

C	2.85263	-1.32247	0.00438
C	2.53897	0.03132	0.02066
C	1.20519	0.42867	0.01343
C	0.26118	-0.56546	0.00447
C	0.49803	-1.91871	-0.02637
C	1.8446	-2.28523	-0.02237
H	3.88939	-1.63508	0.00637
H	3.29795	0.80511	0.03341
H	-0.31078	-2.63443	-0.06185
H	2.09873	-3.33788	-0.04592
O	-0.53232	1.99106	-0.02484
O	-2.35941	-1.6203	-0.02187
H	-2.69235	-1.77691	0.86934
S	0.76361	1.8772	0.00979
O	1.15963	2.48527	-1.18269
O	1.12457	2.45173	1.2297
Cl	-1.3738	0.08565	-0.01763

Sum of electronic and zero-point Energies=-1390.663571
Sum of electronic and thermal Energies= -1390.653141
Sum of electronic and thermal Enthalpies= -1390.652197
Sum of electronic and thermal Free Energies= -1390.700032

4-ClIII-OTf

C	-2.93446	-1.10859	-0.00888
C	-2.52242	0.21846	-0.00736
C	-1.16116	0.50593	-0.00483
C	-0.29394	-0.55644	-0.00398
C	-0.62934	-1.88835	-0.00546
C	-2.00136	-2.14559	-0.00799
H	-3.99147	-1.34315	-0.01083
H	-3.22288	1.04544	-0.00808
H	0.12474	-2.66268	-0.00491
H	-2.33706	-3.17535	-0.00925
O	0.73007	1.89152	-0.00078
S	-0.59322	1.89656	-0.00305
O	-0.92527	2.51485	-1.2097
O	-0.92961	2.51337	1.20317
O	2.10706	-1.65082	0.00018
S	3.59172	-1.98216	0.66714
O	3.32676	-2.88338	1.67691
O	4.05228	-0.70457	0.94529
C	4.38455	-2.66753	-0.56795
F	4.65878	-1.73163	-1.50143
F	5.53789	-3.20648	-0.11868
F	3.61776	-3.6357	-1.11309
Cl	1.57797	0.06966	0.00005

Sum of electronic and zero-point Energies=-2276.340970
Sum of electronic and thermal Energies= -2276.323855
Sum of electronic and thermal Enthalpies= -2276.322910
Sum of electronic and thermal Free Energies= -2276.389101

4-ClIII-OTs

C	4.38816	1.01612	0.85593
C	3.9155	-0.28667	0.77922
C	2.71266	-0.52225	0.13864
C	2.03096	0.54036	-0.39658
C	2.45568	1.84457	-0.34844
C	3.668	2.06424	0.29767
H	5.32919	1.21597	1.34851
H	4.46669	-1.12138	1.19026
H	1.88353	2.65283	-0.7743
H	4.04695	3.07455	0.35803
O	1.23836	-1.7411	-1.38612
S	1.94044	-2.10289	-0.07961
O	2.93749	-3.10086	-0.27359
O	0.99688	-2.25349	0.98741
O	-0.17928	1.91526	-1.03635
S	-1.14548	2.6577	0.03822
O	-0.4796	3.89271	0.26292
O	-1.38761	1.73038	1.09317
C	-2.62484	2.9028	-0.86491

C	-2.76452	4.05268	-1.63146
C	-3.61968	1.93408	-0.81436
C	-3.9265	4.22715	-2.36132
H	-1.97963	4.79634	-1.63594
C	-4.77026	2.12623	-1.56082
H	-3.49721	1.04729	-0.20649
C	-4.93928	3.26742	-2.34205
H	-4.05446	5.12409	-2.95358
H	-5.54824	1.37458	-1.53225
C	-6.17928	3.45213	-3.16846
H	-7.02626	2.92726	-2.73087
H	-6.43409	4.50606	-3.26658
H	-6.02514	3.05355	-4.17294
Cl	0.47506	0.07572	-1.21641

Sum of electronic and zero-point Energies=-2209.524975
Sum of electronic and thermal Energies= -2209.505072
Sum of electronic and thermal Enthalpies= -2209.504128
Sum of electronic and thermal Free Energies= -2209.576142

4-ClIII-radical

C	-2.93446	-1.10859	0.00015
C	-2.52243	0.21846	0.00015
C	-1.16116	0.50593	0.
C	-0.29395	-0.55644	-0.00016
C	-0.62935	-1.88835	-0.00026
C	-2.00137	-2.14559	-0.00009
H	-3.99148	-1.34315	0.0003
H	-3.22289	1.04544	0.00026
H	0.12474	-2.66268	-0.00066
H	-2.33707	-3.17535	-0.00015
O	0.73007	1.89152	-0.00025
S	-0.59322	1.89656	-0.00007
O	-0.92751	2.51416	-1.20645
O	-0.92738	2.51405	1.20642
Cl	1.37517	0.00183	0.00002

Sum of electronic and zero-point Energies=-1314.915005
Sum of electronic and thermal Energies= -1314.905577
Sum of electronic and thermal Enthalpies= -1314.904632
Sum of electronic and thermal Free Energies= -1314.951072

4-ClIII-SCF3

C	2.78872	2.53347	-0.0787
C	3.18535	1.22372	-0.31382
C	2.27986	0.18067	-0.13941
C	1.0132	0.52339	0.25857
C	0.55417	1.79211	0.51366
C	1.48729	2.81374	0.3311
H	3.49368	3.34408	-0.21405
H	4.18916	0.96587	-0.63234
H	-0.45947	1.99472	0.82838
H	1.17977	3.83591	0.514
O	1.66601	-2.0776	-0.15099
F	-3.93551	0.92128	-0.45801
F	-2.96359	-0.85487	-1.19914
F	-1.93008	1.02411	-1.25123
C	-2.77223	0.27822	-0.52401
S	-2.16147	-0.00054	1.15757
S	2.65512	-1.26809	-0.37758
O	3.60669	-1.65601	0.56719
O	2.91876	-1.44953	-1.73631
Cl	0.00684	-0.90941	0.43721

Sum of electronic and zero-point Energies=-2050.732544
Sum of electronic and thermal Energies= -2050.717810
Sum of electronic and thermal Enthalpies= -2050.716865
Sum of electronic and thermal Free Energies= -2050.776131

4-IIII-Br

C	-2.93446	-1.10859	0.00015
C	-2.52243	0.21846	0.00015
C	-1.16116	0.50593	0.
C	-0.29395	-0.55644	-0.00016
C	-0.62935	-1.88835	-0.00026
C	-2.00137	-2.14559	-0.00009
H	-3.99148	-1.34315	0.0003
H	-3.22289	1.04544	0.00026
H	0.12474	-2.66268	-0.00066
H	-2.33707	-3.17535	-0.00015
O	0.73007	1.89152	-0.00025
S	-0.59322	1.89656	-0.00007
O	-0.92751	2.51416	-1.20645
O	-0.92738	2.51405	1.20642
Br	2.14787	-1.78352	0.00019
I	1.57797	0.06966	0.00005

Sum of electronic and zero-point Energies=-3726.572185
Sum of electronic and thermal Energies= -3726.561682
Sum of electronic and thermal Enthalpies= -3726.560738
Sum of electronic and thermal Free Energies= -3726.610753

4-IIII-CCH

C	-1.73448	3.03455	-0.00008
C	-2.34105	1.79113	-0.0001
C	-1.55996	0.64271	-0.00004
C	-0.19406	0.7816	0.00004
C	0.45064	1.99943	0.00006
C	-0.34916	3.13487	-0.00001
H	-2.34077	3.92923	-0.00013
H	-3.41797	1.68502	-0.00015
H	1.52496	2.08236	0.00012
H	0.12713	4.10519	0.00001
O	-1.08176	-1.82188	0.00026
S	-2.33535	-0.95715	-0.00008
O	-3.05502	-1.07363	1.22755
O	-3.05449	-1.0738	-1.228
C	2.56364	-0.20579	0.00002
C	3.67725	0.24449	-0.00003
H	4.66923	0.64558	-0.00008
I	0.79291	-0.92176	0.00011

Sum of electronic and zero-point Energies=-1229.062492
Sum of electronic and thermal Energies= -1229.051233
Sum of electronic and thermal Enthalpies= -1229.050289
Sum of electronic and thermal Free Energies= -1229.100861

4-IIII-CF3

C	-2.69011	2.28973	0.00005
C	-2.95457	0.92757	0.0001
C	-1.91235	-0.00045	0.00001
C	-0.64991	0.52412	-0.00006
C	-0.30538	1.85846	-0.00013
C	-1.37667	2.75141	-0.00009
H	-3.50769	2.99983	0.00013
H	-3.96181	0.52596	0.00018
H	0.70804	2.22727	-0.00024
H	-1.16527	3.81354	-0.00016
O	-1.13277	-2.20926	-0.00037
C	2.40402	0.26982	0.00001
F	3.4394	-0.54682	0.00069
F	2.44547	1.03228	1.07774
F	2.44614	1.03133	-1.07837
S	-2.19866	-1.51874	-0.00005
O	-2.79729	-1.88537	1.20651
O	-2.79782	-1.88533	-1.20636
I	0.77117	-0.94889	0.00007

Sum of electronic and zero-point Energies=-1490.001240
Sum of electronic and thermal Energies= -1489.988396
Sum of electronic and thermal Enthalpies= -1489.987451
Sum of electronic and thermal Free Energies= -1490.042093

4-IIII-CH3

C	-2.69011	2.28973	0.00005
C	-2.95457	0.92757	0.0001
C	-1.91235	-0.00045	0.00001
C	-0.64991	0.52412	-0.00006
C	-0.30538	1.85846	-0.00013
C	-1.37667	2.75141	-0.00009
H	-3.50769	2.99983	0.00013
H	-3.96181	0.52596	0.00018
H	0.70804	2.22727	-0.00024
H	-1.16527	3.81354	-0.00016
O	-1.13277	-2.20926	-0.00037
C	2.40402	0.26982	0.00001
S	-2.19866	-1.51874	-0.00005
O	-2.79729	-1.88537	1.20651
O	-2.79782	-1.88533	-1.20636
H	2.4376	0.8875	0.87308
H	3.24415	-0.39281	0.00056
H	2.43814	0.88672	-0.87359
I	0.77117	-0.94889	0.00007

Sum of electronic and zero-point Energies=-1192.220039
Sum of electronic and thermal Energies= -1192.209310
Sum of electronic and thermal Enthalpies= -1192.208365
Sum of electronic and thermal Free Energies= -1192.257589

4-IIII-CHCH2

C	-1.06689	3.03816	0.03847
C	-1.68012	1.79376	0.06931
C	-0.91811	0.63558	0.01112
C	0.44442	0.78647	-0.08177
C	1.10103	1.99756	-0.12932
C	0.3136	3.14155	-0.06095
H	-1.66928	3.93489	0.08284
H	-2.75511	1.68537	0.12775
H	2.17244	2.07977	-0.22947
H	0.79033	4.11092	-0.09894
O	-0.82187	-1.69238	-0.93556
C	3.15698	-0.20687	0.53153
S	-1.65708	-1.01278	0.06058
O	-1.3946	-1.44641	1.41061
O	-3.02741	-0.82108	-0.31319
H	3.65477	0.38383	-0.22751
C	3.95896	-1.47933	0.86209
H	4.92873	-1.20618	1.22243
H	3.44406	-2.0402	1.6139
I	1.47186	-0.87676	-0.18227

Sum of electronic and zero-point Energies=-1230.289874
Sum of electronic and thermal Energies= -1230.277341
Sum of electronic and thermal Enthalpies= -1230.276397

Sum of electronic and thermal Free Energies= -1230.330871

4-IIII-Cl

C	2.52236	2.11339	-0.00003
C	2.66085	0.73134	-0.00002
C	1.52519	-0.07221	-0.00001
C	0.30752	0.56194	0.00001
C	0.09823	1.91869	0.00008
C	1.25733	2.69731	0.00004
H	3.40253	2.74383	-0.00006
H	3.62846	0.24255	-0.00002
H	-0.89074	2.35437	0.00019
H	1.15606	3.77559	0.00007
O	0.38663	-2.12239	-0.00002
Cl	-2.94445	0.85661	-0.00013
S	1.58381	-1.57491	-0.00003
O	2.14123	-2.00188	-1.20648
O	2.14415	-1.99815	1.20639
I	-1.2452	-0.85194	0.00008

Sum of electronic and zero-point Energies=-1612.571215
Sum of electronic and thermal Energies= -1612.561022
Sum of electronic and thermal Enthalpies= -1612.560078
Sum of electronic and thermal Free Energies= -1612.608660

4-IIII-CN

C	2.38355	2.16507	-0.00011
C	2.54579	0.78572	-0.00018
C	1.42977	-0.04976	-0.00014
C	0.21531	0.57735	0.00019
C	-0.03278	1.92761	0.00009
C	1.11008	2.72995	0.00012
H	3.25324	2.81011	-0.00022
H	3.52047	0.31084	-0.00021
H	-1.02602	2.35531	0.00032
H	0.98659	3.8058	0.00018
O	0.38892	-2.14584	0.00036
C	-2.68934	0.59238	0.00006
N	-3.53721	1.37008	0.00019
S	1.54327	-1.57372	0.00002
O	2.10462	-1.99531	-1.20651
O	2.10502	-1.99529	1.20637
I	-1.2004	-0.88851	-0.00018

Sum of electronic and zero-point Energies=-1245.169942
Sum of electronic and thermal Energies= -1245.158922
Sum of electronic and thermal Enthalpies= -1245.157978
Sum of electronic and thermal Free Energies= -1245.208134

4-IIII-F

C	-2.93446	-1.10859	0.00015
C	-2.52243	0.21846	0.00015
C	-1.16116	0.50593	0.
C	-0.29395	-0.55644	-0.00016
C	-0.62935	-1.88835	-0.00026
C	-2.00137	-2.14559	-0.00009
H	-3.99148	-1.34315	0.0003
H	-3.22289	1.04544	0.00026
H	0.12474	-2.66268	-0.00066
H	-2.33707	-3.17535	-0.00015
O	0.73007	1.89152	-0.00025
F	2.14787	-1.78352	0.00019
S	-0.59322	1.89656	-0.00007
O	-0.92751	2.51416	-1.20645
O	-0.92738	2.51405	1.20642
I	1.69761	0.10968	0.00006

Sum of electronic and zero-point Energies=-1252.209224
Sum of electronic and thermal Energies= -1252.199518
Sum of electronic and thermal Enthalpies= -1252.198573
Sum of electronic and thermal Free Energies= -1252.245662

4-IIII-N3

C	-2.93446	-1.10859	0.00015
C	-2.52243	0.21846	0.00015
C	-1.16116	0.50593	0.
C	-0.29395	-0.55644	-0.00016
C	-0.62935	-1.88835	-0.00026
C	-2.00137	-2.14559	-0.00009
H	-3.99148	-1.34315	0.0003
H	-3.22289	1.04544	0.00026
H	0.12474	-2.66268	-0.00066
H	-2.33707	-3.17535	-0.00015
O	0.73007	1.89152	-0.00025
S	-0.59322	1.89656	-0.00007
O	-0.92751	2.51416	-1.20645
O	-0.92738	2.51405	1.20642
N	2.11882	-1.68906	0.00018
N	2.60112	-3.93983	-0.89211
N	2.35359	-2.93701	-0.53779
I	1.57797	0.06966	0.00005

Sum of electronic and zero-point Energies=-1316.531015
Sum of electronic and thermal Energies= -1316.518446
Sum of electronic and thermal Enthalpies= -1316.517502
Sum of electronic and thermal Free Energies= -1316.572556

4-IIII-NH2

C	-1.73448	3.03455	-0.00008
C	-2.34105	1.79113	-0.0001
C	-1.55996	0.64271	-0.00004
C	-0.19406	0.7816	0.00004
C	0.45064	1.99943	0.00006
C	-0.34916	3.13487	-0.00001
H	-2.34077	3.92923	-0.00013
H	-3.41797	1.68502	-0.00015
H	1.52496	2.08236	0.00012
H	0.12713	4.10519	0.00001
O	-1.08176	-1.82188	0.00026
S	-2.33535	-0.95715	-0.00008
O	-3.05502	-1.07363	1.22755
O	-3.05449	-1.0738	-1.228
N	2.49874	-0.23203	0.00003
H	2.98449	-0.54405	0.81654
H	2.98446	-0.54417	-0.81646
I	0.79291	-0.92176	0.00011

Sum of electronic and zero-point Energies=-1208.276949
Sum of electronic and thermal Energies= -1208.266469
Sum of electronic and thermal Enthalpies= -1208.265525
Sum of electronic and thermal Free Energies= -1208.313988

4-IIII-NHAc

C	-2.17513	2.89633	-0.00006
C	-2.61484	1.58466	-0.00008
C	-1.68624	0.5528	-0.00008
C	-0.34787	0.86082	-0.00002
C	0.12958	2.15803	-0.00002
C	-0.81404	3.17627	-0.00003
H	-2.89259	3.70464	-0.00007
H	-3.66855	1.33932	-0.00009
H	1.18347	2.37431	-0.00002
H	-0.47135	4.20156	-0.00002
O	-0.84815	-1.80657	0.00013
C	3.48374	-0.17056	-0.00013
O	3.5246	-1.37015	-0.00022
C	4.67381	0.74661	0.00039
H	4.6399	1.38871	-0.87819
H	4.6425	1.38351	0.88288
H	5.58034	0.15093	-0.00251
S	-2.22312	-1.13898	0.00009
O	-2.91279	-1.37052	-1.22742
O	-2.91269	-1.37026	1.22771
N	2.33301	0.51156	-0.00017
H	2.46269	1.38405	0.47093
I	0.83503	-0.6835	-0.0001

Sum of electronic and zero-point Energies=-1360.901038
Sum of electronic and thermal Energies= -1360.886187
Sum of electronic and thermal Enthalpies= -1360.885243
Sum of electronic and thermal Free Energies= -1360.945131

4-IIII-OCF3

C	2.67388	2.50582	-0.08055
C	3.07538	1.1861	-0.2436
C	2.13864	0.16777	-0.09614
C	0.85091	0.5348	0.20224
C	0.38735	1.81389	0.38416
C	1.35008	2.81324	0.23105
H	3.39497	3.30541	-0.19349
H	4.09694	0.91345	-0.48175
H	-0.64249	2.02395	0.63
H	1.04878	3.8455	0.35987
O	1.40068	-2.06043	-0.03196
F	-3.25665	-0.89735	-0.63674
F	-2.19308	0.86999	-1.26105
F	-3.78459	1.02948	0.19587
C	-2.75499	0.26941	-0.18023
O	-1.90316	0.11538	0.81056
S	2.46298	-1.28967	-0.23514
O	3.33618	-1.65768	0.78988
O	2.81862	-1.53021	-1.56338
I	-0.3585	-1.17318	0.37575

Sum of electronic and zero-point Energies=-1565.258042
Sum of electronic and thermal Energies= -1565.243749
Sum of electronic and thermal Enthalpies= -1565.242805
Sum of electronic and thermal Free Energies= -1565.302057

4-IIII-OCH3

C	2.67388	2.50582	-0.08055
C	3.07538	1.1861	-0.2436
C	2.13864	0.16777	-0.09614
C	0.85091	0.5348	0.20224
C	0.38735	1.81389	0.38416
C	1.35008	2.81324	0.23105
H	3.39497	3.30541	-0.19349
H	4.09694	0.91345	-0.48175
H	-0.64249	2.02395	0.63
H	1.04878	3.8455	0.35987
O	1.40068	-2.06043	-0.03196
C	-2.75499	0.26941	-0.18023
O	-1.90316	0.11538	0.81056

S	2.46298	-1.28967	-0.23514
O	3.33618	-1.65768	0.78988
O	2.81862	-1.53021	-1.56338
H	-2.3123	0.74256	-1.03173
H	-3.5809	0.87912	0.12146
H	-3.15272	-0.65563	-0.54216
I	-0.3585	-1.17318	0.37575

Sum of electronic and zero-point Energies=-1267.431484
Sum of electronic and thermal Energies= -1267.418977
Sum of electronic and thermal Enthalpies= -1267.418033
Sum of electronic and thermal Free Energies= -1267.471625

4-IIII-OCOCF3

C	3.22289	2.62188	0.00859
C	3.68898	1.31361	0.00651
C	2.77394	0.26568	0.00105
C	1.43871	0.58809	-0.00237
C	0.91134	1.85792	-0.00002
C	1.85448	2.88734	0.0057
H	3.92673	3.44448	0.01292
H	4.74543	1.07154	0.00947
H	-0.15229	2.04153	-0.00164
H	1.50103	3.91113	0.00798
O	2.11919	-1.99141	-0.00185
C	-2.41859	-0.53165	0.0117
O	-2.5234	-1.72768	0.04512
O	-1.32746	0.16971	-0.02174
C	-3.65945	0.39701	0.00003
F	-4.78188	-0.30037	0.05516
F	-3.67375	1.13597	-1.11316
F	-3.62623	1.23022	1.0443
S	3.17219	-1.17944	0.00108
O	3.81228	-1.46262	1.20882
O	3.8193	-1.45784	-1.20404
I	0.2862	-1.16738	-0.00904

Sum of electronic and zero-point Energies=-1678.577558
Sum of electronic and thermal Energies= -1678.561885
Sum of electronic and thermal Enthalpies= -1678.560941
Sum of electronic and thermal Free Energies= -1678.625116

4-IIII-OCOCH3

C	2.94595	2.24262	0.
C	3.14905	0.86891	-0.00023
C	2.05101	0.01452	-0.00014
C	0.80079	0.58421	0.00015
C	0.53351	1.93493	0.00041
C	1.65414	2.76631	0.00033
H	3.79558	2.9138	-0.00007
H	4.13833	0.42604	-0.00045
H	-0.47532	2.31781	0.00073
H	1.50439	3.83907	0.00055
O	1.00226	-2.08015	0.
C	-3.20495	0.1778	0.00021
O	-3.42588	-1.00956	0.00076
O	-1.9799	0.68529	0.00005
C	-4.26655	1.25034	-0.00099
H	-4.14463	1.88403	0.87785
H	-4.15011	1.8754	-0.88681
H	-5.24737	0.78154	0.00398
S	2.175	-1.48618	-0.00024
O	2.75067	-1.8887	1.20606
O	2.75026	-1.88842	-1.20682
I	-0.68643	-0.89842	0.00013

Sum of electronic and zero-point Energies=-1380.792535
Sum of electronic and thermal Energies= -1380.778753
Sum of electronic and thermal Enthalpies= -1380.777809
Sum of electronic and thermal Free Energies= -1380.835027

4-IIII-OCOPh

C	-2.17513	2.89633	-0.00006
C	-2.61484	1.58466	-0.00008
C	-1.68624	0.5528	-0.00008
C	-0.34787	0.86082	-0.00002
C	0.12958	2.15803	-0.00002
C	-0.81404	3.17627	-0.00003
H	-2.89259	3.70464	-0.00007
H	-3.66855	1.33932	-0.00009
H	1.18347	2.37431	-0.00002
H	-0.47135	4.20156	-0.00002
O	-0.84815	-1.80657	0.00013
C	3.48374	-0.17056	-0.00013
O	3.5246	-1.37015	-0.00211
O	2.32856	0.5142	0.00225
S	-2.22312	-1.13898	0.00009
O	-2.91279	-1.37052	-1.22742
O	-2.91269	-1.37026	1.22771
C	4.70352	0.76951	0.0004
C	5.99358	0.23827	-0.00183
C	4.51865	2.15203	0.00301
C	7.09845	1.08942	-0.00078
H	6.13899	-0.85173	-0.00311
C	5.62375	3.00359	0.00307
H	3.50195	2.57091	0.00459
C	6.91353	2.47254	0.00131
H	8.11532	0.67076	-0.00191
H	5.47768	4.0936	0.00471
H	7.78484	3.14345	0.00208
I	0.9291	-0.80632	-0.0001

Sum of electronic and zero-point Energies=-1572.470485
Sum of electronic and thermal Energies= -1572.453906
Sum of electronic and thermal Enthalpies= -1572.452962
Sum of electronic and thermal Free Energies= -1572.517592

4-IIII-OH

C	2.85263	-1.32247	0.00438
C	2.53897	0.03132	0.02066
C	1.20519	0.42867	0.01343
C	0.26118	-0.56546	0.00447
C	0.49803	-1.91871	-0.02637
C	1.8446	-2.28523	-0.02237
H	3.88939	-1.63508	0.00637
H	3.29795	0.80511	0.03341
H	-0.31078	-2.63443	-0.06185
H	2.09873	-3.33788	-0.04592
O	-0.53232	1.99106	-0.02484
O	-2.35941	-1.6203	-0.02187
H	-2.69235	-1.77691	0.86934
S	0.76361	1.8772	0.00979
O	1.15963	2.48527	-1.18269
O	1.12457	2.45173	1.2297
I	-1.68965	0.21143	-0.0219

Sum of electronic and zero-point Energies=-1228.167949
Sum of electronic and thermal Energies= -1228.157026
Sum of electronic and thermal Enthalpies= -1228.156082
Sum of electronic and thermal Free Energies= -1228.205878

4-IIII-OTf

C	-2.93446	-1.10859	-0.00888
C	-2.52242	0.21846	-0.00736
C	-1.16116	0.50593	-0.00483
C	-0.29394	-0.55644	-0.00398
C	-0.62934	-1.88835	-0.00546
C	-2.00136	-2.14559	-0.00799
H	-3.99147	-1.34315	-0.01083
H	-3.22288	1.04544	-0.00808
H	0.12474	-2.66268	-0.00491
H	-2.33706	-3.17535	-0.00925
O	0.73007	1.89152	-0.00078
S	-0.59322	1.89656	-0.00305
O	-0.92527	2.51485	-1.2097
O	-0.92961	2.51337	1.20317
O	2.10706	-1.65082	0.00018
S	3.59172	-1.98216	0.66714
O	3.32676	-2.88338	1.67691
O	4.05228	-0.70457	0.94529
C	4.38455	-2.66753	-0.56795
F	4.65878	-1.73163	-1.50143
F	5.53789	-3.20648	-0.11868
F	3.61776	-3.6357	-1.11309
I	1.57797	0.06966	0.00005

Sum of electronic and zero-point Energies=-2113.856711
Sum of electronic and thermal Energies= -2113.839079
Sum of electronic and thermal Enthalpies= -2113.838135
Sum of electronic and thermal Free Energies= -2113.906169

4-IIII-OTs

C	-2.93446	-1.10859	-0.00888
C	-2.52242	0.21846	-0.00736
C	-1.16116	0.50593	-0.00483
C	-0.29394	-0.55644	-0.00398
C	-0.62934	-1.88835	-0.00546
C	-2.00136	-2.14559	-0.00799
H	-3.99147	-1.34315	-0.01083
H	-3.22288	1.04544	-0.00808
H	0.12474	-2.66268	-0.00491
H	-2.33706	-3.17535	-0.00925
O	0.73007	1.89152	-0.00078
S	-0.59322	1.89656	-0.00305
O	-0.92527	2.51485	-1.2097
O	-0.92961	2.51337	1.20317
O	2.10706	-1.65082	0.00018
S	3.59172	-1.98216	0.66714
O	3.32676	-2.88338	1.67691
O	4.05228	-0.70457	0.94529
C	4.39928	-2.68027	-0.5909
C	4.29138	-4.05719	-0.83807
C	5.21657	-1.91696	-1.4387
C	4.97128	-4.65229	-1.89405
H	3.65929	-4.6737	-0.17312
C	5.90065	-2.50387	-2.4957
H	5.32036	-0.83269	-1.25073
C	5.78172	-3.87969	-2.73634
H	4.87422	-5.73446	-2.06996
H	6.53939	-1.88796	-3.14689
C	6.49302	-4.50693	-3.87273
H	7.41984	-3.93793	-4.13143

H	6.77766	-5.5615	-3.63417
H	5.82634	-4.51922	-4.77277
I	1.57797	0.06966	0.00005

Sum of electronic and zero-point Energies=-2047.036395
Sum of electronic and thermal Energies= -2047.015824
Sum of electronic and thermal Enthalpies= -2047.014880
Sum of electronic and thermal Free Energies= -2047.090711

4-IIII-radical

C	-2.93446	-1.10859	0.00015
C	-2.52243	0.21846	0.00015
C	-1.16116	0.50593	0.
C	-0.29395	-0.55644	-0.00016
C	-0.62935	-1.88835	-0.00026
C	-2.00137	-2.14559	-0.00009
H	-3.99148	-1.34315	0.0003
H	-3.22289	1.04544	0.00026
H	0.12474	-2.66268	-0.00066
H	-2.33707	-3.17535	-0.00015
O	0.73007	1.89152	-0.00025
S	-0.59322	1.89656	-0.00007
O	-0.92751	2.51416	-1.20645
O	-0.92738	2.51405	1.20642
I	1.69761	0.10968	0.00006

Sum of electronic and zero-point Energies=-1152.348364
Sum of electronic and thermal Energies= -1152.339715
Sum of electronic and thermal Enthalpies= -1152.338771
Sum of electronic and thermal Free Energies= -1152.384536

4-IIII-SCF3

C	2.78872	2.53347	-0.0787
C	3.18535	1.22372	-0.31382
C	2.27986	0.18067	-0.13941
C	1.0132	0.52339	0.25857
C	0.55417	1.79211	0.51366
C	1.48729	2.81374	0.3311
H	3.49368	3.34408	-0.21405
H	4.18916	0.96587	-0.63234
H	-0.45947	1.99472	0.82838
H	1.17977	3.83591	0.514
O	1.66601	-2.0776	-0.15099
F	-3.93551	0.92128	-0.45801
F	-2.96359	-0.85487	-1.19914
F	-1.93008	1.02411	-1.25123
C	-2.77223	0.27822	-0.52401
S	-2.16147	-0.00054	1.15757
S	2.65512	-1.26809	-0.37758
O	3.60669	-1.65601	0.56719
O	2.91876	-1.44953	-1.73631
I	-0.18758	-1.1862	0.47172

Sum of electronic and zero-point Energies=-1888.216402
Sum of electronic and thermal Energies= -1888.201291
Sum of electronic and thermal Enthalpies= -1888.200347
Sum of electronic and thermal Free Energies= -1888.261677

5-BrIII-Br

C	-2.93446	-1.10859	0.00015
C	-2.52243	0.21846	0.00015
C	-1.16116	0.50593	0.
C	-0.29395	-0.55644	-0.00016
C	-0.62935	-1.88835	-0.00026
C	-2.00137	-2.14559	-0.00009
H	-3.99148	-1.34315	0.0003
H	-3.22289	1.04544	0.00026
H	0.12474	-2.66268	-0.00066
H	-2.33707	-3.17535	-0.00015
Br	1.57797	0.06966	0.00005
C	-0.59322	1.89656	-0.00007
O	-1.26974	2.88566	0.00007
N	0.74447	1.89146	-0.00025
C	1.48431	3.16171	-0.00059
O	2.74271	3.1619	-0.00077
C	0.71675	4.4968	-0.00073
H	0.1032	4.55537	-0.87539
H	0.1002	4.55383	0.87191
H	1.41356	5.30881	0.00118
Br	2.14787	-1.78352	0.00019

Sum of electronic and zero-point Energies=-5700.551698
Sum of electronic and thermal Energies= -5700.538239
Sum of electronic and thermal Enthalpies= -5700.537295
Sum of electronic and thermal Free Energies= -5700.593850

5-BrIII-CCH

C	-0.12242	3.85594	0.
C	-1.22379	3.01699	0.00002
C	-1.03566	1.64264	0.00001
C	0.25381	1.14434	-0.00001
C	1.37222	1.94558	-0.00003
C	1.15994	3.32056	-0.00002
H	-0.25826	4.92824	0.00001
H	-2.23893	3.39099	0.00004
H	2.367	1.52892	-0.00005
H	2.01967	3.9763	-0.00004
Br	0.32073	-0.79887	-0.00002
C	-2.17681	0.68988	0.00002
O	-3.33648	1.02873	0.00008
N	-1.68975	-0.59082	-0.00004
C	-2.36863	-1.80677	0.
O	-1.72138	-2.82636	0.00006
C	-3.86977	-1.77147	-0.00007
H	-4.23289	-1.23044	0.87185
H	-4.23286	-1.23054	-0.87205
H	-4.22553	-2.7968	0.
C	2.22585	-0.93536	0.00001
C	3.42398	-1.0212	0.00002
H	4.49124	-1.09766	0.00004

Sum of electronic and zero-point Energies=-3203.032229
Sum of electronic and thermal Energies= -3203.017936
Sum of electronic and thermal Enthalpies= -3203.016991
Sum of electronic and thermal Free Energies= -3203.074447

5-BrIII-CF3

C	-2.69011	2.28973	0.00005
C	-2.95457	0.92757	0.0001
C	-1.91235	-0.00045	0.00001
C	-0.64991	0.52412	-0.00006
C	-0.30538	1.85846	-0.00013
C	-1.37667	2.75141	-0.00009
H	-3.50769	2.99983	0.00013
H	-3.96181	0.52596	0.00018
H	0.70804	2.22727	-0.00024
H	-1.16527	3.81354	-0.00016
Br	0.77117	-0.94889	0.00007
C	-2.19866	-1.51874	-0.00005
O	-3.35993	-1.86798	0.00015
C	2.40402	0.26982	0.00001
F	3.4394	-0.54682	0.00069
F	2.44547	1.03228	1.07774
F	2.44614	1.03133	-1.07837
N	-1.11298	-2.22208	-0.00038
C	-1.18829	-3.69015	-0.0004
O	-0.13487	-4.37853	-0.00075
C	-2.56105	-4.3881	-0.00001
H	-3.10734	-4.10011	0.87379
H	-3.10783	-4.10012	-0.87351
H	-2.42178	-5.44899	-0.00004

Sum of electronic and zero-point Energies=-3463.980214
Sum of electronic and thermal Energies= -3463.964339
Sum of electronic and thermal Enthalpies= -3463.963395
Sum of electronic and thermal Free Energies= -3464.025197

5-BrIII-CH3

C	-2.69011	2.28973	0.00005
C	-2.95457	0.92757	0.0001
C	-1.91235	-0.00045	0.00001
C	-0.64991	0.52412	-0.00006
C	-0.30538	1.85846	-0.00013
C	-1.37667	2.75141	-0.00009
H	-3.50769	2.99983	0.00013
H	-3.96181	0.52596	0.00018
H	0.70804	2.22727	-0.00024
H	-1.16527	3.81354	-0.00016
Br	0.77117	-0.94889	0.00007
C	-2.19866	-1.51874	-0.00005
O	-3.35993	-1.86798	0.00015
C	2.40402	0.26982	0.00001
N	-1.11298	-2.22208	-0.00038
C	-1.18829	-3.69015	-0.0004
O	-0.13487	-4.37853	-0.00075
C	-2.56105	-4.3881	-0.00001
H	-3.10734	-4.10011	0.87379
H	-3.10783	-4.10012	-0.87351
H	-2.42178	-5.44899	-0.00004
H	2.4376	0.8875	0.87308
H	3.24415	-0.39281	0.00056
H	2.43814	0.88672	-0.87359

Sum of electronic and zero-point Energies=-3166.193201
Sum of electronic and thermal Energies= -3166.179537
Sum of electronic and thermal Enthalpies= -3166.178592
Sum of electronic and thermal Free Energies= -3166.234654

5-BrIII-CHCH2

C	-2.95217	-2.14162	0.00011
C	-1.58097	-2.33556	0.00027
C	-0.70784	-1.25544	0.00005
C	-1.25578	0.01702	-0.00018
C	-2.61783	0.24712	-0.00043
C	-3.46674	-0.85373	-0.00029
H	-3.62112	-2.99094	0.00024
H	-1.13619	-3.32193	0.00053
H	-3.03887	1.23823	-0.00073
H	-4.53556	-0.6903	-0.00052
Br	0.00294	1.50101	-0.0002
C	0.78828	-1.49791	-0.00013
O	1.21495	-2.64048	0.00052
C	-1.34728	2.96144	0.00136

N	1.41303	-0.31693	-0.00121
C	2.76821	-0.07887	-0.00035
O	3.16153	1.0733	-0.00041
C	3.72445	-1.24393	0.00076
H	3.54599	-1.87415	0.87084
H	3.54678	-1.87522	-0.86868
H	4.73956	-0.85815	0.00104
H	-1.94734	2.91361	0.90356
C	-0.4665	4.2247	0.00257
H	-1.09003	5.09424	0.00131
H	0.15274	4.22859	-0.87002

Sum of electronic and zero-point Energies=-3204.262865
Sum of electronic and thermal Energies= -3204.248269
Sum of electronic and thermal Enthalpies= -3204.247325
Sum of electronic and thermal Free Energies= -3204.305916

5-BrIII-Cl

C	2.52236	2.11339	-0.00003
C	2.66085	0.73134	-0.00002
C	1.52519	-0.07221	-0.00001
C	0.30752	0.56194	0.00001
C	0.09823	1.91869	0.00008
C	1.25733	2.69731	0.00004
H	3.40253	2.74383	-0.00006
H	3.62846	0.24255	-0.00002
H	-0.89074	2.35437	0.00019
H	1.15606	3.77559	0.00007
Br	-1.17132	-0.78467	0.00008
C	1.58381	-1.57491	-0.00003
O	2.61636	-2.18619	-0.00011
Cl	-2.94445	0.85661	-0.00013
N	0.36728	-2.13124	-0.00002
C	0.2283	-3.59465	-0.00005
O	-0.91402	-4.12253	-0.00004
C	1.48498	-4.4848	-0.00011
H	2.06652	-4.28081	-0.87481
H	2.06864	-4.27792	0.87249
H	1.19292	-5.51417	0.00195

Sum of electronic and zero-point Energies=-3586.550053
Sum of electronic and thermal Energies= -3586.536820
Sum of electronic and thermal Enthalpies= -3586.535876
Sum of electronic and thermal Free Energies= -3586.591142

5-BrIII-CN

C	2.38355	2.16507	-0.00011
C	2.54579	0.78572	-0.00018
C	1.42977	-0.04976	-0.00014
C	0.21531	0.57735	0.00019
C	-0.03278	1.92761	0.00009
C	1.11008	2.72995	0.00012
H	3.25324	2.81011	-0.00022
H	3.52047	0.31084	-0.00021
H	-1.02602	2.35531	0.00032
H	0.98659	3.8058	0.00018
Br	-1.29133	-0.79808	-0.00016
C	1.54327	-1.57372	0.00002
O	2.63468	-2.0876	0.00015
C	-2.68934	0.59238	0.00006
N	-3.53721	1.37008	0.00019
N	0.3447	-2.16775	0.00037
C	0.25148	-3.6348	0.0001
O	-0.87381	-4.19808	0.00054
C	1.53533	-4.48528	-0.00073
H	2.11082	-4.26153	-0.87461
H	2.11166	-4.26197	0.8727
H	1.27555	-5.52327	-0.00086

Sum of electronic and zero-point Energies=-3219.144081
Sum of electronic and thermal Energies= -3219.129952
Sum of electronic and thermal Enthalpies= -3219.129008
Sum of electronic and thermal Free Energies= -3219.186092

5-BrIII-F

C	-2.93446	-1.10859	0.00015
C	-2.52243	0.21846	0.00015
C	-1.16116	0.50593	0.
C	-0.29395	-0.55644	-0.00016
C	-0.62935	-1.88835	-0.00026
C	-2.00137	-2.14559	-0.00009
H	-3.99148	-1.34315	0.0003
H	-3.22289	1.04544	0.00026
H	0.12474	-2.66268	-0.00066
H	-2.33707	-3.17535	-0.00015
Br	1.57797	0.06966	0.00005
C	-0.59322	1.89656	-0.00007
O	-1.26974	2.88566	0.00007
F	2.14787	-1.78352	0.00019
N	0.74447	1.89146	-0.00025
C	1.48431	3.16171	-0.00059
O	2.74271	3.1619	-0.00077
C	0.71675	4.4968	-0.00073
H	0.1032	4.55537	-0.87539
H	0.1002	4.55383	0.87191
H	1.41356	5.30881	0.00118

Sum of electronic and zero-point Energies=-3226.184902
Sum of electronic and thermal Energies= -3226.172362
Sum of electronic and thermal Enthalpies= -3226.171418
Sum of electronic and thermal Free Energies= -3226.224442

5-BrIII-N3

C	-2.93446	-1.10859	0.00015
C	-2.52243	0.21846	0.00015
C	-1.16116	0.50593	0.
C	-0.29395	-0.55644	-0.00016
C	-0.62935	-1.88835	-0.00026
C	-2.00137	-2.14559	-0.00009
H	-3.99148	-1.34315	0.0003
H	-3.22289	1.04544	0.00026
H	0.12474	-2.66268	-0.00066
H	-2.33707	-3.17535	-0.00015
Br	1.57797	0.06966	0.00005
C	-0.59322	1.89656	-0.00007
O	-1.26974	2.88566	0.00007
N	0.74447	1.89146	-0.00025
C	1.48431	3.16171	-0.00059
O	2.74271	3.1619	-0.00077
C	0.71675	4.4968	-0.00073
H	0.1032	4.55537	-0.87539
H	0.1002	4.55383	0.87191
H	1.41356	5.30881	0.00118
N	2.11882	-1.68906	0.00018
N	2.65816	-3.9473	0.79181
N	2.41713	-2.93233	0.46901

Sum of electronic and zero-point Energies=-3290.506482
Sum of electronic and thermal Energies= -3290.491706
Sum of electronic and thermal Enthalpies= -3290.490762
Sum of electronic and thermal Free Energies= -3290.550538

5-BrIII-NH2

C	-0.12242	3.85594	0.
C	-1.22379	3.01699	0.00002
C	-1.03566	1.64264	0.00001
C	0.25381	1.14434	-0.00001
C	1.37222	1.94558	-0.00003
C	1.15994	3.32056	-0.00002
H	-0.25826	4.92824	0.00001
H	-2.23893	3.39099	0.00004
H	2.367	1.52892	-0.00005
H	2.01967	3.9763	-0.00004
Br	0.32073	-0.79887	-0.00002
C	-2.17681	0.68988	0.00002
O	-3.33648	1.02873	0.00008
N	-1.68975	-0.59082	-0.00004
C	-2.36863	-1.80677	0.
O	-1.72138	-2.82636	0.00006
C	-3.86977	-1.77147	-0.00007
N	2.15603	-0.93035	0.00001
H	-4.22672	-1.23963	0.85704
H	-4.2267	-1.23972	-0.85724
H	-4.22052	-2.78235	0.
H	2.45492	-1.42281	0.81741
H	2.45472	-1.42594	-0.81558

Sum of electronic and zero-point Energies=-3182.247575
Sum of electronic and thermal Energies= -3182.233996
Sum of electronic and thermal Enthalpies= -3182.233052
Sum of electronic and thermal Free Energies= -3182.288596

5-BrIII-NHAc

C	1.63714	3.63895	-0.00018
C	2.3238	2.43747	-0.00017
C	1.61133	1.2471	-0.00002
C	0.22981	1.28449	0.0001
C	-0.48683	2.46466	0.00011
C	0.24673	3.64566	-0.00004
H	2.18007	4.57361	-0.0003
H	3.40428	2.38576	-0.00027
H	-1.56292	2.46625	0.00019
H	-0.2871	4.58611	-0.00005
Br	-0.57322	-0.46698	0.00031
C	2.28255	-0.07898	0.00003
O	3.48193	-0.22651	-0.0001
C	-3.34264	-0.4131	0.00002
O	-3.14384	-1.60602	0.0004
C	-4.71944	0.20482	-0.00076
H	-4.83446	0.83892	0.87706
H	-4.83535	0.83366	-0.88228
H	-5.46909	-0.57976	0.0018
N	1.32578	-1.06142	0.0005
C	1.48423	-2.44873	-0.00008
O	0.49228	-3.13523	-0.00021
C	2.88066	-2.99879	-0.00053
H	3.42605	-2.64223	-0.8724
H	3.42669	-2.64251	0.87103
H	2.80888	-4.08173	-0.00075
N	-2.36209	0.49681	0.
H	-2.67317	1.32211	0.47129

Sum of electronic and zero-point Energies=-3334.870457

Sum of electronic and thermal Energies= -3334.853445
Sum of electronic and thermal Enthalpies= -3334.852501
Sum of electronic and thermal Free Energies= -3334.916777

5-BrIII-OCF3
C 2.67388 2.50582 -0.08055
C 3.07538 1.1861 -0.2436
C 2.13864 0.16777 -0.09614
C 0.85091 0.5348 0.20224
C 0.38735 1.81389 0.38416
C 1.35008 2.81324 0.23105
H 3.39497 3.30541 -0.19349
H 4.09694 0.91345 -0.48175
H -0.64249 2.02395 0.63
H 1.04878 3.8455 0.35987
Br -0.29449 -1.08279 0.36657
C 2.46298 -1.28967 -0.23514
O 3.55405 -1.70721 -0.49333
F -3.25665 -0.89735 -0.63674
F -2.19308 0.86999 -1.26105
F -3.78459 1.02948 0.19587
C -2.75499 0.26941 -0.18023
O -1.90316 0.11538 0.81056
N 1.39299 -2.066 -0.03049
C 1.5195 -3.52829 -0.11181
O 0.51575 -4.26265 0.07995
C 2.88509 -4.1637 -0.43278
H 3.22204 -3.82107 -1.3888
H 3.59449 -3.8824 0.31725
H 2.78758 -5.22911 -0.44979
Sum of electronic and zero-point Energies=-3539.237134
Sum of electronic and thermal Energies= -3539.220639
Sum of electronic and thermal Enthalpies= -3539.219694
Sum of electronic and thermal Free Energies= -3539.283413

5-BrIII-OCH3
C 2.67388 2.50582 -0.08055
C 3.07538 1.1861 -0.2436
C 2.13864 0.16777 -0.09614
C 0.85091 0.5348 0.20224
C 0.38735 1.81389 0.38416
C 1.35008 2.81324 0.23105
H 3.39497 3.30541 -0.19349
H 4.09694 0.91345 -0.48175
H -0.64249 2.02395 0.63
H 1.04878 3.8455 0.35987
Br -0.29449 -1.08279 0.36657
C 2.46298 -1.28967 -0.23514
O 3.55405 -1.70721 -0.49333
C -2.75499 0.26941 -0.18023
O -1.90316 0.11538 0.81056
N 1.39299 -2.066 -0.03049
C 1.5195 -3.52829 -0.11181
O 0.51575 -4.26265 0.07995
C 2.88509 -4.1637 -0.43278
H 3.22204 -3.82107 -1.3888
H 3.59449 -3.8824 0.31725
H 2.78758 -5.22911 -0.44979
H -2.3123 0.74256 -1.03173
H -3.5809 0.87912 0.12146
H -3.15272 -0.65563 -0.54216
Sum of electronic and zero-point Energies=-3241.402664
Sum of electronic and thermal Energies= -3241.388024
Sum of electronic and thermal Enthalpies= -3241.387079
Sum of electronic and thermal Free Energies= -3241.444993

5-BrIII-OCOCF3
C 3.22289 2.62188 0.00859
C 3.68898 1.31361 0.00651
C 2.77394 0.26568 0.00105
C 1.43871 0.58809 -0.00237
C 0.91134 1.85792 -0.00002
C 1.85448 2.88734 0.0057
H 3.92673 3.44448 0.01292
H 4.74543 1.07154 0.00947
H -0.15229 2.04153 -0.00164
H 1.50103 3.91113 0.00798
Br 0.34895 -1.0718 -0.00868
C 3.17219 -1.17944 0.00108
O 4.30614 -1.55945 0.00406
C -2.41859 -0.53165 0.0117
O -2.5234 -1.72768 0.04512
O -1.32746 0.16971 -0.02174
C -3.65945 0.39701 0.00003
F -4.78188 -0.30037 0.05516
F -3.67375 1.13597 -1.11316
F -3.62623 1.23022 1.0443
N 2.11286 -1.9963 -0.00187
C 2.30819 -3.45326 -0.00066
O 1.31471 -4.22563 -0.00342
C 3.73351 -4.03639 0.00401
H 4.25728 -3.70507 -0.86822
H 4.25194 -3.70414 0.87908
H 3.68162 -5.10513 0.00442
Sum of electronic and zero-point Energies=-3652.557450
Sum of electronic and thermal Energies= -3652.538803
Sum of electronic and thermal Enthalpies= -3652.537859
Sum of electronic and thermal Free Energies= -3652.608148

5-BrIII-OCOCH3
C 2.94595 2.24262 0.
C 3.14905 0.86891 -0.00023
C 2.05101 0.01452 -0.00014
C 0.80079 0.58421 0.00015
C 0.53351 1.93493 0.00041
C 1.65414 2.76631 0.00033
H 3.79558 2.9138 -0.00007
H 4.13833 0.42604 -0.00045
H -0.47532 2.31781 0.00073
H 1.50439 3.83907 0.00055
Br -0.60316 -0.8154 0.00013
C 2.175 -1.48618 -0.00024
O 3.23627 -2.04801 -0.00049
C -3.20495 0.1778 0.00021
O -3.42588 -1.00956 0.00076
O -1.9799 0.68529 0.00005
C -4.26655 1.25034 -0.00099
H -4.14463 1.88403 0.87785
H -4.15011 1.8754 -0.88681
H -5.24737 0.78154 0.00398
N 0.98164 -2.0906 0.
C 0.90116 -3.55839 0.0001
O -0.20818 -4.13204 -0.15442
C 2.17881 -4.39694 0.18995
H 2.82647 -4.25531 -0.64992
H 2.67977 -4.08649 1.08301
H 1.91791 -5.43166 0.26866
Sum of electronic and zero-point Energies=-3354.765434
Sum of electronic and thermal Energies= -3354.748597
Sum of electronic and thermal Enthalpies= -3354.747653
Sum of electronic and thermal Free Energies= -3354.812038

5-BrIII-OCOPh
C 1.63714 3.63895 -0.00018
C 2.3238 2.43747 -0.00017
C 1.61133 1.2471 -0.00002
C 0.22981 1.28449 0.0001
C -0.48683 2.46466 0.00011
C 0.24673 3.64566 -0.00004
H 2.18007 4.57361 -0.0003
H 3.40428 2.38576 -0.00027
H -1.56292 2.46625 0.00019
H -0.2871 4.58611 -0.00005
Br -0.57322 -0.46698 0.00031
C 2.28255 -0.07898 0.00003
O 3.48193 -0.22651 -0.0001
C -3.34264 -0.4131 0.00002
O -3.14384 -1.60602 0.00235
O -2.37521 0.48463 -0.00236
N 1.32578 -1.06142 0.0005
C 1.48423 -2.44873 -0.00008
O 0.49228 -3.13523 -0.00021
C 2.88066 -2.99879 -0.00053
H 3.42605 -2.64223 -0.8724
H 3.42669 -2.64251 0.87103
H 2.80888 -4.08173 -0.00075
C -4.74763 0.21747 -0.00077
C -5.87874 -0.59926 0.00149
C -4.88939 1.60507 -0.00365
C -7.15129 -0.02843 0.0002
H -5.76664 -1.69319 0.00298
C -6.16225 2.17624 -0.00395
H -3.998 2.24893 -0.00526
C -7.29314 1.35976 -0.00216
H -8.04289 -0.67213 0.00136
H -6.27371 3.27033 -0.0058
H -8.2966 1.80961 -0.00312
Sum of electronic and zero-point Energies=-3546.443491
Sum of electronic and thermal Energies= -3546.423898
Sum of electronic and thermal Enthalpies= -3546.422954
Sum of electronic and thermal Free Energies= -3546.495065

5-BrIII-OH
C 2.85263 -1.32247 0.00438
C 2.53897 0.03132 0.02066
C 1.20519 0.42867 0.01343
C 0.26118 -0.56546 0.00447
C 0.49803 -1.91871 -0.02637
C 1.8446 -2.28523 -0.02237
H 3.88939 -1.63508 0.00637
H 3.29795 0.80511 0.03341
H -0.31078 -2.63443 -0.06185
H 2.09873 -3.33788 -0.04592
Br -1.58476 0.16966 -0.02049
C 0.76361 1.8772 0.00979
O 1.56652 2.77539 0.03319

O	-2.35941	-1.6203	-0.02187
H	-2.69235	-1.77691	0.86934
N	-0.56849	1.99424	-0.02581
C	-1.18867	3.32663	-0.05756
O	-2.44135	3.4417	-0.09109
C	-0.30234	4.58598	-0.04976
H	0.29073	4.59921	0.84074
H	0.33963	4.57562	-0.90572
H	-0.92156	5.45812	-0.07888

Sum of electronic and zero-point Energies=-3202.137946
Sum of electronic and thermal Energies= -3202.124812
Sum of electronic and thermal Enthalpies= -3202.123868
Sum of electronic and thermal Free Energies= -3202.178090

5-BrIII-OTf

C	-2.93446	-1.10859	0.00015
C	-2.52243	0.21846	0.00015
C	-1.16116	0.50593	0.
C	-0.29395	-0.55644	-0.00016
C	-0.62935	-1.88835	-0.00026
C	-2.00137	-2.14559	-0.00009
H	-3.99148	-1.34315	0.0003
H	-3.22289	1.04544	0.00026
H	0.12474	-2.66268	-0.00066
H	-2.33707	-3.17535	-0.00015
Br	1.57797	0.06966	0.00005
C	-0.59322	1.89656	-0.00007
O	-1.26974	2.88566	0.00007
N	0.74447	1.89146	-0.00025
C	1.48431	3.16171	-0.00059
O	2.74271	3.1619	-0.00077
C	0.71675	4.4968	-0.00073
H	0.1032	4.55537	-0.87539
H	0.1002	4.55383	0.87191
H	1.41356	5.30881	0.00118
O	2.10706	-1.65082	0.00018
S	3.59545	-1.98101	0.65933
O	3.33619	-2.88048	1.67215
O	4.05757	-0.70294	0.93268
C	4.38132	-2.66853	-0.57901
F	4.64199	-1.73613	-1.51985
F	5.54162	-3.1985	-0.13705
F	3.61531	-3.64395	-1.1122

Sum of electronic and zero-point Energies=-4087.843723
Sum of electronic and thermal Energies= -4087.823799
Sum of electronic and thermal Enthalpies= -4087.822855
Sum of electronic and thermal Free Energies= -4087.895796

5-BrIII-OTs

C	-2.93446	-1.10859	0.00015
C	-2.52243	0.21846	0.00015
C	-1.16116	0.50593	0.
C	-0.29395	-0.55644	-0.00016
C	-0.62935	-1.88835	-0.00026
C	-2.00137	-2.14559	-0.00009
H	-3.99148	-1.34315	0.0003
H	-3.22289	1.04544	0.00026
H	0.12474	-2.66268	-0.00066
H	-2.33707	-3.17535	-0.00015
Br	1.57797	0.06966	0.00005
C	-0.59322	1.89656	-0.00007
O	-1.26974	2.88566	0.00007
N	0.74447	1.89146	-0.00025
C	1.48431	3.16171	-0.00059
O	2.74271	3.1619	-0.00077
C	0.71675	4.4968	-0.00073
H	0.1032	4.55537	-0.87539
H	0.1002	4.55383	0.87191
H	1.41356	5.30881	0.00118
O	2.10706	-1.65082	0.00018
S	3.59545	-1.98101	0.65933
O	3.33619	-2.88048	1.67215
O	4.05757	-0.70294	0.93268
C	4.39592	-2.6813	-0.60202
C	4.28664	-4.05865	-0.84619
C	5.20842	-1.91947	-1.45574
C	4.96058	-4.65559	-1.90495
H	3.6583	-4.67401	-0.17663
C	5.88653	-2.50822	-2.51555
H	5.31325	-0.83488	-1.27023
C	5.76625	-3.88445	-2.75313
H	4.86253	-5.73806	-2.07844
H	6.52159	-1.89344	-3.1714
C	6.47114	-4.51366	-3.89243
H	7.39648	-3.94511	-4.15734
H	6.75712	-5.56782	-3.65365
H	5.7994	-4.52751	-4.78867

Sum of electronic and zero-point Energies=-4021.016928
Sum of electronic and thermal Energies= -4020.994178
Sum of electronic and thermal Enthalpies= -4020.993233
Sum of electronic and thermal Free Energies= -4021.074152

5-BrIII-radical

C	-2.93446	-1.10859	0.00015
C	-2.52243	0.21846	0.00015
C	-1.16116	0.50593	0.
C	-0.29395	-0.55644	-0.00016
C	-0.62935	-1.88835	-0.00026
C	-2.00137	-2.14559	-0.00009
H	-3.99148	-1.34315	0.0003
H	-3.22289	1.04544	0.00026
H	0.12474	-2.66268	-0.00066
H	-2.33707	-3.17535	-0.00015
Br	1.57797	0.06966	0.00005
C	-0.59322	1.89656	-0.00007
O	-1.26974	2.88566	0.00007
N	0.74447	1.89146	-0.00025
C	1.48431	3.16171	-0.00059
O	2.74271	3.1619	-0.00077
C	0.71675	4.4968	-0.00073
H	0.1032	4.55537	-0.87539
H	0.1002	4.55383	0.87191
H	1.41356	5.30881	0.00118

Sum of electronic and zero-point Energies=-3126.353531
Sum of electronic and thermal Energies= -3126.342246
Sum of electronic and thermal Enthalpies= -3126.341302
Sum of electronic and thermal Free Energies= -3126.393078

5-BrIII-SCF3

C	2.78872	2.53347	-0.0787
C	3.18535	1.22372	-0.31382
C	2.27986	0.18067	-0.13941
C	1.0132	0.52339	0.25857
C	0.55417	1.79211	0.51366
C	1.48729	2.81374	0.3311
H	3.49368	3.34408	-0.21405
H	4.18916	0.96587	-0.63234
H	-0.45947	1.99472	0.82838
H	1.17977	3.83591	0.514
C	2.65512	-1.26809	-0.37758
O	3.76604	-1.5693	-0.73478
F	-3.93551	0.92128	-0.45801
F	-2.96359	-0.85487	-1.19914
F	-1.93008	1.02411	-1.25123
C	-2.77223	0.27822	-0.52401
S	-2.16147	-0.00054	1.15757
Br	-0.14178	-1.121	0.46359
N	1.63581	-2.10231	-0.14407
C	1.81673	-3.5507	-0.31822
O	0.82469	-4.32341	-0.2697
C	3.22512	-4.12441	-0.56086
H	3.58501	-3.79549	-1.51332
H	3.88663	-3.78293	0.20771
H	3.18228	-5.19345	-0.54555

Sum of electronic and zero-point Energies=-3862.191279
Sum of electronic and thermal Energies= -3862.173937
Sum of electronic and thermal Enthalpies= -3862.172993
Sum of electronic and thermal Free Energies= -3862.239059

5-ClIII-Br

C	-2.93446	-1.10859	0.00015
C	-2.52243	0.21846	0.00015
C	-1.16116	0.50593	0.
C	-0.29395	-0.55644	-0.00016
C	-0.62935	-1.88835	-0.00026
C	-2.00137	-2.14559	-0.00009
H	-3.99148	-1.34315	0.0003
H	-3.22289	1.04544	0.00026
H	0.12474	-2.66268	-0.00066
H	-2.33707	-3.17535	-0.00015
C	-0.59322	1.89656	-0.00007
O	-1.26974	2.88566	0.00007
N	0.74447	1.89146	-0.00025
C	1.48431	3.16171	-0.00059
O	2.74271	3.1619	-0.00077
C	0.71675	4.4968	-0.00073
H	0.1032	4.55537	-0.87539
H	0.1002	4.55383	0.87191
H	1.41356	5.30881	0.00118
Br	2.14787	-1.78352	0.00019
Cl	1.57797	0.06966	0.00005

Sum of electronic and zero-point Energies=-3586.525017
Sum of electronic and thermal Energies= -3586.511769
Sum of electronic and thermal Enthalpies= -3586.510824
Sum of electronic and thermal Free Energies= -3586.566738

5-ClIII-CCH

C	-0.12242	3.85594	0.
C	-1.22379	3.01699	0.00002
C	-1.03566	1.64264	0.00001
C	0.25381	1.14434	-0.00001
C	1.37222	1.94558	-0.00003
C	1.15994	3.32056	-0.00002
H	-0.25826	4.92824	0.00001
H	-2.23893	3.39099	0.00004
H	2.367	1.52892	-0.00005

H	2.01967	3.9763	-0.00004
C	-2.17681	0.68988	0.00002
O	-3.33648	1.02873	0.00008
N	-1.68975	-0.59082	-0.00004
C	-2.36863	-1.80677	0.
O	-1.72138	-2.82636	0.00006
C	-3.86977	-1.77147	-0.00007
H	-4.23289	-1.23044	0.87185
H	-4.23286	-1.23054	-0.87205
H	-4.22553	-2.7968	0.
C	2.22585	-0.93536	0.00001
C	3.42398	-1.0212	0.00002
H	4.49124	-1.09766	0.00004
Cl	0.32073	-0.79887	-0.00002

Sum of electronic and zero-point Energies=-1089.011730
Sum of electronic and thermal Energies= -1088.997672
Sum of electronic and thermal Enthalpies= -1088.996728
Sum of electronic and thermal Free Energies= -1089.053608

5-ClIII-CF3

C	-2.69011	2.28973	0.00005
C	-2.95457	0.92757	0.0001
C	-1.91235	-0.00045	0.00001
C	-0.64991	0.52412	-0.00006
C	-0.30538	1.85846	-0.00013
C	-1.37667	2.75141	-0.00009
H	-3.50769	2.99983	0.00013
H	-3.96181	0.52596	0.00018
H	0.70804	2.22727	-0.00024
H	-1.16527	3.81354	-0.00016
C	-2.19866	-1.51874	-0.00005
O	-3.35993	-1.86798	0.00015
C	2.40402	0.26982	0.00001
F	3.4394	-0.54682	0.00069
F	2.44547	1.03228	1.07774
F	2.44614	1.03133	-1.07837
N	-1.11298	-2.22208	-0.00038
C	-1.18829	-3.69015	-0.0004
O	-0.13487	-4.37853	-0.00075
C	-2.56105	-4.3881	-0.00001
H	-3.10734	-4.10011	0.87379
H	-3.10783	-4.10012	-0.87351
H	-2.42178	-5.44899	-0.00004
Cl	0.77117	-0.94889	0.00007

Sum of electronic and zero-point Energies=-1349.964292
Sum of electronic and thermal Energies= -1349.948711
Sum of electronic and thermal Enthalpies= -1349.947767
Sum of electronic and thermal Free Energies= -1350.009248

5-ClIII-CH3

C	-2.69011	2.28973	0.00005
C	-2.95457	0.92757	0.0001
C	-1.91235	-0.00045	0.00001
C	-0.64991	0.52412	-0.00006
C	-0.30538	1.85846	-0.00013
C	-1.37667	2.75141	-0.00009
H	-3.50769	2.99983	0.00013
H	-3.96181	0.52596	0.00018
H	0.70804	2.22727	-0.00024
H	-1.16527	3.81354	-0.00016
C	-2.19866	-1.51874	-0.00005
O	-3.35993	-1.86798	0.00015
C	2.40402	0.26982	0.00001
N	-1.11298	-2.22208	-0.00038
C	-1.18829	-3.69015	-0.0004
O	-0.13487	-4.37853	-0.00075
C	-2.56105	-4.3881	-0.00001
H	-3.10734	-4.10011	0.87379
H	-3.10783	-4.10012	-0.87351
H	-2.42178	-5.44899	-0.00004
H	2.4376	0.8875	0.87308
H	3.24415	-0.39281	0.00056
H	2.43814	0.88672	-0.87359
Cl	0.77117	-0.94889	0.00007

Sum of electronic and zero-point Energies=-1052.185886
Sum of electronic and thermal Energies= -1052.172422
Sum of electronic and thermal Enthalpies= -1052.171478
Sum of electronic and thermal Free Energies= -1052.226779

5-ClIII-CHCH2

C	-2.95217	-2.14162	0.00011
C	-1.58097	-2.33556	0.00027
C	-0.70784	-1.25544	0.00005
C	-1.25578	0.01702	-0.00018
C	-2.61783	0.24712	-0.00043
C	-3.46674	-0.85373	-0.00029
H	-3.62112	-2.99094	0.00024
H	-1.13619	-3.32193	0.00053
H	-3.03887	1.23823	-0.00073
H	-4.53556	-0.6903	-0.00052
C	0.78828	-1.49791	-0.00013
O	1.21495	-2.64048	0.00052
C	-1.34728	2.96144	0.00136
N	1.41303	-0.31693	-0.00121
C	2.76821	-0.07887	-0.00035
O	3.16153	1.0733	-0.00041
C	3.72445	-1.24393	0.00076
H	3.54599	-1.87415	0.87084
H	3.54678	-1.87522	-0.86868
H	4.73956	-0.85815	0.00104
H	-1.94734	2.91361	0.90356
C	-0.4665	4.2247	0.00257
H	-1.09003	5.09424	0.00131
H	0.15274	4.22859	-0.87002
Cl	0.00294	1.50101	-0.0002

Sum of electronic and zero-point Energies=-1090.251449
Sum of electronic and thermal Energies= -1090.237119
Sum of electronic and thermal Enthalpies= -1090.236175
Sum of electronic and thermal Free Energies= -1090.293988

5-ClIII-Cl

C	2.52236	2.11339	-0.00003
C	2.66085	0.73134	-0.00002
C	1.52519	-0.07221	-0.00001
C	0.30752	0.56194	0.00001
C	0.09823	1.91869	0.00008
C	1.25733	2.69731	0.00004
H	3.40253	2.74383	-0.00006
H	3.62846	0.24255	-0.00002
H	-0.89074	2.35437	0.00019
H	1.15606	3.77559	0.00007
C	1.58381	-1.57491	-0.00003
O	2.61636	-2.18619	-0.00011
Cl	-2.94445	0.85661	-0.00013
N	0.36728	-2.13124	-0.00002
C	0.2283	-3.59465	-0.00005
O	-0.91402	-4.12253	-0.00004
C	1.48498	-4.4848	-0.00011
H	2.06652	-4.28081	-0.87481
H	2.06864	-4.27792	0.87249
H	1.19292	-5.51417	0.00195
Cl	-0.99381	-0.62303	0.00007

Sum of electronic and zero-point Energies=-1472.522854
Sum of electronic and thermal Energies= -1472.509900
Sum of electronic and thermal Enthalpies= -1472.508956
Sum of electronic and thermal Free Energies= -1472.563283

5-ClIII-CN

C	2.38355	2.16507	-0.00011
C	2.54579	0.78572	-0.00018
C	1.42977	-0.04976	-0.00014
C	0.21531	0.57735	0.00019
C	-0.03278	1.92761	0.00009
C	1.11008	2.72995	0.00012
H	3.25324	2.81011	-0.00022
H	3.52047	0.31084	-0.00021
H	-1.02602	2.35531	0.00032
H	0.98659	3.8058	0.00018
C	1.54327	-1.57372	0.00002
O	2.63468	-2.0876	0.00015
C	-2.68934	0.59238	0.00006
N	-3.53721	1.37008	0.00019
N	0.3447	-2.16775	0.00037
C	0.25148	-3.6348	0.0001
O	-0.87381	-4.19808	0.00054
C	1.53533	-4.48528	-0.00073
H	2.11082	-4.26153	-0.87461
H	2.11166	-4.26197	0.8727
H	1.27555	-5.52327	-0.00086
Cl	-1.44146	-0.64875	-0.00014

Sum of electronic and zero-point Energies=-1105.118303
Sum of electronic and thermal Energies= -1105.104421
Sum of electronic and thermal Enthalpies= -1105.103477
Sum of electronic and thermal Free Energies= -1105.159789

5-ClIII-F

C	-2.93446	-1.10859	0.00015
C	-2.52243	0.21846	0.00015
C	-1.16116	0.50593	0.
C	-0.29395	-0.55644	-0.00016
C	-0.62935	-1.88835	-0.00026
C	-2.00137	-2.14559	-0.00009
H	-3.99148	-1.34315	0.0003
H	-3.22289	1.04544	0.00026
H	0.12474	-2.66268	-0.00066
H	-2.33707	-3.17535	-0.00015
C	-0.59322	1.89656	-0.00007
O	-1.26974	2.88566	0.00007
F	2.14787	-1.78352	0.00019
N	0.74447	1.89146	-0.00025
C	1.48431	3.16171	-0.00059
O	2.74271	3.1619	-0.00077
C	0.71675	4.4968	-0.00073
H	0.1032	4.55537	-0.87539
H	0.1002	4.55383	0.87191
H	1.41356	5.30881	0.00118

Cl	1.37517	0.00183	0.00002

Sum of electronic and zero-point Energies=-1112.157732
Sum of electronic and thermal Energies= -1112.145402
Sum of electronic and thermal Enthalpies= -1112.144458
Sum of electronic and thermal Free Energies= -1112.196846

5-ClIII-N3

C	-3.76213	0.02977	-0.00014
C	-2.84384	1.06556	-0.00019
C	-1.48964	0.77109	-0.00006
C	-1.08508	-0.54967	0.00011
C	-1.96828	-1.60848	0.00016
C	-3.3221	-1.28837	0.00003
H	-4.82155	0.24379	-0.00023
H	-3.1393	2.10611	-0.00033
H	-1.62909	-2.62933	0.00028
H	-4.04043	-2.09648	0.00006
C	-0.43163	1.81269	-0.00011
O	-0.64341	3.00249	-0.00027
N	0.77413	1.17274	0.00005
C	2.07139	1.67971	0.0001
O	2.9976	0.90658	0.00028
C	2.22567	3.17394	-0.00007
H	1.73528	3.60266	-0.87234
H	1.73515	3.60287	0.87203
H	3.28768	3.39832	-0.00001
N	0.5044	-2.7135	-0.05942
N	0.02842	-3.42505	2.12168
N	0.25753	-3.0723	1.07661
Cl	0.69414	-0.74428	0.00027

Sum of electronic and zero-point Energies=-1176.481425
Sum of electronic and thermal Energies= -1176.466811
Sum of electronic and thermal Enthalpies= -1176.465867
Sum of electronic and thermal Free Energies= -1176.525340

5-ClIII-NH2

C	-0.12242	3.85594	0.
C	-1.22379	3.01699	0.00002
C	-1.03566	1.64264	0.00001
C	0.25381	1.14434	-0.00001
C	1.37222	1.94558	-0.00003
C	1.15994	3.32056	-0.00002
H	-0.25826	4.92824	0.00001
H	-2.23893	3.39099	0.00004
H	2.367	1.52892	-0.00005
H	2.01967	3.9763	-0.00004
C	-2.17681	0.68988	0.00002
O	-3.33648	1.02873	0.00008
N	-1.68975	-0.59082	-0.00004
C	-2.36863	-1.80677	0.
O	-1.72138	-2.82636	0.00006
C	-3.86977	-1.77147	-0.00007
N	2.15603	-0.93035	0.00001
H	-4.22672	-1.23963	0.85704
H	-4.2267	-1.23972	-0.85724
H	-4.22052	-2.78235	0.
H	2.45492	-1.42281	0.81741
H	2.45472	-1.42594	-0.81558
Cl	0.32073	-0.79887	-0.00002

Sum of electronic and zero-point Energies=-1068.225524
Sum of electronic and thermal Energies= -1068.212281
Sum of electronic and thermal Enthalpies= -1068.211337
Sum of electronic and thermal Free Energies= -1068.265820

5-ClIII-NHAc

C	1.63714	3.63895	-0.00018
C	2.3238	2.43747	-0.00017
C	1.61133	1.2471	-0.00002
C	0.22981	1.28449	0.0001
C	-0.48683	2.46466	0.00011
C	0.24673	3.64566	-0.00004
H	2.18007	4.57361	-0.0003
H	3.40428	2.38576	-0.00027
H	-1.56292	2.46625	0.00019
H	-0.2871	4.58611	-0.00005
C	2.28255	-0.07898	0.00003
O	3.48193	-0.22651	-0.0001
C	-3.34264	-0.4131	0.00002
O	-3.14384	-1.60602	0.0004
C	-4.71944	0.20482	-0.00076
H	-4.83446	0.83892	0.87706
H	-4.83535	0.83366	-0.88228
H	-5.46909	-0.57976	0.0018
N	1.32578	-1.06142	0.0005
C	1.48423	-2.44873	-0.00008
O	0.49228	-3.13523	-0.00021
C	2.88066	-2.99879	-0.00053
H	3.42605	-2.64223	-0.8724
H	3.42669	-2.64251	0.87103
H	2.80888	-4.08173	-0.00075
N	-2.36209	0.49681	0.
H	-2.67317	1.32211	0.47129
Cl	-0.64541	-0.62444	0.00033

Sum of electronic and zero-point Energies=-1220.844901
Sum of electronic and thermal Energies= -1220.828273
Sum of electronic and thermal Enthalpies= -1220.827329
Sum of electronic and thermal Free Energies= -1220.890491

5-ClIII-OCF3

C	2.67388	2.50582	-0.08055
C	3.07538	1.1861	-0.2436
C	2.13864	0.16777	-0.09614
C	0.85091	0.5348	0.20224
C	0.38735	1.81389	0.38416
C	1.35008	2.81324	0.23105
H	3.39497	3.30541	-0.19349
H	4.09694	0.91345	-0.48175
H	-0.64249	2.02395	0.63
H	1.04878	3.8455	0.35987
C	2.46298	-1.28967	-0.23514
O	3.55405	-1.70721	-0.49333
F	-3.25665	-0.89735	-0.63674
F	-2.19308	0.86999	-1.26105
F	-3.78459	1.02948	0.19587
C	-2.75499	0.26941	-0.18023
O	-1.90316	0.11538	0.81056
N	1.39299	-2.066	-0.03049
C	1.5195	-3.52829	-0.11181
O	0.51575	-4.26265	0.07995
C	2.88509	-4.1637	-0.43278
H	3.22204	-3.82107	-1.3888
H	3.59449	-3.8824	0.31725
H	2.78758	-5.22911	-0.44979
Cl	-0.16269	-0.89665	0.34766

Sum of electronic and zero-point Energies=-1425.211686
Sum of electronic and thermal Energies= -1425.195473
Sum of electronic and thermal Enthalpies= -1425.194529
Sum of electronic and thermal Free Energies= -1425.257451

5-ClIII-OCH3

C	2.67388	2.50582	-0.08055
C	3.07538	1.1861	-0.2436
C	2.13864	0.16777	-0.09614
C	0.85091	0.5348	0.20224
C	0.38735	1.81389	0.38416
C	1.35008	2.81324	0.23105
H	3.39497	3.30541	-0.19349
H	4.09694	0.91345	-0.48175
H	-0.64249	2.02395	0.63
H	1.04878	3.8455	0.35987
C	2.46298	-1.28967	-0.23514
O	3.55405	-1.70721	-0.49333
C	-2.75499	0.26941	-0.18023
O	-1.90316	0.11538	0.81056
N	1.39299	-2.066	-0.03049
C	1.5195	-3.52829	-0.11181
O	0.51575	-4.26265	0.07995
C	2.88509	-4.1637	-0.43278
H	3.22204	-3.82107	-1.3888
H	3.59449	-3.8824	0.31725
H	2.78758	-5.22911	-0.44979
H	-2.3123	0.74256	-1.03173
H	-3.5809	0.87912	0.12146
H	-3.15272	-0.65563	-0.54216
Cl	-0.16269	-0.89665	0.34766

Sum of electronic and zero-point Energies=-1127.377511
Sum of electronic and thermal Energies= -1127.363148
Sum of electronic and thermal Enthalpies= -1127.362204
Sum of electronic and thermal Free Energies= -1127.419313

5-ClIII-OCOCF3

C	3.22289	2.62188	0.00859
C	3.68898	1.31361	0.00651
C	2.77394	0.26568	0.00105
C	1.43871	0.58809	-0.00237
C	0.91134	1.85792	-0.00002
C	1.85448	2.88734	0.0057
H	3.92673	3.44448	0.01292
H	4.74543	1.07154	0.00947
H	-0.15229	2.04153	-0.00164
H	1.50103	3.91113	0.00798
C	3.17219	-1.17944	0.00108
O	4.30614	-1.55945	0.00406
C	-2.41859	-0.53165	0.0117
O	-2.5234	-1.72768	0.04512
O	-1.32746	0.16971	-0.02174
C	-3.65945	0.39701	0.00003
F	-4.78188	-0.30037	0.05516
F	-3.67375	1.13597	-1.11316
F	-3.62623	1.23022	1.0443
N	2.11286	-1.9963	-0.00187
C	2.30819	-3.45326	-0.00066
O	1.31471	-4.22563	-0.00342
C	3.73351	-4.03639	0.00401
H	4.25728	-3.70507	-0.86822
H	4.25194	-3.70414	0.87908

H	3.68162	-5.10513	0.00442
Cl	0.47279	-0.88316	-0.00796

Sum of electronic and zero-point Energies=-1538.531873
Sum of electronic and thermal Energies= -1538.513404
Sum of electronic and thermal Enthalpies= -1538.512460
Sum of electronic and thermal Free Energies= -1538.584863

5-ClIII-OCOCH3

C	-3.27335	-0.12312	0.00031
C	-2.49827	1.03393	0.00063
C	-1.12547	0.91881	0.00047
C	-0.52508	-0.32717	-0.00003
C	-1.26275	-1.49007	-0.00055
C	-2.64893	-1.37174	-0.00039
H	-2.95749	2.0128	0.00095
H	-0.78942	-2.45569	-0.00113
H	-3.23393	-2.27869	-0.00088
C	-0.21095	2.08265	0.00052
O	-0.55325	3.23915	0.00096
C	2.55067	-2.61103	0.00054
O	3.49586	-1.8573	0.00159
O	1.2934	-2.21707	-0.00033
C	2.69052	-4.11664	-0.00021
H	2.19707	-4.52965	0.87846
H	2.20001	-4.52839	-0.88112
H	3.74284	-4.38259	0.00131
N	1.07445	1.59595	0.00007
C	2.29838	2.28421	-0.00058
O	3.31754	1.64842	-0.00131
C	2.23544	3.78431	-0.00059
H	1.69066	4.14082	-0.87275
H	1.69169	4.14088	0.87219
H	3.25628	4.15293	-0.00123
Cl	1.2554	-0.24261	-0.00002
H	-4.3339	0.01879	0.00046

Sum of electronic and zero-point Energies=-1240.737118
Sum of electronic and thermal Energies= -1240.720638
Sum of electronic and thermal Enthalpies= -1240.719693
Sum of electronic and thermal Free Energies= -1240.783838

5-ClIII-OCOPh

C	1.63714	3.63895	-0.00018
C	2.3238	2.43747	-0.00017
C	1.61133	1.2471	-0.00002
C	0.22981	1.28449	0.0001
C	-0.48683	2.46466	0.00011
C	0.24673	3.64566	-0.00004
H	2.18007	4.57361	-0.0003
H	3.40428	2.38576	-0.00027
H	-1.56292	2.46625	0.00019
H	-0.2871	4.58611	-0.00005
C	2.28255	-0.07898	0.00003
O	3.48193	-0.22651	-0.0001
C	-3.34264	-0.4131	0.00002
O	-3.14384	-1.60602	0.00235
O	-2.37521	0.48463	-0.00236
N	1.32578	-1.06142	0.0005
C	1.48423	-2.44873	-0.00008
O	0.49228	-3.13523	-0.00021
C	2.88066	-2.99879	-0.00053
H	3.42605	-2.64223	-0.8724
H	3.42669	-2.64251	0.87103
H	2.80888	-4.08173	-0.00075
C	-4.74763	0.21747	-0.00077
C	-5.87874	-0.59926	0.00149
C	-4.88939	1.60507	-0.00365
C	-7.15129	-0.02843	0.0002
H	-5.76664	-1.69319	0.00298
C	-6.16225	2.17624	-0.00395
H	-3.998	2.24893	-0.00526
C	-7.29314	1.35976	-0.00216
H	-8.04289	-0.67213	0.00136
H	-6.27371	3.27033	-0.0058
H	-8.2966	1.80961	-0.00312
Cl	-0.50371	-0.31537	0.00029

Sum of electronic and zero-point Energies=-1432.415741
Sum of electronic and thermal Energies= -1432.397233
Sum of electronic and thermal Enthalpies= -1432.396288
Sum of electronic and thermal Free Energies= -1432.464563

5-ClIII-OH

C	2.85263	-1.32247	0.00438
C	2.53897	0.03132	0.02066
C	1.20519	0.42867	0.01343
C	0.26118	-0.56546	0.00447
C	0.49803	-1.91871	-0.02637
C	1.8446	-2.28523	-0.02237
H	3.88939	-1.63508	0.00637
H	3.29795	0.80511	0.03341
H	-0.31078	-2.63443	-0.06185
H	2.09873	-3.33788	-0.04592
C	0.76361	1.8772	0.00979
O	1.56652	2.77539	0.03319
O	-2.35941	-1.6203	-0.02187
H	-2.69235	-1.77691	0.86934
N	-0.56849	1.99424	-0.02581
C	-1.18867	3.32663	-0.05756
O	-2.44135	3.4417	-0.09109
C	-0.30234	4.58598	-0.04976
H	0.29073	4.59921	0.84074
H	0.33963	4.57562	-0.90572
H	-0.92156	5.45812	-0.07888
Cl	-1.3738	0.08565	-0.01763

Sum of electronic and zero-point Energies=-1088.111338
Sum of electronic and thermal Energies= -1088.098328
Sum of electronic and thermal Enthalpies= -1088.097383
Sum of electronic and thermal Free Energies= -1088.151220

5-ClIII-OTf

C	-2.93446	-1.10859	0.00015
C	-2.52243	0.21846	0.00015
C	-1.16116	0.50593	0.
C	-0.29395	-0.55644	-0.00016
C	-0.62935	-1.88835	-0.00026
C	-2.00137	-2.14559	-0.00009
H	-3.99148	-1.34315	0.0003
H	-3.22289	1.04544	0.00026
H	0.12474	-2.66268	-0.00066
H	-2.33707	-3.17535	-0.00015
C	-0.59322	1.89656	-0.00007
O	-1.26974	2.88566	0.00007
N	0.74447	1.89146	-0.00025
C	1.48431	3.16171	-0.00059
O	2.74271	3.1619	-0.00077
C	0.71675	4.4968	-0.00073
H	0.1032	4.55537	-0.87539
H	0.1002	4.55383	0.87191
H	1.41356	5.30881	0.00118
O	2.10706	-1.65082	0.00018
S	3.59545	-1.98101	0.65933
O	3.33619	-2.88048	1.67215
O	4.05757	-0.70294	0.93268
C	4.38132	-2.66853	-0.57901
F	4.64199	-1.73613	-1.51985
F	5.54162	-3.1985	-0.13705
F	3.61531	-3.64395	-1.1122
Cl	1.57797	0.06966	0.00005

Sum of electronic and zero-point Energies=-1973.822369
Sum of electronic and thermal Energies= -1973.802727
Sum of electronic and thermal Enthalpies= -1973.801783
Sum of electronic and thermal Free Energies= -1973.874258

5-ClIII-OTs

C	-2.93446	-1.10859	0.00015
C	-2.52243	0.21846	0.00015
C	-1.16116	0.50593	0.
C	-0.29395	-0.55644	-0.00016
C	-0.62935	-1.88835	-0.00026
C	-2.00137	-2.14559	-0.00009
H	-3.99148	-1.34315	0.0003
H	-3.22289	1.04544	0.00026
H	0.12474	-2.66268	-0.00066
H	-2.33707	-3.17535	-0.00015
C	-0.59322	1.89656	-0.00007
O	-1.26974	2.88566	0.00007
N	0.74447	1.89146	-0.00025
C	1.48431	3.16171	-0.00059
O	2.74271	3.1619	-0.00077
C	0.71675	4.4968	-0.00073
H	0.1032	4.55537	-0.87539
H	0.1002	4.55383	0.87191
H	1.41356	5.30881	0.00118
O	2.10706	-1.65082	0.00018
S	3.59545	-1.98101	0.65933
O	3.33619	-2.88048	1.67215
O	4.05757	-0.70294	0.93268
C	4.39592	-2.6813	-0.60202
C	4.28664	-4.05865	-0.84619
C	5.20842	-1.91947	-1.45574
C	4.96058	-4.65559	-1.90495
H	3.6583	-4.67401	-0.17663
C	5.88653	-2.50822	-2.51555
H	5.31325	-0.83488	-1.27023
C	5.76625	-3.88445	-2.75313
H	4.86253	-5.73806	-2.07844
H	6.52159	-1.89344	-3.1714
C	6.47114	-4.51366	-3.89243
H	7.39648	-3.94511	-4.15734
H	6.75712	-5.56782	-3.65365
H	5.7994	-4.52751	-4.78867
Cl	1.57797	0.06966	0.00005

Sum of electronic and zero-point Energies=-1906.993178
Sum of electronic and thermal Energies= -1906.970727
Sum of electronic and thermal Enthalpies= -1906.969782
Sum of electronic and thermal Free Energies= -1907.048905

5-ClIII-radical

C	-2.93446	-1.10859	0.00015
C	-2.52243	0.21846	0.00015
C	-1.16116	0.50593	0.
C	-0.29395	-0.55644	-0.00016
C	-0.62935	-1.88835	-0.00026
C	-2.00137	-2.14559	-0.00009
H	-3.99148	-1.34315	0.0003
H	-3.22289	1.04544	0.00026
H	0.12474	-2.66268	-0.00066
H	-2.33707	-3.17535	-0.00015
C	-0.59322	1.89656	-0.00007
O	-1.26974	2.88566	0.00007
N	0.74447	1.89146	-0.00025
C	1.48431	3.16171	-0.00059
O	2.74271	3.1619	-0.00077
C	0.71675	4.4968	-0.00073
H	0.1032	4.55537	-0.87539
H	0.1002	4.55383	0.87191
H	1.41356	5.30881	0.00118
Cl	1.37517	0.00183	0.00002

Sum of electronic and zero-point Energies=-1012.356905
Sum of electronic and thermal Energies= -1012.345675
Sum of electronic and thermal Enthalpies= -1012.344730
Sum of electronic and thermal Free Energies= -1012.396268

5-ClIII-SCF3

C	2.78872	2.53347	-0.0787
C	3.18535	1.22372	-0.31382
C	2.27986	0.18067	-0.13941
C	1.0132	0.52339	0.25857
C	0.55417	1.79211	0.51366
C	1.48729	2.81374	0.3311
H	3.49368	3.34408	-0.21405
H	4.18916	0.96587	-0.63234
H	-0.45947	1.99472	0.82838
H	1.17977	3.83591	0.514
C	2.65512	-1.26809	-0.37758
O	3.76604	-1.5693	-0.73478
F	-3.93551	0.92128	-0.45801
F	-2.96359	-0.85487	-1.19914
F	-1.93008	1.02411	-1.25123
C	-2.77223	0.27822	-0.52401
S	-2.16147	-0.00054	1.15757
N	1.63581	-2.10231	-0.14407
C	1.81673	-3.5507	-0.31822
O	0.82469	-4.32341	-0.2697
C	3.22512	-4.12441	-0.56086
H	3.58501	-3.79549	-1.51332
H	3.88663	-3.78293	0.20771
H	3.18228	-5.19345	-0.54555
Cl	0.00684	-0.90941	0.43721

Sum of electronic and zero-point Energies=-1748.165481
Sum of electronic and thermal Energies= -1748.148236
Sum of electronic and thermal Enthalpies= -1748.147291
Sum of electronic and thermal Free Energies= -1748.213260

5-IIII-Br

C	-2.93446	-1.10859	0.00015
C	-2.52243	0.21846	0.00015
C	-1.16116	0.50593	0.
C	-0.29395	-0.55644	-0.00016
C	-0.62935	-1.88835	-0.00026
C	-2.00137	-2.14559	-0.00009
H	-3.99148	-1.34315	0.0003
H	-3.22289	1.04544	0.00026
H	0.12474	-2.66268	-0.00066
H	-2.33707	-3.17535	-0.00015
C	-0.59322	1.89656	-0.00007
O	-1.26974	2.88566	0.00007
N	0.74447	1.89146	-0.00025
C	1.48431	3.16171	-0.00059
O	2.74271	3.1619	-0.00077
C	0.71675	4.4968	-0.00073
H	0.1032	4.55537	-0.87539
H	0.1002	4.55383	0.87191
H	1.41356	5.30881	0.00118
Br	2.14787	-1.78352	0.00019
I	1.57797	0.06966	0.00005

Sum of electronic and zero-point Energies=-3424.028542
Sum of electronic and thermal Energies= -3424.014955
Sum of electronic and thermal Enthalpies= -3424.014011
Sum of electronic and thermal Free Energies= -3424.071184

5-IIII-CCH

C	-0.12242	3.85594	0.
C	-1.22379	3.01699	0.00002
C	-1.03566	1.64264	0.00001
C	0.25381	1.14434	-0.00001
C	1.37222	1.94558	-0.00003
C	1.15994	3.32056	-0.00002
H	-0.25826	4.92824	0.00001
H	-2.23893	3.39099	0.00004
H	2.367	1.52892	-0.00005
H	2.01967	3.9763	-0.00004
C	-2.17681	0.68988	0.00002
O	-3.33648	1.02873	0.00008
N	-1.68975	-0.59082	-0.00004
C	-2.36863	-1.80677	0.
O	-1.72138	-2.82636	0.00006
C	-3.86977	-1.77147	-0.00007
H	-4.23289	-1.23044	0.87185
H	-4.23286	-1.23054	-0.87205
H	-4.22553	-2.7968	0.
C	2.22585	-0.93536	0.00001
C	3.42398	-1.0212	0.00002
H	4.49124	-1.09766	0.00004
I	0.32073	-0.79887	-0.00002

Sum of electronic and zero-point Energies= -926.506181
Sum of electronic and thermal Energies= -926.491768
Sum of electronic and thermal Enthalpies=-926.490824
Sum of electronic and thermal Free Energies= -926.548742

5-IIII-CF3

C	-2.69011	2.28973	0.00005
C	-2.95457	0.92757	0.0001
C	-1.91235	-0.00045	0.00001
C	-0.64991	0.52412	-0.00006
C	-0.30538	1.85846	-0.00013
C	-1.37667	2.75141	-0.00009
H	-3.50769	2.99983	0.00013
H	-3.96181	0.52596	0.00018
H	0.70804	2.22727	-0.00024
H	-1.16527	3.81354	-0.00016
C	-2.19866	-1.51874	-0.00005
O	-3.35993	-1.86798	0.00015
C	2.40402	0.26982	0.00001
F	3.4394	-0.54682	0.00069
F	2.44547	1.03228	1.07774
F	2.44614	1.03133	-1.07837
N	-1.11298	-2.22208	-0.00038
C	-1.18829	-3.69015	-0.0004
O	-0.13487	-4.37853	-0.00075
C	-2.56105	-4.3881	-0.00001
H	-3.10734	-4.10011	0.87379
H	-3.10783	-4.10012	-0.87351
H	-2.42178	-5.44899	-0.00004
I	0.77117	-0.94889	0.00007

Sum of electronic and zero-point Energies=-1187.446943
Sum of electronic and thermal Energies= -1187.430818
Sum of electronic and thermal Enthalpies= -1187.429874
Sum of electronic and thermal Free Energies= -1187.492467

5-IIII-CH3

C	-2.69011	2.28973	0.00005
C	-2.95457	0.92757	0.0001
C	-1.91235	-0.00045	0.00001
C	-0.64991	0.52412	-0.00006
C	-0.30538	1.85846	-0.00013
C	-1.37667	2.75141	-0.00009
H	-3.50769	2.99983	0.00013
H	-3.96181	0.52596	0.00018
H	0.70804	2.22727	-0.00024
H	-1.16527	3.81354	-0.00016
C	-2.19866	-1.51874	-0.00005
O	-3.35993	-1.86798	0.00015
C	2.40402	0.26982	0.00001
N	-1.11298	-2.22208	-0.00038
C	-1.18829	-3.69015	-0.0004
O	-0.13487	-4.37853	-0.00075
C	-2.56105	-4.3881	-0.00001
H	-3.10734	-4.10011	0.87379
H	-3.10783	-4.10012	-0.87351
H	-2.42178	-5.44899	-0.00004
H	2.4376	0.8875	0.87308
H	3.24415	-0.39281	0.00056
H	2.43814	0.88672	-0.87359
I	0.77117	-0.94889	0.00007

Sum of electronic and zero-point Energies= -889.657244
Sum of electronic and thermal Energies= -889.643345
Sum of electronic and thermal Enthalpies=-889.642401
Sum of electronic and thermal Free Energies= -889.699226

5-IIII-CHCH2

C	-2.95217	-2.14162	0.00011
C	-1.58097	-2.33556	0.00027
C	-0.70784	-1.25544	0.00005
C	-1.25578	0.01702	-0.00018
C	-2.61783	0.24712	-0.00043
C	-3.46674	-0.85373	-0.00029
H	-3.62112	-2.99094	0.00024
H	-1.13619	-3.32193	0.00053
H	-3.03887	1.23823	-0.00073
H	-4.53556	-0.6903	-0.00052
C	0.78828	-1.49791	-0.00013
O	1.21495	-2.64048	0.00052

C	-1.34728	2.96144	0.00136
N	1.41303	-0.31693	-0.00121
C	2.76821	-0.07887	-0.00035
O	3.16153	1.0733	-0.00041
C	3.72445	-1.24393	0.00076
H	3.54599	-1.87415	0.87084
H	3.54678	-1.87522	-0.86868
H	4.73956	-0.85815	0.00104
H	-1.94734	2.91361	0.90356
C	-0.4665	4.2247	0.00257
H	-1.09003	5.09424	0.00131
H	0.15274	4.22859	-0.87002
I	0.00294	1.50101	-0.0002

Sum of electronic and zero-point Energies= -927.726406
Sum of electronic and thermal Energies= -927.711604
Sum of electronic and thermal Enthalpies=-927.710660
Sum of electronic and thermal Free Energies= -927.769936

5-IIII-Cl

C	2.52236	2.11339	-0.00003
C	2.66085	0.73134	-0.00002
C	1.52519	-0.07221	-0.00001
C	0.30752	0.56194	0.00001
C	0.09823	1.91869	0.00008
C	1.25733	2.69731	0.00004
H	3.40253	2.74383	-0.00006
H	3.62846	0.24255	-0.00002
H	-0.89074	2.35437	0.00019
H	1.15606	3.77559	0.00007
C	1.58381	-1.57491	-0.00003
O	2.61636	-2.18619	-0.00011
Cl	-2.94445	0.85661	-0.00013
N	0.36728	-2.13124	-0.00002
C	0.2283	-3.59465	-0.00005
O	-0.91402	-4.12253	-0.00004
C	1.48498	-4.4848	-0.00011
H	2.06652	-4.28081	-0.87481
H	2.06864	-4.27792	0.87249
H	1.19292	-5.51417	0.00195
I	-1.2452	-0.85194	0.00008

Sum of electronic and zero-point Energies=-1310.027818
Sum of electronic and thermal Energies= -1310.014504
Sum of electronic and thermal Enthalpies= -1310.013560
Sum of electronic and thermal Free Energies= -1310.069319

5-IIII-CN

C	2.38355	2.16507	-0.00011
C	2.54579	0.78572	-0.00018
C	1.42977	-0.04976	-0.00014
C	0.21531	0.57735	0.00019
C	-0.03278	1.92761	0.00009
C	1.11008	2.72995	0.00012
H	3.25324	2.81011	-0.00022
H	3.52047	0.31084	-0.00021
H	-1.02602	2.35531	0.00032
H	0.98659	3.8058	0.00018
C	1.54327	-1.57372	0.00002
O	2.63468	-2.0876	0.00015
C	-2.68934	0.59238	0.00006
N	-3.53721	1.37008	0.00019
N	0.3447	-2.16775	0.00037
C	0.25148	-3.6348	0.0001
O	-0.87381	-4.19808	0.00054
C	1.53533	-4.48528	-0.00073
H	2.11082	-4.26153	-0.87461
H	2.11166	-4.26197	0.8727
H	1.27555	-5.52327	-0.00086
I	-1.2004	-0.88851	-0.00018

Sum of electronic and zero-point Energies= -942.620546
Sum of electronic and thermal Energies= -942.606305
Sum of electronic and thermal Enthalpies=-942.605361
Sum of electronic and thermal Free Energies= -942.662980

5-IIII-F

C	-2.93446	-1.10859	0.00015
C	-2.52243	0.21846	0.00015
C	-1.16116	0.50593	0.
C	-0.29395	-0.55644	-0.00016
C	-0.62935	-1.88835	-0.00026
C	-2.00137	-2.14559	-0.00009
H	-3.99148	-1.34315	0.0003
H	-3.22289	1.04544	0.00026
H	0.12474	-2.66268	-0.00066
H	-2.33707	-3.17535	-0.00015
C	-0.59322	1.89656	-0.00007
O	-1.26974	2.88566	0.00007
F	2.14787	-1.78352	0.00019
N	0.74447	1.89146	-0.00025
C	1.48431	3.16171	-0.00059
O	2.74271	3.1619	-0.00077
C	0.71675	4.4968	-0.00073
H	0.1032	4.55537	-0.87539
H	0.1002	4.55383	0.87191
H	1.41356	5.30881	0.00118
I	1.69761	0.10968	0.00006

Sum of electronic and zero-point Energies= -949.666410
Sum of electronic and thermal Energies= -949.653684
Sum of electronic and thermal Enthalpies=-949.652740
Sum of electronic and thermal Free Energies= -949.706572

5-IIII-N3

C	-2.93446	-1.10859	0.00015
C	-2.52243	0.21846	0.00015
C	-1.16116	0.50593	0.
C	-0.29395	-0.55644	-0.00016
C	-0.62935	-1.88835	-0.00026
C	-2.00137	-2.14559	-0.00009
H	-3.99148	-1.34315	0.0003
H	-3.22289	1.04544	0.00026
H	0.12474	-2.66268	-0.00066
H	-2.33707	-3.17535	-0.00015
C	-0.59322	1.89656	-0.00007
O	-1.26974	2.88566	0.00007
N	0.74447	1.89146	-0.00025
C	1.48431	3.16171	-0.00059
O	2.74271	3.1619	-0.00077
C	0.71675	4.4968	-0.00073
H	0.1032	4.55537	-0.87539
H	0.1002	4.55383	0.87191
H	1.41356	5.30881	0.00118
N	2.11882	-1.68906	0.00018
N	2.65816	-3.9473	0.79181
N	2.41713	-2.93233	0.46901
I	1.57797	0.06966	0.00005

Sum of electronic and zero-point Energies=-1013.982551
Sum of electronic and thermal Energies= -1013.967644
Sum of electronic and thermal Enthalpies= -1013.966699
Sum of electronic and thermal Free Energies= -1014.026796

5-IIII-NH2

C	-0.12242	3.85594	0.
C	-1.22379	3.01699	0.00002
C	-1.03566	1.64264	0.00001
C	0.25381	1.14434	-0.00001
C	1.37222	1.94558	-0.00003
C	1.15994	3.32056	-0.00002
H	-0.25826	4.92824	0.00001
H	-2.23893	3.39099	0.00004
H	2.367	1.52892	-0.00005
H	2.01967	3.9763	-0.00004
C	-2.17681	0.68988	0.00002
O	-3.33648	1.02873	0.00008
N	-1.68975	-0.59082	-0.00004
C	-2.36863	-1.80677	0.
O	-1.72138	-2.82636	0.00006
C	-3.86977	-1.77147	-0.00007
N	2.15603	-0.93035	0.00001
H	-4.22672	-1.23963	0.85704
H	-4.2267	-1.23972	-0.85724
H	-4.22052	-2.78235	0.
H	2.45492	-1.42281	0.81741
H	2.45472	-1.42594	-0.81558
I	0.32073	-0.79887	-0.00002

Sum of electronic and zero-point Energies= -905.720910
Sum of electronic and thermal Energies= -905.707321
Sum of electronic and thermal Enthalpies=-905.706377
Sum of electronic and thermal Free Energies= -905.762005

5-IIII-NHAc

C	1.63714	3.63895	-0.00018
C	2.3238	2.43747	-0.00017
C	1.61133	1.2471	-0.00002
C	0.22981	1.28449	0.0001
C	-0.48683	2.46466	0.00011
C	0.24673	3.64566	-0.00004
H	2.18007	4.57361	-0.0003
H	3.40428	2.38576	-0.00027
H	-1.56292	2.46625	0.00019
H	-0.2871	4.58611	-0.00005
C	2.28255	-0.07898	0.00003
O	3.48193	-0.22651	-0.0001
C	-3.34264	-0.4131	0.00002
O	-3.14384	-1.60602	0.0004
C	-4.71944	0.20482	-0.00076
H	-4.83446	0.83892	0.87706
H	-4.83535	0.83366	-0.88228
H	-5.46909	-0.57976	0.0018
N	1.32578	-1.06142	0.0005
C	1.48423	-2.44873	-0.00008
O	0.49228	-3.13523	-0.00021
C	2.88066	-2.99879	-0.00053
H	3.42605	-2.64223	-0.8724
H	3.42669	-2.64251	0.87103
H	2.80888	-4.08173	-0.00075
N	-2.36209	0.49681	0.
H	-2.67317	1.32211	0.47129

I -0.64541 -0.62444 0.00033
Sum of electronic and zero-point Energies=-1058.347578
Sum of electronic and thermal Energies= -1058.330391
Sum of electronic and thermal Enthalpies= -1058.329447
Sum of electronic and thermal Free Energies= -1058.394575

5-IIII-OCF3
C 2.67388 2.50582 -0.08055
C 3.07538 1.1861 -0.2436
C 2.13864 0.16777 -0.09614
C 0.85091 0.5348 0.20224
C 0.38735 1.81389 0.38416
C 1.35008 2.81324 0.23105
H 3.39497 3.30541 -0.19349
H 4.09694 0.91345 -0.48175
H -0.64249 2.02395 0.63
H 1.04878 3.8455 0.35987
C 2.46298 -1.28967 -0.23514
O 3.55405 -1.70721 -0.49333
F -3.25665 -0.89735 -0.63674
F -2.19308 0.86999 -1.26105
F -3.78459 1.02948 0.19587
C -2.75499 0.26941 -0.18023
O -1.90316 0.11538 0.81056
N 1.39299 -2.066 -0.03049
C 1.5195 -3.52829 -0.11181
O 0.51575 -4.26265 0.07995
C 2.88509 -4.1637 -0.43278
H 3.22204 -3.82107 -1.3888
H 3.59449 -3.8824 0.31725
H 2.78758 -5.22911 -0.44979
I -0.3585 -1.17318 0.37575
Sum of electronic and zero-point Energies=-1262.716054
Sum of electronic and thermal Energies= -1262.699397
Sum of electronic and thermal Enthalpies= -1262.698453
Sum of electronic and thermal Free Energies= -1262.762823

5-IIII-OCH3
C 2.67388 2.50582 -0.08055
C 3.07538 1.1861 -0.2436
C 2.13864 0.16777 -0.09614
C 0.85091 0.5348 0.20224
C 0.38735 1.81389 0.38416
C 1.35008 2.81324 0.23105
H 3.39497 3.30541 -0.19349
H 4.09694 0.91345 -0.48175
H -0.64249 2.02395 0.63
H 1.04878 3.8455 0.35987
C 2.46298 -1.28967 -0.23514
O 3.55405 -1.70721 -0.49333
C -2.75499 0.26941 -0.18023
O -1.90316 0.11538 0.81056
N 1.39299 -2.066 -0.03049
C 1.5195 -3.52829 -0.11181
O 0.51575 -4.26265 0.07995
C 2.88509 -4.1637 -0.43278
H 3.22204 -3.82107 -1.3888
H 3.59449 -3.8824 0.31725
H 2.78758 -5.22911 -0.44979
H -2.3123 0.74256 -1.03173
H -3.5809 0.87912 0.12146
H -3.15272 -0.65563 -0.54216
I -0.3585 -1.17318 0.37575
Sum of electronic and zero-point Energies= -964.879931
Sum of electronic and thermal Energies= -964.865060
Sum of electronic and thermal Enthalpies=-964.864116
Sum of electronic and thermal Free Energies= -964.922912

5-IIII-OCOCF3
C 3.22289 2.62188 0.00859
C 3.68898 1.31361 0.00651
C 2.77394 0.26568 0.00105
C 1.43871 0.58809 -0.00237
C 0.91134 1.85792 -0.00002
C 1.85448 2.88734 0.0057
H 3.92673 3.44448 0.01292
H 4.74543 1.07154 0.00947
H -0.15229 2.04153 -0.00164
H 1.50103 3.91113 0.00798
C 3.17219 -1.17944 0.00108
O 4.30614 -1.55945 0.00406
C -2.41859 -0.53165 0.0117
O -2.5234 -1.72768 0.04512
O -1.32746 0.16971 -0.02174
C -3.65945 0.39701 0.00003
F -4.78188 -0.30037 0.05516
F -3.67375 1.13597 -1.11316
F -3.62623 1.23022 1.0443
N 2.11286 -1.9963 -0.00187
C 2.30819 -3.45326 -0.00066
O 1.31471 -4.22563 -0.00342
C 3.73351 -4.03639 0.00401
H 4.25728 -3.70507 -0.86822
H 4.25194 -3.70414 0.87908
H 3.68162 -5.10513 0.00442
I 0.2862 -1.16738 -0.00904
Sum of electronic and zero-point Energies=-1376.037077
Sum of electronic and thermal Energies= -1376.018304
Sum of electronic and thermal Enthalpies= -1376.017360
Sum of electronic and thermal Free Energies= -1376.087865

5-IIII-OCOCH3
C 2.94595 2.24262 0.
C 3.14905 0.86891 -0.00023
C 2.05101 0.01452 -0.00014
C 0.80079 0.58421 0.00015
C 0.53351 1.93493 0.00041
C 1.65414 2.76631 0.00033
H 3.79558 2.9138 -0.00007
H 4.13833 0.42604 -0.00045
H -0.47532 2.31781 0.00073
H 1.50439 3.83907 0.00055
C 2.175 -1.48618 -0.00024
O 3.23627 -2.04801 -0.00049
C -3.20495 0.1778 0.00021
O -3.42588 -1.00956 0.00076
O -1.9799 0.68529 0.00005
C -4.26655 1.25034 -0.00099
H -4.14463 1.88403 0.87785
H -4.15011 1.8754 -0.88681
H -5.24737 0.78154 0.00398
N 0.98164 -2.0906 0.
C 0.90116 -3.55839 0.0001
O -0.20818 -4.13204 -0.15442
C 2.17881 -4.39694 0.18995
H 2.82647 -4.25531 -0.64992
H 2.67977 -4.08649 1.08301
H 1.91791 -5.43166 0.26866
I -0.68643 -0.89842 0.00013
Sum of electronic and zero-point Energies=-1078.246803
Sum of electronic and thermal Energies= -1078.229909
Sum of electronic and thermal Enthalpies= -1078.228965
Sum of electronic and thermal Free Energies= -1078.293422

5-IIII-OCOPh
C 1.63714 3.63895 -0.00018
C 2.3238 2.43747 -0.00017
C 1.61133 1.2471 -0.00002
C 0.22981 1.28449 0.0001
C -0.48683 2.46466 0.00011
C 0.24673 3.64566 -0.00004
H 2.18007 4.57361 -0.0003
H 3.40428 2.38576 -0.00027
H -1.56292 2.46625 0.00019
H -0.2871 4.58611 -0.00005
C 2.28255 -0.07898 0.00003
O 3.48193 -0.22651 -0.0001
C -3.34264 -0.4131 0.00002
O -3.14384 -1.60602 0.00235
O -2.37521 0.48463 -0.00236
N 1.32578 -1.06142 0.0005
C 1.48423 -2.44873 -0.00008
O 0.49228 -3.13523 -0.00021
C 2.88066 -2.99879 -0.00053
H 3.42605 -2.64223 -0.8724
H 3.42669 -2.64251 0.87103
H 2.80888 -4.08173 -0.00075
C -4.74763 0.21747 -0.00077
C -5.87874 -0.59926 0.00149
C -4.88939 1.60507 -0.00365
C -7.15129 -0.02843 0.0002
H -5.76664 -1.69319 0.00298
C -6.16225 2.17624 -0.00395
H -3.998 2.24893 -0.00526
C -7.29314 1.35976 -0.00216
H -8.04289 -0.67213 0.00136
H -6.27371 3.27033 -0.0058
H -8.2966 1.80961 -0.00312
I -0.64541 -0.62444 0.00033
Sum of electronic and zero-point Energies=-1269.925023
Sum of electronic and thermal Energies= -1269.905333
Sum of electronic and thermal Enthalpies= -1269.904389
Sum of electronic and thermal Free Energies= -1269.976500

5-IIII-OH
C 2.85263 -1.32247 0.00438
C 2.53897 0.03132 0.02066
C 1.20519 0.42867 0.01343
C 0.26118 -0.56546 0.00447
C 0.49803 -1.91871 -0.02637
C 1.8446 -2.28523 -0.02237
H 3.88939 -1.63508 0.00637
H 3.29795 0.80511 0.03341
H -0.31078 -2.63443 -0.06185
H 2.09873 -3.33788 -0.04592
C 0.76361 1.8772 0.00979

O	1.56652	2.77539	0.03319
O	-2.35941	-1.6203	-0.02187
H	-2.69235	-1.77691	0.86934
N	-0.56849	1.99424	-0.02581
C	-1.18867	3.32663	-0.05756
O	-2.44135	3.4417	-0.09109
C	-0.30234	4.58598	-0.04976
H	0.29073	4.59921	0.84074
H	0.33963	4.57562	-0.90572
H	-0.92156	5.45812	-0.07888
I	-1.68965	0.21143	-0.0219

Sum of electronic and zero-point Energies= -925.616848
Sum of electronic and thermal Energies= -925.603647
Sum of electronic and thermal Enthalpies=-925.602703
Sum of electronic and thermal Free Energies= -925.657390

5-IIII-OTf

C	-2.93446	-1.10859	0.00015
C	-2.52243	0.21846	0.00015
C	-1.16116	0.50593	0.
C	-0.29395	-0.55644	-0.00016
C	-0.62935	-1.88835	-0.00026
C	-2.00137	-2.14559	-0.00009
H	-3.99148	-1.34315	0.0003
H	-3.22289	1.04544	0.00026
H	0.12474	-2.66268	-0.00066
H	-2.33707	-3.17535	-0.00015
C	-0.59322	1.89656	-0.00007
O	-1.26974	2.88566	0.00007
N	0.74447	1.89146	-0.00025
C	1.48431	3.16171	-0.00059
O	2.74271	3.1619	-0.00077
C	0.71675	4.4968	-0.00073
H	0.1032	4.55537	-0.87539
H	0.1002	4.55383	0.87191
H	1.41356	5.30881	0.00118
O	2.10706	-1.65082	0.00018
S	3.59545	-1.98101	0.65933
O	3.33619	-2.88048	1.67215
O	4.05757	-0.70294	0.93268
C	4.38132	-2.66853	-0.57901
F	4.64199	-1.73613	-1.51985
F	5.54162	-3.1985	-0.13705
F	3.61531	-3.64395	-1.1122
I	1.57797	0.06966	0.00005

Sum of electronic and zero-point Energies=-1811.320328
Sum of electronic and thermal Energies= -1811.300335
Sum of electronic and thermal Enthalpies= -1811.299391
Sum of electronic and thermal Free Energies= -1811.372559

5-IIII-OTs

C	-2.93446	-1.10859	0.00015
C	-2.52243	0.21846	0.00015
C	-1.16116	0.50593	0.
C	-0.29395	-0.55644	-0.00016
C	-0.62935	-1.88835	-0.00026
C	-2.00137	-2.14559	-0.00009
H	-3.99148	-1.34315	0.0003
H	-3.22289	1.04544	0.00026
H	0.12474	-2.66268	-0.00066
H	-2.33707	-3.17535	-0.00015
C	-0.59322	1.89656	-0.00007
O	-1.26974	2.88566	0.00007
N	0.74447	1.89146	-0.00025
C	1.48431	3.16171	-0.00059
O	2.74271	3.1619	-0.00077
C	0.71675	4.4968	-0.00073
H	0.1032	4.55537	-0.87539
H	0.1002	4.55383	0.87191
H	1.41356	5.30881	0.00118
O	2.10706	-1.65082	0.00018
S	3.59545	-1.98101	0.65933
O	3.33619	-2.88048	1.67215
O	4.05757	-0.70294	0.93268
C	4.39592	-2.6813	-0.60202
C	4.28664	-4.05865	-0.84619
C	5.20842	-1.91947	-1.45574
C	4.96058	-4.65559	-1.90495
H	3.6583	-4.67401	-0.17663
C	5.88653	-2.50822	-2.51555
H	5.31325	-0.83488	-1.27023
C	5.76625	-3.88445	-2.75313
H	4.86253	-5.73806	-2.07844
H	6.52159	-1.89344	-3.1714
C	6.47114	-4.51366	-3.89243
H	7.39648	-3.94511	-4.15734
H	6.75712	-5.56782	-3.65365
H	5.7994	-4.52751	-4.78867
I	1.57797	0.06966	0.00005

Sum of electronic and zero-point Energies=-1744.495204
Sum of electronic and thermal Energies= -1744.472282
Sum of electronic and thermal Enthalpies= -1744.471338
Sum of electronic and thermal Free Energies= -1744.552417

5-IIII-radical

C	-2.93446	-1.10859	0.00015
C	-2.52243	0.21846	0.00015
C	-1.16116	0.50593	0.
C	-0.29395	-0.55644	-0.00016
C	-0.62935	-1.88835	-0.00026
C	-2.00137	-2.14559	-0.00009
H	-3.99148	-1.34315	0.0003
H	-3.22289	1.04544	0.00026
H	0.12474	-2.66268	-0.00066
H	-2.33707	-3.17535	-0.00015
C	-0.59322	1.89656	-0.00007
O	-1.26974	2.88566	0.00007
N	0.74447	1.89146	-0.00025
C	1.48431	3.16171	-0.00059
O	2.74271	3.1619	-0.00077
C	0.71675	4.4968	-0.00073
H	0.1032	4.55537	-0.87539
H	0.1002	4.55383	0.87191
H	1.41356	5.30881	0.00118
I	1.69761	0.10968	0.00006

Sum of electronic and zero-point Energies= -849.800435
Sum of electronic and thermal Energies= -849.788504
Sum of electronic and thermal Enthalpies=-849.787560
Sum of electronic and thermal Free Energies= -849.841093

5-IIII-SCF3

C	2.78872	2.53347	-0.0787
C	3.18535	1.22372	-0.31382
C	2.27986	0.18067	-0.13941
C	1.0132	0.52339	0.25857
C	0.55417	1.79211	0.51366
C	1.48729	2.81374	0.3311
H	3.49368	3.34408	-0.21405
H	4.18916	0.96587	-0.63234
H	-0.45947	1.99472	0.82838
H	1.17977	3.83591	0.514
C	2.65512	-1.26809	-0.37758
O	3.76604	-1.5693	-0.73478
F	-3.93551	0.92128	-0.45801
F	-2.96359	-0.85487	-1.19914
F	-1.93008	1.02411	-1.25123
C	-2.77223	0.27822	-0.52401
S	-2.16147	-0.00054	1.15757
N	1.63581	-2.10231	-0.14407
C	1.81673	-3.5507	-0.31822
O	0.82469	-4.32341	-0.2697
C	3.22512	-4.12441	-0.56086
H	3.58501	-3.79549	-1.51332
H	3.88663	-3.78293	0.20771
H	3.18228	-5.19345	-0.54555
I	-0.18758	-1.1862	0.47172

Sum of electronic and zero-point Energies=-1585.666387
Sum of electronic and thermal Energies= -1585.648906
Sum of electronic and thermal Enthalpies= -1585.647962
Sum of electronic and thermal Free Energies= -1585.714588

R-Br-radical

Br	2.14787	-1.78352	0.00019

Sum of electronic and zero-point Energies=-2574.150304
Sum of electronic and thermal Energies= -2574.148888
Sum of electronic and thermal Enthalpies= -2574.147944
Sum of electronic and thermal Free Energies= -2574.167134

R-CCH-radical

C	3.62908	1.1593	-0.00079
H	4.50254	1.76805	-0.0011
C	2.64213	0.47909	-0.00033

Sum of electronic and zero-point Energies=-76.593849
Sum of electronic and thermal Energies= -76.590850
Sum of electronic and thermal Enthalpies= -76.589905
Sum of electronic and thermal Free Energies=-76.608660

R-CF3-radical

C	2.40402	0.26982	0.00001
F	3.4394	-0.54682	0.00069
F	2.44547	1.03228	1.07774
F	2.44614	1.03133	-1.07837

Sum of electronic and zero-point Energies= -337.587347
Sum of electronic and thermal Energies= -337.583942
Sum of electronic and thermal Enthalpies=-337.582998
Sum of electronic and thermal Free Energies= -337.614016

R-CH3-radical

C	2.40402	0.26982	0.00001
H	2.4376	0.8875	0.87308
H	3.24415	-0.39281	0.00056
H	2.43814	0.88672	-0.87359

Sum of electronic and zero-point Energies=-39.795372
Sum of electronic and thermal Energies= -39.792235
Sum of electronic and thermal Enthalpies= -39.791291
Sum of electronic and thermal Free Energies=-39.815203

R-CHCH2-radical

C	3.72464	0.25538	-0.00009
H	3.80641	0.87362	-0.89051
C	4.7421	-0.87383	-0.00011
H	4.63795	-1.50612	-0.88255
H	5.75531	-0.47167	-0.00043

Sum of electronic and zero-point Energies=-77.852477
Sum of electronic and thermal Energies= -77.849400
Sum of electronic and thermal Enthalpies= -77.848455
Sum of electronic and thermal Free Energies=-77.874945

R-Cl-radical

Cl	2.14787	-1.78352	0.00019

Sum of electronic and zero-point Energies= -460.132636
Sum of electronic and thermal Energies= -460.131220
Sum of electronic and thermal Enthalpies=-460.130276
Sum of electronic and thermal Free Energies= -460.148313

R-CN-radical

C	-2.68934	0.59238	0.00006
N	-3.53721	1.37008	0.00019

Sum of electronic and zero-point Energies=-92.706569
Sum of electronic and thermal Energies= -92.704208
Sum of electronic and thermal Enthalpies= -92.703264
Sum of electronic and thermal Free Energies=-92.726224

R-F-radical

F	2.14787	-1.78352	0.00019

Sum of electronic and zero-point Energies=-99.733728
Sum of electronic and thermal Energies= -99.732311
Sum of electronic and thermal Enthalpies= -99.731367
Sum of electronic and thermal Free Energies=-99.748541

R-N3-radical

N	2.11882	-1.68906	0.00018
N	2.65816	-3.9473	0.79181
N	2.41713	-2.93233	0.46901

Sum of electronic and zero-point Energies= -164.124867
Sum of electronic and thermal Energies= -164.121851
Sum of electronic and thermal Enthalpies=-164.120907
Sum of electronic and thermal Free Energies= -164.140999

R-NH2-radcal

N	2.85796	1.20983	0.50755
H	2.57445	1.71184	1.3418
H	3.02922	1.88108	-0.23213

Sum of electronic and zero-point Energies=-55.851743
Sum of electronic and thermal Energies= -55.848906
Sum of electronic and thermal Enthalpies= -55.847962
Sum of electronic and thermal Free Energies=-55.870704

R-NHAc-radical

C	3.03067	0.32356	0.70615
O	2.2986	0.54165	1.65636
C	4.5177	0.61831	0.77378
H	5.03964	-0.29114	1.07395
H	4.91756	0.94099	-0.18626
H	4.692	1.38146	1.52658
N	2.5616	-0.20209	-0.45249
H	3.23016	-0.39625	-1.18392

Sum of electronic and zero-point Energies= -208.463344
Sum of electronic and thermal Energies= -208.458446
Sum of electronic and thermal Enthalpies=-208.457501
Sum of electronic and thermal Free Energies= -208.491123

R-OCF3-radical

O	-2.35941	-1.6203	-0.02187
C	-2.85321	-1.85258	1.29992
F	-3.8961	-2.70818	1.2468
F	-3.25722	-0.68371	1.84123
F	-1.87248	-2.38512	2.05956

Sum of electronic and zero-point Energies= -412.809276
Sum of electronic and thermal Energies= -412.805135
Sum of electronic and thermal Enthalpies=-412.804191
Sum of electronic and thermal Free Energies= -412.837231

R-OCH3-radical

O	-2.35941	-1.6203	-0.02187
C	-2.85321	-1.85258	1.29992
H	-2.07589	-2.27467	1.90201
H	-3.17343	-0.92614	1.72896
H	-3.6798	-2.53072	1.25781

Sum of electronic and zero-point Energies= -115.006791
Sum of electronic and thermal Energies= -115.003893
Sum of electronic and thermal Enthalpies=-115.002949
Sum of electronic and thermal Free Energies= -115.029686

R-OCOCF3-radical

C	-2.41859	-0.53165	0.0117
O	-2.5234	-1.72768	0.04512
O	-1.32746	0.16971	-0.02174
C	-3.65945	0.39701	0.00003
F	-4.78188	-0.30037	0.05516
F	-3.67375	1.13597	-1.11316
F	-3.62623	1.23022	1.0443

Sum of electronic and zero-point Energies= -526.130937
Sum of electronic and thermal Energies= -526.125650
Sum of electronic and thermal Enthalpies=-526.124705
Sum of electronic and thermal Free Energies= -526.161064

R-OCOCH3-radical

C	-0.16211	-0.0627	-0.00118
O	-0.91789	-0.99221	0.00029
O	-0.60642	1.18531	0.00014
C	1.34365	-0.11785	-0.00041
H	1.79202	0.87143	-0.05508
H	1.65791	-0.61613	0.91469
H	1.65535	-0.71682	-0.85349

Sum of electronic and zero-point Energies= -228.352848
Sum of electronic and thermal Energies= -228.348159
Sum of electronic and thermal Enthalpies=-228.347215
Sum of electronic and thermal Free Energies= -228.381460

R-OCOPh-radical

C	2.26994	0.14906	-0.38897
O	1.77727	-0.12125	-1.47464
O	1.58234	0.40817	0.67958
C	3.76622	0.18143	-0.21379
C	4.34122	0.48998	1.01406
C	4.57789	-0.10576	-1.30615
C	5.72206	0.51171	1.14808
H	3.69517	0.70998	1.85223
C	5.95737	-0.08354	-1.17179
H	4.1052	-0.34217	-2.24957
C	6.53043	0.22526	0.05588
H	6.16881	0.75215	2.10365
H	6.58769	-0.30605	-2.02248
H	7.60731	0.24267	0.161

Sum of electronic and zero-point Energies= -420.029231
Sum of electronic and thermal Energies= -420.022043
Sum of electronic and thermal Enthalpies=-420.021099
Sum of electronic and thermal Free Energies= -420.062359

R-OH-radical

O	-2.35941	-1.6203	-0.02187
H	-2.69235	-1.77691	0.86934

Sum of electronic and zero-point Energies=-75.724871
Sum of electronic and thermal Energies= -75.722510
Sum of electronic and thermal Enthalpies= -75.721566
Sum of electronic and thermal Free Energies=-75.741789

R-OTf-radical

O	2.10706	-1.65082	0.00018
S	3.59721	-1.98047	0.65562
O	3.34066	-2.87911	1.66987
O	4.06006	-0.70218	0.92668
C	4.37976	-2.66901	-0.58425
F	4.64796	-1.73511	-1.52148
F	5.53586	-3.20853	-0.14284
F	3.60767	-3.63722	-1.12177

Sum of electronic and zero-point Energies= -961.408573
Sum of electronic and thermal Energies= -961.401544
Sum of electronic and thermal Enthalpies=-961.400600
Sum of electronic and thermal Free Energies= -961.441623

R-OTs-radical

O	2.10706	-1.65082	0.00018
S	3.59721	-1.98047	0.65562
O	3.34066	-2.87911	1.66987
O	4.06006	-0.70218	0.92668
C	4.39431	-2.6818	-0.60729
C	4.28437	-4.05935	-0.85004
C	5.20451	-1.92067	-1.4638
C	4.95548	-4.65716	-1.9101
H	3.65783	-4.67415	-0.17829
C	5.87979	-2.51029	-2.52494
H	5.30985	-0.83593	-1.27946
C	5.75888	-3.88672	-2.76107
H	4.85696	-5.73977	-2.08244
H	6.5131	-1.89605	-3.18299
C	6.46072	-4.51686	-3.90173
H	7.38535	-3.94853	-4.16957
H	6.74734	-5.57083	-3.66285
H	5.78659	-4.53145	-4.79616

Sum of electronic and zero-point Energies= -894.589227
Sum of electronic and thermal Energies= -894.579907
Sum of electronic and thermal Enthalpies=-894.578963
Sum of electronic and thermal Free Energies= -894.625146

R-SCF3-radical

F	-3.93551	0.92128	-0.45801
F	-2.96359	-0.85487	-1.19914
F	-1.93008	1.02411	-1.25123
C	-2.77223	0.27822	-0.52401
S	-2.16147	-0.00054	1.15757

Sum of electronic and zero-point Energies= -735.794953
Sum of electronic and thermal Energies= -735.790928
Sum of electronic and thermal Enthalpies=-735.789984
Sum of electronic and thermal Free Energies= -735.823614

1-BrIII-CF2SO2Ph

C	-2.50292	2.3336	0.00008
C	-2.56795	0.95015	0.00013
C	-1.40848	0.17603	-0.00005
C	-0.20393	0.8445	-0.00019
C	-0.10246	2.22552	-0.00023
C	-1.27038	2.97377	-0.00012
H	-3.41425	2.91608	0.0002
H	-3.53199	0.45746	0.00031
H	0.85447	2.72311	-0.00037
H	-1.21335	4.05357	-0.0002
O	-0.13735	-1.83753	-0.0011
C	2.85571	1.27624	0.0006
C	-1.43324	-1.3577	0.00003
C	-2.16189	-1.84555	-1.25846
C	-2.15981	-1.84533	1.25981
H	-2.15135	-2.9352	-1.26312
H	-3.197	-1.50258	-1.29655
H	-1.63917	-1.49087	-2.14673
H	-2.14948	-2.93498	1.26457
H	-3.19478	-1.50211	1.29966
H	-1.6355	-1.49065	2.14715
S	4.46476	0.51509	0.00122
C	5.71873	1.7784	0.00185
C	6.211	2.27317	-1.20618
C	6.20914	2.2738	1.21
C	7.19395	3.26263	-1.20597
H	5.82471	1.88185	-2.15849
C	7.19169	3.26426	1.21038
H	5.82114	1.88408	2.16221
C	7.68423	3.75862	0.00268
H	7.58247	3.65221	-2.15812
H	7.57787	3.65496	2.16311
H	8.45921	4.53881	0.00259
O	4.63075	-0.43469	1.36478
O	4.6319	-0.43449	-1.36232
F	2.70976	2.02418	1.11495
F	2.71076	2.02445	-1.11371
Br	1.48653	-0.42543	-0.00015

Sum of electronic and zero-point Energies=-4016.028911
Sum of electronic and thermal Energies= -4016.007079
Sum of electronic and thermal Enthalpies= -4016.006135
Sum of electronic and thermal Free Energies= -4016.081491

1-ClIII-CF2SO2Ph

C	-2.50292	2.3336	0.00008
C	-2.56795	0.95015	0.00013
C	-1.40848	0.17603	-0.00005
C	-0.20393	0.8445	-0.00019
C	-0.10246	2.22552	-0.00023
C	-1.27038	2.97377	-0.00012
H	-3.41425	2.91608	0.0002
H	-3.53199	0.45746	0.00031
H	0.85447	2.72311	-0.00037
H	-1.21335	4.05357	-0.0002
O	-0.13735	-1.83753	-0.0011
C	2.85571	1.27624	0.0006
C	-1.43324	-1.3577	0.00003
C	-2.16189	-1.84555	-1.25846
C	-2.15981	-1.84533	1.25981
H	-2.15135	-2.9352	-1.26312
H	-3.197	-1.50258	-1.29655
H	-1.63917	-1.49087	-2.14673
H	-2.14948	-2.93498	1.26457
H	-3.19478	-1.50211	1.29966
H	-1.6355	-1.49065	2.14715
S	4.46476	0.51509	0.00122
C	5.71873	1.7784	0.00185
C	6.211	2.27317	-1.20618
C	6.20914	2.2738	1.21
C	7.19395	3.26263	-1.20597
H	5.82471	1.88185	-2.15849
C	7.19169	3.26426	1.21038
H	5.82114	1.88408	2.16221
C	7.68423	3.75862	0.00268
H	7.58247	3.65221	-2.15812
H	7.57787	3.65496	2.16311
H	8.45921	4.53881	0.00259
O	4.63075	-0.43469	1.36478
O	4.6319	-0.43449	-1.36232
F	2.70976	2.02418	1.11495
F	2.71076	2.02445	-1.11371
Cl	1.48653	-0.42543	-0.00015

Sum of electronic and zero-point Energies=-1902.009023
Sum of electronic and thermal Energies= -1901.987611
Sum of electronic and thermal Enthalpies= -1901.986667
Sum of electronic and thermal Free Energies= -1902.060470

1-IIII-CF2SO2Ph

C	-2.50292	2.3336	0.00008
C	-2.56795	0.95015	0.00013
C	-1.40848	0.17603	-0.00005
C	-0.20393	0.8445	-0.00019
C	-0.10246	2.22552	-0.00023
C	-1.27038	2.97377	-0.00012
H	-3.41425	2.91608	0.0002
H	-3.53199	0.45746	0.00031
H	0.85447	2.72311	-0.00037
H	-1.21335	4.05357	-0.0002
O	-0.13735	-1.83753	-0.0011
C	2.85571	1.27624	0.0006
C	-1.43324	-1.3577	0.00003
C	-2.16189	-1.84555	-1.25846
C	-2.15981	-1.84533	1.25981
H	-2.15135	-2.9352	-1.26312
H	-3.197	-1.50258	-1.29655
H	-1.63917	-1.49087	-2.14673
H	-2.14948	-2.93498	1.26457
H	-3.19478	-1.50211	1.29966
H	-1.6355	-1.49065	2.14715
I	1.48653	-0.42543	-0.00015
S	4.46476	0.51509	0.00122
C	5.71873	1.7784	0.00185
C	6.211	2.27317	-1.20618
C	6.20914	2.2738	1.21
C	7.19395	3.26263	-1.20597
H	5.82471	1.88185	-2.15849
C	7.19169	3.26426	1.21038
H	5.82114	1.88408	2.16221
C	7.68423	3.75862	0.00268
H	7.58247	3.65221	-2.15812
H	7.57787	3.65496	2.16311
H	8.45921	4.53881	0.00259
O	4.63075	-0.43469	1.36478
O	4.6319	-0.43449	-1.36232
F	2.70976	2.02418	1.11495
F	2.71076	2.02445	-1.11371

Sum of electronic and zero-point Energies=-1739.500310
Sum of electronic and thermal Energies= -1739.478353
Sum of electronic and thermal Enthalpies= -1739.477409
Sum of electronic and thermal Free Energies= -1739.553112

6-BrIII-F

C	-2.67415	2.10311	0.00008
C	-2.61537	0.71778	0.00012
C	-1.38589	0.06496	-0.00013
C	-0.25552	0.85364	-0.00033
C	-0.26904	2.23578	-0.00033
C	-1.50832	2.86008	-0.00017
H	-3.63604	2.59736	0.00025
H	-3.5294	0.1374	0.00035
H	0.65594	2.79207	-0.00044
H	-1.56054	3.93999	-0.00022
F	2.47779	1.45033	0.00118
C	-1.23354	-1.45184	0.0001
C	-1.86863	-2.04079	-1.25852
C	-1.86486	-2.03983	1.2611
H	-1.39732	-1.61922	-2.14583
H	-2.93845	-1.83348	-1.29661
H	-2.93447	-1.83205	1.30248
H	-1.71845	-3.11976	-1.26124
H	-1.71505	-3.11886	1.26402
H	-1.39059	-1.61787	2.14665
S	0.14024	-1.7845	-0.00205
Br	1.48669	-0.28151	-0.00028

Sum of electronic and zero-point Energies=-3420.930393
Sum of electronic and thermal Energies= -3420.918518
Sum of electronic and thermal Enthalpies= -3420.917573
Sum of electronic and thermal Free Energies= -3420.968207

6-BrIII-radical

C	-2.67415	2.10311	0.00008
C	-2.61537	0.71778	0.00012
C	-1.38589	0.06496	-0.00013
C	-0.25552	0.85364	-0.00033
C	-0.26904	2.23578	-0.00033
C	-1.50832	2.86008	-0.00017
H	-3.63604	2.59736	0.00025
H	-3.5294	0.1374	0.00035
H	0.65594	2.79207	-0.00044
H	-1.56054	3.93999	-0.00022
C	-1.23354	-1.45184	0.0001
C	-1.86863	-2.04079	-1.25852
C	-1.86486	-2.03983	1.2611
H	-1.39732	-1.61922	-2.14583
H	-2.93845	-1.83348	-1.29661
H	-2.93447	-1.83205	1.30248
H	-1.71845	-3.11976	-1.26124
H	-1.71505	-3.11886	1.26402
H	-1.39059	-1.61787	2.14665
S	0.14024	-1.7845	-0.00205

Br	1.48669	-0.28151	-0.00028

Sum of electronic and zero-point Energies=-3321.127618
Sum of electronic and thermal Energies= -3321.116861
Sum of electronic and thermal Enthalpies= -3321.115916
Sum of electronic and thermal Free Energies= -3321.165250

6-ClIII-F

C	-2.67415	2.10311	0.00008
C	-2.61537	0.71778	0.00012
C	-1.38589	0.06496	-0.00013
C	-0.25552	0.85364	-0.00033
C	-0.26904	2.23578	-0.00033
C	-1.50832	2.86008	-0.00017
H	-3.63604	2.59736	0.00025
H	-3.5294	0.1374	0.00035
H	0.65594	2.79207	-0.00044
H	-1.56054	3.93999	-0.00022
F	2.47779	1.45033	0.00118
C	-1.23354	-1.45184	0.0001
C	-1.86863	-2.04079	-1.25852
C	-1.86486	-2.03983	1.2611
H	-1.39732	-1.61922	-2.14583
H	-2.93845	-1.83348	-1.29661
H	-2.93447	-1.83205	1.30248
H	-1.71845	-3.11976	-1.26124
H	-1.71505	-3.11886	1.26402
H	-1.39059	-1.61787	2.14665
S	0.14024	-1.7845	-0.00205
Cl	1.48669	-0.28151	-0.00028

Sum of electronic and zero-point Energies=-1306.908217
Sum of electronic and thermal Energies= -1306.896606
Sum of electronic and thermal Enthalpies= -1306.895662
Sum of electronic and thermal Free Energies= -1306.945326

6-ClIII-radical

C	2.42758	2.00975	-0.14499
C	1.04541	2.05581	-0.04056
C	0.26274	0.90305	0.04321
C	0.95626	-0.31126	0.01509
C	2.33664	-0.37686	-0.08591
C	3.07975	0.78985	-0.16611
H	2.98875	2.9317	-0.21112
H	0.56606	3.02275	-0.02695
H	2.82216	-1.34175	-0.10772
H	4.1564	0.73722	-0.24904
C	-1.25091	0.99847	0.21306
C	-1.78525	2.41276	-0.0589
C	-1.64846	0.60772	1.64179
H	-1.48646	2.7774	-1.04055
H	-1.42394	3.1024	0.70571
H	-1.15949	1.28082	2.34944
H	-2.87324	2.40351	-0.00598
H	-2.72809	0.69523	1.75776
H	-1.35885	-0.41213	1.88011
S	-2.11681	-0.01114	-1.03446
Cl	0.1212	-1.85917	0.0803

Sum of electronic and zero-point Energies=-1207.133998
Sum of electronic and thermal Energies= -1207.123512
Sum of electronic and thermal Enthalpies= -1207.122568
Sum of electronic and thermal Free Energies= -1207.170620

6-IIII-F

C	-2.67415	2.10311	0.00008
C	-2.61537	0.71778	0.00012
C	-1.38589	0.06496	-0.00013
C	-0.25552	0.85364	-0.00033
C	-0.26904	2.23578	-0.00033
C	-1.50832	2.86008	-0.00017
H	-3.63604	2.59736	0.00025
H	-3.5294	0.1374	0.00035
H	0.65594	2.79207	-0.00044
H	-1.56054	3.93999	-0.00022
F	2.47779	1.45033	0.00118
C	-1.23354	-1.45184	0.0001
C	-1.86863	-2.04079	-1.25852
C	-1.86486	-2.03983	1.2611
H	-1.39732	-1.61922	-2.14583
H	-2.93845	-1.83348	-1.29661
H	-2.93447	-1.83205	1.30248
H	-1.71845	-3.11976	-1.26124
H	-1.71505	-3.11886	1.26402
H	-1.39059	-1.61787	2.14665
I	1.48669	-0.28151	-0.00028
S	0.14024	-1.7845	-0.00205

Sum of electronic and zero-point Energies=-1144.404042
Sum of electronic and thermal Energies= -1144.392015
Sum of electronic and thermal Enthalpies= -1144.391070
Sum of electronic and thermal Free Energies= -1144.442538

6-IIII-radical

C	-2.67415	2.10311	0.00008
C	-2.61537	0.71778	0.00012
C	-1.38589	0.06496	-0.00013
C	-0.25552	0.85364	-0.00033
C	-0.26904	2.23578	-0.00033
C	-1.50832	2.86008	-0.00017
H	-3.63604	2.59736	0.00025
H	-3.5294	0.1374	0.00035
H	0.65594	2.79207	-0.00044
H	-1.56054	3.93999	-0.00022
C	-1.23354	-1.45184	0.0001
C	-1.86863	-2.04079	-1.25852
C	-1.86486	-2.03983	1.2611
H	-1.39732	-1.61922	-2.14583
H	-2.93845	-1.83348	-1.29661
H	-2.93447	-1.83205	1.30248
H	-1.71845	-3.11976	-1.26124
H	-1.71505	-3.11886	1.26402
H	-1.39059	-1.61787	2.14665
S	0.14024	-1.7845	-0.00205
I	1.48669	-0.28151	-0.00028

Sum of electronic and zero-point Energies=-1044.562720
Sum of electronic and thermal Energies= -1044.551767
Sum of electronic and thermal Enthalpies= -1044.550823
Sum of electronic and thermal Free Energies= -1044.601221

R-CF2SO2Ph-radical

C	2.85571	1.27624	0.0006
S	4.46476	0.51509	0.00122
C	5.71873	1.7784	0.00185
C	6.211	2.27317	-1.20618
C	6.20914	2.2738	1.21
C	7.19395	3.26263	-1.20597
H	5.82471	1.88185	-2.15849
C	7.19169	3.26426	1.21038
H	5.82114	1.88408	2.16221
C	7.68423	3.75862	0.00268
H	7.58247	3.65221	-2.15812
H	7.57787	3.65496	2.16311
H	8.45921	4.53881	0.00259
O	4.63075	-0.43469	1.36478
O	4.6319	-0.43449	-1.36232
F	2.70976	2.02418	1.11495
F	2.71076	2.02445	-1.11371

Sum of electronic and zero-point Energies=-1017.877745
Sum of electronic and thermal Energies= -1017.867308
Sum of electronic and thermal Enthalpies= -1017.866363
Sum of electronic and thermal Free Energies= -1017.915950

1-BrIII-anion (in Acetonitrile)

C	1.37854	-2.69048	0.04194
C	1.7593	-1.35536	0.0273
C	0.8467	-0.2943	-0.00751
C	-0.49836	-0.67495	-0.01075
C	-0.90502	-2.00229	0.01355
C	0.03408	-3.02283	0.03371
H	2.1346	-3.46654	0.05856
H	2.81513	-1.1251	0.03282
H	-1.96266	-2.22841	0.01475
H	-0.28789	-4.05647	0.04284
O	0.62258	1.85159	-1.043
C	1.29997	1.20738	-0.10277
C	2.82961	1.26446	-0.38809
C	1.10673	1.80882	1.32683
H	3.04198	0.76886	-1.33772
H	3.47246	0.84643	0.39539
H	1.68123	1.28478	2.09935
H	3.06378	2.32314	-0.50149
H	1.43088	2.85132	1.28178
H	0.05137	1.79565	1.5976
Br	-1.92945	0.59288	-0.027

Sum of electronic and zero-point Energies= -2998.299041
Sum of electronic and thermal Energies= -2998.288621
Sum of electronic and thermal Enthalpies= -2998.287677
Sum of electronic and thermal Free Energies= -2998.335354

1-BrIII-Br (in Acetonitrile)

C	-2.04035	2.84698	0.05489
C	-2.5659	1.56311	0.11017
C	-1.72726	0.46123	0.01266
C	-0.37785	0.7091	-0.1241
C	0.19157	1.95846	-0.17863
C	-0.67465	3.04314	-0.09188
H	-2.70165	3.69963	0.12404
H	-3.63161	1.41448	0.22532
H	1.25822	2.08618	-0.28069
H	-0.26946	4.04403	-0.14095
Br	0.61073	-0.9639	-0.22502
O	-1.11851	-1.7315	-0.54841
C	-2.14837	-0.99464	0.08134
C	-3.42086	-1.26546	-0.71001
C	-2.3016	-1.41942	1.54222
H	-3.3038	-0.92901	-1.73841
H	-4.27435	-0.75844	-0.25949
H	-3.08641	-0.84221	2.03169
H	-3.61736	-2.33653	-0.70891

H	-2.55741	-2.47759	1.5843
H	-1.37045	-1.26233	2.08775
Br	2.90053	0.10101	0.14738

Sum of electronic and zero-point Energies= -5572.334654
Sum of electronic and thermal Energies= -5572.322529
Sum of electronic and thermal Enthalpies= -5572.321585
Sum of electronic and thermal Free Energies= -5572.374065

1-BrIII-CCH (in Acetonitrile)

C	-1.47784	2.86145	0.03034
C	-2.0225	1.58679	0.05887
C	-1.20342	0.46278	-0.00179
C	0.14941	0.69073	-0.073
C	0.74099	1.93408	-0.09943
C	-0.10271	3.03613	-0.05279
H	-2.12833	3.72443	0.0711
H	-3.09468	1.45695	0.12705
H	1.81297	2.04725	-0.15525
H	0.32231	4.02966	-0.08167
Br	1.18041	-0.97072	-0.11678
O	-0.63703	-1.78431	-0.38264
C	-1.69058	-0.9845	0.03468
C	-2.86618	-1.18298	-0.92188
C	-2.10141	-1.32814	1.47242
H	-2.57499	-0.89886	-1.93205
H	-3.74087	-0.60407	-0.62299
H	-2.91611	-0.69238	1.82282
H	-3.13356	-2.23898	-0.92145
H	-2.42141	-2.36932	1.50635
H	-1.25084	-1.20578	2.14422
C	4.01111	0.34469	0.24723
H	5.01072	0.68796	0.37251
C	2.87922	-0.03555	0.10832

Sum of electronic and zero-point Energies= -3074.809553
Sum of electronic and thermal Energies= -3074.796568
Sum of electronic and thermal Enthalpies= -3074.795624
Sum of electronic and thermal Free Energies= -3074.849174

1-BrIII-CF3 (in Acetonitrile)

C	2.04483	2.79571	-0.06717
C	2.53414	1.49979	-0.1151
C	1.67298	0.41204	-0.01055
C	0.33208	0.68858	0.11799
C	-0.20154	1.95999	0.17675
C	0.68472	3.02513	0.08568
H	2.72702	3.63144	-0.14132
H	3.59587	1.32528	-0.22832
H	-1.25461	2.1478	0.30036
H	0.30146	4.03442	0.13856
Br	-0.73954	-0.94672	0.20357
O	1.06094	-1.79528	0.49966
C	-2.53265	0.02962	-0.10603
F	-3.40966	-0.96422	-0.24962
F	-2.57278	0.77543	-1.20905
F	-2.94495	0.78479	0.91573
C	2.10274	-1.05445	-0.04109
C	3.34801	-1.27403	0.81636
C	2.3705	-1.4602	-1.49627
H	3.56435	-2.34131	0.83732
H	4.21853	-0.75335	0.41594
H	3.16322	-0.93672	1.83512
H	2.64788	-2.5137	-1.52166
H	3.17375	-0.86817	-1.93748
H	1.47041	-1.32548	-2.09769

Sum of electronic and zero-point Energies= -3335.756527
Sum of electronic and thermal Energies= -3335.742070
Sum of electronic and thermal Enthalpies= -3335.741126
Sum of electronic and thermal Free Energies= -3335.798250

1-BrIII-CH3 (in Acetonitrile)

C	-1.73404	2.64387	-0.03259
C	-2.07551	1.301	-0.04431
C	-1.09998	0.30786	0.00764
C	0.20983	0.72882	0.05042
C	0.59499	2.05593	0.06391
C	-0.40049	3.0226	0.02748
H	-2.50861	3.39786	-0.06708
H	-3.11693	1.01069	-0.09217
H	1.6287	2.35538	0.10923
H	-0.12585	4.06805	0.04692
Br	1.51295	-0.72246	0.06784
O	-0.24847	-1.8584	0.34966
C	3.11223	0.46198	-0.17483
C	-1.39782	-1.19697	-0.00015
C	-1.85525	-1.5847	-1.41661
C	-2.50267	-1.51488	1.01458
H	-2.05133	-2.65675	-1.43408
H	-2.75808	-1.05157	-1.72136
H	-1.06163	-1.36899	-2.13331
H	-2.64405	-2.59508	1.02853
H	-3.45479	-1.04359	0.76529
H	-2.19241	-1.19506	2.00869
H	3.27458	1.08039	0.70223
H	3.92108	-0.25676	-0.2793
H	3.01617	1.05571	-1.07838

Sum of electronic and zero-point Energies= -3037.969393
Sum of electronic and thermal Energies= -3037.957133
Sum of electronic and thermal Enthalpies= -3037.956189
Sum of electronic and thermal Free Energies= -3038.007597

1-BrIII-CHCH2 (in Acetonitrile)

C	-1.79294	2.73678	0.01164
C	-2.21849	1.41681	0.02332
C	-1.30259	0.36849	-0.00841
C	0.02769	0.71559	-0.02256
C	0.49918	2.012	-0.04918
C	-0.43836	3.0364	-0.03567
H	-2.52087	3.53656	0.02743
H	-3.2767	1.19223	0.04863
H	1.55468	2.23201	-0.10113
H	-0.10403	4.06399	-0.06831
Br	1.23941	-0.81424	-0.00102
O	-0.55448	-1.81343	-0.4594
C	-1.66421	-1.1231	-0.03398
C	-2.81939	-1.36666	-1.00995
C	-2.07581	-1.54249	1.38715
H	-2.54249	-1.01804	-2.00412
H	-3.74311	-0.87578	-0.6994
H	-2.94117	-0.98246	1.74697
H	-2.99769	-2.44031	-1.05773
H	-2.31653	-2.60552	1.37869
H	-1.24607	-1.38423	2.07772
C	2.82776	0.26686	0.43902
H	2.79896	0.80105	1.38069
C	3.90148	0.18023	-0.32236
H	4.83421	0.65249	-0.03756
H	3.89134	-0.37175	-1.25505

Sum of electronic and zero-point Energies= -3076.039700
Sum of electronic and thermal Energies= -3076.026467
Sum of electronic and thermal Enthalpies= -3076.025523
Sum of electronic and thermal Free Energies= -3076.079577

1-BrIII-Cl (in Acetonitrile)

C	-1.6596	2.8079	0.00019
C	-2.15338	1.51299	0.00034
C	-1.28363	0.42774	-0.00002
C	0.06467	0.70566	-0.00046
C	0.60367	1.97326	-0.00062
C	-0.28979	3.03625	-0.00031
H	-2.34549	3.64374	0.00044
H	-3.22142	1.33609	0.00074
H	1.67134	2.12509	-0.00094
H	0.09524	4.04626	-0.00045
Br	1.11763	-0.9221	-0.00064
O	-0.56344	-1.82993	-0.00146
Cl	3.20662	0.21259	0.00095
C	-1.7243	-1.02059	0.00022
C	-2.52931	-1.33364	-1.25977
C	-2.52624	-1.334	1.26208
H	-3.44378	-0.74579	1.29941
H	-1.93098	-1.11607	2.14786
H	-2.78666	-2.39171	1.26548
H	-2.78938	-2.39143	-1.26313
H	-3.44716	-0.74576	-1.2945
H	-1.93628	-1.11499	-2.14686

Sum of electronic and zero-point Energies= -3458.333406
Sum of electronic and thermal Energies= -3458.321496
Sum of electronic and thermal Enthalpies= -3458.320552
Sum of electronic and thermal Free Energies= -3458.371741

1-BrIII-CN (in Acetonitrile)

C	-1.41061	2.88777	0.02897
C	-1.98064	1.62369	0.06229
C	-1.17946	0.489	-0.00264
C	0.17712	0.69369	-0.08445
C	0.79474	1.92321	-0.1157
C	-0.03346	3.03782	-0.06301
H	-2.04424	3.76282	0.07313
H	-3.05437	1.51298	0.1374
H	1.86783	2.02556	-0.17804
H	0.40895	4.02342	-0.09497
Br	1.13378	-1.00927	-0.13743
O	-0.63471	-1.75043	-0.44989
C	2.90538	-0.04369	0.14838
N	3.9539	0.40292	0.30505
C	-1.67327	-0.95119	0.04677
C	-2.88809	-1.15475	-0.85191
C	-1.99471	-1.32265	1.49719
H	-2.65463	-0.85307	-1.87156
H	-3.74819	-0.58711	-0.49615
H	-2.78924	-0.6931	1.89943
H	-1.11035	-1.20476	2.12473
H	-2.31116	-2.36451	1.53514
H	-3.14856	-2.21222	-0.85074

Sum of electronic and zero-point Energies= -3090.926886
Sum of electronic and thermal Energies= -3090.914017

Sum of electronic and thermal Enthalpies= -3090.913073
Sum of electronic and thermal Free Energies= -3090.966350

1-BrIII-F (in Acetonitrile)

C	-1.85024	2.54684	0.00017
C	-2.12729	1.18811	0.00022
C	-1.08848	0.26401	-0.0001
C	0.19355	0.75989	-0.00035
C	0.52128	2.09957	-0.00038
C	-0.53612	2.99949	-0.00016
H	-2.66396	3.25893	0.00038
H	-3.15245	0.8401	0.00052
H	1.55276	2.41254	-0.00053
H	-0.32688	4.06006	-0.00023
Br	1.4998	-0.64357	-0.00037
O	0.02915	-1.82514	-0.00211
F	2.858	0.67483	0.00107
C	-1.26126	-1.24167	0.00007
C	-1.99685	-1.69509	-1.25903
C	-1.99306	-1.69508	1.26141
H	-1.45293	-1.37336	-2.14628
H	-3.00547	-1.28181	-1.29381
H	-3.00147	-1.28154	1.29938
H	-2.06218	-2.78254	-1.26258
H	-2.0586	-2.78252	1.26498
H	-1.44638	-1.37363	2.14708

Sum of electronic and zero-point Energies= -3097.965925
Sum of electronic and thermal Energies= -3097.954516
Sum of electronic and thermal Enthalpies= -3097.953572
Sum of electronic and thermal Free Energies= -3098.003139

1-BrIII-N3 (in Acetonitrile)

C	1.44498	2.93563	0.02345
C	2.05317	1.70334	0.22219
C	1.32724	0.53219	0.04928
C	0.00382	0.66134	-0.30618
C	-0.64513	1.85693	-0.51337
C	0.10903	3.01287	-0.34581
H	2.01885	3.84278	0.15347
H	3.09552	1.65017	0.50818
H	-1.68346	1.88872	-0.80556
H	-0.35667	3.97461	-0.50869
Br	-0.84667	-1.07589	-0.47121
O	0.96547	-1.72308	-0.48083
C	1.8387	-0.88593	0.24401
C	1.81457	-1.23809	1.73301
C	3.23067	-1.0756	-0.34396
H	0.80599	-1.13559	2.13515
H	2.47711	-0.58226	2.29863
H	3.96884	-0.48049	0.19423
H	2.13834	-2.27036	1.86211
H	3.50819	-2.12543	-0.26116
H	3.23661	-0.79205	-1.39487
N	-3.39037	0.45607	1.60896
N	-3.06461	0.1184	0.58351
N	-2.73961	-0.22382	-0.53792

Sum of electronic and zero-point Energies= -3162.284162
Sum of electronic and thermal Energies= -3162.270738
Sum of electronic and thermal Enthalpies= -3162.269794
Sum of electronic and thermal Free Energies= -3162.324696

1-BrIII-NH2 (in Acetonitrile)

C	-1.90717	2.53521	0.02607
C	-2.16507	1.17223	0.03325
C	-1.12295	0.25249	-0.01525
C	0.15897	0.75392	-0.05477
C	0.45838	2.10002	-0.05532
C	-0.60017	2.99927	-0.02162
H	-2.72798	3.23865	0.0565
H	-3.18587	0.81482	0.07467
H	1.47624	2.45541	-0.08517
H	-0.39614	4.0609	-0.0332
Br	1.53357	-0.62774	-0.0718
O	-0.0696	-1.80411	-0.40049
C	-1.28099	-1.2644	0.00424
C	-2.36973	-1.7114	-0.97119
C	-1.62778	-1.70408	1.43339
N	3.04646	0.53613	0.1853
H	-1.7115	-2.79046	1.45549
H	-2.56745	-1.26623	1.77456
H	-0.83447	-1.40502	2.11972
H	-2.39567	-2.80041	-0.98131
H	-3.35567	-1.34423	-0.68271
H	-2.13515	-1.36056	-1.97518
H	2.95434	1.09092	1.02894
H	3.17481	1.15736	-0.60526

Sum of electronic and zero-point Energies= -3054.023848
Sum of electronic and thermal Energies= -3054.011749
Sum of electronic and thermal Enthalpies= -3054.010805
Sum of electronic and thermal Free Energies= -3054.062223

1-BrIII-NHAc (in Acetonitrile)

C	-1.71238	2.95307	0.00371
C	-2.33495	1.73091	0.21289
C	-1.63038	0.54629	0.03491
C	-0.31141	0.65196	-0.34084
C	0.35391	1.83825	-0.54348
C	-0.37737	3.00714	-0.37366
H	-2.27039	3.86952	0.13852
H	-3.37319	1.6938	0.51562
H	1.39518	1.85466	-0.8217
H	0.10591	3.96093	-0.53283
Br	0.51714	-1.09647	-0.5146
O	-1.32746	-1.72141	-0.45862
C	2.98866	0.06227	0.49711
O	2.39976	0.2611	1.54065
C	4.49265	0.21498	0.38247
H	4.95457	-0.76271	0.52497
H	4.79414	0.59737	-0.59151
H	4.8387	0.88002	1.16805
C	-2.16498	-0.86019	0.2633
C	-3.58019	-1.02482	-0.27734
C	-2.10789	-1.17474	1.76185
H	-3.61051	-0.76332	-1.33388
H	-4.29104	-0.4034	0.26811
H	-3.87748	-2.06675	-0.16576
H	-2.44096	-2.19989	1.92266
H	-2.74691	-0.49774	2.33022
H	-1.08654	-1.07481	2.13221
N	2.33643	-0.30786	-0.63868
H	2.88114	-0.54444	-1.45462

Sum of electronic and zero-point Energies= -3206.656569
Sum of electronic and thermal Energies= -3206.640968
Sum of electronic and thermal Enthalpies= -3206.640024
Sum of electronic and thermal Free Energies= -3206.700084

1-BrIII-OCOCH3 (in Acetonitrile)

C	-2.48749	2.65494	0.07595
C	-2.82287	1.309	0.13098
C	-1.83276	0.34168	0.02766
C	-0.53154	0.77435	-0.1168
C	-0.15068	2.09807	-0.17026
C	-1.16408	3.04518	-0.0759
H	-3.26389	3.404	0.1492
H	-3.85566	1.00869	0.25032
H	0.88405	2.37614	-0.27841
H	-0.90926	4.09471	-0.12394
Br	0.66853	-0.73177	-0.22621
O	-0.90393	-1.73597	-0.5306
C	3.33499	-0.0176	0.10895
O	3.45194	-1.21954	0.00462
O	2.18186	0.61126	0.05843
C	4.50791	0.91287	0.30858
H	4.56178	1.61088	-0.52588
H	4.36296	1.49441	1.21765
H	5.42474	0.33562	0.37207
C	-2.0357	-1.16154	0.09642
C	-3.25395	-1.61798	-0.69284
C	-2.12385	-1.60413	1.55773
H	-3.18826	-1.26973	-1.72199
H	-4.17329	-1.24228	-0.24339
H	-3.28946	-2.70645	-0.69055
H	-2.22146	-2.68838	1.60003
H	-2.98465	-1.14803	2.04745
H	-1.22512	-1.31276	2.10233

Sum of electronic and zero-point Energies= -3226.550838
Sum of electronic and thermal Energies= -3226.535339
Sum of electronic and thermal Enthalpies= -3226.534394
Sum of electronic and thermal Free Energies= -3226.594771

1-BrIII-OH (in Acetonitrile)

C	-2.02238	2.4381	0.02928
C	-2.21814	1.06361	0.04365
C	-1.12897	0.20273	-0.01004
C	0.12189	0.77418	-0.0602
C	0.36782	2.12923	-0.07234
C	-0.74011	2.96788	-0.032
H	-2.87626	3.10091	0.06251
H	-3.2202	0.65758	0.09298
H	1.37853	2.5031	-0.11907
H	-0.5945	4.03899	-0.05275
Br	1.52829	-0.5504	-0.10354
O	0.05748	-1.7648	-0.47437
O	2.8674	0.793	0.16648
H	3.01756	0.85367	1.11479
C	-1.17694	-1.31763	0.01867
C	-2.2686	-1.86324	-0.89476
C	-1.38584	-1.78886	1.46136
H	-2.20633	-2.95066	-0.90482
H	-2.12516	-1.4951	-1.90924
H	-3.26107	-1.577	-0.54506
H	-2.33385	-1.42719	1.86169
H	-0.58002	-1.42341	2.09924
H	-1.38041	-2.87831	1.48479

Sum of electronic and zero-point Energies= -3073.916813
Sum of electronic and thermal Energies= -3073.904993

Sum of electronic and thermal Enthalpies= -3073.904049
Sum of electronic and thermal Free Energies= -3073.954309

1-BrIII-OTs (in Acetonitrile)
C 4.06538 2.37198 0.4061
C 4.32446 1.01175 0.51803
C 3.36019 0.09323 0.13059
C 2.16921 0.59498 -0.34628
C 1.85755 1.92712 -0.4719
C 2.84789 2.82391 -0.08323
H 4.82043 3.08553 0.70571
H 5.27299 0.66509 0.90677
H 0.89935 2.26338 -0.83695
H 2.6526 3.88371 -0.16477
Br 0.99973 -0.87622 -0.80444
O 2.52727 -1.91726 -0.74308
C 3.45025 -1.41701 0.22775
C 3.04671 -1.86915 1.62975
C 4.81247 -1.95657 -0.17201
H 2.05414 -1.49627 1.8873
H 3.7532 -1.48565 2.3658
H 5.57254 -1.6361 0.54016
H 3.04213 -2.9578 1.6732
H 4.78135 -3.04511 -0.17429
H 5.08212 -1.6066 -1.16657
O -0.62081 0.45497 -0.93877
S -1.26661 0.98431 0.34425
O -1.3231 2.41716 0.28428
O -0.63433 0.37282 1.48489
C -2.92256 0.37574 0.24413
C -3.88032 1.1133 -0.43567
C -3.24501 -0.83494 0.83633
C -5.17134 0.6205 -0.52991
H -3.61065 2.06712 -0.86768
C -4.54201 -1.31417 0.73477
H -2.48868 -1.37781 1.38669
C -5.51976 -0.59894 0.04794
H -5.92555 1.19512 -1.05321
H -4.80266 -2.25421 1.20497
C -6.91727 -1.1364 -0.08359
H -7.15826 -1.81334 0.7344
H -7.64964 -0.33036 -0.09216
H -7.02724 -1.69139 -1.01725
Sum of electronic and zero-point Energies= -3892.814206
Sum of electronic and thermal Energies= -3892.794390
Sum of electronic and thermal Enthalpies= -3892.793446
Sum of electronic and thermal Free Energies= -3892.863923

1-ClIII-anion (in Acetonitrile)
C -1.99366 -1.86739 0.055
C -0.61318 -1.71167 0.04228
C 0.02155 -0.4658 -0.00128
C -0.84285 0.6365 -0.01791
C -2.22495 0.50511 0.00274
C -2.81242 -0.7508 0.03434
H -2.42385 -2.86161 0.07985
H 0.00082 -2.60085 0.05677
H -2.83216 1.40042 -0.00781
H -3.89084 -0.84627 0.04248
O 1.89757 0.49769 -1.11877
C 1.57846 -0.30609 -0.11247
C 2.22682 -1.70954 -0.29777
C 2.07273 0.20834 1.27839
H 1.86513 -2.15738 -1.22573
H 2.07639 -2.41113 0.53156
H 1.8214 -0.46676 2.10481
H 3.29564 -1.52513 -0.4091
H 3.15902 0.30797 1.21397
H 1.65594 1.19357 1.48264
Cl -0.25783 2.27963 -0.04819
Sum of electronic and zero-point Energies= -884.306751
Sum of electronic and thermal Energies= -884.296547
Sum of electronic and thermal Enthalpies= -884.295603
Sum of electronic and thermal Free Energies= -884.342212

1-ClIII-Br (in Acetonitrile)
C -1.8223 2.76242 0.13137
C -2.39239 1.49703 0.18422
C -1.59486 0.3755 0.02286
C -0.24384 0.57281 -0.1672
C 0.36684 1.80157 -0.22836
C -0.45925 2.91129 -0.07954
H -2.44666 3.63719 0.24917
H -3.45664 1.37983 0.34146
H 1.43094 1.89428 -0.37672
H -0.02133 3.89809 -0.13062
O -1.01296 -1.75006 -0.68342
C -2.02601 -1.07331 0.04239
C -3.32707 -1.30934 -0.71011
C -2.10398 -1.5867 1.47895
H -3.25493 -0.91733 -1.72263
H -4.15786 -0.82682 -0.19465
H -2.86608 -1.04298 2.03759
H -3.5258 -2.37898 -0.75634
H -2.35547 -2.64662 1.47162
H -1.14826 -1.4561 1.98707
Br 2.89537 -0.17363 0.1186
Cl 0.59911 -1.01004 -0.33159
Sum of electronic and zero-point Energies= -3458.311456
Sum of electronic and thermal Energies= -3458.299587
Sum of electronic and thermal Enthalpies= -3458.298643
Sum of electronic and thermal Free Energies= -3458.350265

1-ClIII-CCH (in Acetonitrile)
C -0.75867 2.85352 0.0623
C -1.51443 1.69221 0.08433
C -0.90625 0.44248 0.00362
C 0.46365 0.43422 -0.07783
C 1.26313 1.55549 -0.10313
C 0.62458 2.78649 -0.03705
H -1.2485 3.81598 0.11906
H -2.59256 1.7464 0.16065
H 2.33721 1.48671 -0.17305
H 1.21761 3.68975 -0.06354
O -0.69841 -1.84267 -0.37052
C -1.62989 -0.90118 0.0046
C -2.78231 -0.88176 -1.0035
C -2.16254 -1.17352 1.41902
H -2.40009 -0.65272 -1.99737
H -3.55435 -0.15769 -0.73825
H -2.87842 -0.41486 1.74092
H -3.23052 -1.87451 -1.02555
H -2.65077 -2.14787 1.4236
H -1.33506 -1.19961 2.12882
C 4.04311 -0.67347 0.17411
H 5.09784 -0.59007 0.28718
C 2.85277 -0.75337 0.04949
Cl 1.16859 -1.24981 -0.13847
Sum of electronic and zero-point Energies= -960.789414
Sum of electronic and thermal Energies= -960.776767
Sum of electronic and thermal Enthalpies= -960.775823
Sum of electronic and thermal Free Energies= -960.828201

1-ClIII-CF3 (in Acetonitrile)
C 1.77949 2.72539 0.13049
C 2.31357 1.44724 0.16999
C 1.49797 0.33166 0.01622
C 0.15184 0.56019 -0.14467
C -0.4258 1.81232 -0.20451
C 0.41828 2.90627 -0.06671
H 2.4268 3.58442 0.24128
H 3.37715 1.30615 0.30901
H -1.47792 1.97069 -0.36406
H -0.00143 3.90086 -0.11904
O 0.94595 -1.83323 -0.60989
C -2.50983 -0.24904 0.06705
F -3.26598 -1.32915 0.20294
F -3.00096 0.4744 -0.93514
F -2.58216 0.46164 1.1837
C 1.96524 -1.12151 -0.01107
C 2.21279 -1.59213 1.4296
C 3.23889 -1.2628 -0.84535
H 2.51378 -2.63927 1.407
H 2.99271 -1.00687 1.91982
H 1.29622 -1.5105 2.01527
H 3.4822 -2.32227 -0.91523
H 4.08685 -0.74259 -0.39751
H 3.06914 -0.87922 -1.85037
Cl -0.79682 -0.98842 -0.27511
Sum of electronic and zero-point Energies= -1221.741008
Sum of electronic and thermal Energies= -1221.726991
Sum of electronic and thermal Enthalpies= -1221.726047
Sum of electronic and thermal Free Energies= -1221.781819

1-ClIII-CH3 (in Acetonitrile)
C 0.02117 2.87553 0.05945
C -0.94741 1.88552 0.07404
C -0.6093 0.53635 0.00104
C 0.73538 0.24096 -0.06147
C 1.73527 1.19589 -0.08173
C 1.36197 2.53153 -0.026
H -0.26798 3.91647 0.10913
H -1.99356 2.15493 0.13709
H 2.77972 0.94456 -0.14753
H 2.12687 3.29492 -0.05063
O -0.94071 -1.73865 -0.38795
C 2.94638 -1.41736 0.14309
C -1.61895 -0.61746 -0.01702
C -2.7368 -0.30829 -1.02413
C -2.21485 -0.74959 1.39733
H -3.39664 -1.1746 -1.06086
H -3.32899 0.56806 -0.75358
H -2.30741 -0.16165 -2.01475
H -2.92031 -1.58061 1.39339
H -2.73208 0.15612 1.72195
H -1.42152 -0.97787 2.11043

H 3.1787 -0.87382 1.05322
H 3.21989 -2.46412 0.24161
H 3.42599 -0.98445 -0.72875
Cl 1.12505 -1.52631 -0.09969
Sum of electronic and zero-point Energies= -923.960537
Sum of electronic and thermal Energies= -923.948438
Sum of electronic and thermal Enthalpies= -923.947494
Sum of electronic and thermal Free Energies= -923.998356

1-ClIII-CHCH2 (in Acetonitrile)
C -1.17625 2.77374 0.03536
C -1.77696 1.52404 0.06436
C -1.01827 0.3579 0.01235
C 0.34673 0.52225 -0.03238
C 0.99166 1.74123 -0.08316
C 0.20413 2.88448 -0.05205
H -1.7872 3.66573 0.06509
H -2.85491 1.44459 0.11362
H 2.06464 1.81733 -0.1674
H 0.67668 3.85533 -0.10282
O -0.56079 -1.86874 -0.50141
C -1.56902 -1.07426 -0.03839
C -2.77208 -1.13232 -0.9888
C -2.00861 -1.47038 1.38361
H -2.47405 -0.79309 -1.98032
H -3.61973 -0.53815 -0.64197
H -2.78871 -0.81496 1.77713
H -3.08576 -2.17316 -1.06141
H -2.38381 -2.49341 1.35332
H -1.15198 -1.44118 2.05874
C 2.88109 -0.32369 0.44511
H 2.89349 0.1977 1.39279
C 3.94242 -0.61731 -0.27763
H 4.93428 -0.33798 0.05279
H 3.8539 -1.15448 -1.21378
Cl 1.29062 -1.02344 -0.02617
Sum of electronic and zero-point Energies= -962.030985
Sum of electronic and thermal Energies= -962.017935
Sum of electronic and thermal Enthalpies= -962.016990
Sum of electronic and thermal Free Energies= -962.070424

1-ClIII-Cl (in Acetonitrile)
C -1.0353 2.8463 0.10212
C -1.72855 1.64348 0.14031
C -1.0388 0.44839 0.01229
C 0.33006 0.51115 -0.13203
C 1.06144 1.67346 -0.17392
C 0.34278 2.85936 -0.05896
H -1.57452 3.77896 0.19365
H -2.80391 1.63234 0.26125
H 2.13377 1.6575 -0.28268
H 0.87648 3.79848 -0.09705
O -0.62962 -1.73296 -0.64117
Cl 3.20041 -0.50829 0.19226
C -1.60711 -0.95194 0.0295
C -2.89022 -1.0762 -0.77726
C -1.79451 -1.43348 1.46679
H -2.52383 -0.81089 1.98543
H -0.85279 -1.38676 2.01408
H -2.14634 -2.46447 1.46062
H -3.18767 -2.1229 -0.81981
H -3.69317 -0.50876 -0.30621
H -2.73675 -0.7094 -1.79022
Cl 1.01764 -1.14604 -0.25948
Sum of electronic and zero-point Energies= -1344.310917
Sum of electronic and thermal Energies= -1344.299283
Sum of electronic and thermal Enthalpies= -1344.298338
Sum of electronic and thermal Free Energies= -1344.348501

1-ClIII-CN (in Acetonitrile)
C -0.61865 2.88725 0.08417
C -1.4338 1.76526 0.11758
C -0.88145 0.49546 0.00818
C 0.48423 0.41829 -0.11189
C 1.33906 1.49569 -0.14975
C 0.75598 2.75372 -0.05436
H -1.05729 3.87245 0.16169
H -2.50552 1.87082 0.22279
H 2.40774 1.38287 -0.24842
H 1.38998 3.62817 -0.09019
O -0.73945 -1.75189 -0.53718
C 2.84056 -0.84797 0.09587
N 3.97149 -0.74351 0.2753
C -1.62814 -0.82735 0.01844
C -2.8745 -0.77279 -0.8581
C -1.98498 -1.20287 1.45963
H -2.60729 -0.47501 -1.87056
H -3.61229 -0.07571 -0.45938
H -2.6519 -0.4651 1.9076
H -1.08323 -1.26972 2.06931
H -2.47695 -2.17501 1.46085
H -3.32076 -1.76573 -0.89165
Cl 1.03781 -1.31156 -0.20884
Sum of electronic and zero-point Energies= -976.902499
Sum of electronic and thermal Energies= -976.889912
Sum of electronic and thermal Enthalpies= -976.888968
Sum of electronic and thermal Free Energies= -976.941049

1-ClIII-F (in Acetonitrile)
C 0.93069 2.72119 0.00009
C -0.30996 2.10196 0.00008
C -0.39021 0.71587 -0.00008
C 0.78564 0.00564 -0.0002
C 2.04203 0.5745 -0.00015
C 2.09468 1.96236 -0.00003
H 0.99196 3.80065 0.00019
H -1.21839 2.69073 0.0002
H 2.92494 -0.04148 -0.0002
H 3.05958 2.44971 0.
O -1.23938 -1.46019 -0.00114
F 2.27896 -2.05335 0.00037
C -1.65624 -0.10275 0.00002
C -2.47708 0.15576 -1.26006
C -2.47581 0.15427 1.26126
H -1.87852 -0.04997 -2.14626
H -2.81701 1.19143 -1.29527
H -2.81575 1.18989 1.29803
H -3.34752 -0.49932 -1.2633
H -3.34622 -0.50086 1.26458
H -1.8764 -0.05246 2.14665
Cl 0.49796 -1.74997 -0.00026
Sum of electronic and zero-point Energies= -983.942758
Sum of electronic and thermal Energies= -983.931662
Sum of electronic and thermal Enthalpies= -983.930717
Sum of electronic and thermal Free Energies= -983.979042

1-ClIII-N3 (in Acetonitrile)
C 0.93564 2.87637 0.20469
C 1.6766 1.71122 0.35449
C 1.10354 0.48608 0.0501
C -0.20434 0.48266 -0.37768
C -0.97891 1.60723 -0.5439
C -0.37582 2.82461 -0.24631
H 1.38642 3.83168 0.43531
H 2.70141 1.75362 0.69952
H -1.99597 1.54158 -0.89582
H -0.94404 3.73535 -0.37218
O 0.97054 -1.68882 -0.73062
C 1.74098 -0.88482 0.13829
C 1.66932 -1.40652 1.57397
C 3.16901 -0.89411 -0.38741
H 0.63659 -1.43201 1.92281
H 2.2427 -0.76727 2.24593
H 3.82013 -0.29727 0.25202
H 2.07546 -2.41681 1.60997
H 3.53927 -1.91825 -0.39555
H 3.19986 -0.49935 -1.4012
N -3.35043 -0.33499 1.5211
N -3.03357 -0.50327 0.45013
N -2.70518 -0.66932 -0.70709
Cl -0.77753 -1.19318 -0.68837
Sum of electronic and zero-point Energies= -1048.262089
Sum of electronic and thermal Energies= -1048.248894
Sum of electronic and thermal Enthalpies= -1048.247950
Sum of electronic and thermal Free Energies= -1048.301986

1-ClIII-NH2 (in Acetonitrile)
C 0.18686 2.88707 -0.04876
C -0.84524 1.96119 -0.05645
C -0.5758 0.59886 0.00815
C 0.74742 0.22318 0.06102
C 1.80733 1.10484 0.06482
C 1.50567 2.46017 0.01599
H -0.03716 3.94426 -0.09134
H -1.87415 2.29287 -0.10759
H 2.82206 0.74642 0.09809
H 2.31307 3.17904 0.02761
O -0.94009 -1.65111 0.43118
C -1.6092 -0.51722 0.0092
C -2.15734 -0.69058 -1.41459
C -2.74692 -0.20756 0.98331
N 2.78667 -1.49041 -0.13563
H -3.41742 -1.06587 1.0117
H -3.32036 0.6702 0.68092
H -2.34509 -0.04783 1.98287
H -2.86915 -1.51603 -1.41762
H -2.65645 0.21243 -1.7709
H -1.3442 -0.93354 -2.09966
H 2.94944 -1.98346 -1.00768
H 3.15914 -2.06894 0.6106
Cl 0.98575 -1.57102 0.10863
Sum of electronic and zero-point Energies= -940.003623
Sum of electronic and thermal Energies= -939.991634
Sum of electronic and thermal Enthalpies= -939.990689
Sum of electronic and thermal Free Energies= -940.041304

1-ClIII-NHAc (in Acetonitrile)

C	-1.61887	2.78535	0.2108
C	-2.21609	1.54091	0.35354
C	-1.50971	0.3897	0.0347
C	-0.21542	0.54025	-0.40362
C	0.42411	1.74853	-0.549
C	-0.31072	2.88727	-0.24125
H	-2.17555	3.68012	0.45322
H	-3.23511	1.46013	0.70859
H	1.4461	1.80615	-0.88354
H	0.15311	3.85728	-0.35258
O	-1.16149	-1.75697	-0.74014
C	2.94421	-0.20024	0.39129
O	2.34802	-0.05517	1.43836
C	4.45756	-0.21499	0.32007
H	4.79863	-1.24726	0.40921
H	4.83117	0.19185	-0.61806
H	4.85316	0.35244	1.15735
C	-1.9919	-1.04495	0.13393
C	-3.43054	-1.20151	-0.34113
C	-1.8269	-1.53521	1.57594
H	-3.53416	-0.82001	-1.35563
H	-4.12419	-0.67446	0.31508
H	-3.68884	-2.25973	-0.33616
H	-2.11157	-2.58592	1.62902
H	-2.45367	-0.96057	2.25922
H	-0.78835	-1.43463	1.89423
N	2.28766	-0.34857	-0.79526
H	2.81417	-0.65808	-1.59995
Cl	0.55734	-1.05535	-0.74179

Sum of electronic and zero-point Energies= -1092.633152
Sum of electronic and thermal Energies= -1092.617812
Sum of electronic and thermal Enthalpies= -1092.616868
Sum of electronic and thermal Free Energies= -1092.676093

1-ClIII-OCOCH3 (in Acetonitrile)

C	-2.35629	2.54706	0.13828
C	-2.6879	1.19966	0.19278
C	-1.69749	0.24374	0.03378
C	-0.40396	0.67671	-0.16016
C	-0.02698	2.00009	-0.21907
C	-1.04154	2.93997	-0.07008
H	-3.71325	0.89143	0.35011
H	1.00051	2.28256	-0.36832
H	-0.79164	3.99052	-0.11965
O	-0.71098	-1.73401	-0.64371
C	3.31051	-0.20102	0.06609
O	3.3597	-1.40567	-0.06153
O	2.20414	0.50313	0.00935
C	4.53742	0.64976	0.30761
H	4.65313	1.35959	-0.51051
H	4.41232	1.22105	1.22616
H	5.41431	0.0136	0.37575
C	-1.85072	-1.26044	0.06144
C	-3.07106	-1.74402	-0.70427
C	-1.84776	-1.77172	1.50077
H	-3.06006	-1.3524	-1.71958
H	-3.98638	-1.42508	-0.20524
H	-3.06184	-2.8323	-0.74381
H	-1.89688	-2.85995	1.49888
H	-2.70444	-1.37631	2.04701
H	-0.93852	-1.46325	2.01695
Cl	0.69372	-0.72533	-0.31865
H	-3.12878	3.29476	0.25334

Sum of electronic and zero-point Energies= -1112.527042
Sum of electronic and thermal Energies= -1112.511834
Sum of electronic and thermal Enthalpies= -1112.510890
Sum of electronic and thermal Free Energies= -1112.570225

1-ClIII-OH (in Acetonitrile)

C	0.85748	2.76384	0.07026
C	-0.36814	2.1117	0.08851
C	-0.41815	0.72804	-0.00036
C	0.77592	0.05004	-0.08434
C	2.01497	0.65101	-0.10392
C	2.03745	2.0394	-0.03067
H	0.89247	3.843	0.12974
H	-1.28802	2.67691	0.16429
H	2.91291	0.0603	-0.17723
H	2.98909	2.5517	-0.05634
O	-1.21878	-1.37162	-0.5408
O	2.29907	-2.06667	0.12688
H	2.37954	-2.20681	1.07596
C	-1.65087	-0.15336	0.00083
C	-2.74977	0.40254	-0.89641
C	-2.1539	-0.33763	1.43563
H	-3.56965	-0.31363	-0.93511
H	-2.36825	0.55325	-1.90477
H	-3.13438	1.34793	-0.51166
H	-2.45388	0.61541	1.87342
H	-1.37143	-0.77296	2.0581
H	-3.0093	-1.0127	1.43054
Cl	0.54757	-1.72762	-0.15957

Sum of electronic and zero-point Energies= -959.891776
Sum of electronic and thermal Energies= -959.880237
Sum of electronic and thermal Enthalpies= -959.879293
Sum of electronic and thermal Free Energies= -959.928399

1-ClIII-OTs (in Acetonitrile)

C	4.11232	2.19583	0.4869
C	4.33131	0.82555	0.56974
C	3.37472	-0.04328	0.07401
C	2.23697	0.50475	-0.47294
C	1.96522	1.84548	-0.57731
C	2.94845	2.69626	-0.07939
H	4.85735	2.87922	0.86987
H	5.23752	0.43686	1.01538
H	1.0424	2.21483	-0.99481
H	2.78682	3.76325	-0.13509
O	2.50212	-1.89018	-1.01399
C	3.37625	-1.55308	0.08451
C	2.80462	-2.07635	1.39748
C	4.71906	-2.16682	-0.25714
H	1.82281	-1.64264	1.59521
H	3.46665	-1.80364	2.21918
H	5.43663	-1.94709	0.53304
H	2.72061	-3.16172	1.3542
H	4.61744	-3.24807	-0.33697
H	5.09188	-1.77099	-1.19967
O	-0.47385	0.45686	-1.05872
S	-1.05135	0.83521	0.29239
O	-1.07086	2.26552	0.43704
O	-0.39979	0.06877	1.32942
C	-2.73152	0.28925	0.19526
C	-3.70559	1.15456	-0.27991
C	-3.05788	-1.00012	0.58369
C	-5.01535	0.71375	-0.37492
H	-3.43145	2.16402	-0.55356
C	-4.37318	-1.42757	0.48371
H	-2.28778	-1.64872	0.97859
C	-5.36749	-0.58154	-0.00014
H	-5.78116	1.38811	-0.73819
H	-4.63448	-2.43127	0.79526
C	-6.78837	-1.05435	-0.13208
H	-6.97793	-1.91822	0.50273
H	-7.49098	-0.26698	0.13858
H	-7.00195	-1.34452	-1.16253
Cl	1.17113	-0.83338	-1.01871

Sum of electronic and zero-point Energies= -1778.796198
Sum of electronic and thermal Energies= -1778.774822
Sum of electronic and thermal Enthalpies= -1778.773878
Sum of electronic and thermal Free Energies= -1778.849434

1-IIII-anion (in Acetonitrile)

C	-2.4998	2.1716	0.0302
C	-2.45729	0.78472	0.01134
C	-1.26168	0.05691	-0.02342
C	-0.09485	0.8201	-0.01817
C	-0.1128	2.20966	0.0127
C	-1.31833	2.89563	0.02836
H	-3.45547	2.68223	0.04486
H	-3.39222	0.24197	0.01433
H	0.82259	2.75306	0.0222
H	-1.32603	3.97837	0.03922
O	-0.27114	-1.95669	-0.87659
C	-1.23813	-1.51271	-0.08428
C	-2.62464	-2.02859	-0.5668
C	-1.07505	-1.98249	1.39621
H	-2.8487	-1.6085	-1.5495
H	-3.46316	-1.83606	0.11142
H	-1.872	-1.62536	2.05807
H	-2.51023	-3.10704	-0.67749
H	-1.07057	-3.07501	1.39119
H	-0.11291	-1.64243	1.78232
I	1.83949	-0.03013	-0.01775

Sum of electronic and zero-point Energies= -721.735661
Sum of electronic and thermal Energies= -721.725023
Sum of electronic and thermal Enthalpies= -721.724078
Sum of electronic and thermal Free Energies= -721.772957

1-IIII-Br (in Acetonitrile)

C	-2.26944	2.91298	0.00213
C	-2.7451	1.61054	0.05462
C	-1.86509	0.53521	-0.00112
C	-0.51884	0.83066	-0.0972
C	-0.00411	2.10767	-0.14503
C	-0.90787	3.16203	-0.09948
H	-2.96647	3.73889	0.04019
H	-3.80813	1.42648	0.13924
H	1.06002	2.27878	-0.21469
H	-0.53991	4.17754	-0.14448
O	-1.23164	-1.7137	-0.43096
C	-2.28324	-0.92759	0.09164
C	-3.51243	-1.22026	-0.76009
C	-2.53335	-1.28709	1.55783
H	-3.33236	-0.92621	-1.79264

H	-4.39008	-0.69433	-0.3846
H	-3.34299	-0.68641	1.97313
H	-3.71286	-2.29023	-0.72927
H	-2.7978	-2.3417	1.62848
H	-1.63679	-1.1106	2.15405
Br	2.91151	0.38715	0.14545
I	0.62744	-0.93882	-0.14967

Sum of electronic and zero-point Energies= -3295.810035
Sum of electronic and thermal Energies= -3295.797797
Sum of electronic and thermal Enthalpies= -3295.796852
Sum of electronic and thermal Free Energies= -3295.849982

1-IIII-CCH (in Acetonitrile)

C	-2.06861	2.77849	-0.00223
C	-2.43367	1.44138	0.02349
C	-1.4665	0.43982	-0.01244
C	-0.15009	0.84366	-0.06049
C	0.256	2.1627	-0.0807
C	-0.72871	3.14053	-0.05684
H	-2.8328	3.5435	0.02073
H	-3.48004	1.1695	0.07321
H	1.30421	2.42253	-0.11454
H	-0.4447	4.18348	-0.08076
O	-0.6402	-1.76723	-0.31776
C	-1.78414	-1.05653	0.04496
C	-2.89592	-1.41291	-0.94031
C	-2.1964	-1.41393	1.47768
H	-2.60546	-1.12351	-1.94924
H	-3.83861	-0.92789	-0.68596
H	-3.08708	-0.86553	1.7875
H	-3.04422	-2.49183	-0.91684
H	-2.39878	-2.48339	1.53097
H	-1.38695	-1.17884	2.1704
C	3.84766	1.18973	0.23336
H	4.74568	1.75236	0.33465
C	2.82977	0.55605	0.12045
I	1.18743	-0.79012	-0.07754

Sum of electronic and zero-point Energies= -798.285049
Sum of electronic and thermal Energies= -798.271880
Sum of electronic and thermal Enthalpies= -798.270936
Sum of electronic and thermal Free Energies= -798.325284

1-IIII-CF3 (in Acetonitrile)

C	2.27137	2.86797	-0.00009
C	2.72746	1.56109	-0.00024
C	1.83249	0.49234	0.00001
C	0.48897	0.79527	0.00031
C	-0.00499	2.08862	0.00048
C	0.90763	3.13249	0.0003
H	2.97965	3.68516	-0.00027
H	3.79121	1.36061	-0.00055
H	-1.06138	2.30229	0.00078
H	0.54713	4.15166	0.00045
O	1.17317	-1.80125	0.00121
C	-2.59526	0.2969	-0.00035
F	-3.59297	-0.60067	-0.00083
F	-2.76701	1.07511	-1.07717
F	-2.76778	1.07477	1.07665
C	2.29105	-0.96672	-0.00017
C	3.11736	-1.24224	1.25872
C	3.11481	-1.24258	-1.26067
H	3.40762	-2.29231	1.26322
H	4.01748	-0.62766	1.29545
H	2.51831	-1.0431	2.14698
H	3.4053	-2.29258	-1.26539
H	4.01468	-0.62777	-1.29952
H	2.51387	-1.04391	-2.14778
I	-0.72216	-0.9397	0.00036

Sum of electronic and zero-point Energies= -1059.226703
Sum of electronic and thermal Energies= -1059.211970
Sum of electronic and thermal Enthalpies= -1059.211026
Sum of electronic and thermal Free Energies= -1059.269304

1-IIII-CH3 (in Acetonitrile)

C	-2.50292	2.3336	0.00008
C	-2.56795	0.95015	0.00013
C	-1.40848	0.17603	-0.00005
C	-0.20393	0.8445	-0.00019
C	-0.10246	2.22552	-0.00023
C	-1.27038	2.97377	-0.00012
H	-3.41425	2.91608	0.0002
H	-3.53199	0.45746	0.00031
H	0.85447	2.72311	-0.00037
H	-1.21335	4.05357	-0.0002
O	-0.13735	-1.83753	-0.0011
C	2.85571	1.27624	0.0006
C	-1.43324	-1.3577	0.00003
C	-2.16189	-1.84555	-1.25846
C	-2.15981	-1.84533	1.25981
H	-2.15135	-2.9352	-1.26312
H	-3.197	-1.50258	-1.29655
H	-1.63917	-1.49087	-2.14673
H	-2.14948	-2.93498	1.26457
H	-3.19478	-1.50211	1.29966
H	-1.6355	-1.49065	2.14715
H	2.73829	1.878	0.89716
H	3.8398	0.81072	0.00098
H	2.73909	1.87822	-0.89593
I	1.48653	-0.42543	-0.00015

Sum of electronic and zero-point Energies= -761.433408
Sum of electronic and thermal Energies= -761.421732
Sum of electronic and thermal Enthalpies= -761.420788
Sum of electronic and thermal Free Energies= -761.471002

1-IIII-CHCH2 (in Acetonitrile)

C	-2.28102	2.67292	0.00235
C	-2.5721	1.317	0.0108
C	-1.55193	0.36892	0.02809
C	-0.25711	0.83951	0.01414
C	0.07194	2.18183	0.01386
C	-0.96225	3.10782	0.01211
H	-3.08648	3.39483	-0.00389
H	-3.60364	0.98957	0.00808
H	1.10208	2.5072	0.03321
H	-0.73423	4.16476	0.02286
O	-0.6045	-1.77524	0.40377
C	-1.78575	-1.14787	0.04008
C	-2.21757	-1.58202	-1.3678
C	-2.86275	-1.51291	1.0631
H	-1.4372	-1.33803	-2.09044
H	-3.1421	-1.09181	-1.67737
H	-3.83649	-1.0929	0.80865
H	-2.3663	-2.66172	-1.37235
H	-2.95112	-2.59833	1.09203
H	-2.56837	-1.1626	2.05154
C	2.75796	0.74116	-0.41363
H	2.71415	1.29233	-1.347
C	3.80348	0.83161	0.39185
H	3.85484	0.28159	1.3253
H	4.65678	1.45698	0.1516
I	1.19579	-0.69524	-0.0189

Sum of electronic and zero-point Energies= -799.503402
Sum of electronic and thermal Energies= -799.489928
Sum of electronic and thermal Enthalpies= -799.488984
Sum of electronic and thermal Free Energies= -799.544109

1-IIII-Cl (in Acetonitrile)

C	-2.22479	2.69818	0.00019
C	-2.53636	1.34793	0.00037
C	-1.52855	0.387	-0.00004
C	-0.22699	0.8448	-0.00047
C	0.12648	2.17911	-0.00063
C	-0.89935	3.11363	-0.00036
H	-3.01968	3.43131	0.00052
H	-3.57173	1.03147	0.00084
H	1.16377	2.47806	-0.00095
H	-0.65691	4.16715	-0.00057
O	-0.58103	-1.81346	-0.0021
Cl	3.07199	0.81675	0.00091
C	-1.80636	-1.10993	0.0002
C	-2.58035	-1.49878	-1.25867
C	-2.57627	-1.49903	1.2615
H	-3.54684	-1.00383	1.3024
H	-2.00432	-1.22488	2.14742
H	-2.73076	-2.57735	1.2643
H	-2.73444	-2.57716	-1.2614
H	-3.55125	-1.004	-1.29611
H	-2.01142	-1.22406	-2.14634
I	1.14346	-0.74814	-0.0004

Sum of electronic and zero-point Energies= -1181.810367
Sum of electronic and thermal Energies= -1181.798306
Sum of electronic and thermal Enthalpies= -1181.797361
Sum of electronic and thermal Free Energies= -1181.849410

1-IIII-CN (in Acetonitrile)

C	-2.03536	2.78144	0.00004
C	-2.40366	1.44631	0.0003
C	-1.43775	0.44182	-0.00017
C	-0.12102	0.84463	-0.00069
C	0.29096	2.16306	-0.00092
C	-0.6931	3.14069	-0.00062
H	-2.79796	3.54811	0.00036
H	-3.45141	1.17427	0.0009
H	1.33806	2.42983	-0.00131
H	-0.40496	4.18253	-0.00087
O	-0.59937	-1.79684	-0.00345
C	2.86385	0.60478	0.0013
N	3.77955	1.30173	0.00239
C	-1.78497	-1.04547	0.00042
C	-2.58433	-1.38936	-1.25699
C	-2.57706	-1.38958	1.26241
H	-2.00456	-1.14388	-2.14633
H	-3.52938	-0.84666	-1.29224
H	-3.52149	-0.84619	1.30358
H	-1.99189	-1.14489	2.14846
H	-2.78568	-2.45877	1.26627

H	-2.7923	-2.45867	-1.26012
I	1.19197	-0.80685	-0.00072

Sum of electronic and zero-point Energies= -814.406256
Sum of electronic and thermal Energies= -814.393299
Sum of electronic and thermal Enthalpies= -814.392355
Sum of electronic and thermal Free Energies= -814.446301

1-IIII-F (in Acetonitrile)

C	-2.67415	2.10311	0.00008
C	-2.61537	0.71778	0.00012
C	-1.38589	0.06496	-0.00013
C	-0.25552	0.85364	-0.00033
C	-0.26904	2.23578	-0.00033
C	-1.50832	2.86008	-0.00017
H	-3.63604	2.59736	0.00025
H	-3.5294	0.1374	0.00035
H	0.65594	2.79207	-0.00044
H	-1.56054	3.93999	-0.00022
O	0.14024	-1.7845	-0.00205
F	2.47779	1.45033	0.00118
C	-1.23354	-1.45184	0.0001
C	-1.86863	-2.04079	-1.25852
C	-1.86486	-2.03983	1.2611
H	-1.39732	-1.61922	-2.14583
H	-2.93845	-1.83348	-1.29661
H	-2.93447	-1.83205	1.30248
H	-1.71845	-3.11976	-1.26124
H	-1.71505	-3.11886	1.26402
H	-1.39059	-1.61787	2.14665
I	1.48669	-0.28151	-0.00028

Sum of electronic and zero-point Energies= -821.447599
Sum of electronic and thermal Energies= -821.436017
Sum of electronic and thermal Enthalpies= -821.435073
Sum of electronic and thermal Free Energies= -821.485457

1-IIII-N3 (in Acetonitrile)

C	1.92099	2.92775	-0.098
C	2.40272	1.64523	0.12359
C	1.55144	0.54899	0.03526
C	0.22908	0.8026	-0.26577
C	-0.29085	2.06048	-0.49002
C	0.5833	3.13672	-0.40652
H	2.595	3.77081	-0.03172
H	3.44676	1.49501	0.36564
H	-1.33386	2.1968	-0.73582
H	0.21425	4.13694	-0.58546
O	0.99219	-1.73733	-0.28847
C	1.96435	-0.89784	0.29369
C	2.03174	-1.13577	1.80438
C	3.29528	-1.22922	-0.37053
H	1.06564	-0.92895	2.26737
H	2.77813	-0.49089	2.26914
H	4.11292	-0.65575	0.06602
H	2.29223	-2.17707	1.99162
H	3.50272	-2.28879	-0.22771
H	3.24279	-1.02316	-1.43828
N	-3.42036	1.1571	1.56201
N	-3.06895	0.68415	0.60243
N	-2.72668	0.20272	-0.4622
I	-0.89567	-0.97354	-0.32651

Sum of electronic and zero-point Energies= -885.761338
Sum of electronic and thermal Energies= -885.747734
Sum of electronic and thermal Enthalpies= -885.746790
Sum of electronic and thermal Free Energies= -885.802704

1-IIII-NH2 (in Acetonitrile)

C	-2.64585	2.18753	0.00506
C	-2.62875	0.80055	0.00847
C	-1.42325	0.10503	-0.02308
C	-0.26148	0.8487	-0.04511
C	-0.24275	2.22969	-0.04041
C	-1.45643	2.90367	-0.02151
H	-3.59098	2.713	0.02392
H	-3.5611	0.25129	0.03701
H	0.69027	2.77416	-0.05348
H	-1.46938	3.98493	-0.02737
O	-0.00578	-1.77244	-0.33208
C	-1.30926	-1.41972	0.01222
C	-2.25986	-2.05888	-0.99906
C	-1.63119	-1.90115	1.43237
N	2.76847	1.2778	0.17477
H	-1.5255	-2.98517	1.47025
H	-2.64555	-1.63008	1.72915
H	-0.93202	-1.45965	2.14414
H	-2.10198	-3.1367	-0.98811
H	-3.30529	-1.85905	-0.76168
H	-2.0428	-1.68604	-1.99912
H	2.62842	1.81213	1.02309
H	2.78815	1.90923	-0.61604
I	1.48845	-0.33256	-0.04759

Sum of electronic and zero-point Energies= -777.498791
Sum of electronic and thermal Energies= -777.486533
Sum of electronic and thermal Enthalpies= -777.485589
Sum of electronic and thermal Free Energies= -777.537441

1-IIII-NHAc (in Acetonitrile)

C	-1.76439	3.1231	-0.14159
C	-2.4188	1.92207	0.08744
C	-1.72937	0.71575	0.00887
C	-0.38518	0.77592	-0.29453
C	0.30343	1.9504	-0.51384
C	-0.40863	3.13963	-0.44225
H	-2.31366	4.05279	-0.0812
H	-3.473	1.92037	0.33204
H	1.35983	1.94182	-0.73497
H	0.1003	4.07753	-0.61613
O	-1.50369	-1.6324	-0.25107
C	3.05035	0.29678	0.5296
O	2.4975	0.46274	1.59837
C	4.47763	0.74628	0.28571
H	5.0506	-0.01581	-0.2409
H	4.47215	1.64935	-0.32648
H	4.94422	0.97026	1.23983
C	-2.34649	-0.65247	0.29482
C	-3.71184	-0.79899	-0.36891
C	-2.45522	-0.83261	1.81246
H	-3.62579	-0.63265	-1.44166
H	-4.43912	-0.10049	0.04516
H	-4.07278	-1.8128	-0.20035
H	-2.86168	-1.82117	2.02515
H	-3.10435	-0.07576	2.25445
H	-1.47013	-0.7519	2.27481
N	2.41256	-0.26786	-0.5332
H	2.92678	-0.35448	-1.39683
I	0.49055	-1.13453	-0.33445

Sum of electronic and zero-point Energies= -930.134267
Sum of electronic and thermal Energies= -930.118452
Sum of electronic and thermal Enthalpies= -930.117508
Sum of electronic and thermal Free Energies= -930.178619

1-IIII-OCOCH3 (in Acetonitrile)

C	-2.5956	2.77339	0.00029
C	-2.94095	1.43179	0.00084
C	-1.95688	0.44678	0.00012
C	-0.64019	0.86247	-0.00098
C	-0.25779	2.19318	-0.0015
C	-1.2593	3.15307	-0.0009
H	-3.37105	3.52715	0.00081
H	-3.98401	1.14168	0.00182
H	0.78475	2.46654	-0.00231
H	-0.99051	4.20035	-0.00136
O	-1.07028	-1.77733	-0.00301
C	3.39473	0.18825	0.00093
O	3.56557	-1.01383	0.00016
O	2.20214	0.73897	0.00068
C	4.52398	1.18671	0.00262
H	4.44234	1.82733	-0.87425
H	4.44441	1.82133	0.88407
H	5.47531	0.66496	-0.00007
C	-2.27598	-1.04248	0.00093
C	-3.06269	-1.41102	-1.25629
C	-3.0552	-1.4109	1.26289
H	-2.48813	-1.15182	-2.14504
H	-4.02055	-0.89126	-1.29263
H	-3.2444	-2.48512	-1.25822
H	-3.23757	-2.48488	1.26573
H	-4.01244	-0.89045	1.30529
H	-2.47503	-1.15225	2.14818
I	0.67647	-0.76233	-0.00129

Sum of electronic and zero-point Energies= -950.029502
Sum of electronic and thermal Energies= -950.013813
Sum of electronic and thermal Enthalpies= -950.012869
Sum of electronic and thermal Free Energies= -950.074041

1-IIII-OH (in Acetonitrile)

C	-2.7009	2.09867	0.00306
C	-2.64028	0.71221	0.01324
C	-1.41128	0.06105	-0.01864
C	-0.27989	0.84962	-0.04615
C	-0.29731	2.23025	-0.05404
C	-1.53698	2.85617	-0.03343
H	-3.6632	2.59206	0.02147
H	-3.55386	0.13264	0.04593
H	0.62746	2.7873	-0.08628
H	-1.59022	3.93604	-0.04964
O	0.08234	-1.75232	-0.38003
O	2.55383	1.43518	0.14692
H	2.68795	1.63099	1.07793
C	-1.22879	-1.45497	0.02447
C	-2.17789	-2.15397	-0.94401
C	-1.45989	-1.9415	1.45854
H	-1.9624	-3.22166	-0.93375
H	-2.02419	-1.77641	-1.95373
H	-3.22089	-2.00837	-0.66206
H	-2.47184	-1.71365	1.79575
H	-0.75398	-1.46118	2.13788

H	-1.30336	-3.01917	1.49929
I	1.48185	-0.28088	-0.06502

Sum of electronic and zero-point Energies= -797.395703
Sum of electronic and thermal Energies= -797.383630
Sum of electronic and thermal Enthalpies= -797.382686
Sum of electronic and thermal Free Energies= -797.434029

1-IIII-OTs (in Acetonitrile)

C	-4.01903	2.57937	-0.27635
C	-4.33342	1.23464	-0.41612
C	-3.38116	0.26118	-0.13911
C	-2.13473	0.69818	0.26526
C	-1.77603	2.02109	0.41083
C	-2.75186	2.97076	0.13332
H	-4.76895	3.32801	-0.49211
H	-5.32148	0.94071	-0.74523
H	-0.78379	2.3155	0.72035
H	-2.51073	4.01919	0.23801
O	-2.62333	-1.90703	0.48139
C	-3.5928	-1.23787	-0.31578
C	-3.4009	-1.60206	-1.78847
C	-4.95289	-1.69107	0.19366
H	-2.41127	-1.29946	-2.13494
H	-4.14395	-1.09923	-2.40758
H	-5.75798	-1.26322	-0.40306
H	-3.5039	-2.67993	-1.91033
H	-5.01209	-2.77624	0.12355
H	-5.08264	-1.39735	1.23379
O	0.76735	0.44304	0.84357
S	1.47108	1.11279	-0.34904
O	1.5411	2.52323	-0.10593
O	0.86904	0.64914	-1.56988
C	3.10944	0.46168	-0.25855
C	4.04479	1.08048	0.55901
C	3.4402	-0.66362	-0.99513
C	5.32105	0.55207	0.64266
H	3.76902	1.97191	1.10533
C	4.72431	-1.18046	-0.90091
H	2.70301	-1.11066	-1.64815
C	5.67831	-0.58574	-0.08083
H	6.0583	1.03338	1.27337
H	4.99201	-2.05409	-1.48172
C	7.06418	-1.15536	0.03629
H	7.26598	-1.87139	-0.75809
H	7.81581	-0.36783	-0.01072
H	7.18812	-1.66909	0.99119
I	-0.88119	-0.94248	0.60904

Sum of electronic and zero-point Energies= -1616.291583
Sum of electronic and thermal Energies= -1616.270793
Sum of electronic and thermal Enthalpies= -1616.269849
Sum of electronic and thermal Free Energies= -1616.343876

2-BrIII-anion (in Acetonitrile)

C	0.91381	3.0276	0.58522
C	-0.04312	2.05223	0.3629
C	0.28058	0.737	0.01423
C	1.64059	0.4371	-0.06494
C	2.61414	1.40806	0.15871
C	2.25762	2.70607	0.47515
H	0.60728	4.0331	0.8437
H	-1.08335	2.32186	0.465
H	3.65523	1.12679	0.08477
H	3.02472	3.4519	0.64006
O	-0.4828	-1.28649	-1.09968
C	-0.8315	-0.33717	-0.28763
C	-2.09934	0.39646	-0.88453
C	-1.25747	-0.89733	1.11795
F	-0.27543	-1.6422	1.63543
F	-1.53984	0.04376	2.05103
F	-2.34346	-1.67811	1.03963
F	-2.95906	-0.48277	-1.39215
F	-2.83265	1.15453	-0.02248
F	-1.7486	1.2189	-1.88661
Br	2.34566	-1.29568	-0.42635

Sum of electronic and zero-point Energies= -3593.929904
Sum of electronic and thermal Energies= -3593.915282
Sum of electronic and thermal Enthalpies= -3593.914338
Sum of electronic and thermal Free Energies= -3593.972759

2-BrIII-Br (in Acetonitrile)

C	-0.47339	3.37971	-0.01403
C	-1.28184	2.25306	-0.01052
C	-0.70918	0.98656	-0.00149
C	0.66886	0.90555	0.00251
C	1.50293	1.99845	-0.00088
C	0.90669	3.25336	-0.00896
H	-0.92706	4.36065	-0.02077
H	-2.35676	2.35259	-0.01497
H	2.5753	1.88668	0.00247
H	1.53646	4.13184	-0.01147
Br	1.31673	-0.9275	0.01205
O	-0.60468	-1.38736	0.02369
C	-1.4746	-0.32518	0.00363
C	-2.32241	-0.44355	-1.28674
C	-2.34819	-0.42083	1.27867
F	-2.95031	-1.60769	-1.35466
F	-3.23969	0.52402	-1.38379
F	-1.51681	-0.34491	-2.34349
F	-3.27659	0.53959	1.33289
F	-2.96653	-1.58906	1.36088
F	-1.56694	-0.28921	2.34978
Br	3.73607	-0.39394	0.00017

Sum of electronic and zero-point Energies= -6167.925613
Sum of electronic and thermal Energies= -6167.909334
Sum of electronic and thermal Enthalpies= -6167.908390
Sum of electronic and thermal Free Energies= -6167.972452

2-BrIII-CCH (in Acetonitrile)

C	0.31466	3.28823	0.0845
C	-0.5932	2.24095	0.06278
C	-0.14762	0.92262	0.00987
C	1.21353	0.72378	-0.01257
C	2.14947	1.73241	0.00883
C	1.6779	3.03733	0.05635
H	-0.04703	4.30613	0.12375
H	-1.65354	2.44115	0.08721
H	3.20722	1.52094	-0.01054
H	2.38649	3.85352	0.07201
Br	1.75427	-1.15805	-0.07227
O	-0.30418	-1.43777	-0.13511
C	-1.04796	-0.31703	-0.02405
C	-1.98818	-0.23191	-1.25322
C	-1.85556	-0.38352	1.29751
C	4.81392	-0.73148	0.01067
H	5.87717	-0.685	0.03741
C	3.61549	-0.77638	-0.01863
F	-1.00269	-0.45277	2.32266
F	-2.63642	-1.4537	1.342
F	-2.63004	0.69164	1.49853
F	-2.71998	-1.32858	-1.38374
F	-2.82977	0.81109	-1.20219
F	-1.25342	-0.09356	-2.35856

Sum of electronic and zero-point Energies= -3670.416769
Sum of electronic and thermal Energies= -3670.399762
Sum of electronic and thermal Enthalpies= -3670.398818
Sum of electronic and thermal Free Energies= -3670.462812

2-BrIII-CF3 (in Acetonitrile)

C	-0.4398	3.3486	-0.29681
C	-1.23467	2.21645	-0.22442
C	-0.65979	0.96217	-0.04132
C	0.7136	0.89936	0.04138
C	1.53875	2.00383	-0.02118
C	0.93782	3.24359	-0.18953
H	-0.89998	4.31696	-0.43371
H	-2.30768	2.30022	-0.30762
H	2.60957	1.94155	0.06186
H	1.56117	4.1254	-0.23581
Br	1.408	-0.91814	0.24715
O	-0.61523	-1.35249	0.46049
C	3.37065	-0.42665	0.01734
F	3.98486	-1.60234	0.02986
F	3.86007	0.31012	1.00849
F	3.63749	0.17832	-1.13167
C	-1.43979	-0.35104	0.08379
C	-2.05935	-0.68784	-1.29726
C	-2.53951	-0.20787	1.16699
F	-3.51154	0.64776	0.81604
F	-3.11118	-1.37249	1.4301
F	-1.99632	0.24905	2.29584
F	-2.78986	-1.79322	-1.25432
F	-2.83155	0.29286	-1.78114
F	-1.06962	-0.88041	-2.17463

Sum of electronic and zero-point Energies= -3931.361545
Sum of electronic and thermal Energies= -3931.343012
Sum of electronic and thermal Enthalpies= -3931.342068
Sum of electronic and thermal Free Energies= -3931.410061

2-BrIII-CH3 (in Acetonitrile)

C	0.549	3.23614	-0.25031
C	-0.38449	2.21404	-0.18255
C	0.0167	0.88933	-0.02988
C	1.37278	0.64427	0.0305
C	2.33002	1.63732	-0.03384
C	1.90208	2.95054	-0.17185
H	0.21607	4.25831	-0.36361
H	-1.43776	2.44095	-0.24808
H	3.38423	1.42641	0.02441
H	2.63582	3.74308	-0.21813
Br	1.89375	-1.22511	0.1997
O	-0.27086	-1.42629	0.39484
C	3.84395	-0.94112	0.01061
C	-0.94017	-0.31218	0.07755
C	-1.6345	-0.49397	-1.29956
C	-1.99059	-0.0338	1.18619
F	-2.82761	0.97738	0.89451

F	-2.7362	-1.10268	1.42382
F	-1.36627	0.29091	2.32124
F	-2.51138	-1.48939	-1.28309
F	-2.27866	0.60261	-1.7281
F	-0.70487	-0.78122	-2.21756
H	4.23172	-0.3848	0.85704
H	4.22961	-1.957	0.01821
H	4.06193	-0.45964	-0.93661

Sum of electronic and zero-point Energies= -3633.586431
Sum of electronic and thermal Energies= -3633.569967
Sum of electronic and thermal Enthalpies= -3633.569023
Sum of electronic and thermal Free Energies= -3633.631518

2-BrIII-CHCH2 (in Acetonitrile)

C	0.08632	3.31339	-0.29042
C	-0.7593	2.21802	-0.20455
C	-0.24466	0.93315	-0.054
C	1.12661	0.80805	-0.02632
C	2.00061	1.87287	-0.09451
C	1.4606	3.14495	-0.22517
H	-0.33258	4.30397	-0.39943
H	-1.82916	2.35672	-0.24905
H	3.06878	1.73576	-0.02972
H	2.12197	3.99879	-0.2722
O	-0.30589	-1.37188	0.49808
C	-1.08071	-0.34988	0.11181
C	-1.73839	-0.66823	-1.25785
C	-2.16803	-0.12076	1.19543
C	3.65724	-0.56058	-0.29205
H	3.83266	-0.17175	-1.2861
C	4.60512	-0.87446	0.56869
H	4.37634	-1.27136	1.54993
H	5.64983	-0.74916	0.31197
F	-0.77473	-0.94261	-2.14531
F	-2.45	0.35204	-1.75958
F	-2.54212	-1.72184	-1.1917
F	-2.78688	-1.25072	1.5027
F	-1.60215	0.35217	2.30814
F	-3.1132	0.7625	0.82773
Br	1.80882	-1.01403	0.11217

Sum of electronic and zero-point Energies= -3671.655772
Sum of electronic and thermal Energies= -3671.638328
Sum of electronic and thermal Enthalpies= -3671.637384
Sum of electronic and thermal Free Energies= -3671.702471

2-BrIII-Cl (in Acetonitrile)

C	0.19565	3.33233	0.00417
C	-0.69987	2.27355	0.00322
C	-0.2274	0.96652	0.00062
C	1.13929	0.77717	-0.00062
C	2.05874	1.79991	0.00029
C	1.56176	3.09751	0.00264
H	-0.17885	4.34616	0.00612
H	-1.76371	2.45696	0.00448
H	3.1181	1.60146	-0.00074
H	2.2579	3.92441	0.00331
Br	1.63636	-1.09851	-0.00338
O	-0.29771	-1.40731	-0.00653
Cl	3.92074	-0.71732	-0.00007
C	-1.08888	-0.28359	-0.00098
C	-1.95778	-0.32418	-1.28172
C	-1.95141	-0.33137	1.28378
F	-2.66478	-1.44145	-1.35821
F	-2.80747	0.70523	-1.35174
F	-1.1604	-0.26478	-2.3475
F	-2.79774	0.69984	1.36603
F	-2.66114	-1.44725	1.35567
F	-1.14818	-0.28216	2.34574

Sum of electronic and zero-point Energies= -4053.923432
Sum of electronic and thermal Energies= -4053.907361
Sum of electronic and thermal Enthalpies= -4053.906416
Sum of electronic and thermal Free Energies= -4053.971019

2-BrIII-CN (in Acetonitrile)

C	0.39925	3.28252	0.00748
C	-0.53453	2.25805	0.00566
C	-0.1162	0.93052	0.00095
C	1.23974	0.7	-0.00118
C	2.20193	1.68345	0.0006
C	1.75645	2.99891	0.00485
H	0.06254	4.30954	0.01102
H	-1.59022	2.4829	0.00796
H	3.25716	1.45667	-0.00117
H	2.48402	3.79814	0.00619
Br	1.69086	-1.20567	-0.00632
O	-0.30223	-1.43412	-0.01188
C	3.61486	-0.82112	-0.00143
N	4.75728	-0.70051	0.00103
C	-1.03612	-0.28825	-0.00195
C	-1.91129	-0.26918	-1.27973
C	-1.89994	-0.28278	1.28365
F	-2.70787	0.80407	-1.34463
F	-2.67605	-1.34683	-1.36356
F	-1.11457	-0.2427	-2.34914
F	-2.69008	0.79351	1.37107
F	-2.66953	-1.35765	1.35939
F	-1.09321	-0.2754	2.34595

Sum of electronic and zero-point Energies= -3686.522957
Sum of electronic and thermal Energies= -3686.506166
Sum of electronic and thermal Enthalpies= -3686.505222
Sum of electronic and thermal Free Energies= -3686.568808

2-BrIII-F (in Acetonitrile)

C	0.83683	3.21127	0.00239
C	-0.19559	2.28461	0.00173
C	0.10234	0.92744	0.00005
C	1.42895	0.55872	-0.00068
C	2.48292	1.44535	0.00003
C	2.16118	2.79672	0.00152
H	0.60288	4.26646	0.00366
H	-1.22484	2.61046	0.00259
H	3.50157	1.09403	-0.00049
H	2.9588	3.52634	0.00204
Br	1.68141	-1.34159	-0.00228
O	-0.2387	-1.42383	-0.00453
F	3.5185	-1.08283	-0.00036
C	-0.89892	-0.21442	-0.00069
C	-1.76549	-0.15669	-1.28107
C	-1.76104	-0.16154	1.28288
F	-2.60447	-1.17913	-1.35372
F	-2.48148	0.9692	-1.35442
F	-0.96697	-0.20015	-2.34673
F	-2.47469	0.96525	1.3642
F	-2.60153	-1.1829	1.35343
F	-0.95882	-0.21163	2.34551

Sum of electronic and zero-point Energies= -3693.557301
Sum of electronic and thermal Energies= -3693.541789
Sum of electronic and thermal Enthalpies= -3693.540845
Sum of electronic and thermal Free Energies= -3693.601738

2-BrIII-N3 (in Acetonitrile)

C	0.04576	3.31083	0.46151
C	-0.82888	2.23516	0.41898
C	-0.36214	0.97808	0.05365
C	0.97866	0.85162	-0.2355
C	1.87808	1.89336	-0.20801
C	1.38578	3.14351	0.14505
H	-0.32437	4.28697	0.74136
H	-1.87151	2.36796	0.66698
H	2.91488	1.7442	-0.4647
H	2.06162	3.98676	0.16996
Br	1.50431	-0.96165	-0.68399
O	-0.45773	-1.27825	-0.65781
C	-1.19399	-0.29208	-0.05925
C	-2.43477	-0.04858	-0.95179
C	-1.60256	-0.75832	1.36007
F	-3.07112	-1.1784	-1.21759
F	-3.31048	0.78869	-0.38118
F	-2.05359	0.4926	-2.10699
F	-2.2572	0.18657	2.04091
F	-2.36621	-1.84103	1.32585
F	-0.49623	-1.05424	2.04706
N	3.45501	-0.50686	-0.75303
N	3.94959	-0.62503	0.36467
N	4.45655	-0.71544	1.36091

Sum of electronic and zero-point Energies= -3757.878638
Sum of electronic and thermal Energies= -3757.861115
Sum of electronic and thermal Enthalpies= -3757.860171
Sum of electronic and thermal Free Energies= -3757.926024

2-BrIII-NH2 (in Acetonitrile)

C	0.61888	3.24878	0.11075
C	-0.34353	2.25091	0.07965
C	0.03302	0.91338	0.01116
C	1.38138	0.62614	-0.0161
C	2.36224	1.59406	0.01672
C	1.96565	2.92348	0.07827
H	0.31344	4.28439	0.16136
H	-1.39182	2.5072	0.10923
H	3.41011	1.34147	-0.00505
H	2.7176	3.69983	0.10163
Br	1.83973	-1.26624	-0.09117
O	-0.2267	-1.42859	-0.18334
C	-0.92239	-0.2801	-0.03366
C	-1.88003	-0.13065	-1.24231
C	-1.70991	-0.34006	1.2999
F	-0.84474	-0.48677	2.30704
F	-2.55053	-1.36478	1.33112
F	-2.41739	0.77172	1.54549
F	-2.65123	-1.19769	-1.39013
F	-2.68502	0.93853	-1.1464
F	-1.16183	0.01477	-2.35774
N	3.71808	-1.10497	-0.0212
H	4.02739	-0.6583	0.83388
H	4.08471	-0.61028	-0.82554

Sum of electronic and zero-point Energies= -3649.633771

Sum of electronic and thermal Energies= -3649.617649
Sum of electronic and thermal Enthalpies= -3649.616705
Sum of electronic and thermal Free Energies= -3649.678692

2-BrIII-NHAc (in Acetonitrile)

C	-0.34726	3.33203	0.34911
C	-1.19704	2.24846	0.18993
C	-0.67438	0.96335	0.09522
C	0.69215	0.81966	0.19026
C	1.56845	1.86961	0.34255
C	1.02534	3.14515	0.41857
H	-0.76046	4.3288	0.41313
H	-2.26542	2.39551	0.13615
H	2.63241	1.71139	0.38974
H	1.6869	3.99311	0.52725
Br	1.29995	-1.02104	0.08299
O	-0.63675	-1.35055	-0.38345
C	4.04659	-0.30359	-0.36739
O	3.72955	0.08302	-1.46771
C	5.48471	-0.47455	0.06868
H	5.6485	-0.08119	1.07115
H	5.7312	-1.5368	0.07551
H	6.12837	0.03482	-0.64128
C	-1.4778	-0.31731	-0.10715
C	-2.44136	-0.16468	-1.30916
C	-2.25723	-0.62958	1.19514
F	-3.01294	-1.3182	-1.62158
F	-3.42098	0.71993	-1.07096
F	-1.7662	0.26229	-2.37394
F	-3.04244	0.37959	1.58678
F	-3.0159	-1.7107	1.07455
F	-1.37859	-0.85275	2.17815
N	3.10736	-0.60389	0.58757
H	3.40648	-0.99461	1.46863

Sum of electronic and zero-point Energies= -3802.261394
Sum of electronic and thermal Energies= -3802.241616
Sum of electronic and thermal Enthalpies= -3802.240672
Sum of electronic and thermal Free Energies= -3802.311911

2-BrIII-OCOCH3 (in Acetonitrile)

C	-0.85473	3.41472	0.00147
C	-1.53185	2.20509	0.00111
C	-0.81706	1.01329	0.00004
C	0.56125	1.0802	-0.00047
C	1.26756	2.26562	-0.00011
C	0.53151	3.44327	0.00083
H	-1.41506	4.33894	0.00226
H	-2.61108	2.18285	0.00168
H	2.34335	2.27341	-0.0005
H	1.05628	4.38841	0.00108
Br	1.39201	-0.65847	-0.00148
O	-0.43038	-1.3286	-0.00333
C	4.12689	-0.63449	-0.00053
O	3.94053	-1.82591	-0.00192
O	3.14066	0.25571	0.00012
C	5.48582	0.01505	0.00106
H	5.58621	0.65234	-0.87609
H	5.5874	0.6443	0.88391
H	6.2516	-0.75352	-0.00275
C	-1.42375	-0.37925	-0.00049
C	-2.26756	-0.58778	-1.28126
C	-2.26336	-0.59025	1.28264
F	-2.74982	-1.81897	-1.35607
F	-3.29743	0.26172	-1.35504
F	-1.49524	-0.38027	-2.34723
F	-3.29196	0.26023	1.36229
F	-2.74673	-1.82109	1.35599
F	-1.48702	-0.38636	2.34643

Sum of electronic and zero-point Energies= -3822.144474
Sum of electronic and thermal Energies= -3822.126577
Sum of electronic and thermal Enthalpies= -3822.125633
Sum of electronic and thermal Free Energies= -3822.191587

2-BrIII-OH (in Acetonitrile)

C	0.73193	3.22992	0.26144
C	-0.2697	2.27223	0.19727
C	0.06455	0.93331	0.03287
C	1.40113	0.6087	-0.0405
C	2.42417	1.52901	0.01703
C	2.06657	2.863	0.16736
H	0.46607	4.27053	0.38324
H	-1.30749	2.56075	0.27383
H	3.45247	1.21539	-0.06311
H	2.84213	3.61502	0.20882
Br	1.74613	-1.28164	-0.23144
O	-0.23393	-1.36666	-0.43173
O	3.60092	-1.04618	-0.08509
H	3.82836	-1.18777	0.83999
C	-0.90761	-0.23494	-0.07091
C	-1.96619	0.03922	-1.16542
C	-1.57229	-0.4627	1.30928
F	-2.7167	-1.02758	-1.39502
F	-2.78442	1.04922	-0.83992
F	-1.35678	0.36683	-2.30338
F	-2.19079	0.6288	1.77075
F	-2.45699	-1.44972	1.27907
F	-0.62404	-0.78756	2.19313

Sum of electronic and zero-point Energies= -3669.518032
Sum of electronic and thermal Energies= -3669.502134
Sum of electronic and thermal Enthalpies= -3669.501190
Sum of electronic and thermal Free Energies= -3669.562321

2-BrIII-OTs (in Acetonitrile)

C	-2.91488	3.10695	-0.55244
C	-3.32957	1.78585	-0.47485
C	-2.43649	0.81295	-0.04513
C	-1.15524	1.20849	0.27296
C	-0.70251	2.50589	0.21088
C	-1.61868	3.46251	-0.20953
H	-3.61113	3.86381	-0.8848
H	-4.33735	1.50925	-0.74713
H	0.30834	2.77807	0.47119
H	-1.30172	4.49394	-0.27116
Br	-0.05575	-0.28981	0.79985
O	-1.68357	-1.27841	0.77777
C	-2.72634	-0.66798	0.10387
C	-2.85939	-1.31834	-1.29497
C	-3.98885	-0.90206	0.96589
F	-3.15996	-2.60778	-1.21272
F	-3.79319	-0.72237	-2.03753
F	-1.69514	-1.20336	-1.93101
F	-5.09704	-0.50212	0.33483
F	-4.13553	-2.18042	1.27725
F	-3.89084	-0.21028	2.09783
O	1.55828	0.87272	0.91137
S	2.3618	1.23496	-0.37456
O	2.71171	2.61846	-0.27414
O	1.62982	0.77158	-1.51734
C	3.82603	0.27074	-0.21181
C	4.89232	0.76915	0.52238
C	3.88864	-0.97721	-0.81216
C	6.03111	-0.00549	0.66247
H	4.82686	1.75568	0.95997
C	5.03704	-1.73874	-0.66203
H	3.05556	-1.33027	-1.40472
C	6.1187	-1.26864	0.07878
H	6.87129	0.37838	1.22732
H	5.09863	-2.70989	-1.13651
C	7.34964	-2.1106	0.26148
H	7.46842	-2.81905	-0.5564
H	8.24434	-1.49241	0.3172
H	7.2853	-2.68199	1.18938

Sum of electronic and zero-point Energies= -4488.399202
Sum of electronic and thermal Energies= -4488.373710
Sum of electronic and thermal Enthalpies= -4488.372766
Sum of electronic and thermal Free Energies= -4488.458326

2-ClIII-anion (in Acetonitrile)

C	2.07962	2.24004	0.7755
C	0.85523	1.6475	0.5137
C	0.73249	0.36697	-0.03269
C	1.92676	-0.31745	-0.27457
C	3.16407	0.26744	-0.01411
C	3.24837	1.54685	0.50262
H	2.11574	3.23888	1.19112
H	-0.04074	2.2033	0.74452
H	4.0566	-0.30668	-0.22098
H	4.21716	1.98986	0.69509
O	-0.6671	-1.08459	-1.39127
C	-0.66549	-0.26677	-0.38427
C	-1.69475	0.89709	-0.68131
C	-1.15071	-0.95901	0.94192
Cl	2.00753	-1.94743	-0.86909
F	-0.41496	-2.0458	1.19589
F	-1.06758	-0.1876	2.05304
F	-2.42722	-1.36016	0.86468
F	-2.81495	0.41482	-1.21341
F	-2.09935	1.64643	0.38231
F	-1.18854	1.76805	-1.56983

Sum of electronic and zero-point Energies= -1479.937180
Sum of electronic and thermal Energies= -1479.922807
Sum of electronic and thermal Enthalpies= -1479.921863
Sum of electronic and thermal Free Energies= -1479.979234

2-ClIII-Br (in Acetonitrile)

C	-0.17091	3.27513	0.4348
C	-1.03027	2.19187	0.32927
C	-0.5184	0.93026	0.06389
C	0.84973	0.79317	-0.06386
C	1.7317	1.84349	0.03166
C	1.19508	3.10133	0.27987
H	-0.57232	4.25827	0.6355
H	-2.09608	2.32034	0.44802
H	2.79375	1.69938	-0.07747
H	1.86459	3.94659	0.35364
O	-0.4787	-1.30897	-0.62406

C	-1.31263	-0.34636	-0.11244
C	-2.45328	-0.15051	-1.14085
C	-1.85716	-0.81266	1.26005
F	-3.01866	-1.30317	-1.46152
F	-3.40967	0.66031	-0.67338
F	-1.96527	0.39505	-2.25129
F	-2.59602	0.12736	1.85451
F	-2.59363	-1.90923	1.15396
F	-0.82479	-1.08133	2.06051
Br	3.71005	-0.66427	-0.08389
Cl	1.35631	-0.90933	-0.35709

Sum of electronic and zero-point Energies= -4053.898840
Sum of electronic and thermal Energies= -4053.882902
Sum of electronic and thermal Enthalpies= -4053.881958
Sum of electronic and thermal Free Energies= -4053.943931

2-ClIII-CCH (in Acetonitrile)

C	0.89798	3.05126	0.20313
C	-0.12328	2.11575	0.15029
C	0.16259	0.75866	0.02529
C	1.49212	0.41205	-0.0308
C	2.54007	1.30152	0.0192
C	2.22223	2.64797	0.1359
H	0.65651	4.10066	0.29725
H	-1.15408	2.43139	0.20549
H	3.56665	0.97575	-0.03013
H	3.02148	3.37451	0.17472
O	-0.23552	-1.53802	-0.26677
C	-0.86604	-0.37505	-0.05713
C	-1.83139	-0.09974	-1.2396
C	-1.63753	-0.43389	1.28782
C	4.7137	-1.50763	-0.0788
H	5.76679	-1.66024	-0.05136
C	3.5312	-1.32388	-0.10729
F	-0.76954	-0.67209	2.27519
F	-2.5404	-1.40426	1.29422
F	-2.27089	0.71016	1.58982
F	-2.67427	-1.10511	-1.42242
F	-2.56357	1.01527	-1.08111
F	-1.12316	0.05294	-2.36077
Cl	1.81726	-1.38661	-0.17436

Sum of electronic and zero-point Energies= -1556.402041
Sum of electronic and thermal Energies= -1556.385275
Sum of electronic and thermal Enthalpies= -1556.384331
Sum of electronic and thermal Free Energies= -1556.447786

2-ClIII-CF3 (in Acetonitrile)

C	-0.09422	3.22786	0.5386
C	-0.94052	2.13908	0.40925
C	-0.43474	0.88533	0.08268
C	0.92975	0.77437	-0.07094
C	1.80609	1.83499	0.03649
C	1.2689	3.07768	0.34271
H	-0.50239	4.19803	0.78421
H	-2.0043	2.25485	0.55351
H	2.8667	1.74425	-0.11462
H	1.93332	3.92603	0.42565
O	-0.49094	-1.31384	-0.73116
C	3.3307	-0.68958	-0.12769
F	3.79046	-1.92294	-0.2031
F	3.59982	-0.19387	1.06377
F	3.92054	0.04392	-1.05679
C	-1.26555	-0.37964	-0.15733
C	-2.44659	-0.06118	-1.11103
C	-1.78834	-0.88173	1.21462
F	-2.46828	0.05212	1.89391
F	-2.57377	-1.94223	1.08906
F	-0.74131	-1.23056	1.97058
F	-3.06083	-1.16763	-1.49831
F	-3.37239	0.73881	-0.5558
F	-1.99071	0.56013	-2.19977
Cl	1.50055	-0.91855	-0.42798

Sum of electronic and zero-point Energies= -1817.350998
Sum of electronic and thermal Energies= -1817.332847
Sum of electronic and thermal Enthalpies= -1817.331902
Sum of electronic and thermal Free Energies= -1817.398322

2-ClIII-CH3 (in Acetonitrile)

C	1.6293	2.66398	-0.71181
C	0.49052	1.89921	-0.51699
C	0.54288	0.5862	-0.03705
C	1.82328	0.12188	0.21544
C	2.9889	0.84851	0.02854
C	2.88643	2.1446	-0.44171
H	1.53044	3.6756	-1.0809
H	-0.4733	2.32812	-0.74032
H	3.94911	0.40629	0.2522
H	3.77865	2.73511	-0.59329
O	-0.48688	-1.48141	0.69689
C	1.74454	-2.70582	-0.39167
C	-0.73172	-0.30195	0.18397
C	-1.39532	-0.46908	-1.22251
C	-1.68989	0.49153	1.12876
F	-2.15727	1.65113	0.62054
F	-2.74689	-0.23561	1.45613
F	-1.04102	0.80411	2.25684
F	-2.50975	-1.18293	-1.14985
F	-1.698	0.6706	-1.86849
F	-0.53954	-1.13583	-2.02174
H	2.15437	-2.30615	-1.31269
H	2.25258	-3.60925	-0.06811
H	0.65667	-2.73225	-0.35136
Cl	2.14972	-1.49788	0.89815

Sum of electronic and zero-point Energies= -1519.578891
Sum of electronic and thermal Energies= -1519.562365
Sum of electronic and thermal Enthalpies= -1519.561421
Sum of electronic and thermal Free Energies= -1519.623890

2-ClIII-CHCH2 (in Acetonitrile)

C	0.64646	3.11205	-0.50256
C	-0.30757	2.11418	-0.37034
C	0.06277	0.80422	-0.08213
C	1.41428	0.55403	0.02237
C	2.39514	1.51754	-0.08481
C	1.99193	2.81963	-0.34758
H	0.33447	4.12493	-0.71556
H	-1.35565	2.34978	-0.48114
H	3.43999	1.28483	0.04847
H	2.73784	3.59787	-0.42667
O	-0.23433	-1.36839	0.77686
C	-0.89274	-0.37699	0.1824
C	-1.45687	-0.83082	-1.19332
C	-2.05229	0.0929	1.10389
C	3.6001	-1.07259	-0.1654
H	3.7589	-0.77499	-1.19203
C	4.52003	-1.49905	0.67405
H	4.27261	-1.79136	1.68618
H	5.55195	-1.57701	0.35901
F	-0.44997	-1.31313	-1.93522
F	-2.01252	0.16508	-1.90201
F	-2.36537	-1.78978	-1.07086
F	-2.7823	-0.93257	1.51456
F	-1.55518	0.70127	2.18405
F	-2.89279	0.96609	0.5157
Cl	1.89001	-1.16766	0.31361

Sum of electronic and zero-point Energies= -1557.651468
Sum of electronic and thermal Energies= -1557.634305
Sum of electronic and thermal Enthalpies= -1557.633361
Sum of electronic and thermal Free Energies= -1557.697230

2-ClIII-Cl (in Acetonitrile)

C	0.76403	3.11418	0.42585
C	-0.25207	2.1755	0.32717
C	0.06164	0.85084	0.06101
C	1.391	0.50744	-0.07594
C	2.42546	1.40889	0.01388
C	2.08687	2.73357	0.26387
H	0.51929	4.14726	0.62786
H	-1.28504	2.46474	0.45298
H	3.45111	1.10158	-0.10069
H	2.87741	3.46727	0.33419
O	-0.22445	-1.37732	-0.59703
Cl	3.83531	-1.28893	-0.15298
C	-0.91296	-0.29378	-0.10476
C	-2.004	0.05382	-1.14562
C	-1.52791	-0.65886	1.26831
F	-2.73776	-1.00449	-1.45064
F	-2.82598	1.01066	-0.70024
F	-1.43144	0.4969	-2.26123
F	-2.12785	0.3861	1.84353
F	-2.41377	-1.63934	1.16962
F	-0.55042	-1.06228	2.08044
Cl	1.62501	-1.25224	-0.36901

Sum of electronic and zero-point Energies= -1939.896167
Sum of electronic and thermal Energies= -1939.880423
Sum of electronic and thermal Enthalpies= -1939.879479
Sum of electronic and thermal Free Energies= -1939.940555

2-ClIII-CN (in Acetonitrile)

C	0.99397	3.02543	0.23427
C	-0.05881	2.12568	0.17695
C	0.18947	0.76453	0.03108
C	1.50493	0.37503	-0.03886
C	2.58354	1.22656	0.01384
C	2.30411	2.58036	0.15057
H	0.78872	4.08079	0.34465
H	-1.07936	2.47128	0.24479
H	3.60312	0.87922	-0.04669
H	3.12626	3.28051	0.1921
O	-0.23956	-1.51624	-0.30642
C	3.49406	-1.39063	-0.12114
N	4.63424	-1.51659	-0.08274
C	-0.85917	-0.33981	-0.06104
C	-1.82876	-0.04052	-1.23141
C	-1.61238	-0.42923	1.29024
F	-2.54463	1.07612	-1.0383

F	-2.6793	-1.03631	-1.42318
F	-1.12619	0.12589	-2.3523
F	-2.21668	0.71782	1.62522
F	-2.53046	-1.38357	1.27883
F	-0.73326	-0.71115	2.25447
Cl	1.72041	-1.4382	-0.20435

Sum of electronic and zero-point Energies= -1572.502365
Sum of electronic and thermal Energies= -1572.486801
Sum of electronic and thermal Enthalpies= -1572.485857
Sum of electronic and thermal Free Energies= -1572.545375

2-ClIII-F (in Acetonitrile)

C	1.66678	2.75385	-0.00132
C	0.45839	2.073	-0.00109
C	0.46169	0.68545	-0.00028
C	1.67237	0.03144	0.00026
C	2.89293	0.66956	0.00006
C	2.86767	2.0588	-0.00075
H	1.66942	3.8346	-0.00195
H	-0.47903	2.60891	-0.00154
H	3.81283	0.11103	0.00047
H	3.80524	2.59674	-0.00095
O	-0.28432	-1.5354	0.00158
F	3.25126	-1.94181	0.00064
C	-0.73766	-0.23291	0.00024
C	-1.57554	-0.02467	-1.28278
C	-1.57631	-0.02237	1.28239
F	-2.58747	-0.8763	-1.35353
F	-2.07041	1.21412	-1.35778
F	-0.79713	-0.21695	-2.34556
F	-2.07237	1.21614	1.35418
F	-2.58751	-0.87469	1.35472
F	-0.79835	-0.21141	2.34608
Cl	1.50834	-1.74134	0.00131

Sum of electronic and zero-point Energies= -1579.529953
Sum of electronic and thermal Energies= -1579.515718
Sum of electronic and thermal Enthalpies= -1579.514774
Sum of electronic and thermal Free Energies= -1579.571214

2-ClIII-N3 (in Acetonitrile)

C	-0.48604	3.08421	0.84546
C	0.46464	2.08482	0.69994
C	0.11092	0.87731	0.11383
C	-1.19749	0.70777	-0.28271
C	-2.16849	1.67636	-0.1614
C	-1.78731	2.8838	0.41081
H	-0.20629	4.02576	1.29634
H	1.48002	2.23791	1.03481
H	-3.17488	1.50891	-0.50756
H	-2.52349	3.66853	0.51337
O	0.35561	-1.17894	-0.97417
C	1.01996	-0.30689	-0.16064
C	1.36613	-1.00486	1.17766
C	2.2965	0.14701	-0.90789
N	-4.36112	-1.41129	0.96819
N	-3.8816	-1.07624	0.0123
N	-3.41081	-0.6967	-1.05928
Cl	-1.54216	-0.91837	-0.97628
F	3.00689	-0.8882	-1.32705
F	1.95856	0.87418	-1.97032
F	3.08987	0.90202	-0.13612
F	1.91973	-0.16857	2.06118
F	2.19287	-2.02641	1.00687
F	0.23857	-1.47133	1.72036

Sum of electronic and zero-point Energies= -1643.855459
Sum of electronic and thermal Energies= -1643.838121
Sum of electronic and thermal Enthalpies= -1643.837177
Sum of electronic and thermal Free Energies= -1643.903670

2-ClIII-NH2 (in Acetonitrile)

C	1.42954	2.84602	0.32072
C	0.29389	2.0553	0.2397
C	0.40447	0.68396	0.03796
C	1.67455	0.15565	-0.06006
C	2.8271	0.90594	0.01892
C	2.68689	2.27481	0.2078
H	1.32981	3.91158	0.47279
H	-0.68678	2.49747	0.33142
H	3.79522	0.44218	-0.0594
H	3.57465	2.88869	0.26827
O	-0.26601	-1.51068	-0.42752
C	-0.75317	-0.30291	-0.08762
C	-1.72869	0.17081	-1.19451
C	-1.47376	-0.39699	1.28223
F	-0.60856	-0.84718	2.19629
F	-2.50068	-1.23612	1.24579
F	-1.93128	0.78388	1.72282
F	-2.66773	-0.7329	-1.43506
F	-2.34857	1.3238	-0.89437
F	-1.05281	0.37089	-2.32747
N	3.51527	-1.77578	-0.19395
H	3.79213	-2.2539	-1.04467
H	3.69627	-2.39013	0.59271

Cl	1.75539	-1.641	-0.29095

Sum of electronic and zero-point Energies= -1535.618126
Sum of electronic and thermal Energies= -1535.602179
Sum of electronic and thermal Enthalpies= -1535.601234
Sum of electronic and thermal Free Energies= -1535.662129

2-ClIII-NHAc (in Acetonitrile)

C	-0.11551	3.23975	0.37508
C	-0.99039	2.17573	0.22135
C	-0.49734	0.88177	0.10817
C	0.86612	0.70408	0.18585
C	1.76774	1.73375	0.32872
C	1.25277	3.02017	0.4193
H	-0.50432	4.24532	0.45225
H	-2.05665	2.34254	0.18203
H	2.82862	1.55844	0.35921
H	1.93641	3.85124	0.52053
O	-0.45781	-1.38949	-0.43294
C	4.05642	-0.5816	-0.36112
O	3.79331	-0.16449	-1.46085
C	5.45226	-0.93701	0.09317
H	5.64208	-0.60177	1.11181
H	5.56883	-2.02097	0.06203
H	6.16586	-0.48485	-0.58816
C	-1.30954	-0.38663	-0.11232
C	-2.30152	-0.19664	-1.28531
C	-2.06098	-0.73166	1.19845
F	-2.87873	-1.34084	-1.6208
F	-3.27775	0.6784	-0.99598
F	-1.65463	0.26714	-2.35237
F	-2.83038	0.27138	1.6381
F	-2.82952	-1.80457	1.06696
F	-1.16156	-0.98666	2.15532
N	3.07757	-0.74391	0.59825
H	3.2939	-1.23851	1.45175
Cl	1.40418	-1.018	0.08012

Sum of electronic and zero-point Energies= -1688.242619
Sum of electronic and thermal Energies= -1688.223123
Sum of electronic and thermal Enthalpies= -1688.222179
Sum of electronic and thermal Free Energies= -1688.292252

2-ClIII-OCOCH3 (in Acetonitrile)

C	-0.60132	3.37029	0.00316
C	-1.31001	2.17922	0.00251
C	-0.62326	0.97315	0.00028
C	0.75576	0.99643	-0.00091
C	1.49279	2.1631	-0.00034
C	0.78488	3.35824	0.00165
H	-2.38966	2.17972	0.00373
H	2.56717	2.14787	-0.00134
H	1.33632	4.28799	0.00207
O	-0.22459	-1.33625	-0.00598
C	4.09696	-0.86534	-0.00147
O	3.81964	-2.03617	-0.00416
O	3.18723	0.10892	-0.00009
C	5.5014	-0.31896	0.00094
H	5.65176	0.30708	-0.87717
H	5.65085	0.30107	0.88347
H	6.20507	-1.14499	-0.00145
C	-1.23819	-0.40794	-0.00098
C	-2.08079	-0.61817	-1.28163
C	-2.07392	-0.62309	1.28335
F	-2.56223	-1.84946	-1.35622
F	-3.11023	0.23233	-1.35065
F	-1.31048	-0.40852	-2.34755
F	-3.10145	0.22881	1.36242
F	-2.557	-1.85393	1.35479
F	-1.29709	-0.41985	2.34583
Cl	1.47504	-0.6404	-0.00289
H	-1.13484	4.31017	0.00485

Sum of electronic and zero-point Energies= -1708.116855
Sum of electronic and thermal Energies= -1708.098506
Sum of electronic and thermal Enthalpies= -1708.097562
Sum of electronic and thermal Free Energies= -1708.164392

2-ClIII-OH (in Acetonitrile)

C	1.56689	2.77117	0.47725
C	0.38692	2.04915	0.37514
C	0.43268	0.69623	0.06766
C	1.66684	0.10896	-0.1025
C	2.85972	0.79215	-0.01355
C	2.79007	2.14897	0.27788
H	1.52899	3.82606	0.70967
H	-0.5676	2.5302	0.52971
H	3.79952	0.2916	-0.17282
H	3.70723	2.71697	0.3469
O	-0.27575	-1.41713	-0.62693
O	3.34242	-1.87635	-0.34211
H	3.50414	-2.18214	0.558
C	-0.74438	-0.24473	-0.11498
C	-1.75907	0.34221	-1.12387
C	-1.40549	-0.50596	1.26058
F	-2.70006	-0.53628	-1.43511

F	-2.36652	1.43967	-0.65123
F	-1.12796	0.68511	-2.24501
F	-1.7964	0.62112	1.86514
F	-2.4598	-1.30388	1.16441
F	-0.51327	-1.09757	2.0604
Cl	1.61207	-1.65043	-0.45107

Sum of electronic and zero-point Energies= -1555.494071
Sum of electronic and thermal Energies= -1555.478534
Sum of electronic and thermal Enthalpies= -1555.477589
Sum of electronic and thermal Free Energies= -1555.537513

2-ClIII-OTs (in Acetonitrile)

C	2.96674	3.03729	0.56518
C	3.32387	1.69964	0.48697
C	2.40138	0.78129	0.00909
C	1.15195	1.23219	-0.35293
C	0.75715	2.5481	-0.29401
C	1.70267	3.45332	0.17357
H	3.68127	3.7591	0.93436
H	4.3057	1.36812	0.79158
H	-0.22914	2.86933	-0.58633
H	1.43076	4.49749	0.23482
O	1.52681	-1.18018	-0.91718
C	2.59819	-0.7029	-0.17502
C	2.62148	-1.41627	1.19814
C	3.86605	-1.0051	-1.00572
F	2.8306	-2.7203	1.07298
F	3.56769	-0.91792	1.99445
F	1.44545	-1.23797	1.79212
F	4.97417	-0.71333	-0.31946
F	3.92393	-2.27935	-1.35961
F	3.86241	-0.26859	-2.11213
O	-1.44984	1.00745	-0.98595
S	-2.24445	1.28589	0.32212
O	-2.65276	2.65702	0.28509
O	-1.4752	0.80883	1.43541
C	-3.67377	0.27091	0.15116
C	-4.78502	0.76341	-0.51785
C	-3.66374	-1.01101	0.67781
C	-5.89464	-0.05043	-0.66633
H	-4.77529	1.77527	-0.89873
C	-4.78395	-1.81255	0.51984
H	-2.79623	-1.36156	1.22048
C	-5.90922	-1.34816	-0.15587
H	-6.76935	0.32809	-1.1803
H	-4.78778	-2.81169	0.9366
C	-7.11273	-2.22721	-0.34733
H	-7.1199	-3.04888	0.36637
H	-8.0352	-1.65975	-0.23087
H	-7.11573	-2.65616	-1.35102
Cl	0.0996	-0.11862	-0.89089

Sum of electronic and zero-point Energies= -2374.373230
Sum of electronic and thermal Energies= -2374.347979
Sum of electronic and thermal Enthalpies= -2374.347035
Sum of electronic and thermal Free Energies= -2374.432378

2-IIII-anion (in Acetonitrile)

C	-0.07544	3.3029	0.41141
C	-0.79322	2.13104	0.24791
C	-0.17712	0.89529	0.02306
C	1.21486	0.88327	0.00121
C	1.94821	2.05692	0.17031
C	1.30977	3.26839	0.36678
H	-0.59846	4.23664	0.57423
H	-1.87082	2.17356	0.29853
H	3.02819	2.01012	0.14896
H	1.8933	4.17206	0.48939
O	-0.40144	-1.38937	-0.78021
C	-1.02683	-0.411	-0.1974
C	-2.30129	-0.04156	-1.05183
C	-1.52987	-0.83432	1.22809
F	-0.50143	-1.26954	1.96306
F	-2.11675	0.14656	1.95519
F	-2.42186	-1.83146	1.16673
F	-2.91139	-1.13771	-1.49511
F	-3.27006	0.67596	-0.41996
F	-1.95803	0.67709	-2.13204
I	2.41435	-0.83644	-0.22279

Sum of electronic and zero-point Energies= -1317.366499
Sum of electronic and thermal Energies= -1317.351699
Sum of electronic and thermal Enthalpies= -1317.350754
Sum of electronic and thermal Free Energies= -1317.410236

2-IIII-Br (in Acetonitrile)

C	-0.78138	3.45879	-0.00672
C	-1.5331	2.29365	-0.00499
C	-0.90049	1.05424	-0.00004
C	0.48229	1.03851	0.0021
C	1.25712	2.17753	0.00032
C	0.60372	3.40291	-0.00389
H	-1.28437	4.41552	-0.01045
H	-2.61116	2.3457	-0.00771
H	2.335	2.11992	0.00205
H	1.18789	4.3125	-0.00515
O	-0.74956	-1.34046	0.01536
C	-1.62983	-0.28612	0.00209
C	-2.47912	-0.42125	-1.28558
C	-2.49856	-0.40864	1.27807
F	-3.0762	-1.60177	-1.35704
F	-3.42261	0.52039	-1.38028
F	-1.67801	-0.29935	-2.34467
F	-3.45103	0.52661	1.34334
F	-3.08766	-1.5926	1.35726
F	-1.71627	-0.26476	2.34818
Br	3.75346	-0.10036	-0.00157
I	1.27849	-0.91343	0.00687

Sum of electronic and zero-point Energies= -3891.405857
Sum of electronic and thermal Energies= -3891.389437
Sum of electronic and thermal Enthalpies= -3891.388493
Sum of electronic and thermal Free Energies= -3891.453116

2-IIII-CCH (in Acetonitrile)

C	-0.21743	3.43619	-0.00012
C	-1.01586	2.30285	-0.00009
C	-0.43651	1.03626	-0.00001
C	0.94106	0.96409	0.00002
C	1.7634	2.07151	-0.00002
C	1.16487	3.32361	-0.00009
H	-0.68058	4.41299	-0.00018
H	-2.09074	2.40014	-0.00012
H	2.83834	1.96729	0.00001
H	1.78608	4.20847	-0.00011
O	-0.39748	-1.35666	0.00026
C	-1.22572	-0.27947	0.00004
C	-2.09509	-0.35419	-1.27975
C	-2.09539	-0.35394	1.27964
C	4.84815	-0.01601	-0.00007
H	5.88595	0.22225	-0.00012
C	3.67518	-0.28105	-0.00001
F	-1.30272	-0.25532	2.34912
F	-2.74916	-1.50421	1.3616
F	-2.99883	0.63097	1.35901
F	-2.74897	-1.5044	-1.36156
F	-2.99838	0.63081	-1.35962
F	-1.30213	-0.25595	-2.34906
I	1.70759	-1.00253	0.00011

Sum of electronic and zero-point Energies= -1393.891723
Sum of electronic and thermal Energies= -1393.874488
Sum of electronic and thermal Enthalpies= -1393.873544
Sum of electronic and thermal Free Energies= -1393.939054

2-IIII-CF3 (in Acetonitrile)

C	-0.75458	3.4406	0.00137
C	-1.5013	2.27365	0.00109
C	-0.86705	1.03366	0.00007
C	0.51295	1.01556	-0.00043
C	1.2839	2.16282	-0.00019
C	0.63065	3.38684	0.00067
H	-1.25937	4.39642	0.00214
H	-2.57927	2.3232	0.00168
H	2.36083	2.1344	-0.00068
H	1.21469	4.29652	0.00083
O	-0.74343	-1.35841	-0.00269
C	3.42662	-0.14875	0.00017
F	4.19302	-1.24446	0.00031
F	3.75004	0.57005	1.07722
F	3.7516	0.5708	-1.07589
C	-1.60871	-0.30907	-0.0004
C	-2.47537	-0.41562	-1.28003
C	-2.47207	-0.41799	1.28123
F	-3.41078	0.53231	1.36343
F	-3.0815	-1.59191	1.36165
F	-1.68014	-0.29148	2.34817
F	-3.08355	-1.59008	-1.36185
F	-3.41554	0.53367	-1.35718
F	-1.68657	-0.28532	-2.3488
I	1.3421	-0.92917	-0.00127

Sum of electronic and zero-point Energies= -1654.831381
Sum of electronic and thermal Energies= -1654.813395
Sum of electronic and thermal Enthalpies= -1654.812451
Sum of electronic and thermal Free Energies= -1654.878761

2-IIII-CH3 (in Acetonitrile)

C	-0.17485	3.42882	0.00033
C	-0.94484	2.27629	0.00025
C	-0.34045	1.02121	-0.00001
C	1.0391	0.97044	-0.00011
C	1.82972	2.10486	-0.00001
C	1.2086	3.34571	0.00019
H	-0.66002	4.39489	0.00052
H	-2.02146	2.35019	0.00039
H	2.90617	2.04596	-0.00009
H	1.81232	4.2427	0.00026
O	-0.2804	-1.37037	-0.00073
C	3.91254	-0.19883	0.00026
C	-1.11386	-0.30963	-0.00012

C	-1.98781	-0.38291	-1.27849
C	-1.98689	-0.38353	1.27882
F	-2.90259	0.59297	1.35894
F	-2.63172	-1.53901	1.36598
F	-1.19831	-0.27296	2.35145
F	-2.63223	-1.53858	-1.36602
F	-2.904	0.59327	-1.35717
F	-1.20011	-0.27119	-2.35164
H	4.10973	0.37774	0.89847
H	4.52341	-1.09934	0.00056
H	4.11036	0.37756	-0.89793
I	1.88747	-0.96306	-0.00032

Sum of electronic and zero-point Energies= -1357.048574
Sum of electronic and thermal Energies= -1357.032819
Sum of electronic and thermal Enthalpies= -1357.031874
Sum of electronic and thermal Free Energies= -1357.092445

2-IIII-CHCH2 (in Acetonitrile)

C	-0.41876	3.43762	-0.15967
C	-1.16397	2.27017	-0.09784
C	-0.52999	1.03221	-0.02753
C	0.84913	1.01581	-0.04231
C	1.61746	2.1628	-0.09392
C	0.96703	3.38735	-0.15074
H	-0.92542	4.39135	-0.20917
H	-2.24234	2.31905	-0.10232
H	2.69618	2.1168	-0.07472
H	1.54863	4.29819	-0.18566
O	-0.39848	-1.3303	0.31156
C	-1.26109	-0.31798	0.07573
C	-1.99172	-0.58258	-1.26577
C	-2.26774	-0.27528	1.25365
C	3.69227	-0.09728	-0.30718
H	3.8903	0.35127	-1.27351
C	4.64437	-0.27851	0.59222
H	4.44534	-0.73212	1.55621
H	5.6684	0.0189	0.39534
I	1.73833	-0.89949	0.00929
F	-1.08047	-0.67749	-2.2404
F	-2.8383	0.39565	-1.61241
F	-2.67961	-1.71692	-1.24111
F	-2.80254	-1.46674	1.47856
F	-1.6363	0.10602	2.3657
F	-3.27912	0.58457	1.05511

Sum of electronic and zero-point Energies= -1395.116840
Sum of electronic and thermal Energies= -1395.099222
Sum of electronic and thermal Enthalpies= -1395.098278
Sum of electronic and thermal Free Energies= -1395.164380

2-IIII-Cl (in Acetonitrile)

C	-0.33995	3.45529	0.00158
C	-1.11658	2.30641	0.00112
C	-0.50908	1.05463	0.00001
C	0.87273	1.01009	-0.00043
C	1.67247	2.13249	0.00009
C	1.04398	3.37077	0.00103
H	-0.82262	4.42245	0.00242
H	-2.19336	2.381	0.00167
H	2.7484	2.0492	-0.00023
H	1.6462	4.26853	0.00138
O	-0.39971	-1.3425	-0.00366
Cl	3.94177	-0.17761	0.00061
C	-1.26233	-0.27275	-0.00052
C	-2.12539	-0.38506	-1.28101
C	-2.12083	-0.38818	1.28274
F	-2.73875	-1.55685	-1.35743
F	-3.05728	0.56969	-1.3576
F	-1.33332	-0.26404	-2.34692
F	-3.05043	0.56816	1.36647
F	-2.73625	-1.55903	1.35719
F	-1.32436	-0.27271	2.34603
I	1.62797	-0.95153	-0.00158

Sum of electronic and zero-point Energies= -1777.406463
Sum of electronic and thermal Energies= -1777.390255
Sum of electronic and thermal Enthalpies= -1777.389310
Sum of electronic and thermal Free Energies= -1777.452331

2-IIII-CN (in Acetonitrile)

C	-0.15433	3.43846	0.00056
C	-0.96932	2.31706	0.00036
C	-0.40638	1.0434	0.00008
C	0.97045	0.95504	0.00007
C	1.81091	2.04842	0.00027
C	1.22629	3.30745	0.00051
H	-0.60353	4.42163	0.00076
H	-2.04277	2.42871	0.00044
H	2.88556	1.93901	0.00026
H	1.85994	4.1832	0.00066
O	-0.3925	-1.35362	-0.00102
C	3.68597	-0.29054	-0.00021
N	4.78181	0.05737	-0.00061
C	-1.21155	-0.25892	-0.00019
C	-2.07832	-0.33136	-1.28122
C	-2.07739	-0.33235	1.28141
F	-2.96959	0.66179	-1.36025
F	-2.74025	-1.47596	-1.35945
F	-1.28041	-0.24304	-2.34699
F	-2.96795	0.66127	1.36233
F	-2.74001	-1.47663	1.3588
F	-1.27858	-0.24582	2.34665
I	1.67135	-1.03509	-0.00018

Sum of electronic and zero-point Energies= -1410.006483
Sum of electronic and thermal Energies= -1409.989463
Sum of electronic and thermal Enthalpies= -1409.988518
Sum of electronic and thermal Free Energies= -1410.053589

2-IIII-F (in Acetonitrile)

C	-0.00297	3.44544	0.00098
C	-0.83215	2.33302	0.00064
C	-0.27679	1.05768	0.00005
C	1.09989	0.95346	-0.00016
C	1.95299	2.0385	0.00022
C	1.37736	3.30188	0.00077
H	-0.44197	4.43325	0.00142
H	-1.9045	2.45641	0.00086
H	3.02274	1.89656	0.00008
H	2.01513	4.17485	0.00103
O	-0.23919	-1.34449	-0.00218
F	3.61105	-0.27969	0.0003
C	-1.07053	-0.2476	-0.00032
C	-1.93505	-0.33428	-1.28095
C	-1.93255	-0.33631	1.28186
F	-2.58767	-1.48503	-1.35688
F	-2.83368	0.65145	-1.35887
F	-1.13856	-0.24179	-2.34643
F	-2.82971	0.65038	1.36412
F	-2.58649	-1.4864	1.3564
F	-1.13363	-0.24742	2.34586
I	1.78168	-1.00999	-0.00085

Sum of electronic and zero-point Energies= -1417.045380
Sum of electronic and thermal Energies= -1417.029603
Sum of electronic and thermal Enthalpies= -1417.028658
Sum of electronic and thermal Free Energies= -1417.090940

2-IIII-N3 (in Acetonitrile)

C	-0.36356	3.44841	0.06229
C	-1.16278	2.31719	0.13356
C	-0.59764	1.05554	-0.02425
C	0.76465	0.982	-0.23542
C	1.58606	2.08722	-0.31438
C	1.00046	3.33704	-0.16434
H	-0.81262	4.42417	0.18334
H	-2.22314	2.41358	0.31191
H	2.64404	1.98103	-0.50165
H	1.61762	4.22248	-0.22598
O	-0.56807	-1.32284	-0.30928
C	-1.37081	-0.26229	0.02256
C	-2.52544	-0.23984	-1.0082
C	-1.89827	-0.49291	1.45998
F	-3.1215	-1.419	-1.09865
F	-3.46198	0.66694	-0.7062
F	-2.03729	0.06777	-2.20931
F	-2.6734	0.50552	1.89171
F	-2.59198	-1.61821	1.55758
F	-0.85469	-0.57942	2.28885
N	3.48802	-0.2732	-0.57154
N	4.0356	-0.1122	0.51249
N	4.59438	0.05699	1.47032
I	1.48234	-0.98427	-0.42035

Sum of electronic and zero-point Energies= -1481.360201
Sum of electronic and thermal Energies= -1481.342460
Sum of electronic and thermal Enthalpies= -1481.341516
Sum of electronic and thermal Free Energies= -1481.408288

2-IIII-NH2 (in Acetonitrile)

C	-0.15633	3.44497	0.00062
C	-0.93721	2.29874	0.00039
C	-0.33706	1.04261	0.00002
C	1.04243	0.98185	-0.00008
C	1.84086	2.10781	0.00019
C	1.22739	3.35292	0.00053
H	-0.63386	4.41482	0.00088
H	-2.0136	2.38005	0.0005
H	2.91771	2.02618	0.00013
H	1.83484	4.24744	0.00071
O	-0.24027	-1.3452	-0.00141
C	-1.09549	-0.28959	-0.00025
C	-1.96468	-0.3834	-1.27848
C	-1.96296	-0.38469	1.27908
F	-1.17288	-0.27042	2.34935
F	-2.59343	-1.54809	1.36118
F	-2.88693	0.58173	1.36061
F	-2.59431	-1.54718	-1.36148
F	-2.88961	0.58238	-1.35707
F	-1.17634	-0.26677	-2.34975
N	3.81129	-0.35266	0.00021

H 4.1009 0.14854 0.82966
H 4.10147 0.14897 -0.82879
I 1.86444 -0.96129 -0.00058
Sum of electronic and zero-point Energies= -1373.108725
Sum of electronic and thermal Energies= -1373.093247
Sum of electronic and thermal Enthalpies= -1373.092303
Sum of electronic and thermal Free Energies= -1373.152135

2-IIII-NHAc (in Acetonitrile)
C -0.55504 3.42469 0.31782
C -1.37995 2.32177 0.16032
C -0.83069 1.04493 0.08906
C 0.54038 0.92436 0.19784
C 1.386 2.00277 0.35166
C 0.82033 3.26855 0.40916
H -0.9911 4.41272 0.36527
H -2.44954 2.45119 0.09226
H 2.45379 1.86726 0.41545
H 1.46146 4.13193 0.52016
O -0.81526 -1.31517 -0.29231
C 4.04515 -0.01652 -0.383
O 3.67009 0.28194 -1.49488
C 5.48998 0.09521 0.05323
H 5.57384 0.66443 0.97894
H 5.89686 -0.90026 0.23206
H 6.05841 0.58227 -0.73227
C -1.63669 -0.24294 -0.09544
C -2.54441 -0.13075 -1.34468
C -2.47297 -0.50081 1.18269
F -3.13333 -1.28483 -1.62321
F -3.50823 0.78891 -1.19982
F -1.80889 0.21988 -2.39801
F -3.28507 0.51545 1.4896
F -3.21582 -1.59464 1.07753
F -1.63907 -0.67006 2.21393
N 3.17408 -0.47267 0.57105
H 3.53897 -0.68327 1.48773
I 1.23663 -1.05418 0.0994
Sum of electronic and zero-point Energies= -1525.739703
Sum of electronic and thermal Energies= -1525.719651
Sum of electronic and thermal Enthalpies= -1525.718707
Sum of electronic and thermal Free Energies= -1525.791037

2-IIII-OCOCH3 (in Acetonitrile)
C -1.1116 3.46342 0.00016
C -1.75066 2.2332 0.00011
C -1.00205 1.06049 -0.00002
C 0.37735 1.16564 -0.00011
C 1.03962 2.37937 -0.00007
C 0.27318 3.53653 0.00007
H -1.70174 4.36908 0.00027
H -2.8288 2.18386 0.00019
H 2.11617 2.42074 -0.00014
H 0.76788 4.49792 0.00009
O -0.62214 -1.30809 -0.00066
C 4.16896 -0.36895 -0.00013
O 4.06239 -1.57531 -0.00064
O 3.11731 0.43172 0.00008
C 5.48246 0.36367 0.00052
H 5.5392 1.00641 -0.87663
H 5.54144 0.9998 0.88238
H 6.29817 -0.35144 -0.00295
C -1.60077 -0.34426 -0.00007
C -2.44445 -0.5548 -1.28082
C -2.44346 -0.55525 1.28124
F -2.92395 -1.7877 -1.35594
F -3.47716 0.29016 -1.36136
F -1.66988 -0.34861 -2.34685
F -3.47559 0.29028 1.36332
F -2.9236 -1.78791 1.35593
F -1.6678 -0.35022 2.34672
I 1.34184 -0.68946 -0.00028
Sum of electronic and zero-point Energies= -1545.627927
Sum of electronic and thermal Energies= -1545.608039
Sum of electronic and thermal Enthalpies= -1545.607095
Sum of electronic and thermal Free Energies= -1545.680542

2-IIII-OH (in Acetonitrile)
C -0.07761 3.4457 0.13049
C -0.88468 2.3178 0.0973
C -0.30828 1.05446 0.01241
C 1.06938 0.97321 -0.01961
C 1.89891 2.07595 0.00909
C 1.30417 3.32834 0.08247
H -0.53438 4.42356 0.19195
H -1.95876 2.41909 0.13804
H 2.9707 1.95508 -0.03733
H 1.92678 4.21216 0.10003
O -0.24238 -1.3216 -0.26874
O 3.69258 -0.27285 -0.00518
H 3.98479 -0.24994 0.91048
C -1.08066 -0.26493 -0.04077
C -2.09909 -0.23962 -1.20585
C -1.78527 -0.49336 1.3189
F -2.68376 -1.41746 -1.3692
F -3.0651 0.66984 -1.02444
F -1.46662 0.06535 -2.33844
F -2.61254 0.50316 1.64856
F -2.48281 -1.62104 1.33398
F -0.85509 -0.57419 2.27514
I 1.81663 -0.97775 -0.11602
Sum of electronic and zero-point Energies= -1392.999656
Sum of electronic and thermal Energies= -1392.983486
Sum of electronic and thermal Enthalpies= -1392.982542
Sum of electronic and thermal Free Energies= -1393.045018

2-IIII-OTs (in Acetonitrile)
C -2.87892 3.24576 -0.16697
C -3.36996 1.94905 -0.19302
C -2.50115 0.87624 -0.0234
C -1.16096 1.15565 0.16919
C -0.64086 2.43215 0.20957
C -1.5281 3.48612 0.03394
H -3.56032 4.07364 -0.30366
H -4.42375 1.77063 -0.3437
H 0.40972 2.62078 0.37277
H -1.14965 4.49842 0.0544
O -1.83809 -1.42933 0.11656
C -2.92385 -0.58917 -0.02163
C -3.59588 -0.95525 -1.36671
C -3.85996 -0.85913 1.18148
F -3.87883 -2.24822 -1.43547
F -4.72982 -0.2778 -1.5677
F -2.76271 -0.66203 -2.36266
F -4.97069 -0.12002 1.14097
F -4.22034 -2.13285 1.24848
F -3.21051 -0.55554 2.30646
O 1.68313 0.6095 0.75558
S 2.5617 1.20418 -0.38343
O 2.80469 2.5773 -0.06536
O 1.96157 0.86751 -1.64227
C 4.07098 0.3127 -0.21911
C 5.01845 0.74099 0.70035
C 4.28538 -0.81156 -0.99995
C 6.19158 0.02059 0.83905
H 4.83345 1.63127 1.28543
C 5.46768 -1.5206 -0.84847
H 3.54171 -1.11066 -1.72604
C 6.43344 -1.11745 0.06929
H 6.93703 0.34679 1.55349
H 5.64531 -2.39761 -1.45771
C 7.72691 -1.86867 0.21085
H 7.65 -2.87117 -0.2056
H 8.5291 -1.34815 -0.31519
H 8.02012 -1.94972 1.25687
I -0.01439 -0.57637 0.40057
Sum of electronic and zero-point Energies= -2211.884123
Sum of electronic and thermal Energies= -2211.858342
Sum of electronic and thermal Enthalpies= -2211.857398
Sum of electronic and thermal Free Energies= -2211.944997

3-BrIII-anion (in Acetonitrile)
C -2.64228 -1.29154 0.00032
C -2.24654 0.03444 0.00033
C -0.90415 0.42563 0.00017
C 0.03468 -0.60152 -0.00003
C -0.33995 -1.94385 -0.00006
C -1.67917 -2.29263 0.00012
H -3.69515 -1.54612 0.00046
H -2.96304 0.84502 0.00046
H 0.42724 -2.70568 -0.00024
H -1.96235 -3.33796 0.0001
Br 1.92399 -0.31138 -0.00033
C -0.62452 1.97299 0.00026
O 0.56426 2.31189 0.00046
O -1.65611 2.66583 0.00006
11 Br 11 3.0600 1.000 1.923991 -
0.311377 -0.000328
11 Br 11 3.0600 1.000 1.935989 -
0.282209 -0.000213
11 Br 11 3.0600 1.000 1.926880 -
0.309114 0.003862
11 Br 11 3.0600 1.000 1.926008 -
0.312280 -0.000239
11 Br 11 3.0600 1.000 1.931610 -
0.292699 -0.000356
11 Br 11 3.0600 1.000 1.928803 -
0.300580 -0.000664
11 Br 11 3.0600 1.000 1.928978 -
0.300481 -0.001428
11 Br 11 3.0600 1.000 1.929757 -
0.299858 -0.003650
11 Br 11 3.0600 1.000 1.929380 -
0.308283 -0.016837
11 Br 11 3.0600 1.000 1.895989 -
0.405951 -0.033474

11 Br 11 3.0600 1.000 1.909563 -
0.368921 -0.041610
11 Br 11 3.0600 1.000 2.012396
0.030242 -0.062140
11 Br 11 3.0600 1.000 1.977249 -
0.157339 -0.067998
11 Br 11 3.0600 1.000 1.900047 -
0.383943 -0.060915
11 Br 11 3.0600 1.000 1.878867 -
0.381985 -0.007051
11 Br 11 3.0600 1.000 1.890718 -
0.402251 -0.081855
11 Br 11 3.0600 1.000 1.890435 -
0.409874 -0.083536
11 Br 11 3.0600 1.000 1.881704 -
0.433882 -0.082626
11 Br 11 3.0600 1.000 1.857379 -
0.508824 -0.078008
11 Br 11 3.0600 1.000 1.821801 -
0.604509 -0.068177
11 Br 11 3.0600 1.000 1.740900 -
0.761106 -0.027642
11 Br 11 3.0600 1.000 1.726978 -
0.783701 -0.022355
11 Br 11 3.0600 1.000 1.712452 -
0.805327 -0.006806
11 Br 11 3.0600 1.000 1.758448 -
0.730788 -0.050397
11 Br 11 3.0600 1.000 1.729949 -
0.778484 -0.034088
11 Br 11 3.0600 1.000 1.712656 -
0.804634 -0.021336
11 Br 11 3.0600 1.000 1.711922 -
0.806354 -0.018931
11 Br 11 3.0600 1.000 1.712320 -
0.805969 -0.017600
11 Br 11 3.0600 1.000 1.708700 -
0.811099 -0.012795
11 Br 11 3.0600 1.000 1.711597 -
0.807053 -0.016124
11 Br 11 3.0600 1.000 1.711029 -
0.807877 -0.015864
11 Br 11 3.0600 1.000 1.711029 -
0.807877 -0.015864
Sum of electronic and zero-point Energies= -2993.861551
Sum of electronic and thermal Energies= -2993.853153
Sum of electronic and thermal Enthalpies= -2993.852208
Sum of electronic and thermal Free Energies= -2993.896690

3-BrIII-Br (in Acetonitrile)
C -2.63836 2.37145 -0.00017
C -2.96026 1.02422 -0.00021
C -1.94291 0.08138 -0.00015
C -0.6403 0.52162 -0.00005
C -0.27053 1.84604 -0.00002
C -1.30744 2.77375 -0.00008
H -3.42304 3.11482 -0.00022
H -3.98224 0.66925 -0.00028
H 0.76272 2.15392 0.00006
H -1.06056 3.82641 -0.00005
Br 0.59854 -0.97968 0.00003
C -2.19376 -1.39221 -0.00019
O -1.06845 -2.08072 -0.00012
O -3.29097 -1.87622 -0.00027
Br 2.66717 0.36635 0.00022
11 Br 11 3.0600 1.000 -0.598544 -
0.979677 -0.000027
15 Br 15 3.0600 1.000 -2.667166
0.366353 -0.000224
11 Br 11 3.0600 1.000 -0.608476 -
0.959257 -0.000018
15 Br 15 3.0600 1.000 -2.683863
0.370310 -0.000239
11 Br 11 3.0600 1.000 -0.605379 -
0.972617 -0.000204
15 Br 15 3.0600 1.000 -2.671290
0.365291 0.000046
11 Br 11 3.0600 1.000 -0.607469 -
0.968192 0.002305
15 Br 15 3.0600 1.000 -2.674221
0.366496 -0.001851
11 Br 11 3.0600 1.000 -0.611199 -
0.962417 -0.041240
15 Br 15 3.0600 1.000 -2.675431
0.367681 0.033050
11 Br 11 3.0600 1.000 -0.608565 -
0.967253 -0.000106
15 Br 15 3.0600 1.000 -2.673533
0.366265 -0.000302
11 Br 11 3.0600 1.000 -0.608565 -
0.967253 -0.000106
15 Br 15 3.0600 1.000 -2.673533
0.366265 -0.000302

Sum of electronic and zero-point Energies= -5567.837920
Sum of electronic and thermal Energies= -5567.828081
Sum of electronic and thermal Enthalpies= -5567.827137
Sum of electronic and thermal Free Energies= -5567.875384

3-BrIII-CCH (in Acetonitrile)
C -2.33918 2.20085 -0.00008
C -2.52969 0.82843 -0.00026
C -1.43423 -0.02574 -0.00008
C -0.18928 0.54909 0.0002
C 0.0611 1.90208 0.00042
C -1.05434 2.73224 0.0003
H -3.19245 2.86482 -0.00022
H -3.51281 0.37676 -0.00053
H 1.06243 2.30384 0.00075
H -0.90796 3.8035 0.00053
Br 1.24671 -0.78724 0.00015
C -1.57539 -1.53805 -0.00007
O -0.4302 -2.12237 0.00067
O -2.67577 -2.03854 -0.00074
C 3.62908 1.1593 -0.00079
H 4.50254 1.76805 -0.0011
C 2.64213 0.47909 -0.00033
11 Br 11 3.0600 1.000 -1.246710 -
0.787240 -0.000148
11 Br 11 3.0600 1.000 -1.264428 -
0.875125 -0.000214
11 Br 11 3.0600 1.000 -1.271883 -
0.811543 -0.000558
11 Br 11 3.0600 1.000 -1.259095 -
0.800642 0.010068
11 Br 11 3.0600 1.000 -1.266291 -
0.783158 -0.093199
11 Br 11 3.0600 1.000 -1.264262 -
0.790105 -0.002240
11 Br 11 3.0600 1.000 -1.269863 -
0.783318 0.035537
11 Br 11 3.0600 1.000 -1.265510 -
0.793120 -0.000808
11 Br 11 3.0600 1.000 -1.265198 -
0.793017 0.000229
11 Br 11 3.0600 1.000 -1.265052 -
0.792729 -0.000139
11 Br 11 3.0600 1.000 -1.265082 -
0.792779 0.000056
11 Br 11 3.0600 1.000 -1.265082 -
0.792779 0.000056
Sum of electronic and zero-point Energies= -3070.333589
Sum of electronic and thermal Energies= -3070.322904
Sum of electronic and thermal Enthalpies= -3070.321960
Sum of electronic and thermal Free Energies= -3070.371072

3-BrIII-CF3 (in Acetonitrile)
C 2.67427 2.27507 -0.00001
C 2.93756 0.91591 0.0001
C 1.89025 0.00382 -0.00004
C 0.61006 0.50004 -0.0002
C 0.29331 1.84339 -0.00031
C 1.36207 2.73201 -0.00022
H 3.48936 2.98523 0.00007
H 3.94286 0.51621 0.00029
H -0.71478 2.22031 -0.00048
H 1.1538 3.79289 -0.00032
Br -0.72549 -0.93456 -0.00024
C 2.13383 -1.49514 0.00004
O 1.03312 -2.16052 -0.00088
O 3.26518 -1.91932 0.00104
C -2.35345 0.27073 0.00025
F -3.3784 -0.5678 0.00043
F -2.43067 1.03725 -1.07709
F -2.43012 1.03706 1.07776
11 Br 11 3.0600 1.000 -0.725494 -
0.934556 -0.000237
11 Br 11 3.0600 1.000 -0.763497 -
0.954802 -0.000488
11 Br 11 3.0600 1.000 -0.760840 -
0.904525 -0.001160
11 Br 11 3.0600 1.000 -0.745883 -
0.918776 0.018106
11 Br 11 3.0600 1.000 -0.747774 -
0.910484 -0.078468
11 Br 11 3.0600 1.000 -0.744506 -
0.913054 0.041319
11 Br 11 3.0600 1.000 -0.744993 -
0.916906 -0.011792
11 Br 11 3.0600 1.000 -0.744970 -
0.911368 0.011069
11 Br 11 3.0600 1.000 -0.745353 -
0.912388 0.001751
11 Br 11 3.0600 1.000 -0.746226 -
0.912912 0.000849
11 Br 11 3.0600 1.000 -0.746348 -
0.912828 0.000548

11 Br 11 3.0600 1.000 -0.746348 -
0.912828 0.000548
Sum of electronic and zero-point Energies= -3331.277938
Sum of electronic and thermal Energies= -3331.265754
Sum of electronic and thermal Enthalpies= -3331.264810
Sum of electronic and thermal Free Energies= -3331.317973

3-BrIII-CH3 (in Acetonitrile)
C 2.68911 1.6098 0.00003
C 2.51212 0.23579 0.00001
C 1.23564 -0.3128 0.00003
C 0.17416 0.56033 0.00005
C 0.29744 1.93574 0.00006
C 1.58679 2.45486 0.00005
H 3.68606 2.02841 0.00002
H 3.3422 -0.45835 -0.00003
H -0.54848 2.60334 0.00008
H 1.71939 3.52797 0.00007
Br -1.57045 -0.31046 0.00005
C 1.0184 -1.82903 -0.00002
O -0.21422 -2.13627 0.00029
O 1.99913 -2.54616 -0.00033
C -2.66346 1.32609 -0.00029
H -2.46589 1.89557 -0.90164
H -3.68161 0.94827 -0.00053
H -2.46635 1.89565 0.9011
11 Br 11 3.0600 1.000 1.570447 -
0.310461 -0.000053
11 Br 11 3.0600 1.000 1.593270 -
0.490386 -0.000043
11 Br 11 3.0600 1.000 1.588676 -
0.405146 -0.000527
11 Br 11 3.0600 1.000 1.578157 -
0.407006 0.002658
11 Br 11 3.0600 1.000 1.576294 -
0.400577 -0.031775
11 Br 11 3.0600 1.000 1.578507 -
0.399846 -0.009196
11 Br 11 3.0600 1.000 1.577574 -
0.373988 0.038160
11 Br 11 3.0600 1.000 1.579094 -
0.399213 -0.002501
11 Br 11 3.0600 1.000 1.579086 -
0.398709 -0.004527
11 Br 11 3.0600 1.000 1.579220 -
0.397410 -0.009130
11 Br 11 3.0600 1.000 1.580334 -
0.396226 -0.017276
11 Br 11 3.0600 1.000 1.579296 -
0.394582 -0.017324
11 Br 11 3.0600 1.000 1.579984 -
0.398590 -0.013185
11 Br 11 3.0600 1.000 1.579863 -
0.398305 -0.015875
11 Br 11 3.0600 1.000 1.579763 -
0.398396 -0.014710
11 Br 11 3.0600 1.000 1.579884 -
0.398424 -0.015190
11 Br 11 3.0600 1.000 1.579852 -
0.398420 -0.014997
11 Br 11 3.0600 1.000 1.579852 -
0.398420 -0.014997
Sum of electronic and zero-point Energies= -3033.508510
Sum of electronic and thermal Energies= -3033.498283
Sum of electronic and thermal Enthalpies= -3033.497339
Sum of electronic and thermal Free Energies= -3033.545428

3-BrIII-CHCH2 (in Acetonitrile)
C -2.48117 2.11786 -0.04135
C -2.61555 0.73872 -0.05135
C -1.49311 -0.07982 -0.0134
C -0.26949 0.54011 0.04006
C -0.0778 1.90617 0.0374
C -1.21909 2.69829 -0.00247
H -3.35962 2.74761 -0.07222
H -3.579 0.24799 -0.09384
H 0.90295 2.35579 0.04785
H -1.11131 3.77418 -0.00771
C -1.60609 -1.60598 -0.06643
O -0.46689 -2.16976 -0.09355
O -2.71684 -2.09707 -0.08605
C 2.61871 0.64578 0.35407
H 2.58955 1.15916 1.30494
C 3.56442 0.78578 -0.55268
H 4.39416 1.46001 -0.38055
H 3.54911 0.23708 -1.48558
Br 1.24454 -0.69511 0.11145
19 Br 19 3.0600 1.000 -1.244544 -
0.695106 -0.111451
19 Br 19 3.0600 1.000 -1.271926 -
0.773290 -0.115053
19 Br 19 3.0600 1.000 -1.268478 -
0.758062 0.019498
19 Br 19 3.0600 1.000 -1.274723 -
0.751485 0.015573
19 Br 19 3.0600 1.000 -1.278936 -
0.726086 -0.087951
19 Br 19 3.0600 1.000 -1.271016 -
0.725266 -0.098419
19 Br 19 3.0600 1.000 -1.267020 -
0.704206 -0.113876
19 Br 19 3.0600 1.000 -1.264000 -
0.716752 -0.118083
19 Br 19 3.0600 1.000 -1.263993 -
0.717645 -0.114451
19 Br 19 3.0600 1.000 -1.263628 -
0.718857 -0.112976
19 Br 19 3.0600 1.000 -1.263681 -
0.718509 -0.112975
19 Br 19 3.0600 1.000 -1.263681 -
0.718509 -0.112975
Sum of electronic and zero-point Energies= -3071.576951
Sum of electronic and thermal Energies= -3071.565793
Sum of electronic and thermal Enthalpies= -3071.564849
Sum of electronic and thermal Free Energies= -3071.615656

3-BrIII-Cl (in Acetonitrile)
C 2.57036 2.02836 -0.00005
C 2.66222 0.64617 -0.00009
C 1.50008 -0.11112 -0.00007
C 0.29068 0.5423 0.
C 0.1472 1.91019 0.00005
C 1.32581 2.6493 0.00002
H 3.46851 2.62975 -0.00007
H 3.61065 0.12581 -0.00014
H -0.82077 2.3845 0.0001
H 1.26064 3.72856 0.00006
Br -1.17897 -0.72602 0.00003
C 1.49061 -1.60536 -0.00011
O 0.25926 -2.08527 0.00001
O 2.48371 -2.27636 -0.00008
Cl -2.83062 0.88682 0.00006
11 Br 11 3.0600 1.000 1.178974 -
0.726015 -0.000032
11 Br 11 3.0600 1.000 1.183575 -
0.707446 0.000003
11 Br 11 3.0600 1.000 1.180410 -
0.722041 -0.000274
11 Br 11 3.0600 1.000 1.181263 -
0.718657 0.003210
11 Br 11 3.0600 1.000 1.182815 -
0.713462 -0.020500
11 Br 11 3.0600 1.000 1.181507 -
0.718129 0.000072
11 Br 11 3.0600 1.000 1.181527 -
0.718328 0.000365
11 Br 11 3.0600 1.000 1.181665 -
0.718363 -0.000005
11 Br 11 3.0600 1.000 1.181665 -
0.718363 -0.000005
Sum of electronic and zero-point Energies= -3453.835370
Sum of electronic and thermal Energies= -3453.825776
Sum of electronic and thermal Enthalpies= -3453.824832
Sum of electronic and thermal Free Energies= -3453.871866

3-BrIII-CN (in Acetonitrile)
C 2.38344 2.13726 -0.00003
C 2.53599 0.7602 0.
C 1.41264 -0.05577 -0.00004
C 0.18413 0.55355 -0.00007
C -0.02895 1.91265 -0.00008
C 1.11425 2.70536 -0.00007
H 3.25483 2.77693 -0.00001
H 3.50611 0.28155 0.00004
H -1.01434 2.35315 -0.00011
H 0.99954 3.78034 -0.00009
Br -1.25543 -0.77664 -0.00007
C 1.49016 -1.56256 0.00001
O 0.30608 -2.10371 -0.0003
O 2.54777 -2.13552 0.00043
C -2.62762 0.60631 0.00017
N -3.48875 1.36603 0.0003
11 Br 11 3.0600 1.000 -1.255425 -
0.776638 -0.000069
11 Br 11 3.0600 1.000 -1.265086 -
0.747782 -0.000239
11 Br 11 3.0600 1.000 -1.261704 -
0.760656 0.003161
11 Br 11 3.0600 1.000 -1.264935 -
0.753135 -0.044430
11 Br 11 3.0600 1.000 -1.263994 -
0.754517 -0.000449
11 Br 11 3.0600 1.000 -1.265247 -
0.754054 0.000300
11 Br 11 3.0600 1.000 -1.265854 -
0.754322 0.000442

11 Br 11 3.0600 1.000 -1.266089 -
0.754921 0.000101
11 Br 11 3.0600 1.000 -1.266089 -
0.754921 0.000101
Sum of electronic and zero-point Energies= -3086.435777
Sum of electronic and thermal Energies= -3086.425405
Sum of electronic and thermal Enthalpies= -3086.424461
Sum of electronic and thermal Free Energies= -3086.472849

3-BrIII-F (in Acetonitrile)
C 2.96785 -0.98514 -0.00011
C 2.49999 0.3192 -0.00012
C 1.13062 0.54082 -0.00009
C 0.28825 -0.54352 -0.00002
C 0.70207 -1.85646 -0.00001
C 2.0783 -2.05669 -0.00007
H 4.03187 -1.17549 -0.00014
H 3.16286 1.1739 -0.00018
H -0.00534 -2.67002 0.00002
H 2.45731 -3.0694 -0.00006
Br -1.54776 0.00413 0.00011
C 0.49326 1.88974 -0.00018
O -0.83287 1.79682 -0.00004
O 1.09682 2.92326 -0.00023
F -2.06097 -1.7791 0.00026
11 Br 11 3.0600 1.000 1.547760
0.004130 -0.000107
11 Br 11 3.0600 1.000 1.545397
0.004795 -0.000206
11 Br 11 3.0600 1.000 1.547974
0.006133 0.002145
11 Br 11 3.0600 1.000 1.548878
0.001015 -0.044642
11 Br 11 3.0600 1.000 1.547652
0.005591 -0.016519
11 Br 11 3.0600 1.000 1.546785
0.005345 0.000446
11 Br 11 3.0600 1.000 1.546763
0.005226 0.000463
11 Br 11 3.0600 1.000 1.546917
0.005770 0.000010
11 Br 11 3.0600 1.000 1.546917
0.005770 0.000010
Sum of electronic and zero-point Energies= -3093.468764
Sum of electronic and thermal Energies= -3093.459778
Sum of electronic and thermal Enthalpies= -3093.458834
Sum of electronic and thermal Free Energies= -3093.503830

3-BrIII-N3 (in Acetonitrile)
C -2.3804 2.32794 0.05934
C -2.65705 0.97933 0.217
C -1.62888 0.05538 0.09806
C -0.36432 0.51848 -0.16973
C -0.03949 1.84488 -0.34251
C -1.08571 2.75318 -0.2202
H -3.17412 3.05617 0.14975
H -3.65173 0.61059 0.42901
H 0.9644 2.16218 -0.5759
H -0.87862 3.8065 -0.34928
Br 0.91936 -0.93103 -0.29065
C -1.82322 -1.42589 0.23462
O -0.69924 -2.07945 0.05634
O -2.89125 -1.92083 0.47378
N 2.4447 0.298 -0.67238
N 3.00971 0.59854 0.37701
N 3.56857 0.9082 1.29805
11 Br 11 3.0600 1.000 0.919359 -
0.931032 -0.290648
11 Br 11 3.0600 1.000 0.902435 -
0.978032 -0.239904
11 Br 11 3.0600 1.000 0.916884 -
0.994022 -0.297296
11 Br 11 3.0600 1.000 0.925378 -
0.940211 -0.286520
11 Br 11 3.0600 1.000 0.908694 -
0.979195 -0.271547
11 Br 11 3.0600 1.000 0.919516 -
0.949862 -0.276631
11 Br 11 3.0600 1.000 0.919519 -
0.954664 -0.276760
11 Br 11 3.0600 1.000 0.919363 -
0.956548 -0.277040
11 Br 11 3.0600 1.000 0.919337 -
0.956828 -0.277388
11 Br 11 3.0600 1.000 0.920148 -
0.954379 -0.277602
11 Br 11 3.0600 1.000 0.919639 -
0.956130 -0.277336
11 Br 11 3.0600 1.000 0.919639 -
0.956130 -0.277336
Sum of electronic and zero-point Energies= -3157.791213
Sum of electronic and thermal Energies= -3157.780074
Sum of electronic and thermal Enthalpies= -3157.779130
Sum of electronic and thermal Free Energies= -3157.830140

3-BrIII-NH2 (in Acetonitrile)
C 2.8199 -1.38764 0.00001
C 2.53393 -0.0313 -0.00001
C 1.21429 0.39728 -0.00001
C 0.22299 -0.55389 0.
C 0.46009 -1.91182 0.00002
C 1.78938 -2.32031 0.00001
H 3.84705 -1.72496 0.00002
H 3.3069 0.72585 -0.00002
H -0.3356 -2.64049 0.00001
H 2.01343 -3.37814 0.00002
Br -1.57489 0.19181 -0.00001
C 0.82223 1.86015 0.00001
O -0.45795 2.00441 0.00011
O 1.67149 2.72047 -0.00007
N -2.55477 -1.41277 -0.00003
H -2.3563 -1.95999 0.8295
H -2.35632 -1.96001 -0.82956
11 Br 11 3.0600 1.000 1.574885
0.191806 0.000006
11 Br 11 3.0600 1.000 1.606481
0.381325 0.000053
11 Br 11 3.0600 1.000 1.597894
0.303473 0.000866
11 Br 11 3.0600 1.000 1.587151
0.251407 -0.020002
11 Br 11 3.0600 1.000 1.584605
0.240963 0.019986
11 Br 11 3.0600 1.000 1.586401
0.247225 0.004225
11 Br 11 3.0600 1.000 1.585831
0.243110 -0.001163
11 Br 11 3.0600 1.000 1.586921
0.247024 0.001536
11 Br 11 3.0600 1.000 1.587259
0.248380 -0.000267
11 Br 11 3.0600 1.000 1.586809
0.247204 -0.000352
11 Br 11 3.0600 1.000 1.586809
0.247204 -0.000352
Sum of electronic and zero-point Energies= -3049.550581
Sum of electronic and thermal Energies= -3049.540831
Sum of electronic and thermal Enthalpies= -3049.539887
Sum of electronic and thermal Free Energies= -3049.586523

3-BrIII-NHAc (in Acetonitrile)
C -2.29613 2.61008 0.02303
C -2.7867 1.32281 -0.12215
C -1.91568 0.24503 -0.04496
C -0.58598 0.50136 0.17979
C -0.04888 1.75988 0.32359
C -0.93929 2.82416 0.24244
H -2.96782 3.45495 -0.03844
H -3.83363 1.11512 -0.29838
H 1.00444 1.91798 0.48576
H -0.55994 3.83134 0.34621
Br 0.473 -1.12507 0.24976
C -2.35604 -1.18682 -0.20159
O -1.35083 -2.0067 -0.10764
O -3.5107 -1.47651 -0.38869
C 2.8961 0.26307 -0.37422
O 2.45978 0.42595 -1.48793
C 4.321 0.58149 0.01814
H 4.36466 1.11223 0.9684
H 4.87983 -0.34912 0.12197
H 4.77227 1.18212 -0.76497
N 2.10747 -0.21942 0.64339
H 2.5164 -0.41943 1.54373
Sum of electronic and zero-point Energies= -3202.176852
Sum of electronic and thermal Energies= -3202.163420
Sum of electronic and thermal Enthalpies= -3202.162476
Sum of electronic and thermal Free Energies= -3202.218620

3-BrIII-OCOCH3 (in Acetonitrile)
C -2.96551 2.1948 0.00012
C -3.14135 0.82115 0.00005
C -2.02605 -0.00366 -0.00001
C -0.77557 0.56842 0.00001
C -0.55317 1.92978 0.00009
C -1.68457 2.73807 0.00014
H -3.82524 2.85 0.00016
H -4.11978 0.3599 0.00003
H 0.44096 2.34251 0.00011
H -1.55512 3.81166 0.0002
Br 0.5975 -0.7825 -0.00007
C -2.1044 -1.49596 -0.00007
O -0.90086 -2.04357 -0.00014
O -3.13436 -2.1089 -0.00015
C 3.15326 0.18449 -0.00018
O 3.38959 -0.99771 -0.00038
O 1.91808 0.67682 -0.00004

C 4.2019 1.26499 0.0006
H 4.07476 1.89669 -0.87709
H 4.0784 1.89115 0.88282
H 5.18679 0.80999 -0.00262
Sum of electronic and zero-point Energies= -3222.056788
Sum of electronic and thermal Energies= -3222.043648
Sum of electronic and thermal Enthalpies= -3222.042704
Sum of electronic and thermal Free Energies= -3222.098842

3-BrIII-OH (in Acetonitrile)
C 2.89956 -1.19212 0.007
C 2.52512 0.14231 0.02093
C 1.17699 0.4696 0.01162
C 0.25802 -0.55031 -0.00552
C 0.57866 -1.88914 -0.02903
C 1.93531 -2.19509 -0.02064
H 3.94724 -1.45863 0.0126
H 3.24676 0.94805 0.03599
H -0.18468 -2.64988 -0.06644
H 2.23699 -3.2333 -0.04062
Br -1.55942 0.09824 -0.01568
C 0.65434 1.87705 0.00738
O -0.65415 1.89995 -0.02916
O 1.37918 2.83655 0.03131
O -2.26855 -1.6321 -0.03024
H -2.48617 -1.85383 0.88142
11 Br 11 3.0600 1.000 -1.559424
0.098242 -0.015676
11 Br 11 3.0600 1.000 -1.563646
0.102655 0.006717
11 Br 11 3.0600 1.000 -1.577266
0.102594 -0.050271
11 Br 11 3.0600 1.000 -1.570195
0.114440 -0.021652
11 Br 11 3.0600 1.000 -1.567625
0.114291 -0.006832
11 Br 11 3.0600 1.000 -1.566644
0.113664 -0.007906
11 Br 11 3.0600 1.000 -1.567393
0.114967 -0.007903
11 Br 11 3.0600 1.000 -1.567694
0.115882 -0.007758
11 Br 11 3.0600 1.000 -1.567665
0.116163 -0.007536
11 Br 11 3.0600 1.000 -1.567605
0.116050 -0.007481
11 Br 11 3.0600 1.000 -1.567589
0.116061 -0.007346
11 Br 11 3.0600 1.000 -1.567589
0.116061 -0.007346
Sum of electronic and zero-point Energies= -3069.431414
Sum of electronic and thermal Energies= -3069.421930
Sum of electronic and thermal Enthalpies= -3069.420986
Sum of electronic and thermal Free Energies= -3069.466937

3-BrIII-OTs (in Acetonitrile)
C 4.57226 1.91751 0.18665
C 4.71592 0.54189 0.25464
C 3.602 -0.25985 0.05175
C 2.39381 0.34125 -0.20713
C 2.19928 1.70054 -0.29103
C 3.3303 2.48405 -0.08416
H 5.42867 2.55778 0.34429
H 5.66465 0.06505 0.4605
H 1.2402 2.14726 -0.50274
H 3.22741 3.55907 -0.13552
Br 1.02293 -0.99893 -0.4404
C 3.63148 -1.74679 0.09055
O 2.42319 -2.26559 -0.15055
O 4.60287 -2.40915 0.30253
O -0.33734 0.40654 -0.82125
S -1.09027 1.11331 0.3459
O -1.12365 2.51109 0.04231
O -0.52114 0.67438 1.58713
C -2.72341 0.47039 0.21116
C -3.62647 1.07964 -0.64767
C -3.08134 -0.64066 0.95907
C -4.90252 0.5549 -0.76208
H -3.32937 1.95957 -1.20125
C -4.36324 -1.15192 0.83247
H -2.36899 -1.07887 1.64481
C -5.28753 -0.56756 -0.03033
H -5.61655 1.02832 -1.4241
H -4.65458 -2.01264 1.42097
C -6.66661 -1.14379 -0.1835
H -6.9717 -1.6806 0.71302
H -7.3987 -0.36375 -0.38651
H -6.69419 -1.84749 -1.01755
Sum of electronic and zero-point Energies= -3888.309879
Sum of electronic and thermal Energies= -3888.290915
Sum of electronic and thermal Enthalpies= -3888.289971
Sum of electronic and thermal Free Energies= -3888.360117

3-ClIII-anion (in Acetonitrile)
C 2.05854 -1.52427 0.00025
C 0.67902 -1.63937 0.00026
C -0.17525 -0.53359 0.0001
C 0.43702 0.71849 -0.00011
C 1.82431 0.85645 -0.00014
C 2.63871 -0.26192 0.00005
H 2.67995 -2.41149 0.00039
H 0.18606 -2.60246 0.00039
H 2.24856 1.85134 -0.00031
H 3.7153 -0.14396 0.00003
C -1.71813 -0.8321 0.00019
O -2.46568 0.15242 0.00042
O -1.99008 -2.04548 -0.00005
Cl -0.44995 2.22053 -0.00041
Sum of electronic and zero-point Energies= -879.864582
Sum of electronic and thermal Energies= -879.857430
Sum of electronic and thermal Enthalpies= -879.856486
Sum of electronic and thermal Free Energies= -879.897154

3-ClIII-Br (in Acetonitrile)
C -2.43457 2.28833 -0.00007
C -2.79673 0.95148 -0.00018
C -1.80775 -0.01855 -0.00011
C -0.49007 0.37492 0.00003
C -0.08219 1.68867 0.0002
C -1.0917 2.64626 0.00015
H -3.19496 3.05642 -0.00015
H -3.82838 0.62569 -0.00029
H 0.95727 1.97059 0.00038
H -0.81059 3.69021 0.00028
C -2.07965 -1.48447 -0.00001
O -0.95365 -2.16634 0.00019
O -3.17886 -1.96477 -0.00006
Br 2.68906 0.0824 -0.00007
Cl 0.61856 -1.05046 0.00006
14 Br 14 3.0600 1.000 2.689059
0.082403 -0.000069
14 Br 14 3.0600 1.000 2.702637
0.091820 -0.000920
14 Br 14 3.0600 1.000 2.688619
0.081918 0.005154
14 Br 14 3.0600 1.000 2.685878
0.082642 -0.046992
14 Br 14 3.0600 1.000 2.691301
0.083411 -0.015281
14 Br 14 3.0600 1.000 2.691891
0.084335 -0.001215
14 Br 14 3.0600 1.000 2.691246
0.083377 -0.000599
14 Br 14 3.0600 1.000 2.690989
0.082849 -0.000730
14 Br 14 3.0600 1.000 2.691194
0.082992 -0.000563
14 Br 14 3.0600 1.000 2.691030
0.082916 -0.000665
14 Br 14 3.0600 1.000 2.691030
0.082916 -0.000665
Sum of electronic and zero-point Energies= -3453.810946
Sum of electronic and thermal Energies= -3453.801353
Sum of electronic and thermal Enthalpies= -3453.800408
Sum of electronic and thermal Free Energies= -3453.847766

3-ClIII-CCH (in Acetonitrile)
C -1.69589 2.35502 -0.00003
C -2.10761 1.03201 -0.00015
C -1.17257 0.00432 -0.00015
C 0.14343 0.37823 -0.00004
C 0.62166 1.66707 0.00017
C -0.34206 2.66956 0.00017
H -2.4289 3.14979 -0.00002
H -3.15073 0.74392 -0.00027
H 1.67442 1.90072 0.00036
H -0.01981 3.70145 0.00034
C -1.56429 -1.47462 0.
O -0.53386 -2.21757 0.00039
O -2.743 -1.75971 -0.00016
C 3.88073 0.21773 -0.00012
H 4.86863 0.61439 -0.00016
C 2.76798 -0.21989 -0.00021
Cl 1.299 -1.06286 0.
Sum of electronic and zero-point Energies= -956.323124
Sum of electronic and thermal Energies= -956.312604
Sum of electronic and thermal Enthalpies= -956.311660
Sum of electronic and thermal Free Energies= -956.360119

3-ClIII-CF3 (in Acetonitrile)
C 2.44253 2.20354 -0.00008
C 2.73832 0.8514 -0.00017
C 1.71806 -0.09057 -0.00006
C 0.42892 0.37706 0.00012
C 0.07369 1.71099 0.00027
C 1.1195 2.62617 0.00017

H	3.2392	2.93431	-0.00018
H	3.75205	0.47297	-0.0003
H	-0.94048	2.06858	0.0005
H	0.88162	3.68069	0.00029
C	2.0011	-1.5906	-0.00001
O	0.91869	-2.26091	0.00043
O	3.15259	-1.9673	-0.00032
C	-2.37581	-0.00652	-0.00012
F	-3.30983	-0.93302	-0.00027
F	-2.49765	0.74002	-1.07829
F	-2.49808	0.74007	1.07798
Cl	-0.80179	-0.98491	0.00019

Sum of electronic and zero-point Energies= -1217.271304
Sum of electronic and thermal Energies= -1217.259349
Sum of electronic and thermal Enthalpies= -1217.258404
Sum of electronic and thermal Free Energies= -1217.311098

3-ClIII-CH3 (in Acetonitrile)

C	1.84919	2.09564	-0.00026
C	2.03211	0.72259	-0.00036
C	0.94595	-0.1438	-0.0002
C	-0.303	0.43	0.00006
C	-0.54379	1.79014	0.00034
C	0.56625	2.62624	0.00012
H	2.70392	2.75794	-0.00043
H	3.01223	0.26343	-0.00053
H	-1.5312	2.22037	0.00075
H	0.41394	3.69656	0.00029
C	1.13934	-1.67296	-0.00004
O	0.03732	-2.28217	-0.00078
O	2.28626	-2.08358	0.00075
C	-3.1018	0.35593	-0.00026
H	-3.09138	0.95627	-0.90317
H	-3.94406	-0.32918	-0.00021
H	-3.09178	0.95685	0.90224
Cl	-1.68034	-0.75405	0.00029

Sum of electronic and zero-point Energies= -919.506800
Sum of electronic and thermal Energies= -919.496831
Sum of electronic and thermal Enthalpies= -919.495886
Sum of electronic and thermal Free Energies= -919.543280

3-ClIII-CHCH2 (in Acetonitrile)

C	-1.96308	2.22022	-0.0493
C	-2.25146	0.865	-0.02349
C	-1.23368	-0.08084	0.01137
C	0.04904	0.40546	0.03103
C	0.40015	1.73881	-0.01049
C	-0.64368	2.65518	-0.05026
H	-2.76551	2.94443	-0.07968
H	-3.26414	0.48313	-0.03635
H	1.42523	2.07416	-0.03176
H	-0.4131	3.71077	-0.08706
C	-1.52771	-1.59419	-0.03061
O	-0.47043	-2.27114	-0.13084
O	-2.69768	-1.9293	0.02143
C	2.76881	0.12227	0.42979
H	2.73725	0.63429	1.38012
Cl	1.34674	-0.87415	0.10734
C	3.78391	0.08216	-0.40663
H	4.70207	0.60112	-0.16667
H	3.73459	-0.46818	-1.33654

Sum of electronic and zero-point Energies= -957.575355
Sum of electronic and thermal Energies= -957.564522
Sum of electronic and thermal Enthalpies= -957.563578
Sum of electronic and thermal Free Energies= -957.612899

3-ClIII-Cl (in Acetonitrile)

C	2.0278	2.19672	0.00028
C	2.31752	0.84237	0.00031
C	1.27493	-0.07011	0.00006
C	-0.01814	0.39531	-0.00018
C	-0.35571	1.72898	-0.00019
C	0.70619	2.62819	0.00004
H	2.82881	2.92234	0.00047
H	3.33014	0.46178	0.00051
H	-1.3789	2.0641	-0.00031
H	0.48395	3.68624	0.00006
C	1.45108	-1.54821	0.00013
O	0.27554	-2.15135	-0.00057
O	2.51028	-2.10805	0.00076
Cl	-3.03498	0.25614	0.00006
Cl	-1.19871	-0.96783	-0.00035

Sum of electronic and zero-point Energies= -1339.807792
Sum of electronic and thermal Energies= -1339.798469
Sum of electronic and thermal Enthalpies= -1339.797525
Sum of electronic and thermal Free Energies= -1339.843365

3-ClIII-CN (in Acetonitrile)

C	1.68989	2.34818	0.00001
C	2.10028	1.02476	0.00001
C	1.15626	0.00697	-0.00002
C	-0.15921	0.38046	-0.00005
C	-0.63557	1.66987	-0.00002
C	0.33656	2.66541	0.
H	2.42377	3.14186	0.00003
H	3.14291	0.73568	0.00003
H	-1.68562	1.91794	-0.00001
H	0.01781	3.69826	0.00001
C	1.51119	-1.46777	-0.00001
O	0.44664	-2.18883	-0.00002
O	2.66659	-1.81485	0.00003
C	-2.76029	-0.17007	0.00005
N	-3.79167	0.33194	0.00001
Cl	-1.27634	-1.09028	0.

Sum of electronic and zero-point Energies= -972.418651
Sum of electronic and thermal Energies= -972.408518
Sum of electronic and thermal Enthalpies= -972.407574
Sum of electronic and thermal Free Energies= -972.455014

3-ClIII-F (in Acetonitrile)

C	-2.76402	0.46697	0.00001
C	-2.06275	-0.7276	-0.00029
C	-0.67736	-0.68528	-0.00038
C	-0.04573	0.53239	-0.00006
C	-0.69654	1.7456	0.00026
C	-2.08586	1.68317	0.00028
H	-3.84484	0.45857	0.00005
H	-2.55407	-1.69103	-0.00053
H	-0.15736	2.67756	0.0005
H	-2.64487	2.60869	0.00052
C	0.22919	-1.86104	-0.00088
O	1.49604	-1.44857	0.00044
O	-0.10976	-3.00725	0.00052
F	1.93432	2.08692	-0.00021
Cl	1.72473	0.34619	0.

Sum of electronic and zero-point Energies= -979.441321
Sum of electronic and thermal Energies= -979.432649
Sum of electronic and thermal Enthalpies= -979.431705
Sum of electronic and thermal Free Energies= -979.475522

3-ClIII-N3 (in Acetonitrile)

C	-2.04516	2.29126	-0.13982
C	-2.39899	0.9562	-0.25145
C	-1.4329	-0.02053	-0.06869
C	-0.14812	0.37153	0.2131
C	0.2524	1.68261	0.34274
C	-0.73398	2.64581	0.15719
H	-2.78963	3.06244	-0.27928
H	-3.40817	0.63688	-0.47499
H	1.26516	1.95343	0.59032
H	-0.46356	3.68832	0.25154
C	-1.69354	-1.49458	-0.14555
O	-0.59852	-2.16246	0.09474
O	-2.77727	-1.9537	-0.39289
N	2.48808	-0.01428	0.76096
N	3.10628	0.11611	-0.29807
N	3.71482	0.26333	-1.22607
Cl	0.96705	-1.03304	0.41146

Sum of electronic and zero-point Energies= -1043.769651
Sum of electronic and thermal Energies= -1043.758733
Sum of electronic and thermal Enthalpies= -1043.757789
Sum of electronic and thermal Free Energies= -1043.808023

3-ClIII-NH2 (in Acetonitrile)

C	2.18738	-1.75542	0.00004
C	2.14272	-0.36991	0.00006
C	0.92184	0.28734	0.00004
C	-0.22121	-0.47465	0.00001
C	-0.22785	-1.85378	-0.00004
C	1.009	-2.49031	-0.00002
H	3.13895	-2.26858	0.00005
H	3.03468	0.24294	0.00009
H	-1.13249	-2.44029	-0.00014
H	1.0398	-3.57101	-0.00006
C	0.78615	1.80046	0.00001
O	-0.44534	2.13743	0.0002
O	1.78108	2.49243	-0.00017
N	-2.95109	-0.76379	0.00004
H	-2.86586	-1.33438	0.83371
H	-2.86603	-1.3344	-0.83362
Cl	-1.76268	0.47947	-0.00007

Sum of electronic and zero-point Energies= -935.540022
Sum of electronic and thermal Energies= -935.530375
Sum of electronic and thermal Enthalpies= -935.529430
Sum of electronic and thermal Free Energies= -935.575482

3-ClIII-NHAc (in Acetonitrile)

C	-2.20102	2.42746	-0.04341
C	-2.67748	1.13069	-0.1454
C	-1.80054	0.06378	-0.0184
C	-0.47722	0.34055	0.21224
C	0.04929	1.60759	0.31478
C	-0.84932	2.66015	0.18342
H	-2.87867	3.26373	-0.14433
H	-3.71988	0.90381	-0.32553
H	1.09706	1.78657	0.4853

H	-0.47727	3.67277	0.2544
C	-2.21423	-1.38393	-0.12369
O	-1.18955	-2.15344	0.01461
O	-3.36694	-1.69256	-0.31126
C	2.89969	-0.01369	-0.33344
O	2.48253	0.11507	-1.454
C	4.33912	0.19584	0.06862
H	4.41969	0.73188	1.01319
H	4.81978	-0.77635	0.18384
H	4.84215	0.74827	-0.71847
N	2.05682	-0.35145	0.71221
H	2.43769	-0.61979	1.60784
Cl	0.54375	-1.15142	0.35148

Sum of electronic and zero-point Energies= -1088.161225
Sum of electronic and thermal Energies= -1088.147922
Sum of electronic and thermal Enthalpies= -1088.146978
Sum of electronic and thermal Free Energies= -1088.202910

3-ClIII-OCOCH3 (in Acetonitrile)

C	-2.83079	2.07059	-0.00048
C	-3.00386	0.69692	-0.00038
C	-1.88513	-0.12077	-0.00019
C	-0.63434	0.44805	-0.00009
C	-0.41481	1.81005	-0.00019
C	-1.54996	2.6133	-0.00038
H	-3.98035	0.23176	-0.00045
H	0.57513	2.22953	-0.00009
H	-1.42106	3.68689	-0.00046
C	-1.92422	-1.60853	-0.00008
O	-0.69333	-2.09259	0.00028
O	-2.92463	-2.26703	0.00006
C	3.15067	-0.02674	-0.00002
O	3.3373	-1.21464	-0.00035
O	1.93626	0.52803	0.00031
C	4.23929	1.01425	0.00063
H	4.1384	1.6493	-0.87804
H	4.14084	1.64513	0.88262
H	5.20503	0.51961	-0.00172
Cl	0.64157	-0.80592	0.0002
H	-3.6906	2.72551	-0.00064

Sum of electronic and zero-point Energies= -1108.029491
Sum of electronic and thermal Energies= -1108.016606
Sum of electronic and thermal Enthalpies= -1108.015662
Sum of electronic and thermal Free Energies= -1108.071555

3-ClIII-OH (in Acetonitrile)

C	-2.53839	1.19894	0.01154
C	-2.1807	-0.13999	0.02462
C	-0.83804	-0.48273	0.01043
C	0.09747	0.5209	-0.00975
C	-0.20624	1.86403	-0.03373
C	-1.55938	2.1863	-0.02029
H	-3.58203	1.48054	0.02079
H	-2.91063	-0.9382	0.04229
H	0.56024	2.61946	-0.07486
H	-1.84464	3.22905	-0.04002
C	-0.31069	-1.88573	0.00308
O	0.99225	-1.8708	-0.03838
O	-1.02498	-2.85394	0.02923
O	2.52119	1.47812	-0.0336
H	2.76247	1.65493	0.88333
Cl	1.78369	-0.09666	-0.02382

Sum of electronic and zero-point Energies= -955.408627
Sum of electronic and thermal Energies= -955.399407
Sum of electronic and thermal Enthalpies= -955.398462
Sum of electronic and thermal Free Energies= -955.443362

3-ClIII-OTs (in Acetonitrile)

C	4.69769	1.64741	0.17666
C	4.77194	0.26576	0.21334
C	3.61865	-0.46661	-0.02119
C	2.44253	0.19345	-0.27787
C	2.31827	1.56258	-0.33245
C	3.48732	2.27861	-0.09455
H	5.58189	2.24169	0.35832
H	5.69228	-0.26427	0.41714
H	1.38839	2.06512	-0.54327
H	3.43825	3.35825	-0.12171
C	3.53265	-1.94212	-0.02998
O	2.27168	-2.32214	-0.3065
O	4.41901	-2.71461	0.16447
O	-0.16308	0.40427	-0.87069
S	-0.90125	1.03494	0.34274
O	-0.9409	2.45066	0.13205
O	-0.31705	0.5213	1.54877
C	-2.53822	0.40309	0.1937
C	-3.46844	1.09089	-0.57098
C	-2.87156	-0.77924	0.8367
C	-4.7473	0.57489	-0.69762
H	-3.18928	2.02296	-1.04236
C	-4.15567	-1.28124	0.69897
H	-2.13753	-1.28137	1.45225
C	-5.10763	-0.61698	-0.07127
H	-5.48256	1.10964	-1.28564
H	-4.42722	-2.19843	1.20607
C	-6.49018	-1.18107	-0.23862
H	-6.76627	-1.80671	0.60837
H	-7.22929	-0.38773	-0.33885
H	-6.54352	-1.79733	-1.13806
Cl	1.11747	-0.99019	-0.53588

Sum of electronic and zero-point Energies= -1774.283591
Sum of electronic and thermal Energies= -1774.265653
Sum of electronic and thermal Enthalpies= -1774.264708
Sum of electronic and thermal Free Energies= -1774.332528

3-IIII-anion (in Acetonitrile)

C	-2.95991	-1.4275	0.00032
C	-2.64315	-0.08066	0.00035
C	-1.32421	0.38349	0.0002
C	-0.32444	-0.58288	0.00002
C	-0.62171	-1.94527	-0.00002
C	-1.93963	-2.37018	0.00013
H	-3.99597	-1.74373	0.00043
H	-3.4054	0.68707	0.00047
H	0.18255	-2.66811	-0.00017
H	-2.16181	-3.43022	0.0001
C	-1.13013	1.94076	0.00028
O	0.04196	2.33877	0.00029
O	-2.19304	2.58226	0.00023
I	1.74054	-0.14566	-0.00024

Sum of electronic and zero-point Energies= -717.293036
Sum of electronic and thermal Energies= -717.285477
Sum of electronic and thermal Enthalpies= -717.284532
Sum of electronic and thermal Free Energies= -717.327419

3-IIII-Br (in Acetonitrile)

C	-2.84973	2.43736	-0.00018
C	-3.12848	1.08062	-0.00021
C	-2.08288	0.16681	-0.00015
C	-0.79031	0.64666	-0.00005
C	-0.47047	1.98657	-0.00002
C	-1.53252	2.884	-0.00008
H	-3.65871	3.1544	-0.00023
H	-4.1398	0.69644	-0.00028
H	0.5542	2.32624	0.00006
H	-1.32066	3.94438	-0.00006
C	-2.32174	-1.31432	-0.00018
O	-1.20004	-2.01405	-0.00011
O	-3.42019	-1.79531	-0.00026
Br	2.65741	0.64971	0.0002
I	0.59574	-0.93797	0.00003

14 Br 14 3.0600 1.000 -2.657409
0.649711 -0.000197

14 Br 14 3.0600 1.000 -2.671866
0.648125 -0.000314

14 Br 14 3.0600 1.000 -2.669076
0.647738 0.000938

14 Br 14 3.0600 1.000 -2.668944
0.648162 -0.018306

14 Br 14 3.0600 1.000 -2.669055
0.648108 0.000365

14 Br 14 3.0600 1.000 -2.667615
0.648028 -0.000125

14 Br 14 3.0600 1.000 -2.667615
0.648028 -0.000125

Sum of electronic and zero-point Energies= -3291.318567
Sum of electronic and thermal Energies= -3291.308610
Sum of electronic and thermal Enthalpies= -3291.307666
Sum of electronic and thermal Free Energies= -3291.356687

3-IIII-CCH (in Acetonitrile)

C	-2.81945	2.04695	0.00003
C	-2.85462	0.66205	0.
C	-1.66725	-0.05922	-0.00001
C	-0.48258	0.64249	-0.00001
C	-0.40164	2.01931	0.00001
C	-1.60196	2.71957	0.00003
H	-3.7423	2.61028	0.00005
H	-3.78249	0.10608	-0.00002
H	0.54901	2.53199	-0.00001
H	-1.57965	3.80077	0.00006
C	-1.6519	-1.57187	-0.00002
O	-0.44612	-2.0595	0.00013
O	-2.68092	-2.19977	-0.00014
C	3.33959	1.85945	-0.00007
H	4.08337	2.62151	-0.00008
C	2.49851	1.00062	-0.00007
I	1.19502	-0.63231	0.00001

Sum of electronic and zero-point Energies= -793.806719
Sum of electronic and thermal Energies= -793.795766
Sum of electronic and thermal Enthalpies= -793.794822
Sum of electronic and thermal Free Energies= -793.845422

3-IIII-CF3 (in Acetonitrile)

C	2.88153	2.35469	-0.00004
C	3.12035	0.991	-0.00032

C 2.05295 0.10174 -0.00026
C 0.77371 0.61377 0.00012
C 0.49403 1.96718 0.00039
C 1.57763 2.83694 0.00031
H 3.71017 3.04907 -0.0001
H 4.11977 0.57725 -0.00065
H -0.5104 2.35807 0.00063
H 1.39236 3.90224 0.0005
C 2.27621 -1.3937 -0.00078
O 1.16304 -2.06952 0.00032
O 3.38982 -1.85242 0.00013
C -2.35358 0.53976 -0.00016
F -3.46821 -0.19593 -0.00051
F -2.38486 1.32646 -1.07654
F -2.38547 1.32628 1.07634
I -0.67783 -0.91869 0.00013
Sum of electronic and zero-point Energies= -1054.746028
Sum of electronic and thermal Energies= -1054.734449
Sum of electronic and thermal Enthalpies= -1054.733504
Sum of electronic and thermal Free Energies= -1054.785234

3-IIII-CH3 (in Acetonitrile)
C -3.12078 -1.42941 -0.00044
C -2.83035 -0.07481 -0.00038
C -1.50975 0.35671 -0.0002
C -0.51422 -0.59587 -0.00009
C -0.76483 -1.95563 -0.00013
C -2.09272 -2.36467 -0.00031
H -4.14941 -1.76274 -0.00057
H -3.60244 0.68293 -0.00047
H 0.02624 -2.68929 -0.00002
H -2.31846 -3.42232 -0.00034
C -1.16413 1.83841 -0.00011
O 0.10376 2.05365 0.00006
O -2.05632 2.65687 -0.00022
C 2.36858 -1.7299 0.0003
H 2.09977 -2.27661 -0.8978
H 3.4352 -1.51809 0.00082
H 2.09895 -2.27689 0.89798
I 1.43019 0.21339 0.00019
Sum of electronic and zero-point Energies= -756.967325
Sum of electronic and thermal Energies= -756.956959
Sum of electronic and thermal Enthalpies= -756.956015
Sum of electronic and thermal Free Energies= -757.004888

3-IIII-CHCH2 (in Acetonitrile)
C -2.90267 2.01246 -0.02678
C -2.919 0.62754 -0.06393
C -1.72632 -0.08473 -0.03041
C -0.54953 0.62635 0.04385
C -0.49017 2.00632 0.07224
C -1.69463 2.69796 0.03739
H -3.8317 2.56508 -0.05426
H -3.83853 0.0608 -0.12359
H 0.44923 2.53809 0.10578
H -1.683 3.77922 0.05645
C -1.70609 -1.60352 -0.09859
O -0.51201 -2.08621 -0.10194
O -2.75035 -2.21307 -0.14867
C 2.47806 1.07471 0.30607
H 2.44572 1.60469 1.2502
C 3.35852 1.36312 -0.63664
H 4.08817 2.15322 -0.49899
H 3.39125 0.82677 -1.57751
I 1.1696 -0.59349 0.09863
Sum of electronic and zero-point Energies= -795.034600
Sum of electronic and thermal Energies= -795.023275
Sum of electronic and thermal Enthalpies= -795.022331
Sum of electronic and thermal Free Energies= -795.074034

3-IIII-Cl (in Acetonitrile)
C -2.98615 1.88739 0.
C -2.94254 0.50291 0.00017
C -1.71279 -0.14184 0.00007
C -0.56712 0.62511 -0.00003
C -0.56674 2.00325 -0.00023
C -1.80872 2.62834 -0.00023
H -3.93963 2.3968 0.00007
H -3.83712 -0.10535 0.00031
H 0.3518 2.57025 -0.00038
H -1.85025 3.70891 -0.00044
C -1.59606 -1.63736 -0.00014
O -0.33958 -2.0517 -0.00032
O -2.54882 -2.36512 0.00059
Cl 2.63251 1.34053 0.0004
I 1.14548 -0.58928 -0.00012
Sum of electronic and zero-point Energies= -1177.318946
Sum of electronic and thermal Energies= -1177.309213
Sum of electronic and thermal Enthalpies= -1177.308269
Sum of electronic and thermal Free Energies= -1177.356093

3-IIII-CN (in Acetonitrile)
C 2.87686 1.94413 0.00002
C 2.85766 0.55912 0.00002
C 1.64091 -0.11108 0.00002
C 0.48589 0.63885 0.00002
C 0.45735 2.01737 0.00003
C 1.68744 2.66513 0.00002
H 3.8213 2.47012 0.00002
H 3.76273 -0.03319 0.00001
H -0.46697 2.57648 0.00003
H 1.70825 3.74617 0.00002
C 1.5596 -1.61593 -0.00004
O 0.32226 -2.05558 0.00004
O 2.54437 -2.30307 -0.00017
C -2.45236 1.15048 0.00003
N -3.13458 2.0757 -0.00021
I -1.21691 -0.60205 0.00003
Sum of electronic and zero-point Energies= -809.919388
Sum of electronic and thermal Energies= -809.908898
Sum of electronic and thermal Enthalpies= -809.907954
Sum of electronic and thermal Free Energies= -809.957059

3-IIII-F (in Acetonitrile)
C 3.24318 -1.06652 -0.00016
C 2.79634 0.24517 -0.00019
C 1.43077 0.49598 -0.0001
C 0.56322 -0.57441 0.00002
C 0.96613 -1.89379 0.00006
C 2.33684 -2.12377 -0.00003
H 4.30431 -1.27322 -0.00022
H 3.47531 1.08721 -0.00029
H 0.24591 -2.69803 0.00016
H 2.69865 -3.14287 -0.00001
C 0.85762 1.88171 -0.00013
O -0.46946 1.88407 -0.00003
O 1.53265 2.87182 -0.00026
F -1.93075 -1.84671 0.
I -1.41543 0.05309 0.00011
Sum of electronic and zero-point Energies= -816.957622
Sum of electronic and thermal Energies= -816.948379
Sum of electronic and thermal Enthalpies= -816.947435
Sum of electronic and thermal Free Energies= -816.993491

3-IIII-N3 (in Acetonitrile)
C -2.68504 2.34427 0.0158
C -2.89408 0.98711 -0.16858
C -1.81585 0.11451 -0.10121
C -0.56468 0.63389 0.14743
C -0.31526 1.976 0.343
C -1.40732 2.83291 0.27112
H -3.51917 3.03002 -0.03553
H -3.87452 0.5737 -0.36371
H 0.67781 2.34229 0.55658
H -1.25365 3.893 0.41909
C -1.96833 -1.37 -0.28115
O -0.82435 -2.01518 -0.16776
O -3.0294 -1.8863 -0.50079
N 2.41072 0.57402 0.61449
N 2.91025 1.06267 -0.39209
N 3.40824 1.54969 -1.27124
I 0.89808 -0.86875 0.20293
Sum of electronic and zero-point Energies= -881.272966
Sum of electronic and thermal Energies= -881.261661
Sum of electronic and thermal Enthalpies= -881.260717
Sum of electronic and thermal Free Energies= -881.312435

3-IIII-NH2 (in Acetonitrile)
C 3.18552 -1.29708 -0.00002
C 2.83671 0.04421 -0.00002
C 1.49649 0.40753 0.
C 0.54316 -0.58701 0.00002
C 0.85446 -1.93173 0.00002
C 2.20023 -2.27883 0.
H 4.22782 -1.58455 -0.00004
H 3.57593 0.83414 -0.00003
H 0.08698 -2.6923 0.00005
H 2.47701 -3.32433 0.00002
C 1.05304 1.85239 0.
O -0.24187 1.9736 -0.00004
O 1.85912 2.74964 0.00003
N -2.33375 -1.67612 -0.00001
H -2.15463 -2.22894 0.82823
H -2.15462 -2.22881 -0.82834
I -1.42789 0.14934 0.
Sum of electronic and zero-point Energies= -773.024100
Sum of electronic and thermal Energies= -773.014085
Sum of electronic and thermal Enthalpies= -773.013141
Sum of electronic and thermal Free Energies= -773.060800

3-IIII-NHAc (in Acetonitrile)
C -2.38148 2.79098 0.07004
C -2.88402 1.51049 -0.0918
C -2.01967 0.424 -0.05016
C -0.67826 0.65863 0.15699
C -0.13942 1.9178 0.31593

C -1.01927 2.99188 0.27085
H -3.04856 3.64115 0.03634
H -3.93591 1.31733 -0.25392
H 0.91924 2.0658 0.46251
H -0.63204 3.99477 0.38785
C -2.50159 -0.99241 -0.22916
O -1.52668 -1.86497 -0.17357
O -3.6645 -1.24888 -0.40183
C 2.91575 0.52334 -0.38485
O 2.47969 0.65095 -1.50666
C 4.2772 1.03487 0.03202
H 4.19917 1.6696 0.91471
H 4.92643 0.19442 0.27804
H 4.70854 1.59735 -0.78945
N 2.19139 -0.09588 0.60074
H 2.59106 -0.15391 1.52511
I 0.4379 -1.11528 0.17644
Sum of electronic and zero-point Energies= -925.653750
Sum of electronic and thermal Energies= -925.640193
Sum of electronic and thermal Enthalpies= -925.639249
Sum of electronic and thermal Free Energies= -925.696233

3-IIII-OCOCH3 (in Acetonitrile)
C -3.11341 2.30593 0.0003
C -3.28272 0.9316 0.00031
C -2.16564 0.10679 0.00011
C -0.91084 0.68027 -0.00009
C -0.70481 2.04715 -0.00011
C -1.83502 2.85554 0.00009
H -3.97676 2.9566 0.00045
H -4.26029 0.4685 0.00047
H 0.28882 2.46487 -0.00026
H -1.71024 3.92985 0.00008
C -2.28096 -1.38923 0.00008
O -1.10221 -1.98951 0.00006
O -3.33443 -1.96242 0.00052
C 3.16005 0.42152 0.00059
O 3.43922 -0.7571 0.00152
O 1.90835 0.84666 -0.00093
C 4.17452 1.53127 0.00009
H 4.02533 2.15795 -0.87783
H 4.02897 2.15459 0.88105
H 5.17434 1.11051 -0.00251
I 0.58293 -0.77903 -0.00036
Sum of electronic and zero-point Energies= -945.540726
Sum of electronic and thermal Energies= -945.527309
Sum of electronic and thermal Enthalpies= -945.526364
Sum of electronic and thermal Free Energies= -945.584293

3-IIII-OH (in Acetonitrile)
C 3.21291 -1.18583 0.00366
C 2.8179 0.14243 0.01713
C 1.46428 0.45182 0.01102
C 0.55258 -0.58065 -0.004
C 0.90511 -1.91437 -0.02568
C 2.26443 -2.20434 -0.02037
H 4.26494 -1.43518 0.00721
H 3.52916 0.95737 0.02996
H 0.15333 -2.68882 -0.06022
H 2.58286 -3.23772 -0.03927
C 0.95436 1.86729 0.00991
O -0.35804 1.92897 -0.01953
O 1.69388 2.81489 0.03078
O -2.08964 -1.78766 -0.03218
H -2.25856 -2.09372 0.86343
I -1.4202 0.1017 -0.01101
Sum of electronic and zero-point Energies= -792.912867
Sum of electronic and thermal Energies= -792.903168
Sum of electronic and thermal Enthalpies= -792.902224
Sum of electronic and thermal Free Energies= -792.949122

3-IIII-OTs (in Acetonitrile)
C 4.43131 2.26789 0.10724
C 4.68256 0.9094 0.20484
C 3.63105 0.01336 0.06606
C 2.36558 0.51027 -0.16449
C 2.07225 1.85293 -0.27232
C 3.14068 2.73136 -0.12921
H 5.24192 2.9748 0.2157
H 5.67304 0.51476 0.38703
H 1.07245 2.21862 -0.45643
H 2.95284 3.79363 -0.20287
C 3.82458 -1.46772 0.15505
O 2.68868 -2.14484 -0.01064
O 4.88376 -1.98901 0.35021
O -0.5237 0.34625 -0.73399
S -1.28695 1.12485 0.37599
O -1.2793 2.50932 0.01667
O -0.76311 0.72469 1.65055
C -2.93049 0.51419 0.21882
C -3.77434 1.07234 -0.73238
C -3.35495 -0.52043 1.03565
C -5.05684 0.57273 -0.86603
H -3.42429 1.892 -1.34456
C -4.64578 -1.00786 0.88886
H -2.68694 -0.92106 1.78585
C -5.51071 -0.4728 -0.06047
H -5.72321 1.00226 -1.60371
H -4.98756 -1.81229 1.52743
C -6.91454 -0.9868 -0.21011
H -7.07248 -1.88626 0.38138
H -7.6346 -0.23548 0.11747
H -7.13515 -1.21695 -1.25243
I 0.99061 -1.05332 -0.32457
Sum of electronic and zero-point Energies= -1611.796354
Sum of electronic and thermal Energies= -1611.777170
Sum of electronic and thermal Enthalpies= -1611.776226
Sum of electronic and thermal Free Energies= -1611.847412

4-BrIII-anion (in Acetonitrile)
C -0.03296 -3.03534 -0.00004
C -0.90423 -1.96465 0.00001
C -0.45517 -0.63985 0.0001
C 0.91532 -0.43159 0.0001
C 1.80849 -1.50457 0.00008
C 1.33872 -2.80331 0.00001
H -0.41726 -4.04723 -0.0001
H -1.97476 -2.12193 0.00006
H 2.87022 -1.30225 0.0001
H 2.04005 -3.62794 0.
Br 1.73088 1.29062 -0.00002
O -1.20969 1.89159 0.00204
S -1.81387 0.57952 0.
O -2.52505 0.23869 -1.21806
O -2.52755 0.23612 1.21589
11 Br 11 3.0600 1.000 1.730883 -
1.290622 0.000020
11 Br 11 3.0600 1.000 1.713535 -
1.330457 0.000034
11 Br 11 3.0600 1.000 1.727188 -
1.295978 0.000113
11 Br 11 3.0600 1.000 1.746463 -
1.276323 0.000474
11 Br 11 3.0600 1.000 1.777406 -
1.242529 0.002503
11 Br 11 3.0600 1.000 1.795061 -
1.221983 0.003390
11 Br 11 3.0600 1.000 1.763586 -
1.248712 0.003509
11 Br 11 3.0600 1.000 1.689035 -
1.296548 0.005799
11 Br 11 3.0600 1.000 1.642142 -
1.347831 0.003968
11 Br 11 3.0600 1.000 -0.576680 -
1.835178 0.009750
11 Br 11 3.0600 1.000 0.744307 -
1.888474 0.006987
11 Br 11 3.0600 1.000 0.883180 -
1.835490 -0.008457
11 Br 11 3.0600 1.000 1.653690 -
1.349054 0.003029
11 Br 11 3.0600 1.000 0.799983 -
1.871919 0.011930
11 Br 11 3.0600 1.000 1.416640 -
1.547897 0.027667
11 Br 11 3.0600 1.000 1.415485 -
1.545123 0.018911
11 Br 11 3.0600 1.000 1.226431 -
1.659064 0.002617
11 Br 11 3.0600 1.000 1.226820 -
1.660587 0.003653
11 Br 11 3.0600 1.000 1.244818 -
1.648240 0.002234
11 Br 11 3.0600 1.000 1.230723 -
1.655412 0.000326
11 Br 11 3.0600 1.000 1.321472 -
1.597248 0.000727
11 Br 11 3.0600 1.000 1.319614 -
1.598600 0.000813
11 Br 11 3.0600 1.000 1.306196 -
1.607507 0.000437
11 Br 11 3.0600 1.000 1.307016 -
1.606980 0.000213
11 Br 11 3.0600 1.000 1.308147 -
1.606271 0.000059
11 Br 11 3.0600 1.000 1.309001 -
1.605713 -0.000028
11 Br 11 3.0600 1.000 1.309001 -
1.605713 -0.000028
Sum of electronic and zero-point Energies= -3429.174825
Sum of electronic and thermal Energies= -3429.165518
Sum of electronic and thermal Enthalpies= -3429.164574
Sum of electronic and thermal Free Energies= -3429.211058

4-BrIII-Br (in Acetonitrile)
C -1.73448 3.03455 -0.00008

C -2.34105 1.79113 -0.0001
C -1.55996 0.64271 -0.00004
C -0.19406 0.7816 0.00004
C 0.45064 1.99943 0.00006
C -0.34916 3.13487 -0.00001
H -2.34077 3.92923 -0.00013
H -3.41797 1.68502 -0.00015
H 1.52496 2.08236 0.00012
H 0.12713 4.10519 0.00001
Br 0.79291 -0.92176 0.00011
O -1.08176 -1.82188 0.00026
S -2.33535 -0.95715 -0.00008
O -3.05502 -1.07363 1.22755
O -3.05449 -1.0738 -1.228
Br 3.01769 -0.0222 0.
11 Br 11 3.0600 1.000 -0.792906 -
0.921760 -0.000107
16 Br 16 3.0600 1.000 -3.017687 -
0.022200 -0.000001
11 Br 11 3.0600 1.000 -0.860965 -
0.947171 -0.000115
16 Br 16 3.0600 1.000 -3.075178 -
0.050428 0.000040
11 Br 11 3.0600 1.000 -0.848906 -
0.902628 -0.000284
16 Br 16 3.0600 1.000 -3.054762 -
0.035127 0.000359
11 Br 11 3.0600 1.000 -0.822405 -
0.913563 0.002270
16 Br 16 3.0600 1.000 -3.011560 -
0.026333 -0.001701
11 Br 11 3.0600 1.000 -0.829449 -
0.904514 -0.049105
16 Br 16 3.0600 1.000 -3.018255 -
0.023705 0.036912
11 Br 11 3.0600 1.000 -0.830904 -
0.902266 0.001394
16 Br 16 3.0600 1.000 -3.018903 -
0.022485 -0.000972
11 Br 11 3.0600 1.000 -0.833382 -
0.903033 -0.001323
16 Br 16 3.0600 1.000 -3.022127 -
0.026561 0.001051
11 Br 11 3.0600 1.000 -0.831491 -
0.902996 -0.001696
16 Br 16 3.0600 1.000 -3.021624 -
0.025511 0.001312
11 Br 11 3.0600 1.000 -0.831777 -
0.903634 -0.002782
16 Br 16 3.0600 1.000 -3.021568 -
0.025635 0.002137
11 Br 11 3.0600 1.000 -0.831620 -
0.904058 -0.005580
16 Br 16 3.0600 1.000 -3.021265 -
0.025737 0.004268
11 Br 11 3.0600 1.000 -0.830536 -
0.900084 -0.012091
16 Br 16 3.0600 1.000 -3.021352 -
0.024938 0.009168
11 Br 11 3.0600 1.000 -0.831103 -
0.901111 -0.026733
16 Br 16 3.0600 1.000 -3.022408 -
0.025616 0.020213
11 Br 11 3.0600 1.000 -0.829228 -
0.904902 -0.073949
16 Br 16 3.0600 1.000 -3.020807 -
0.025614 0.055629
11 Br 11 3.0600 1.000 -0.846378 -
0.883919 -0.129426
16 Br 16 3.0600 1.000 -3.020866 -
0.030228 0.096914
11 Br 11 3.0600 1.000 -0.845932 -
0.895347 -0.218729
16 Br 16 3.0600 1.000 -2.955910 -
0.020939 0.167802
11 Br 11 3.0600 1.000 -0.830653 -
0.902839 -0.164786
16 Br 16 3.0600 1.000 -2.973107 -
0.018933 0.125929
11 Br 11 3.0600 1.000 -0.803722 -
0.891005 -0.220117
16 Br 16 3.0600 1.000 -2.969700 -
0.009677 0.165957
11 Br 11 3.0600 1.000 -0.821279 -
0.873125 -0.238915
16 Br 16 3.0600 1.000 -2.984056 -
0.017376 0.176451
11 Br 11 3.0600 1.000 -0.834304 -
0.879630 -0.224727
16 Br 16 3.0600 1.000 -2.979384 -
0.021039 0.167140
11 Br 11 3.0600 1.000 -0.822390 -
0.886854 -0.206694
16 Br 16 3.0600 1.000 -2.984637 -
0.016285 0.153375
11 Br 11 3.0600 1.000 -0.801679 -
0.881580 -0.229079
16 Br 16 3.0600 1.000 -2.970126 -
0.012294 0.171997
11 Br 11 3.0600 1.000 -0.802483 -
0.872720 -0.286969
16 Br 16 3.0600 1.000 -2.945408 -
0.006791 0.219008
11 Br 11 3.0600 1.000 -0.800852 -
0.871539 -0.258226
16 Br 16 3.0600 1.000 -2.959286 -
0.012902 0.193798
11 Br 11 3.0600 1.000 -0.797981 -
0.872797 -0.262914
16 Br 16 3.0600 1.000 -2.954689 -
0.012425 0.197423
11 Br 11 3.0600 1.000 -0.797847 -
0.870931 -0.267055
16 Br 16 3.0600 1.000 -2.953260 -
0.011251 0.200130
11 Br 11 3.0600 1.000 -0.807579 -
0.890420 -0.166106
16 Br 16 3.0600 1.000 -2.985851 -
0.019081 0.125178
11 Br 11 3.0600 1.000 -0.799703 -
0.875207 -0.250819
16 Br 16 3.0600 1.000 -2.960084 -
0.012955 0.188138
11 Br 11 3.0600 1.000 -0.793564 -
0.863089 -0.296840
16 Br 16 3.0600 1.000 -2.939899 -
0.007195 0.221770
11 Br 11 3.0600 1.000 -0.798693 -
0.872452 -0.260590
16 Br 16 3.0600 1.000 -2.955950 -
0.011965 0.195586
11 Br 11 3.0600 1.000 -0.798312 -
0.871884 -0.263331
16 Br 16 3.0600 1.000 -2.954839 -
0.011697 0.197518
11 Br 11 3.0600 1.000 -0.798312 -
0.871933 -0.262774
16 Br 16 3.0600 1.000 -2.955011 -
0.011725 0.196943
11 Br 11 3.0600 1.000 -0.798312 -
0.871933 -0.262774
16 Br 16 3.0600 1.000 -2.955011 -
0.011725 0.196943
Sum of electronic and zero-point Energies= -6003.119123
Sum of electronic and thermal Energies= -6003.107943
Sum of electronic and thermal Enthalpies= -6003.106999
Sum of electronic and thermal Free Energies= -6003.158349

4-BrIII-CCH (in Acetonitrile)
C 1.08995 3.08229 0.01725
C 1.76104 1.86935 0.05492
C 1.04791 0.67911 0.00663
C -0.3156 0.77491 -0.08467
C -1.03578 1.94485 -0.12691
C -0.29535 3.12082 -0.0718
H 1.65028 4.00613 0.05192
H 2.84008 1.81337 0.11103
H -2.11204 1.96161 -0.20393
H -0.81536 4.06769 -0.10868
Br -1.25666 -0.97226 -0.19262
O 0.87627 -1.70684 -0.71535
S 1.84505 -0.9294 0.09592
O 3.12896 -0.7726 -0.51402
O 1.79724 -1.26849 1.49076
C -2.93242 -0.32537 0.19642
C -4.06216 -0.01701 0.44152
H -5.06572 0.26041 0.66536
11 Br 11 3.0600 1.000 -1.256663 -
0.972259 -0.192619
11 Br 11 3.0600 1.000 -1.317342 -
1.031029 -0.189258
11 Br 11 3.0600 1.000 -1.329677 -
0.984858 -0.246302
11 Br 11 3.0600 1.000 -1.334271 -
0.956251 -0.178061
11 Br 11 3.0600 1.000 -1.313085 -
0.983121 -0.117301
11 Br 11 3.0600 1.000 -1.312791 -
0.979510 -0.164625
11 Br 11 3.0600 1.000 -1.310268 -
0.977181 -0.191753
11 Br 11 3.0600 1.000 -1.308792 -
0.978090 -0.183020
11 Br 11 3.0600 1.000 -1.310392 -
0.978997 -0.179532
11 Br 11 3.0600 1.000 -1.309853 -

0.980092 -0.177412
11 Br 11 3.0600 1.000 -1.309939 -
0.979914 -0.177830
11 Br 11 3.0600 1.000 -1.309939 -
0.979914 -0.177830
Sum of electronic and zero-point Energies= -3505.628390
Sum of electronic and thermal Energies= -3505.616292
Sum of electronic and thermal Enthalpies= -3505.615348
Sum of electronic and thermal Free Energies= -3505.668171

4-BrIII-CF3 (in Acetonitrile)
C 1.79939 2.98495 -0.08346
C 2.34755 1.71319 -0.15901
C 1.53091 0.59861 -0.03938
C 0.19026 0.8161 0.15657
C -0.40324 2.05491 0.26111
C 0.43795 3.15414 0.12731
H 2.43918 3.85155 -0.17426
H 3.40923 1.5534 -0.29249
H -1.45457 2.19548 0.45178
H 0.01468 4.14561 0.2047
Br -0.8642 -0.8424 0.34508
O 1.24784 -1.70868 0.87177
C -2.63674 -0.08207 -0.20644
F -3.40748 -1.12978 -0.40465
F -2.53363 0.61746 -1.31176
F -3.15822 0.66756 0.7461
S 2.16764 -1.08236 -0.11005
O 1.90536 -1.50355 -1.45834
O 3.52851 -1.0103 0.32232
11 Br 11 3.0600 1.000 0.864198 -
0.842402 -0.345080
11 Br 11 3.0600 1.000 0.924750 -
0.873350 -0.353862
11 Br 11 3.0600 1.000 1.125103 -
0.195938 -0.954272
11 Br 11 3.0600 1.000 1.105384 -
0.230286 -0.932936
11 Br 11 3.0600 1.000 0.985805 -
0.540925 -0.765582
11 Br 11 3.0600 1.000 0.985221 -
0.619983 -0.718383
11 Br 11 3.0600 1.000 0.978266 -
0.648266 -0.715335
11 Br 11 3.0600 1.000 0.989376 -
0.634826 -0.751411
11 Br 11 3.0600 1.000 1.002639 -
0.599323 -0.788558
11 Br 11 3.0600 1.000 1.029164 -
0.510072 -0.855001
11 Br 11 3.0600 1.000 1.127602 -
0.267425 -0.965477
11 Br 11 3.0600 1.000 1.053658 -
0.581095 -0.929977
11 Br 11 3.0600 1.000 1.003072 -
0.633743 -0.858849
11 Br 11 3.0600 1.000 1.103711 -
0.174703 -0.974277
11 Br 11 3.0600 1.000 1.072114 -
0.395129 -0.935356
11 Br 11 3.0600 1.000 1.080266 -
0.379995 -0.940429
11 Br 11 3.0600 1.000 1.101891 -
0.319400 -0.980417
11 Br 11 3.0600 1.000 1.092834 -
0.347775 -0.963353
11 Br 11 3.0600 1.000 1.076423 -
0.394866 -0.936784
11 Br 11 3.0600 1.000 1.090507 -
0.332559 -0.965229
11 Br 11 3.0600 1.000 1.096446 -
0.334845 -0.969172
11 Br 11 3.0600 1.000 1.091910 -
0.341618 -0.961255
11 Br 11 3.0600 1.000 1.093337 -
0.337631 -0.963497
11 Br 11 3.0600 1.000 1.092903 -
0.338347 -0.962610
11 Br 11 3.0600 1.000 1.092903 -
0.338347 -0.962610
Sum of electronic and zero-point Energies= -3766.574295
Sum of electronic and thermal Energies= -3766.560546
Sum of electronic and thermal Enthalpies= -3766.559602
Sum of electronic and thermal Free Energies= -3766.617265

4-BrIII-CH3 (in Acetonitrile)
C -1.06689 3.03816 0.03847
C -1.68012 1.79376 0.06931
C -0.91811 0.63558 0.01112
C 0.44442 0.78647 -0.08177
C 1.10103 1.99756 -0.12932
C 0.3136 3.14155 -0.06095
H -1.66928 3.93489 0.08284
H -2.75511 1.68537 0.12775
H 2.17244 2.07977 -0.22947
H 0.79033 4.11092 -0.09894
Br 1.47186 -0.87676 -0.18227
O -0.82187 -1.69238 -0.93556
C 3.15698 -0.20687 0.53153
S -1.65708 -1.01278 0.06058
O -1.3946 -1.44641 1.41061
O -3.02741 -0.82108 -0.31319
H 2.92827 0.36146 1.42592
H 3.72206 -1.10346 0.76445
H 3.65477 0.38383 -0.22751
11 Br 11 3.0600 1.000 1.471862 -
0.876756 -0.182272
11 Br 11 3.0600 1.000 1.508528 -
0.974722 -0.176912
11 Br 11 3.0600 1.000 1.537523 -
0.863101 -0.225719
11 Br 11 3.0600 1.000 1.485520 -
0.917342 -0.112942
11 Br 11 3.0600 1.000 1.505489 -
0.899857 -0.185292
11 Br 11 3.0600 1.000 1.504738 -
0.904388 -0.182294
11 Br 11 3.0600 1.000 1.503171 -
0.908395 -0.178429
11 Br 11 3.0600 1.000 1.501690 -
0.909268 -0.176878
11 Br 11 3.0600 1.000 1.501390 -
0.908820 -0.176862
11 Br 11 3.0600 1.000 1.501390 -
0.908820 -0.176862
Sum of electronic and zero-point Energies= -3468.811301
Sum of electronic and thermal Energies= -3468.799696
Sum of electronic and thermal Enthalpies= -3468.798752
Sum of electronic and thermal Free Energies= -3468.850296

4-BrIII-CHCH2 (in Acetonitrile)
C -3.27859 0.63988 0.40329
C -2.46773 -0.47016 0.22639
C -1.10316 -0.33813 -0.01966
C -0.62581 0.9546 -0.07478
C -1.39513 2.09302 0.09706
C -2.74869 1.92197 0.34072
H -4.33537 0.50405 0.58836
H -2.86827 -1.47444 0.25976
H -0.95712 3.07889 0.03772
H -3.37967 2.78894 0.47641
Br 1.24125 1.31013 -0.49358
O 0.90739 -1.5165 -1.14369
C 2.19023 0.73695 1.08194
S -0.10922 -1.86037 -0.16279
O 0.41529 -2.0135 1.17916
O -1.0677 -2.84433 -0.57853
H 2.15304 1.48283 1.85692
C 2.72474 -0.46201 1.07811
H 3.24009 -0.79513 1.97058
H 2.63624 -1.13572 0.23636
11 Br 11 3.0600 1.000 -1.241247 -
1.310129 -0.493577
11 Br 11 3.0600 1.000 -1.594930 -
0.912499 -0.523590
11 Br 11 3.0600 1.000 -1.545052 -
0.836934 -0.584858
11 Br 11 3.0600 1.000 -1.341950 -
1.148912 -0.521167
11 Br 11 3.0600 1.000 -1.409965 -
1.078301 -0.537325
11 Br 11 3.0600 1.000 -1.445798 -
1.040015 -0.549970
11 Br 11 3.0600 1.000 -1.203340 -
1.375680 -0.465643
11 Br 11 3.0600 1.000 -1.386305 -
1.137635 -0.524308
11 Br 11 3.0600 1.000 -1.397767 -
1.114592 -0.534570
11 Br 11 3.0600 1.000 -1.395801 -
1.121689 -0.531546
11 Br 11 3.0600 1.000 -1.392359 -
1.127946 -0.529701
11 Br 11 3.0600 1.000 -1.393430 -
1.130924 -0.528503
11 Br 11 3.0600 1.000 -1.391831 -
1.134866 -0.527185
11 Br 11 3.0600 1.000 -1.389243 -
1.139789 -0.525919
11 Br 11 3.0600 1.000 -1.389673 -
1.139750 -0.526037
11 Br 11 3.0600 1.000 -1.389341 -
1.140930 -0.525615
11 Br 11 3.0600 1.000 -1.389341 -
1.140930 -0.525615
Sum of electronic and zero-point Energies= -3506.875306

Sum of electronic and thermal Energies= -3506.862894
Sum of electronic and thermal Enthalpies= -3506.861949
Sum of electronic and thermal Free Energies= -3506.915333

4-BrIII-Cl (in Acetonitrile)
C -1.26453 3.04777 -0.0002
C -1.8896 1.81326 -0.00044
C -1.12242 0.65594 -0.00027
C 0.24489 0.77509 0.00018
C 0.90764 1.98331 0.00045
C 0.12226 3.1289 0.00024
H -1.85789 3.95106 -0.00036
H -2.96786 1.72291 -0.00072
H 1.98295 2.04808 0.00079
H 0.61101 4.093 0.00046
Br 1.20526 -0.93703 0.00013
O -0.64714 -1.80741 0.00088
Cl 3.27496 -0.05781 -0.00037
S -1.9056 -0.93888 -0.00005
O -2.62235 -1.06082 1.22783
O -2.62133 -1.06173 -1.22841
11 Br 11 3.0600 1.000 -1.205260 -
0.937028 -0.000132
11 Br 11 3.0600 1.000 -1.222327 -
0.921097 -0.001842
11 Br 11 3.0600 1.000 -1.223994 -
0.936480 0.024303
11 Br 11 3.0600 1.000 -1.252223 -
0.943561 -0.108932
11 Br 11 3.0600 1.000 -1.245436 -
0.929486 -0.040138
11 Br 11 3.0600 1.000 -1.234739 -
0.924912 0.004747
11 Br 11 3.0600 1.000 -1.237257 -
0.923615 0.001366
11 Br 11 3.0600 1.000 -1.238216 -
0.923282 -0.000165
11 Br 11 3.0600 1.000 -1.238216 -
0.923282 -0.000165
Sum of electronic and zero-point Energies= -3889.114047
Sum of electronic and thermal Energies= -3889.103900
Sum of electronic and thermal Enthalpies= -3889.102956
Sum of electronic and thermal Free Energies= -3889.151043

4-BrIII-CN (in Acetonitrile)
C -1.00384 3.10683 -0.00741
C -1.70583 1.91151 -0.04003
C -1.01637 0.70797 -0.00092
C 0.34971 0.76723 0.07622
C 1.09956 1.91872 0.11177
C 0.38273 3.11034 0.06597
H -1.54088 4.04445 -0.03453
H -2.78635 1.88358 -0.0859
H 2.177 1.92057 0.1761
H 0.92646 4.04385 0.09764
Br 1.20981 -1.02482 0.17189
O -0.80738 -1.68585 0.65097
C 2.92624 -0.33597 -0.20425
N 3.99533 0.02266 -0.41531
S -1.8349 -0.88076 -0.09293
O -3.06837 -0.76141 0.61366
O -1.86447 -1.204 -1.48755
11 Br 11 3.0600 1.000 -1.209813 -
1.024817 -0.171885
11 Br 11 3.0600 1.000 -1.276085 -
1.046861 -0.184729
11 Br 11 3.0600 1.000 -1.324233 -
0.914670 -0.353213
11 Br 11 3.0600 1.000 -1.308404 -
0.955297 -0.231541
11 Br 11 3.0600 1.000 -1.268966 -
1.000026 -0.178257
11 Br 11 3.0600 1.000 -1.256839 -
1.007447 -0.159836
11 Br 11 3.0600 1.000 -1.261222 -
1.007736 -0.160642
11 Br 11 3.0600 1.000 -1.263735 -
1.007665 -0.159634
11 Br 11 3.0600 1.000 -1.263956 -
1.007888 -0.160555
11 Br 11 3.0600 1.000 -1.263956 -
1.007888 -0.160555
Sum of electronic and zero-point Energies= -3521.721864
Sum of electronic and thermal Energies= -3521.710075
Sum of electronic and thermal Enthalpies= -3521.709130
Sum of electronic and thermal Free Energies= -3521.761205

4-BrIII-F (in Acetonitrile)
C -0.74213 3.11979 0.00018
C -1.48753 1.95302 0.00029
C -0.82926 0.73136 -0.00017
C 0.54179 0.71717 -0.00059
C 1.32213 1.85439 -0.00067
C 0.64749 3.06835 -0.00034
H -1.2447 4.07658 0.00047
H -2.56916 1.97036 0.00063
H 2.39836 1.79819 -0.00095
H 1.2227 3.98356 -0.0005
Br 1.33754 -1.0455 -0.00059
O -0.50002 -1.75089 -0.00417
F 3.00093 -0.29895 0.00222
S -1.71575 -0.80043 0.0004
O -2.42741 -0.90106 1.23103
O -2.4341 -0.89845 -1.22653
11 Br 11 3.0600 1.000 1.337536 -
1.045503 -0.000593
11 Br 11 3.0600 1.000 1.354311 -
1.026307 -0.001298
11 Br 11 3.0600 1.000 1.351767 -
1.037931 0.009804
11 Br 11 3.0600 1.000 1.375263 -
1.047490 -0.062235
11 Br 11 3.0600 1.000 1.366438 -
1.036995 -0.028383
11 Br 11 3.0600 1.000 1.355980 -
1.030706 -0.000146
11 Br 11 3.0600 1.000 1.357261 -
1.030099 -0.001002
11 Br 11 3.0600 1.000 1.358595 -
1.029250 -0.001765
11 Br 11 3.0600 1.000 1.358794 -
1.029426 -0.002268
11 Br 11 3.0600 1.000 1.359049 -
1.029501 -0.002835
11 Br 11 3.0600 1.000 1.359755 -
1.029195 -0.003962
11 Br 11 3.0600 1.000 1.360902 -
1.028923 -0.006545
11 Br 11 3.0600 1.000 1.363483 -
1.028982 -0.019779
11 Br 11 3.0600 1.000 1.361637 -
1.030402 -0.016756
11 Br 11 3.0600 1.000 1.357311 -
1.033011 -0.022387
11 Br 11 3.0600 1.000 1.343975 -
1.038822 -0.051688
11 Br 11 3.0600 1.000 1.370494 -
0.974019 -0.046259
11 Br 11 3.0600 1.000 1.353180 -
0.971544 -0.089119
11 Br 11 3.0600 1.000 1.351408 -
1.039845 -0.053821
11 Br 11 3.0600 1.000 1.337322 -
1.019943 -0.071742
11 Br 11 3.0600 1.000 1.341267 -
0.998052 -0.086083
11 Br 11 3.0600 1.000 1.339730 -
1.001861 -0.084721
11 Br 11 3.0600 1.000 1.339784 -
1.001788 -0.084936
11 Br 11 3.0600 1.000 1.339710 -
1.002164 -0.084686
11 Br 11 3.0600 1.000 1.339762 -
1.001908 -0.084852
11 Br 11 3.0600 1.000 1.339762 -
1.001908 -0.084852
Sum of electronic and zero-point Energies= -3528.745450
Sum of electronic and thermal Energies= -3528.735094
Sum of electronic and thermal Enthalpies= -3528.734150
Sum of electronic and thermal Free Energies= -3528.782540

4-BrIII-N3 (in Acetonitrile)
C -1.31614 3.07104 0.07969
C -1.95069 1.84736 0.2397
C -1.22809 0.67997 0.0562
C 0.09863 0.77808 -0.28306
C 0.76857 1.96718 -0.45693
C 0.02771 3.12933 -0.2653
H -1.87476 3.98626 0.21632
H -3.0001 1.77696 0.4928
H 1.8081 1.99962 -0.74495
H 0.51279 4.08571 -0.40118
Br 1.00247 -0.94132 -0.54258
O -0.98587 -1.67529 -0.68769
S -1.91336 -0.9505 0.26713
O -1.65567 -1.30934 1.62839
O -3.25964 -0.9337 -0.20281
N 2.78941 -0.18148 -0.50309
N 3.59352 0.07643 1.69413
N 3.17288 -0.0643 0.67015
11 Br 11 3.0600 1.000 1.002474 -
0.941317 -0.542584
11 Br 11 3.0600 1.000 1.061378 -
0.999024 -0.535467
11 Br 11 3.0600 1.000 1.005633 -
1.070250 -0.469049

11 Br 11 3.0600 1.000 1.218821 -
0.494959 -0.756605
11 Br 11 3.0600 1.000 1.204974 -
0.488957 -0.550318
11 Br 11 3.0600 1.000 1.080739 -
0.875961 -0.524962
11 Br 11 3.0600 1.000 1.045042 -
0.966547 -0.490330
11 Br 11 3.0600 1.000 1.039946 -
0.984415 -0.483260
11 Br 11 3.0600 1.000 1.043299 -
0.980104 -0.485136
11 Br 11 3.0600 1.000 1.042870 -
0.979181 -0.483940
11 Br 11 3.0600 1.000 1.043225 -
0.978842 -0.483392
11 Br 11 3.0600 1.000 1.043036 -
0.979740 -0.482974
11 Br 11 3.0600 1.000 1.043410 -
0.978897 -0.483415
11 Br 11 3.0600 1.000 1.043184 -
0.979376 -0.483200
11 Br 11 3.0600 1.000 1.043184 -
0.979376 -0.483200
Sum of electronic and zero-point Energies= -3593.077050
Sum of electronic and thermal Energies= -3593.064502
Sum of electronic and thermal Enthalpies= -3593.063558
Sum of electronic and thermal Free Energies= -3593.118016

4-BrIII-NH2 (in Acetonitrile)
C 1.15836 3.01452 0.00619
C 1.74472 1.75736 0.01121
C 0.9446 0.62521 -0.01183
C -0.41667 0.80268 -0.04664
C -1.04635 2.02543 -0.04681
C -0.22358 3.14693 -0.01959
H 1.78201 3.8975 0.01945
H 2.81869 1.6272 0.02292
H -2.12145 2.10798 -0.06275
H -0.67698 4.12828 -0.02615
Br -1.47698 -0.8497 -0.11502
O 0.52608 -1.73346 -0.69632
S 1.60999 -1.03476 0.05263
O 2.86215 -1.0112 -0.63761
O 1.61402 -1.37195 1.44858
N -3.09847 -0.01327 0.30715
H -3.76699 -0.29895 -0.39978
H -3.39601 -0.37342 1.20731
11 Br 11 3.0600 1.000 -1.476982 -
0.849700 -0.115016
11 Br 11 3.0600 1.000 -1.516196 -
0.965228 -0.111084
11 Br 11 3.0600 1.000 -1.562687 -
0.832564 -0.172617
11 Br 11 3.0600 1.000 -1.570859 -
0.822808 0.038411
11 Br 11 3.0600 1.000 -1.542958 -
0.864540 -0.026645
11 Br 11 3.0600 1.000 -1.516416 -
0.902508 -0.118566
11 Br 11 3.0600 1.000 -1.519900 -
0.890224 -0.113462
11 Br 11 3.0600 1.000 -1.520720 -
0.890240 -0.110105
11 Br 11 3.0600 1.000 -1.520644 -
0.891567 -0.107565
11 Br 11 3.0600 1.000 -1.521916 -
0.892630 -0.104777
11 Br 11 3.0600 1.000 -1.522504 -
0.894716 -0.102918
11 Br 11 3.0600 1.000 -1.522040 -
0.894116 -0.103924
11 Br 11 3.0600 1.000 -1.522208 -
0.894271 -0.103622
11 Br 11 3.0600 1.000 -1.522208 -
0.894271 -0.103622
Sum of electronic and zero-point Energies= -3484.846310
Sum of electronic and thermal Energies= -3484.834971
Sum of electronic and thermal Enthalpies= -3484.834027
Sum of electronic and thermal Free Energies= -3484.885018

4-BrIII-NHAc (in Acetonitrile)
C -1.54241 3.1085 0.06629
C -2.25289 1.91731 0.0358
C -1.56579 0.71416 0.02283
C -0.19366 0.75023 0.04083
C 0.55388 1.90416 0.06001
C -0.1537 3.10154 0.07579
H -2.07361 4.05 0.06925
H -3.33408 1.89657 0.00698
H 1.63157 1.89162 0.03705
H 0.39498 4.03286 0.07795
Br 0.67871 -1.00422 -0.00858
O -1.29589 -1.6342 -0.73209
C 3.34011 -0.05758 -0.18656
O 3.13509 0.18646 -1.34653
C 4.69465 0.0186 0.47386
H 4.64289 0.54559 1.42571
H 5.05931 -0.99157 0.66208
H 5.37895 0.52707 -0.19727
S -2.36749 -0.88132 0.00102
O -3.57498 -0.7481 -0.74987
O -2.45778 -1.27879 1.37682
N 2.31259 -0.42212 0.6662
H 2.50415 -0.69321 1.61907
Sum of electronic and zero-point Energies= -3637.469284
Sum of electronic and thermal Energies= -3637.454257
Sum of electronic and thermal Enthalpies= -3637.453312
Sum of electronic and thermal Free Energies= -3637.514540

4-BrIII-OCOCH3 (in Acetonitrile)
C -2.17513 2.89633 -0.00006
C -2.61484 1.58466 -0.00008
C -1.68624 0.5528 -0.00008
C -0.34787 0.86082 -0.00002
C 0.12958 2.15803 -0.00002
C -0.81404 3.17627 -0.00003
H -2.89259 3.70464 -0.00007
H -3.66855 1.33932 -0.00009
H 1.18347 2.37431 -0.00002
H -0.47135 4.20156 -0.00002
Br 0.83503 -0.6835 -0.0001
O -0.84815 -1.80657 0.00013
C 3.48374 -0.17056 -0.00013
O 3.5246 -1.37015 -0.00022
O 2.32856 0.5142 -0.00017
C 4.67381 0.74661 0.00039
H 4.6399 1.38871 -0.87819
H 4.6425 1.38351 0.88288
H 5.58034 0.15093 -0.00251
S -2.22312 -1.13898 0.00009
O -2.91279 -1.37052 -1.22742
O -2.91269 -1.37026 1.22771
Sum of electronic and zero-point Energies= -3657.337727
Sum of electronic and thermal Energies= -3657.324873
Sum of electronic and thermal Enthalpies= -3657.323928
Sum of electronic and thermal Free Energies= -3657.378575

4-BrIII-OH (in Acetonitrile)
C -1.0426 3.05623 0.01013
C -1.68233 1.82481 0.02216
C -0.92132 0.66804 -0.00713
C 0.44644 0.7808 -0.05113
C 1.12281 1.97917 -0.06464
C 0.34374 3.13145 -0.03155
H -1.62863 3.96437 0.02727
H -2.76067 1.74217 0.04407
H 2.19988 2.01813 -0.11084
H 0.8341 4.09467 -0.05054
Br 1.41505 -0.90836 -0.11914
O -0.46526 -1.68626 -0.63879
O 3.03567 -0.12289 0.24916
H 3.14728 -0.14343 1.20708
S -1.60622 -0.97265 0.06159
O -2.8049 -0.99694 -0.7106
O -1.66797 -1.31442 1.45028
11 Br 11 3.0600 1.000 1.415048 -
0.908359 -0.119141
11 Br 11 3.0600 1.000 1.443184 -
1.017964 -0.110377
11 Br 11 3.0600 1.000 1.437984 -
0.977345 -0.041297
11 Br 11 3.0600 1.000 1.496932 -
0.913002 -0.080890
11 Br 11 3.0600 1.000 1.483057 -
0.909738 -0.096069
11 Br 11 3.0600 1.000 1.446704 -
0.941896 -0.093971
11 Br 11 3.0600 1.000 1.438986 -
0.936828 -0.093432
11 Br 11 3.0600 1.000 1.445588 -
0.933856 -0.092911
11 Br 11 3.0600 1.000 1.445254 -
0.934580 -0.092531
11 Br 11 3.0600 1.000 1.445223 -
0.934779 -0.092418
11 Br 11 3.0600 1.000 1.445223 -
0.934779 -0.092418
Sum of electronic and zero-point Energies= -3504.717320
Sum of electronic and thermal Energies= -3504.706465
Sum of electronic and thermal Enthalpies= -3504.705521
Sum of electronic and thermal Free Energies= -3504.754865

4-BrIII-OTs (in Acetonitrile)
C 4.38992 1.98561 0.35498
C 4.40184 0.59992 0.30176

C 3.20901 -0.07134 0.09331
C 2.04965 0.65295 -0.04636
C 1.99381 2.02645 -0.00943
C 3.2014 2.68603 0.19737
H 5.31223 2.52344 0.5228
H 5.31332 0.03081 0.42486
H 1.07378 2.57452 -0.12359
H 3.19807 3.76586 0.24408
Br 0.47093 -0.46603 -0.30898
O 1.617 -1.95886 0.33313
S 3.10022 -1.83064 -0.07726
O 3.27099 -2.12823 -1.46212
O 3.93304 -2.45958 0.88978
O -0.55218 1.0462 -0.92052
S -1.55303 1.72409 0.10152
O -1.94659 2.94326 -0.52024
O -0.93169 1.72772 1.39069
C -2.90822 0.60643 0.1294
C -3.83199 0.63913 -0.90905
C -3.02542 -0.30063 1.17014
C -4.87993 -0.26108 -0.89837
H -3.72714 1.36957 -1.69963
C -4.08537 -1.19709 1.16248
H -2.30674 -0.28903 1.97818
C -5.02063 -1.19259 0.13326
H -5.6082 -0.24241 -1.69942
H -4.18843 -1.90571 1.97388
C -6.16657 -2.16334 0.11959
H -6.17854 -2.77591 1.01843
H -7.11828 -1.63614 0.05011
H -6.09737 -2.8261 -0.7439
Sum of electronic and zero-point Energies= -4323.585776
Sum of electronic and thermal Energies= -4323.565363
Sum of electronic and thermal Enthalpies= -4323.564419
Sum of electronic and thermal Free Energies= -4323.638583

4-ClIII-anion (in Acetonitrile)
C 2.16 -1.84625 0.00002
C 0.78054 -1.69666 0.00004
C 0.18554 -0.43993 0.00002
C 1.01909 0.67467 0.
C 2.40161 0.53922 -0.00003
C 2.97446 -0.72324 -0.00001
H 2.59647 -2.8369 0.00004
H 0.11882 -2.55191 0.00006
H 3.01551 1.42944 -0.00006
H 4.05247 -0.82273 -0.00004
O -1.93792 0.37831 -1.22162
S -1.62833 -0.33627 -0.00001
O -2.04113 -1.72833 0.00016
O -1.93798 0.37862 1.22141
Cl 0.38111 2.28745 0.00002
Sum of electronic and zero-point Energies= -1315.181799
Sum of electronic and thermal Energies= -1315.172679
Sum of electronic and thermal Enthalpies= -1315.171735
Sum of electronic and thermal Free Energies= -1315.217369

4-ClIII-Br (in Acetonitrile)
C -1.5297 2.9621 0.12258
C -2.15731 1.72646 0.18863
C -1.4056 0.57381 0.04597
C -0.05141 0.69411 -0.15679
C 0.61127 1.89487 -0.24372
C -0.16199 3.0424 -0.09609
H -2.1111 3.86705 0.22868
H -3.226 1.63307 0.32885
H 1.67217 1.9605 -0.42053
H 0.32313 4.00582 -0.164
O -1.06448 -1.6905 -0.85369
S -2.08627 -1.07049 0.07566
O -1.94552 -1.5365 1.42034
O -3.38626 -1.02482 -0.51055
Br 2.92587 -0.29236 0.15044
Cl 0.80324 -0.90906 -0.35655
15 Br 15 3.0600 1.000 2.925874 -
0.292356 0.150442
15 Br 15 3.0600 1.000 2.974859 -
0.346198 0.161843
15 Br 15 3.0600 1.000 2.877338 -
0.308940 0.298507
15 Br 15 3.0600 1.000 2.910362 -
0.313997 0.259730
15 Br 15 3.0600 1.000 2.892907 -
0.322697 0.222224
15 Br 15 3.0600 1.000 2.908372 -
0.318011 0.196662
15 Br 15 3.0600 1.000 2.916280 -
0.313979 0.195264
15 Br 15 3.0600 1.000 2.919718 -
0.316846 0.190255
15 Br 15 3.0600 1.000 2.919670 -
0.316657 0.191286
15 Br 15 3.0600 1.000 2.919173 -
0.316639 0.192332
15 Br 15 3.0600 1.000 2.918728 -
0.316482 0.192556
15 Br 15 3.0600 1.000 2.918728 -
0.316482 0.192556
Sum of electronic and zero-point Energies= -3889.095944
Sum of electronic and thermal Energies= -3889.084779
Sum of electronic and thermal Enthalpies= -3889.083835
Sum of electronic and thermal Free Energies= -3889.135597

4-ClIII-CCH (in Acetonitrile)
C 0.29394 3.02851 -0.08779
C 1.17643 1.95859 -0.12733
C 0.70544 0.65617 -0.01544
C -0.64549 0.52717 0.13736
C -1.57883 1.53066 0.19139
C -1.07042 2.81991 0.06981
H 0.67226 4.03758 -0.17408
H 2.24455 2.09637 -0.23341
H -2.63425 1.34616 0.3215
H -1.7536 3.65643 0.10808
O 1.1119 -1.6127 0.95964
S 1.77701 -0.80242 -0.06368
O 1.58214 -1.29943 -1.40111
O 3.07981 -0.31633 0.27809
C -2.81064 -1.11089 -0.13189
C -3.96247 -1.11362 -0.44546
H -4.98791 -1.11606 -0.73303
Cl -1.22423 -1.24312 0.32312
Sum of electronic and zero-point Energies= -1391.622895
Sum of electronic and thermal Energies= -1391.610965
Sum of electronic and thermal Enthalpies= -1391.610021
Sum of electronic and thermal Free Energies= -1391.662912

4-ClIII-CF3 (in Acetonitrile)
C 2.57989 2.13707 -0.50287
C 2.45863 0.7537 -0.50206
C 1.31209 0.14718 -0.00719
C 0.33668 0.99847 0.4621
C 0.40372 2.37854 0.49025
C 1.56354 2.94959 -0.01034
H 3.48109 2.59176 -0.89131
H 3.24053 0.10865 -0.88042
H -0.40446 2.9774 0.88507
H 1.66957 4.0249 -0.01204
O 0.97839 -1.90447 1.45678
C -2.31197 0.11047 -0.3728
F -2.79659 -1.08003 -0.35162
F -1.64057 0.38997 -1.43197
F -3.24401 1.00364 -0.15331
S 1.10073 -1.65993 0.03788
O -0.1457 -1.81652 -0.69289
O 2.27775 -2.1627 -0.60939
Cl -1.14158 0.2502 1.12624
Sum of electronic and zero-point Energies= -1652.579391
Sum of electronic and thermal Energies= -1652.566142
Sum of electronic and thermal Enthalpies= -1652.565197
Sum of electronic and thermal Free Energies= -1652.620710

4-ClIII-CH3 (in Acetonitrile)
C 2.37147 1.83191 0.24901
C 0.98346 1.85914 0.23776
C 0.25313 0.70369 -0.00829
C 0.98725 -0.44254 -0.228
C 2.3667 -0.52162 -0.23255
C 3.06531 0.65073 0.01519
H 2.92075 2.74398 0.43893
H 0.42691 2.77005 0.41363
H 2.87528 -1.45518 -0.42772
H 4.14588 0.63665 0.01937
O -1.88593 -0.23957 1.00935
C -0.21552 -2.64045 1.08657
S -1.56559 0.71852 -0.03995
O -1.90245 2.08326 0.24958
O -1.85707 0.24325 -1.37182
H 0.75342 -2.77474 1.55265
H -0.8627 -1.90005 1.55141
H -0.71652 -3.5816 0.88325
Cl 0.10584 -1.95742 -0.56514
Sum of electronic and zero-point Energies= -1354.815767
Sum of electronic and thermal Energies= -1354.804424
Sum of electronic and thermal Enthalpies= -1354.803480
Sum of electronic and thermal Free Energies= -1354.853680

4-ClIII-CHCH2 (in Acetonitrile)
C -1.54383 2.69318 0.39714
C -1.83598 1.33601 0.3889
C -0.87399 0.40622 0.01516
C 0.35713 0.91738 -0.33155
C 0.70123 2.25429 -0.33856
C -0.28396 3.15596 0.03657
H -2.30813 3.40089 0.68808
H -2.81025 0.95769 0.66825

H	1.69048	2.57995	-0.62671
H	-0.063	4.21377	0.04343
O	-0.96748	-1.73832	-1.37088
C	2.40202	-0.73675	0.68478
S	-1.22735	-1.3774	0.00267
O	-0.23037	-1.87756	0.94015
O	-2.58822	-1.46217	0.44998
H	1.66623	-1.28217	1.26483
C	3.67064	-0.45295	0.8583
H	4.15144	-0.79548	1.76503
H	4.25885	0.09707	0.13803
Cl	1.63417	-0.23229	-0.84717

Sum of electronic and zero-point Energies= -1392.883052
Sum of electronic and thermal Energies= -1392.870795
Sum of electronic and thermal Enthalpies= -1392.869850
Sum of electronic and thermal Free Energies= -1392.923188

4-ClIII-Cl (in Acetonitrile)

C	0.55162	3.04938	-0.08619
C	1.38212	1.93907	-0.13444
C	0.82793	0.6758	-0.03179
C	-0.53334	0.56156	0.11444
C	-1.39399	1.63111	0.17583
C	-0.81829	2.89388	0.07048
H	0.97508	4.04101	-0.15997
H	2.45558	2.03039	-0.2323
H	-2.45729	1.51311	0.30165
H	-1.4614	3.76103	0.12133
O	0.78635	-1.63885	0.7835
Cl	-3.14478	-0.84726	-0.23555
S	1.76307	-0.83376	-0.06264
O	1.77973	-1.27433	-1.42152
O	2.99683	-0.62193	0.61893
Cl	-1.10938	-1.16622	0.26345

Sum of electronic and zero-point Energies= -1775.089673
Sum of electronic and thermal Energies= -1775.078882
Sum of electronic and thermal Enthalpies= -1775.077938
Sum of electronic and thermal Free Energies= -1775.127544

4-ClIII-CN (in Acetonitrile)

C	-0.11625	3.0318	0.06279
C	-1.06041	2.01649	0.09367
C	-0.65944	0.68844	0.00861
C	0.68227	0.48312	-0.11018
C	1.67796	1.42464	-0.15212
C	1.23682	2.74163	-0.05818
H	-0.43645	4.06223	0.12682
H	-2.12067	2.21872	0.17068
H	2.72656	1.18941	-0.25313
H	1.96905	3.53575	-0.08962
O	-1.04679	-1.62688	-0.81682
C	2.76436	-1.20711	0.13688
N	3.88264	-1.17009	0.38518
S	-1.80361	-0.7064	0.0569
O	-1.79549	-1.10508	1.43567
O	-3.02721	-0.22083	-0.49867
Cl	1.13793	-1.35088	-0.2595

Sum of electronic and zero-point Energies= -1407.712375
Sum of electronic and thermal Energies= -1407.700777
Sum of electronic and thermal Enthalpies= -1407.699832
Sum of electronic and thermal Free Energies= -1407.751177

4-ClIII-F (in Acetonitrile)

C	-1.30653	2.66808	0.00001
C	-0.01782	2.1626	0.
C	0.16592	0.78828	-0.00002
C	-0.93099	-0.03307	-0.00004
C	-2.23093	0.42774	-0.00002
C	-2.39751	1.8067	-0.00001
H	-1.46435	3.73716	0.00002
H	0.84817	2.81075	0.00001
H	-3.07151	-0.24458	-0.00003
H	-3.40191	2.20611	0.
O	1.26619	-1.42243	-0.00025
F	-2.22237	-2.20426	0.00009
S	1.7641	0.04017	0.00002
O	2.4133	0.35575	-1.22838
O	2.41306	0.35541	1.22864
Cl	-0.56282	-1.7968	-0.00005

Sum of electronic and zero-point Energies= -1414.714613
Sum of electronic and thermal Energies= -1414.705413
Sum of electronic and thermal Enthalpies= -1414.704469
Sum of electronic and thermal Free Energies= -1414.749536

4-ClIII-N3 (in Acetonitrile)

C	-0.97971	2.99902	0.08254
C	-1.71208	1.82298	0.13709
C	-1.06116	0.60362	0.03908
C	0.30379	0.61049	-0.10047
C	1.0744	1.74839	-0.16931
C	0.39921	2.96037	-0.0723
H	-1.48446	3.95142	0.16295
H	-2.78632	1.82344	0.26602
H	2.14263	1.72109	-0.30579
H	0.96931	3.87751	-0.11643
O	-0.76112	-1.7918	0.55421
S	-1.90811	-0.96757	0.05206
O	-2.98092	-0.86773	0.99019
O	-2.22356	-1.23809	-1.31859
N	2.66261	-0.52628	-0.82594
N	4.28144	-0.5532	0.88976
N	3.47876	-0.55118	0.11794
Cl	1.07688	-1.04506	-0.20108

Sum of electronic and zero-point Energies= -1479.062083
Sum of electronic and thermal Energies= -1479.049765
Sum of electronic and thermal Enthalpies= -1479.048821
Sum of electronic and thermal Free Energies= -1479.102328

4-ClIII-NH2 (in Acetonitrile)

C	-0.62101	2.89241	-0.00007
C	0.49047	2.06944	-0.00008
C	0.34681	0.68527	0.00002
C	-0.93402	0.19681	0.00013
C	-2.07712	0.96767	0.00014
C	-1.89793	2.34378	0.00004
H	-0.49635	3.96622	-0.00014
H	1.4947	2.47287	-0.00014
H	-3.06053	0.52875	0.00022
H	-2.76925	2.98345	0.00005
O	1.15029	-1.69157	0.00095
S	1.80526	-0.36899	-0.00004
O	2.48346	-0.05241	-1.22378
O	2.48455	-0.05112	1.22276
N	-2.81887	-1.75052	-0.0004
H	-3.07414	-2.27341	-0.83107
H	-3.07472	-2.2733	0.83016
Cl	-1.11539	-1.63639	0.00023

Sum of electronic and zero-point Energies= -1370.840418
Sum of electronic and thermal Energies= -1370.829264
Sum of electronic and thermal Enthalpies= -1370.828319
Sum of electronic and thermal Free Energies= -1370.879098

4-ClIII-NHAc (in Acetonitrile)

C	-1.42159	2.98268	0.13334
C	-2.12528	1.78601	0.13624
C	-1.44032	0.58423	0.05244
C	-0.07346	0.64853	-0.0297
C	0.67597	1.79979	-0.05636
C	-0.03596	2.99121	0.03411
H	-1.95776	3.91963	0.19308
H	-3.20568	1.75487	0.18457
H	1.7491	1.79096	-0.16697
H	0.50406	3.92703	0.00841
O	-1.30026	-1.67535	-0.95838
C	3.35953	-0.30932	-0.14224
O	3.26568	-0.01108	-1.29748
C	4.63982	-0.38969	0.64425
H	4.53133	0.0564	1.63171
H	4.91537	-1.43759	0.76742
H	5.4179	0.12067	0.08632
S	-2.21433	-1.03789	0.00826
O	-3.55118	-0.83384	-0.45977
O	-2.04414	-1.53622	1.34933
N	2.22527	-0.60596	0.62032
H	2.28444	-0.97588	1.55842
Cl	0.77533	-0.96569	-0.14349

Sum of electronic and zero-point Energies= -1523.459678
Sum of electronic and thermal Energies= -1523.444975
Sum of electronic and thermal Enthalpies= -1523.444031
Sum of electronic and thermal Free Energies= -1523.503368

4-ClIII-OCOCH3 (in Acetonitrile)

C	-2.09653	2.7841	0.07335
C	-2.50438	1.46019	0.13948
C	-1.55681	0.45764	0.03552
C	-0.23662	0.79951	-0.13202
C	0.21062	2.10039	-0.20793
C	-0.75512	3.09581	-0.10005
H	-3.54462	1.18674	0.25366
H	1.24997	2.34192	-0.34552
H	-0.44251	4.12869	-0.16219
O	-0.71586	-1.71789	-0.71644
C	3.41695	-0.32312	0.05153
O	3.432	-1.48608	-0.22128
O	2.27638	0.40884	0.08673
C	4.60407	0.52894	0.39779
H	4.68306	1.35369	-0.30849
H	4.47329	0.94897	1.39355
H	5.49752	-0.08519	0.36089
S	-1.92292	-1.27736	0.10276
O	-3.14414	-1.51968	-0.59089
O	-1.80808	-1.66661	1.47272
Cl	0.87425	-0.62253	-0.28096
H	-2.82775	3.57655	0.14729

Sum of electronic and zero-point Energies= -1543.313092
Sum of electronic and thermal Energies= -1543.298853

Sum of electronic and thermal Enthalpies= -1543.297909
Sum of electronic and thermal Free Energies= -1543.356322

4-ClIII-OH (in Acetonitrile)
C 1.36817 2.66055 0.05656
C 0.06526 2.18275 0.08014
C -0.15694 0.81848 0.01058
C 0.92809 -0.01625 -0.08024
C 2.23644 0.40765 -0.11114
C 2.4395 1.78242 -0.03897
H 1.55029 3.725 0.10348
H -0.78673 2.84692 0.13603
H 3.05882 -0.28384 -0.197
H 3.4513 2.16138 -0.07012
O -1.36006 -1.18388 -0.74256
O 2.08981 -2.34149 0.21617
H 2.09425 -2.44201 1.17772
S -1.7582 0.04012 0.03867
O -2.67499 0.87646 -0.66609
O -2.0183 -0.25866 1.41581
Cl 0.54049 -1.78839 -0.17982
Sum of electronic and zero-point Energies= -1390.698412
Sum of electronic and thermal Energies= -1390.687619
Sum of electronic and thermal Enthalpies= -1390.686674
Sum of electronic and thermal Free Energies= -1390.735923

4-ClIII-OTs (in Acetonitrile)
C 4.38849 1.01612 0.85548
C 3.91588 -0.28668 0.77884
C 2.71285 -0.52224 0.13861
C 2.03097 0.54035 -0.39638
C 2.45562 1.8446 -0.34824
C 3.66808 2.06426 0.29756
H 5.32968 1.216 1.34776
H 4.46719 -1.12141 1.18966
H 1.88323 2.65281 -0.77389
H 4.04699 3.07458 0.35796
O 1.23856 -1.74118 -1.38607
S 1.94044 -2.10294 -0.07958
O 2.93739 -3.10108 -0.27337
O 0.99675 -2.25331 0.98739
O -0.23909 1.67947 -1.12681
S -1.04326 1.98662 0.251
O -1.60268 3.27079 0.01208
O -0.13779 1.74822 1.3256
C -2.28908 0.75687 0.26309
C -3.49469 1.01182 -0.37791
C -2.04018 -0.45574 0.89411
C -4.46474 0.02581 -0.38454
H -3.66464 1.97223 -0.84464
C -3.02289 -1.43122 0.86637
H -1.09776 -0.6351 1.39469
C -4.24149 -1.20739 0.22916
H -5.41376 0.2148 -0.86962
H -2.83604 -2.38093 1.35025
C -5.28918 -2.28202 0.182
H -5.20064 -2.95629 1.03161
H -6.29095 -1.85569 0.17712
H -5.17785 -2.8768 -0.72654
Cl 0.4751 0.07567 -1.21626
Sum of electronic and zero-point Energies= -2209.559596
Sum of electronic and thermal Energies= -2209.539353
Sum of electronic and thermal Enthalpies= -2209.538409
Sum of electronic and thermal Free Energies= -2209.612327

4-IIII-anion (in Acetonitrile)
C 1.82747 2.7407 -0.00017
C 2.08822 1.38559 -0.0002
C 1.06307 0.4331 -0.00016
C -0.24432 0.89335 -0.00007
C -0.52025 2.26349 -0.00006
C 0.50935 3.18485 -0.00011
H 2.64493 3.45029 -0.00021
H 3.10551 1.01683 -0.00031
H -1.54826 2.59755 0.
H 0.2816 4.24322 -0.0001
O 0.52603 -2.14694 -0.00133
S 1.68464 -1.28147 -0.00007
O 2.47395 -1.32236 -1.21648
O 2.4718 -1.323 1.21771
I -1.95384 -0.33722 0.00014
Sum of electronic and zero-point Energies= -1152.609832
Sum of electronic and thermal Energies= -1152.600240
Sum of electronic and thermal Enthalpies= -1152.599296
Sum of electronic and thermal Free Energies= -1152.647867

4-IIII-Br (in Acetonitrile)
C -1.98988 3.09718 -0.00014
C -2.55095 1.83235 -0.00015
C -1.72556 0.71472 -0.00008
C -0.36127 0.89564 0.00001
C 0.22998 2.14332 0.00003
C -0.60883 3.24961 -0.00005
H -2.6295 3.96846 -0.00019
H -3.62361 1.68987 -0.00022
H 1.30227 2.26339 0.0001
H -0.17068 4.23788 -0.00003
O -1.19538 -1.76135 0.00022
S -2.46301 -0.90416 -0.00013
O -3.17491 -1.04653 1.22829
O -3.17435 -1.04668 -1.22886
Br 3.03891 0.26902 0.00005
I 0.76536 -0.90319 0.00011
15 Br 15 3.0600 1.000 -3.038905
0.269015 -0.000050
15 Br 15 3.0600 1.000 -3.053020
0.269022 0.000286
15 Br 15 3.0600 1.000 -3.049076
0.274513 -0.002525
15 Br 15 3.0600 1.000 -3.047081
0.273522 0.046312
15 Br 15 3.0600 1.000 -3.046882
0.274669 -0.018415
15 Br 15 3.0600 1.000 -3.045316
0.272876 -0.020431
15 Br 15 3.0600 1.000 -3.045321
0.271726 0.001876
15 Br 15 3.0600 1.000 -3.046973
0.271646 0.001157
15 Br 15 3.0600 1.000 -3.046525
0.271343 0.001282
15 Br 15 3.0600 1.000 -3.046525
0.271343 0.001282
Sum of electronic and zero-point Energies= -3726.600608
Sum of electronic and thermal Energies= -3726.590027
Sum of electronic and thermal Enthalpies= -3726.589083
Sum of electronic and thermal Free Energies= -3726.639359

4-IIII-CCH (in Acetonitrile)
C 1.63939 3.06029 -0.00019
C 2.16487 1.78066 -0.00006
C 1.31461 0.67967 -0.00003
C -0.0414 0.90532 -0.00013
C -0.60273 2.16857 -0.00026
C 0.26269 3.25271 -0.00029
H 2.30304 3.9136 -0.00022
H 3.23324 1.60946 0.
H -1.67246 2.31349 -0.00034
H -0.14821 4.25269 -0.0004
O 0.76582 -1.78979 -0.00049
S 2.02324 -0.96275 0.00023
O 2.74979 -1.08295 -1.22603
O 2.74848 -1.0831 1.22725
C -2.9983 0.27849 0.00012
C -4.07487 0.80996 0.00026
H -5.02806 1.28518 0.0004
I -1.26713 -0.82902 -0.0001
Sum of electronic and zero-point Energies= -1229.098924
Sum of electronic and thermal Energies= -1229.087670
Sum of electronic and thermal Enthalpies= -1229.086726
Sum of electronic and thermal Free Energies= -1229.137344

4-IIII-CF3 (in Acetonitrile)
C 1.98315 3.06557 -0.00002
C 2.53033 1.79598 -0.00009
C 1.70011 0.67972 -0.00001
C 0.33861 0.87483 0.00016
C -0.24265 2.13226 0.00024
C 0.60347 3.23104 0.00016
H 2.63074 3.93101 -0.00007
H 3.60148 1.64301 -0.00019
H -1.309 2.28378 0.0004
H 0.17222 4.22229 0.00024
O 1.22152 -1.80686 0.00068
C -2.753 0.18115 -0.00021
F -3.68086 -0.77306 -0.0005
F -2.93492 0.93853 -1.07619
F -2.93548 0.93842 1.07576
S 2.45532 -0.94189 -0.00015
O 3.18176 -1.04108 -1.22766
O 3.18312 -1.04068 1.22658
I -0.83152 -0.8981 0.00023
Sum of electronic and zero-point Energies= -1490.037692
Sum of electronic and thermal Energies= -1490.023928
Sum of electronic and thermal Enthalpies= -1490.022984
Sum of electronic and thermal Free Energies= -1490.080504

4-IIII-CH3 (in Acetonitrile)
C -1.93392 2.83441 0.00001
C -2.26662 1.49225 0.00003
C -1.2713 0.51951 -0.00002
C 0.04162 0.93213 -0.00009
C 0.40791 2.26888 -0.00012
C -0.59952 3.22165 -0.00008
H -2.71371 3.5831 0.00003
H -3.29906 1.16816 0.00005

H 1.43891 2.58468 -0.00021
H -0.33366 4.26958 -0.00012
O -0.44599 -1.87941 -0.00084
C 3.16925 0.7986 0.00054
S -1.77218 -1.20546 0.00009
O -2.49374 -1.38632 1.22546
O -2.49533 -1.38604 -1.22437
H 3.14286 1.40339 0.9004
H 4.05115 0.16312 0.00077
H 3.14347 1.40359 -0.8992
I 1.53058 -0.57507 -0.00013
Sum of electronic and zero-point Energies= -1192.268565
Sum of electronic and thermal Energies= -1192.256922
Sum of electronic and thermal Enthalpies= -1192.255978
Sum of electronic and thermal Free Energies= -1192.307852

4-IIII-CHCH2 (in Acetonitrile)
C -1.9684 2.92138 0.00709
C -2.36874 1.59592 0.07962
C -1.4233 0.58228 0.01136
C -0.09998 0.93165 -0.13786
C 0.33566 2.24031 -0.20489
C -0.62444 3.24208 -0.12998
H -2.707 3.70935 0.05655
H -3.40967 1.31969 0.18274
H 1.3802 2.49222 -0.31142
H -0.31284 4.27578 -0.18758
O -0.82799 -1.77434 -0.63726
C 2.97705 0.54084 0.01985
S -1.89087 -1.13992 0.19015
O -1.72083 -1.41156 1.59161
O -3.20403 -1.2671 -0.36334
H 3.34658 1.06966 -0.84828
C 3.58227 0.5641 1.19361
H 4.48963 1.1403 1.33379
H 3.20453 0.02027 2.05024
I 1.27971 -0.6769 -0.28451
Sum of electronic and zero-point Energies= -1230.334434
Sum of electronic and thermal Energies= -1230.321790
Sum of electronic and thermal Enthalpies= -1230.320846
Sum of electronic and thermal Free Energies= -1230.375947

4-IIII-Cl (in Acetonitrile)
C -1.86249 2.98344 0.00002
C -2.31758 1.67649 0.00005
C -1.40142 0.63221 0.00004
C -0.05711 0.92621 -0.00002
C 0.42971 2.21849 -0.00006
C -0.49873 3.25058 -0.00005
H -2.57196 3.79883 0.00001
H -3.3748 1.44622 0.00007
H 1.4888 2.42406 -0.00012
H -0.14501 4.27211 -0.00008
O -0.65141 -1.78598 -0.00023
Cl 3.21206 0.56282 0.0001
S -1.99271 -1.04439 0.00003
O -2.68657 -1.25328 1.22868
O -2.68695 -1.25313 -1.22843
I 1.21371 -0.76574 -0.00004
Sum of electronic and zero-point Energies= -1612.601064
Sum of electronic and thermal Energies= -1612.590795
Sum of electronic and thermal Enthalpies= -1612.589851
Sum of electronic and thermal Free Energies= -1612.638667

4-IIII-CN (in Acetonitrile)
C -1.62365 3.06348 -0.00001
C -2.14859 1.78351 0.00003
C -1.29393 0.6866 0.00003
C 0.06147 0.91452 -0.00005
C 0.62333 2.17725 -0.00008
C -0.24746 3.25786 -0.00007
H -2.28809 3.91601 0.
H -3.21673 1.61108 0.00007
H 1.69147 2.33425 -0.00012
H 0.16231 4.25813 -0.00009
O -0.71148 -1.77698 -0.00022
C 2.99866 0.33001 -0.00006
N 3.9776 0.93111 0.00036
S -1.99159 -0.95705 0.00006
O -2.70412 -1.10135 1.22859
O -2.70455 -1.10123 -1.22824
I 1.25312 -0.84467 -0.00006
Sum of electronic and zero-point Energies= -1245.205605
Sum of electronic and thermal Energies= -1245.193726
Sum of electronic and thermal Enthalpies= -1245.192782
Sum of electronic and thermal Free Energies= -1245.246069

4-IIII-F (in Acetonitrile)
C -2.16763 2.66471 -0.00006
C -2.38824 1.29774 -0.00006
C -1.29957 0.43587 -0.00004
C -0.02915 0.96204 -0.00002
C 0.22562 2.32075 -0.00002
C -0.87197 3.17055 -0.00004
H -3.00966 3.34242 -0.00008
H -3.38904 0.88688 -0.00008
H 1.23716 2.69728 -0.00001
H -0.70863 4.23922 -0.00004
O -0.08179 -1.78582 0.00015
F 2.77755 1.04539 0.00034
S -1.55114 -1.32301 -0.00009
O -2.18292 -1.67386 1.22889
O -2.1825 -1.67382 -1.22931
I 1.51799 -0.44242 0.00004
Sum of electronic and zero-point Energies= -1252.239607
Sum of electronic and thermal Energies= -1252.229800
Sum of electronic and thermal Enthalpies= -1252.228856
Sum of electronic and thermal Free Energies= -1252.276199

4-IIII-N3 (in Acetonitrile)
C -1.74755 3.08777 -0.07383
C -2.28803 1.82473 0.11973
C -1.46164 0.71585 0.03341
C -0.12603 0.89573 -0.24885
C 0.44295 2.13595 -0.44689
C -0.39566 3.24137 -0.3529
H -2.38555 3.95819 -0.01261
H -3.33985 1.68183 0.32828
H 1.49129 2.24331 -0.68453
H 0.01646 4.22808 -0.51194
O -1.0169 -1.69425 -0.49104
S -2.05163 -0.93539 0.33857
O -1.86028 -1.17749 1.73505
O -3.35518 -1.06408 -0.22162
N 2.80057 0.1775 -0.409
N 3.64414 0.79378 1.69408
N 3.20887 0.47958 0.71304
I 0.99591 -0.891 -0.39408
Sum of electronic and zero-point Energies= -1316.559122
Sum of electronic and thermal Energies= -1316.546491
Sum of electronic and thermal Enthalpies= -1316.545546
Sum of electronic and thermal Free Energies= -1316.600532

4-IIII-NH2 (in Acetonitrile)
C 2.04537 2.76793 -0.00002
C 2.33245 1.41419 -0.00013
C 1.29635 0.48725 -0.00009
C 0.00103 0.95034 -0.00007
C -0.32146 2.29418 0.00024
C 0.72561 3.20494 0.00021
H 2.852 3.4877 0.00002
H 3.35262 1.05359 -0.00015
H -1.35251 2.61439 0.00062
H 0.50309 4.26302 0.00041
O 0.28796 -1.82992 0.00162
S 1.68164 -1.25666 -0.00017
O 2.36912 -1.51942 -1.22721
O 2.37204 -1.519 1.22532
N -2.97756 0.91408 -0.00117
H -3.55665 0.84061 -0.82758
H -3.55668 0.84205 0.82537
I -1.52857 -0.51245 0.00026
Sum of electronic and zero-point Energies= -1208.318633
Sum of electronic and thermal Energies= -1208.307945
Sum of electronic and thermal Enthalpies= -1208.307001
Sum of electronic and thermal Free Energies= -1208.356261

4-IIII-NHAc (in Acetonitrile)
C -1.64882 3.24194 0.05009
C -2.36058 2.05241 0.00275
C -1.67177 0.85009 0.0054
C -0.29523 0.86604 0.05109
C 0.44161 2.03049 0.09221
C -0.26073 3.23029 0.09414
H -2.17834 4.18437 0.04184
H -3.44106 2.03646 -0.04688
H 1.52041 2.01838 0.09473
H 0.28923 4.16087 0.11279
O -1.42537 -1.54906 -0.66291
C 3.35399 0.20284 -0.19181
O 3.09416 0.4237 -1.35075
C 4.70809 0.46798 0.42357
H 4.61307 1.09235 1.3118
H 5.16799 -0.47445 0.72108
H 5.33589 0.96255 -0.31009
S -2.50864 -0.7231 0.00428
O -3.67265 -0.61008 -0.81221
O -2.66133 -1.08868 1.38147
N 2.41041 -0.3079 0.66999
H 2.66059 -0.45206 1.63663
I 0.62301 -1.03515 0.00133
Sum of electronic and zero-point Energies= -1360.944767
Sum of electronic and thermal Energies= -1360.929737
Sum of electronic and thermal Enthalpies= -1360.928793
Sum of electronic and thermal Free Energies= -1360.990004

4-IIII-OCOCH3 (in Acetonitrile)
C -2.3611 2.96732 -0.00005
C -2.78349 1.6499 -0.00001
C -1.8404 0.63035 -0.00002
C -0.50012 0.94764 -0.00006
C -0.05028 2.25796 -0.00011
C -1.0036 3.26614 -0.0001
H -3.08995 3.76551 -0.00005
H -3.83466 1.39369 0.00003
H 1.00248 2.48729 -0.00014
H -0.67586 4.29646 -0.00014
O -1.03201 -1.76788 -0.00011
C 3.50595 0.07445 0.00003
O 3.59493 -1.13066 0.00002
O 2.32633 0.68704 -0.00012
C 4.67006 1.02226 0.00049
H 4.61482 1.66385 -0.8775
H 4.61732 1.65921 0.88206
H 5.59606 0.45756 -0.00212
S -2.39103 -1.06032 0.00006
O -3.08083 -1.28767 -1.228
O -3.08052 -1.28766 1.2283
I 0.79964 -0.70491 -0.00009
Sum of electronic and zero-point Energies= -1380.824576
Sum of electronic and thermal Energies= -1380.809924
Sum of electronic and thermal Enthalpies= -1380.808980
Sum of electronic and thermal Free Energies= -1380.868674

4-IIII-OH (in Acetonitrile)
C -2.31689 2.56817 -0.02227
C -2.47622 1.18983 -0.01478
C -1.35136 0.38079 -0.01754
C -0.10389 0.9638 -0.03423
C 0.08877 2.32976 -0.03943
C -1.0471 3.13194 -0.03167
H -3.18852 3.2076 -0.02545
H -3.4556 0.7305 -0.01414
H 1.08314 2.75109 -0.06315
H -0.93228 4.20693 -0.04523
O -0.11288 -1.72546 -0.57547
O 2.79214 1.10644 0.26061
H 2.92424 1.2189 1.20713
S -1.45175 -1.3937 0.07493
O -2.5428 -1.83165 -0.73116
O -1.43784 -1.72217 1.46788
I 1.51789 -0.37394 -0.08818
Sum of electronic and zero-point Energies= -1228.201092
Sum of electronic and thermal Energies= -1228.190019
Sum of electronic and thermal Enthalpies= -1228.189075
Sum of electronic and thermal Free Energies= -1228.239582

4-IIII-OTs (in Acetonitrile)
C 3.93603 2.75458 -0.21877
C 4.33377 1.42651 -0.1727
C 3.36448 0.43871 -0.12188
C 2.03299 0.79568 -0.10905
C 1.60334 2.10422 -0.16574
C 2.58774 3.08625 -0.2207
H 4.68233 3.53575 -0.2523
H 5.37789 1.14397 -0.16988
H 0.55707 2.37404 -0.16429
H 2.28437 4.12323 -0.25505
O 2.5061 -1.83086 0.51968
S 3.78017 -1.28578 -0.16615
O 3.80403 -1.67351 -1.54001
O 4.91648 -1.52327 0.65605
O -0.85023 0.37205 -0.5097
S -1.75993 1.11386 0.52964
O -1.76341 2.49868 0.17636
O -1.34856 0.7176 1.84396
C -3.34498 0.43593 0.18852
C -4.09392 0.95475 -0.85978
C -3.81825 -0.60979 0.96351
C -5.33138 0.40248 -1.13382
H -3.7084 1.78334 -1.43761
C -5.06353 -1.14893 0.67511
H -3.22539 -0.97922 1.78895
C -5.8334 -0.65523 -0.37351
H -5.92445 0.79929 -1.94827
H -5.44465 -1.96224 1.27903
C -7.18863 -1.22676 -0.67876
H -7.38192 -2.1234 -0.09374
H -7.97005 -0.4997 -0.45315
H -7.27393 -1.47962 -1.73554
I 0.72609 -0.84907 0.03435
Sum of electronic and zero-point Energies= -2047.075786
Sum of electronic and thermal Energies= -2047.055163
Sum of electronic and thermal Enthalpies= -2047.054219
Sum of electronic and thermal Free Energies= -2047.129385

5-BrIII-anion (in Acetonitrile)
C -2.70564 2.22362 0.0009
C -1.32461 2.16515 0.00019
C -0.60895 0.96239 -0.00023
C -1.36975 -0.21105 0.00004
C -2.76407 -0.16528 0.00072
C -3.43558 1.04355 0.00118
H -3.20907 3.18214 0.00122
H -0.72356 3.06373 -0.00011
H -3.31402 -1.09561 0.00087
H -4.51829 1.05677 0.00173
Br -0.66777 -1.98139 -0.00073
C 0.92848 1.12735 -0.00092
O 1.32963 2.30023 -0.00199
N 1.59349 -0.01914 0.00087
C 2.96119 -0.07315 0.00136
O 3.54356 -1.15094 0.00247
C 3.81382 1.19019 -0.00067
H 3.58375 1.80608 -0.86863
H 3.5838 1.80879 0.86537
H 4.85975 0.88972 -0.00024
11 Br 11 3.0600 1.000 0.667765 -
1.981388 0.000730
11 Br 11 3.0600 1.000 0.804627 -
1.989882 0.002469
11 Br 11 3.0600 1.000 0.730652 -
1.986477 0.011590
11 Br 11 3.0600 1.000 0.675317 -
1.982244 0.051833
11 Br 11 3.0600 1.000 0.709429 -
1.981829 0.106029
11 Br 11 3.0600 1.000 1.102643 -
1.972143 0.161919
11 Br 11 3.0600 1.000 0.979963 -
1.975293 0.190696
11 Br 11 3.0600 1.000 0.713068 -
1.964638 0.252474
11 Br 11 3.0600 1.000 0.507934 -
1.934322 0.200570
11 Br 11 3.0600 1.000 0.435107 -
1.926372 0.185809
11 Br 11 3.0600 1.000 0.390511 -
1.924961 0.142061
11 Br 11 3.0600 1.000 0.367390 -
1.919659 0.054855
11 Br 11 3.0600 1.000 0.403275 -
1.922199 0.014896
11 Br 11 3.0600 1.000 0.454021 -
1.927474 -0.009635
11 Br 11 3.0600 1.000 0.475505 -
1.924874 -0.039903
11 Br 11 3.0600 1.000 0.504615 -
1.928296 -0.046966
11 Br 11 3.0600 1.000 0.541427 -
1.927724 -0.065022
11 Br 11 3.0600 1.000 0.567204 -
1.930104 -0.070430
11 Br 11 3.0600 1.000 0.625037 -
1.937096 -0.075922
11 Br 11 3.0600 1.000 0.627779 -
1.933210 -0.085234
11 Br 11 3.0600 1.000 0.630500 -
1.935091 -0.082485
11 Br 11 3.0600 1.000 0.633221 -
1.935326 -0.083017
11 Br 11 3.0600 1.000 0.653176 -
1.935776 -0.088310
11 Br 11 3.0600 1.000 0.657947 -
1.935880 -0.089487
11 Br 11 3.0600 1.000 0.669288 -
1.936084 -0.092082
11 Br 11 3.0600 1.000 0.662507 -
1.936103 -0.090096
11 Br 11 3.0600 1.000 0.665691 -
1.936033 -0.091078
11 Br 11 3.0600 1.000 0.669010 -
1.935934 -0.092174
11 Br 11 3.0600 1.000 0.669010 -
1.935934 -0.092174
Sum of electronic and zero-point Energies= -3126.579732
Sum of electronic and thermal Energies= -3126.567678
Sum of electronic and thermal Enthalpies= -3126.566734
Sum of electronic and thermal Free Energies= -3126.620912

5-BrIII-Br (in Acetonitrile)
C -0.12242 3.85594 0.
C -1.22379 3.01699 0.00002
C -1.03566 1.64264 0.00001
C 0.25381 1.14434 -0.00001
C 1.37222 1.94558 -0.00003
C 1.15994 3.32056 -0.00002
H -0.25826 4.92824 0.00001
H -2.23893 3.39099 0.00004
H 2.367 1.52892 -0.00005
H 2.01967 3.9763 -0.00004
Br 0.32073 -0.79887 -0.00002

C -2.17681 0.68988 0.00002
O -3.33648 1.02873 0.00008
N -1.68975 -0.59082 -0.00004
C -2.36863 -1.80677 0.
O -1.72138 -2.82636 0.00006
C -3.86977 -1.77147 -0.00007
H -4.23289 -1.23044 0.87185
H -4.23286 -1.23054 -0.87205
H -4.22553 -2.7968 0.
Br 2.85525 -0.98045 0.00002
Sum of electronic and zero-point Energies= -5700.573522
Sum of electronic and thermal Energies= -5700.559986
Sum of electronic and thermal Enthalpies= -5700.559042
Sum of electronic and thermal Free Energies= -5700.615885

5-BrIII-CCH (in Acetonitrile)
C 2.38241 -2.80943 0.00016
C 0.99904 -2.74249 0.00011
C 0.35761 -1.51109 0.00002
C 1.14235 -0.374 -0.00001
C 2.51932 -0.39581 0.00005
C 3.13494 -1.64258 0.00014
H 2.8781 -3.77015 0.00023
H 0.37575 -3.62675 0.00013
H 3.10248 0.51165 0.00003
H 4.2151 -1.69047 0.00018
Br 0.14425 1.30572 -0.00014
C -1.1448 -1.41901 -0.00003
O -1.83436 -2.41972 0.00005
N -1.49488 -0.11859 -0.00002
C -2.76386 0.42705 0.00003
O -2.87468 1.63666 -0.00008
C -3.95427 -0.49274 0.00003
H -3.92334 -1.14629 -0.87039
H -3.92335 -1.14625 0.87047
H -4.85452 0.11408 0.00001
C 1.72983 2.40094 0.00009
C 2.57784 3.2507 0.00019
H 3.33502 3.99934 -0.00017
Sum of electronic and zero-point Energies= -3203.052622
Sum of electronic and thermal Energies= -3203.038251
Sum of electronic and thermal Enthalpies= -3203.037307
Sum of electronic and thermal Free Energies= -3203.095366

5-BrIII-CF3 (in Acetonitrile)
C -0.16406 3.81735 -0.00041
C 0.97592 3.03259 0.00027
C 0.87458 1.64797 0.00037
C -0.38825 1.08404 -0.00013
C -1.54758 1.83397 -0.00086
C -1.41548 3.2178 -0.00099
H -0.08177 4.89518 -0.00053
H 1.9717 3.4554 0.00068
H -2.53192 1.4007 -0.00132
H -2.31108 3.82338 -0.00157
Br -0.39482 -0.86947 0.00002
C 2.1156 0.79458 0.00073
O 3.22111 1.2988 0.0013
C -2.44616 -1.07392 0.00029
F -3.03811 -0.5726 -1.07855
F -3.0378 -0.57182 1.07895
F -2.62072 -2.38847 0.00079
N 1.75012 -0.49974 0.00026
C 2.55293 -1.62501 -0.00048
O 2.02184 -2.71644 -0.00116
C 4.04708 -1.45021 -0.00046
H 4.35683 -0.8738 -0.87081
H 4.35705 -0.87473 0.8704
H 4.50574 -2.43422 -0.00113
Sum of electronic and zero-point Energies= -3463.997995
Sum of electronic and thermal Energies= -3463.982000
Sum of electronic and thermal Enthalpies= -3463.981055
Sum of electronic and thermal Free Energies= -3464.043728

5-BrIII-CH3 (in Acetonitrile)
C -2.95217 -2.14162 0.00011
C -1.58097 -2.33556 0.00027
C -0.70784 -1.25544 0.00005
C -1.25578 0.01702 -0.00018
C -2.61783 0.24712 -0.00043
C -3.46674 -0.85373 -0.00029
H -3.62112 -2.99094 0.00024
H -1.13619 -3.32193 0.00053
H -3.03887 1.23823 -0.00073
H -4.53556 -0.6903 -0.00052
Br 0.00294 1.50101 -0.0002
C 0.78828 -1.49791 -0.00013
O 1.21495 -2.64048 0.00052
C -1.34728 2.96144 0.00136
N 1.41303 -0.31693 -0.00121
C 2.76821 -0.07887 -0.00035
O 3.16153 1.0733 -0.00041
C 3.72445 -1.24393 0.00076
H 3.54599 -1.87415 0.87084
H 3.54678 -1.87522 -0.86868
H 4.73956 -0.85815 0.00104
H -1.94734 2.91361 0.90356
H -0.72585 3.85272 0.00222
H -1.94748 2.91551 -0.90085
Sum of electronic and zero-point Energies= -3166.222387
Sum of electronic and thermal Energies= -3166.208500
Sum of electronic and thermal Enthalpies= -3166.207556
Sum of electronic and thermal Free Energies= -3166.265859

5-BrIII-CHCH2 (in Acetonitrile)
C -2.08826 -3.06158 -0.07537
C -0.71891 -2.85539 -0.03913
C -0.19444 -1.56987 0.00692
C -1.08875 -0.51452 0.02802
C -2.45794 -0.67844 -0.02552
C -2.95304 -1.9763 -0.07595
H -2.48432 -4.06687 -0.11327
H -0.01039 -3.67304 -0.05262
H -3.1345 0.1614 -0.04544
H -4.02266 -2.12825 -0.11998
Br -0.31576 1.27274 0.12696
C 1.30592 -1.359 -0.00538
O 2.05241 -2.32244 0.0341
C -2.00918 2.21894 0.37185
N 1.55342 -0.04649 -0.07319
C 2.77882 0.58026 -0.06738
O 2.81474 1.79704 -0.06807
C 4.03582 -0.25124 -0.06207
H 4.05959 -0.88572 0.82287
H 4.04357 -0.92692 -0.91601
H 4.89195 0.41644 -0.08456
H -2.51932 2.02608 1.30597
C -2.36695 3.13409 -0.50658
H -3.23411 3.75973 -0.33348
H -1.80801 3.29808 -1.41952
Sum of electronic and zero-point Energies= -3204.290981
Sum of electronic and thermal Energies= -3204.276297
Sum of electronic and thermal Enthalpies= -3204.275353
Sum of electronic and thermal Free Energies= -3204.334618

5-BrIII-Cl (in Acetonitrile)
C 2.6144 2.65395 0.00001
C 1.23197 2.72934 -0.00023
C 0.48589 1.5599 -0.00013
C 1.14528 0.3455 0.00012
C 2.51663 0.22967 0.00041
C 3.24468 1.41512 0.00035
H 3.20604 3.55851 -0.00005
H 0.70161 3.67218 -0.00047
H 3.00125 -0.73347 0.00064
H 4.32447 1.35865 0.00058
Br -0.06053 -1.17417 0.00019
C -1.00013 1.56634 -0.00017
O -1.67017 2.57137 -0.00073
Cl 1.68312 -2.78892 -0.00054
N -1.45179 0.27122 0.00061
C -2.75755 -0.21569 0.00018
O -2.92296 -1.41205 -0.00001
C -3.87975 0.78123 0.00007
H -3.80737 1.42931 0.87159
H -3.80768 1.42852 -0.87207
H -4.81376 0.22852 0.00041
Sum of electronic and zero-point Energies= -3586.572288
Sum of electronic and thermal Energies= -3586.558930
Sum of electronic and thermal Enthalpies= -3586.557986
Sum of electronic and thermal Free Energies= -3586.613565

5-BrIII-CN (in Acetonitrile)
C 2.60084 2.6064 -0.00002
C 1.21704 2.65502 -0.00002
C 0.48475 1.47627 -0.00003
C 1.17097 0.27792 -0.00003
C 2.54497 0.18457 -0.00001
C 3.25468 1.38095 0.00001
H 3.17497 3.5222 -0.00002
H 0.66877 3.58755 -0.00002
H 3.06183 -0.76243 0.00001
H 4.33505 1.34113 0.00003
Br -0.0046 -1.27897 -0.00002
C -1.01177 1.4823 -0.00004
O -1.65624 2.50716 0.00003
C 1.59047 -2.53041 0.00011
N 2.42488 -3.32144 0.00015
N -1.45392 0.19802 -0.00016
C -2.75836 -0.27842 -0.00006
O -2.92899 -1.47748 -0.00025
C -3.88492 0.71419 0.0003
H -3.8135 1.36276 0.87188
H -3.81327 1.36364 -0.8706
H -4.81984 0.16278 -0.00011
Sum of electronic and zero-point Energies= -3219.163926

Sum of electronic and thermal Energies= -3219.149594
Sum of electronic and thermal Enthalpies= -3219.148650
Sum of electronic and thermal Free Energies= -3219.206478

5-BrIII-F (in Acetonitrile)
C 3.26195 1.66685 -0.00017
C 1.94749 2.10243 -0.00049
C 0.92502 1.16453 -0.00032
C 1.24771 -0.17701 0.00001
C 2.54167 -0.65071 0.00045
C 3.55107 0.30549 0.00036
H 4.069 2.38593 -0.00028
H 1.6842 3.15153 -0.00085
H 2.74366 -1.70948 0.00072
H 4.58054 -0.02537 0.0007
Br -0.28078 -1.33269 0.00009
C -0.51485 1.53002 -0.00037
O -0.9251 2.666 -0.00088
F 0.89751 -2.81504 -0.00054
N -1.26579 0.3777 0.00065
C -2.65272 0.23513 0.0003
O -3.11034 -0.88329 -0.00013
C -3.4938 1.47723 0.00072
H -3.26486 2.08794 0.87214
H -3.26504 2.08799 -0.87076
H -4.53511 1.17143 0.00066
Sum of electronic and zero-point Energies= -3226.205999
Sum of electronic and thermal Energies= -3226.193232
Sum of electronic and thermal Enthalpies= -3226.192288
Sum of electronic and thermal Free Energies= -3226.246271

5-BrIII-N3 (in Acetonitrile)
C -1.55061 3.41802 0.08288
C -0.222 3.04577 0.20157
C 0.12756 1.70915 0.07351
C -0.86717 0.78179 -0.16576
C -2.19726 1.1134 -0.29799
C -2.52494 2.45876 -0.16736
H -1.83214 4.45699 0.18108
H 0.56653 3.76186 0.39056
H -2.94426 0.36443 -0.50683
H -3.56063 2.75317 -0.26623
Br -0.22005 -1.0393 -0.31587
C 1.53889 1.24103 0.17575
O 2.46999 1.98271 0.38992
N 1.57082 -0.11144 -0.01446
C 2.65703 -0.97898 0.0032
O 2.45333 -2.15831 -0.17235
C 4.02309 -0.40168 0.239
H 4.25035 0.3488 -0.51601
H 4.05339 0.11018 1.19932
H 4.73944 -1.21652 0.20854
N -2.11964 -1.77388 -0.63989
N -3.01107 -2.63304 1.35273
N -2.56369 -2.2134 0.40955
Sum of electronic and zero-point Energies= -3290.522585
Sum of electronic and thermal Energies= -3290.507739
Sum of electronic and thermal Enthalpies= -3290.506795
Sum of electronic and thermal Free Energies= -3290.566272

5-BrIII-NH2 (in Acetonitrile)
C 3.1376 1.89196 0.00013
C 1.79155 2.21901 0.00026
C 0.83151 1.21658 0.00026
C 1.25933 -0.09841 0.0001
C 2.5874 -0.46398 -0.00003
C 3.52912 0.55947 -0.00001
H 3.88466 2.67347 0.00013
H 1.44406 3.24356 0.00038
H 2.87205 -1.50393 -0.00016
H 4.57945 0.3021 -0.00012
Br -0.15508 -1.43499 0.00011
C -0.63477 1.54004 0.00042
O -1.03248 2.68844 0.00023
N -1.32957 0.3822 0.00008
C -2.69771 0.20468 -0.00034
O -3.13975 -0.92763 -0.00073
C -3.59068 1.41606 -0.0006
N 1.09846 -2.91164 0.00013
H -3.38217 2.03624 0.86972
H -3.38139 2.03648 -0.87055
H -4.62261 1.07858 -0.0011
H 0.87418 -3.4678 0.81858
H 0.87527 -3.46692 -0.81921
Sum of electronic and zero-point Energies= -3182.270169
Sum of electronic and thermal Energies= -3182.256456
Sum of electronic and thermal Enthalpies= -3182.255512
Sum of electronic and thermal Free Energies= -3182.312276

5-BrIII-NHAc (in Acetonitrile)
C 0.10175 3.79428 -0.00092
C 1.19917 2.95853 -0.11667
C 1.03167 1.58436 -0.01717
C -0.23835 1.08655 0.20055
C -1.35399 1.88497 0.3132
C -1.16263 3.25783 0.21144
H 0.22557 4.86526 -0.0799
H 2.19936 3.33407 -0.28634
H -2.33309 1.46574 0.47064
H -2.02092 3.91033 0.29308
Br -0.32736 -0.84663 0.31105
C 2.18234 0.63849 -0.1416
O 3.31982 1.01557 -0.32425
C -3.12658 -0.85927 -0.40466
O -2.80254 -0.40736 -1.48105
C -4.49882 -1.43924 -0.1387
H -4.43011 -2.52773 -0.14636
H -4.88476 -1.12965 0.83156
H -5.17358 -1.12215 -0.92758
N 1.71644 -0.63159 -0.0125
C 2.42308 -1.8216 -0.04466
O 1.8187 -2.85905 0.12787
C 3.90471 -1.77356 -0.29262
H 4.11256 -1.27269 -1.23655
H 4.39539 -1.18349 0.47965
H 4.27822 -2.79279 -0.30043
N -2.2648 -0.8569 0.65973
H -2.5344 -1.35674 1.49521
Sum of electronic and zero-point Energies= -3334.899494
Sum of electronic and thermal Energies= -3334.882307
Sum of electronic and thermal Enthalpies= -3334.881362
Sum of electronic and thermal Free Energies= -3334.946673

5-BrIII-OCOCH3 (in Acetonitrile)
C 1.63714 3.63895 -0.00018
C 2.3238 2.43747 -0.00017
C 1.61133 1.2471 -0.00002
C 0.22981 1.28449 0.0001
C -0.48683 2.46466 0.00011
C 0.24673 3.64566 -0.00004
H 2.18007 4.57361 -0.0003
H 3.40428 2.38576 -0.00027
H -1.56292 2.46625 0.00019
H -0.2871 4.58611 -0.00005
Br -0.57322 -0.46698 0.00031
C 2.28255 -0.07898 0.00003
O 3.48193 -0.22651 -0.0001
C -3.34264 -0.4131 0.00002
O -3.14384 -1.60602 0.0004
O -2.37521 0.48463 0.
C -4.71944 0.20482 -0.00076
H -4.83446 0.83892 0.87706
H -4.83535 0.83366 -0.88228
H -5.46909 -0.57976 0.0018
N 1.32578 -1.06142 0.0005
C 1.48423 -2.44873 -0.00008
O 0.49228 -3.13523 -0.00021
C 2.88066 -2.99879 -0.00053
H 3.42605 -2.64223 -0.8724
H 3.42669 -2.64251 0.87103
H 2.80888 -4.08173 -0.00075
Sum of electronic and zero-point Energies= -3354.790414
Sum of electronic and thermal Energies= -3354.774308
Sum of electronic and thermal Enthalpies= -3354.773364
Sum of electronic and thermal Free Energies= -3354.835843

5-BrIII-OH (in Acetonitrile)
C 3.21768 1.75417 0.00207
C 1.89019 2.14934 0.01605
C 0.89019 1.18766 0.01092
C 1.24981 -0.14545 -0.00285
C 2.55805 -0.57723 -0.02621
C 3.5445 0.4029 -0.02149
H 4.00317 2.49685 0.00498
H 1.59533 3.19007 0.02866
H 2.79114 -1.62939 -0.0586
H 4.58206 0.09878 -0.04058
Br -0.23393 -1.37952 -0.01295
C -0.56014 1.53899 0.01195
O -0.96016 2.68231 0.03317
O 0.93976 -2.88065 -0.03835
H 0.94814 -3.2383 0.85544
N -1.28731 0.38664 -0.01527
C -2.66582 0.23325 -0.00794
O -3.12193 -0.88933 -0.0041
C -3.52615 1.46508 -0.006
H -3.30906 2.07371 0.87017
H -3.29908 2.08529 -0.8713
H -4.56443 1.14865 -0.01354
Sum of electronic and zero-point Energies= -3202.159072
Sum of electronic and thermal Energies= -3202.145838
Sum of electronic and thermal Enthalpies= -3202.144894
Sum of electronic and thermal Free Energies= -3202.199583

5-BrIII-OTs (in Acetonitrile)
C 3.83639 3.0855 0.07705

C	4.26995	1.7758	0.18766
C	3.35192	0.74718	0.03222
C	2.03157	1.05831	-0.22611
C	1.56073	2.34552	-0.35181
C	2.49949	3.35999	-0.19065
H	4.53753	3.899	0.19857
H	5.30075	1.51976	0.39216
H	0.52706	2.57363	-0.55921
H	2.1652	4.38466	-0.27559
Br	0.93508	-0.52191	-0.3933
C	3.72405	-0.68245	0.12453
O	4.83434	-1.09495	0.34459
N	2.59816	-1.45949	-0.08897
C	2.45951	-2.85157	-0.08536
O	1.36306	-3.31207	-0.28747
C	3.68497	-3.67594	0.16897
H	4.4489	-3.4564	-0.57444
H	4.1136	-3.4245	1.13732
H	3.39153	-4.72001	0.13119
O	-0.74574	0.66484	-0.78531
S	-1.55533	1.24135	0.38454
O	-1.73699	2.64909	0.17014
O	-0.95923	0.81072	1.62138
C	-3.1265	0.45566	0.22004
C	-4.13345	1.08092	-0.49671
C	-3.33006	-0.78676	0.80342
C	-5.3575	0.4444	-0.63642
H	-3.95638	2.05865	-0.92267
C	-4.55741	-1.40928	0.65397
H	-2.53887	-1.24383	1.38236
C	-5.58544	-0.80669	-0.07004
H	-6.15172	0.93096	-1.18849
H	-4.72612	-2.37515	1.11367
C	-6.90421	-1.50552	-0.2465
H	-7.18358	-2.05054	0.65426
H	-7.69751	-0.79846	-0.48243
H	-6.84752	-2.22756	-1.06321

Sum of electronic and zero-point Energies= -4021.052183
Sum of electronic and thermal Energies= -4021.030127
Sum of electronic and thermal Enthalpies= -4021.029183
Sum of electronic and thermal Free Energies= -4021.107158

5-ClIII-anion (in Acetonitrile)

C	-2.9595	1.60822	0.00123
C	-1.57793	1.66298	0.00041
C	-0.76606	0.52383	-0.00031
C	-1.4279	-0.70965	-0.00027
C	-2.82174	-0.77668	0.00051
C	-3.59051	0.37204	0.00129
H	-3.5401	2.52202	0.00181
H	-1.05311	2.60808	0.00027
H	-3.28767	-1.75222	0.00046
H	-4.67068	0.29696	0.00191
C	0.75289	0.80796	-0.00106
O	1.05921	2.0095	-0.0024
N	1.50893	-0.28209	0.00068
C	2.87631	-0.22086	0.00096
O	3.54759	-1.24604	0.00258
C	3.62249	1.10873	-0.00027
H	3.34329	1.70412	-0.86813
H	3.34236	1.70628	0.86581
H	4.68953	0.89474	0.00057
Cl	-0.64052	-2.25721	-0.0014

Sum of electronic and zero-point Energies= -1012.585565
Sum of electronic and thermal Energies= -1012.573692
Sum of electronic and thermal Enthalpies= -1012.572747
Sum of electronic and thermal Free Energies= -1012.626075

5-ClIII-Br (in Acetonitrile)

C	0.11857	3.74082	0.00015
C	-1.03032	2.96877	0.00031
C	-0.91631	1.58776	0.00023
C	0.33853	1.00945	0.
C	1.50237	1.74347	-0.00016
C	1.36594	3.12823	-0.00008
H	0.04797	4.81929	0.0002
H	-2.02241	3.40002	0.00049
H	2.47108	1.27133	-0.00034
H	2.26301	3.73175	-0.00021
C	-2.08211	0.67578	0.00039
O	-3.23754	1.02314	0.0006
N	-1.60441	-0.6098	0.00023
C	-2.2655	-1.84042	0.00027
O	-1.61205	-2.85134	0.00011
C	-3.76695	-1.80053	0.00053
H	-4.12857	-1.26011	0.87347
H	-4.12888	-1.25977	-0.87209
H	-4.12373	-2.82556	0.0004
Br	2.72078	-1.22393	-0.00051
Cl	0.28451	-0.78972	-0.00007

Sum of electronic and zero-point Energies= -3586.549938
Sum of electronic and thermal Energies= -3586.536632
Sum of electronic and thermal Enthalpies= -3586.535688
Sum of electronic and thermal Free Energies= -3586.591863

5-ClIII-CCH (in Acetonitrile)

C	2.56717	-2.4175	0.0004
C	1.18215	-2.43792	0.00028
C	0.45658	-1.25438	0.00012
C	1.17413	-0.07527	0.0001
C	2.54821	-0.00177	0.00022
C	3.24367	-1.20598	0.00037
H	3.12311	-3.34463	0.00053
H	0.61387	-3.35877	0.0003
H	3.07398	0.93943	0.0002
H	4.32443	-1.1821	0.00046
C	-1.05493	-1.26232	-0.00002
O	-1.66399	-2.3168	0.00006
N	-1.46162	0.01049	-0.00021
C	-2.75421	0.49066	-0.0003
O	-2.93231	1.6929	-0.00041
C	-3.89862	-0.48921	-0.00022
H	-3.83379	-1.14095	-0.87039
H	-3.83383	-1.14077	0.87009
H	-4.83059	0.06802	-0.0003
C	1.46263	2.59628	-0.00005
C	2.187	3.54923	-0.00004
H	2.83955	4.3903	-0.00003
Cl	0.1672	1.45507	-0.0001

Sum of electronic and zero-point Energies= -1089.037404
Sum of electronic and thermal Energies= -1089.023929
Sum of electronic and thermal Enthalpies= -1089.022984
Sum of electronic and thermal Free Energies= -1089.079439

5-ClIII-CF3 (in Acetonitrile)

C	-0.65961	3.62908	-0.00017
C	0.55862	2.97283	0.00018
C	0.61523	1.58627	0.00018
C	-0.57913	0.88909	-0.00011
C	-1.81518	1.50526	-0.0005
C	-1.83614	2.89512	-0.00052
H	-0.6965	4.7094	-0.00019
H	1.50404	3.49906	0.00043
H	-2.7492	0.97407	-0.00079
H	-2.79429	3.39559	-0.00082
C	1.94733	0.87395	0.00037
O	2.98696	1.50672	0.0008
C	-2.22206	-1.42764	0.00017
F	-2.15077	-2.74168	0.00059
F	-2.86795	-1.02647	-1.07959
F	-2.86799	-1.02578	1.07963
N	1.7016	-0.43895	-0.00001
C	2.59925	-1.48652	-0.00026
O	2.17252	-2.6234	-0.00033
C	4.0733	-1.17697	-0.00047
H	4.32878	-0.57341	-0.8703
H	4.32925	-0.57551	0.87069
H	4.62263	-2.11357	-0.00162
Cl	-0.40241	-0.92511	0.00001

Sum of electronic and zero-point Energies= -1349.985955
Sum of electronic and thermal Energies= -1349.970344
Sum of electronic and thermal Enthalpies= -1349.969400
Sum of electronic and thermal Free Energies= -1350.031471

5-ClIII-CH3 (in Acetonitrile)

C	-3.15846	-1.50802	-0.32806
C	-1.80629	-1.77595	-0.18986
C	-0.87437	-0.7695	0.05344
C	-1.40176	0.50864	0.16758
C	-2.74586	0.82204	0.04937
C	-3.63411	-0.20852	-0.21257
H	-3.84831	-2.31736	-0.52486
H	-1.41171	-2.78088	-0.2619
H	-3.08673	1.84046	0.16732
H	-4.68802	0.00779	-0.31551
C	0.60859	-1.1687	0.13157
O	0.84585	-2.35303	0.3355
C	0.60536	2.45862	-0.79419
N	1.37838	-0.10726	-0.07522
C	2.7476	-0.13507	-0.10432
O	3.35035	0.92296	-0.25397
C	3.51114	-1.42933	0.03403
H	3.28873	-1.89032	0.99555
H	3.1822	-2.14389	-0.71913
H	4.57339	-1.22341	-0.06032
H	0.24735	1.86643	-1.62743
H	0.3704	3.51471	-0.88135
H	1.63407	2.19965	-0.54036
Cl	-0.38768	1.90311	0.63532

Sum of electronic and zero-point Energies= -1052.220022
Sum of electronic and thermal Energies= -1052.206037
Sum of electronic and thermal Enthalpies= -1052.205093
Sum of electronic and thermal Free Energies= -1052.262640

5-ClIII-CHCH2 (in Acetonitrile)

C	-2.39104	-2.62097	-0.10459

C	-1.01105	-2.53668	-0.01466
C	-0.36829	-1.30643	0.03555
C	-1.17091	-0.18016	0.01529
C	-2.54582	-0.21584	-0.09698
C	-3.15435	-1.46394	-0.15631
H	-2.87363	-3.5875	-0.14789
H	-0.37685	-3.41332	0.00905
H	-3.14225	0.68083	-0.15611
H	-4.2302	-1.51826	-0.2472
C	1.15078	-1.2364	0.05233
O	1.78766	-2.27011	0.1894
C	-1.73112	2.47787	0.47659
N	1.50648	0.03569	-0.11191
C	2.78787	0.53868	-0.10204
O	2.9506	1.74408	-0.1569
C	3.95998	-0.40915	-0.02664
H	3.91437	-0.98458	0.89715
H	3.91014	-1.13715	-0.83498
H	4.87808	0.16884	-0.07776
H	-2.24373	2.25165	1.4002
C	-1.94304	3.51432	-0.30611
H	-2.70159	4.24226	-0.05113
H	-1.36398	3.674	-1.20609
Cl	-0.33646	1.42435	0.1355

Sum of electronic and zero-point Energies= -1090.288530
Sum of electronic and thermal Energies= -1090.274058
Sum of electronic and thermal Enthalpies= -1090.273114
Sum of electronic and thermal Free Energies= -1090.331567

5-ClIII-Cl (in Acetonitrile)

C	-2.79565	-2.2418	0.00032
C	-1.42715	-2.4491	0.00035
C	-0.58007	-1.35227	0.00019
C	-1.11562	-0.07942	0.00001
C	-2.46953	0.16721	-0.00003
C	-3.30253	-0.94733	0.00013
H	-3.47211	-3.08472	0.00044
H	-0.98729	-3.43732	0.00049
H	-2.86089	1.17095	-0.00018
H	-4.37193	-0.78803	0.00011
C	0.89513	-1.4563	0.0002
O	1.52342	-2.48579	0.00037
Cl	-1.32493	2.99942	-0.00048
N	1.39982	-0.17867	0.00001
C	2.71977	0.28457	0.
O	2.91682	1.47187	-0.0001
C	3.80282	-0.755	0.0001
H	3.70703	-1.39853	0.87276
H	3.70716	-1.3986	-0.87252
H	4.75626	-0.23646	0.00015
Cl	0.13893	1.20713	-0.00017

Sum of electronic and zero-point Energies= -1472.549796
Sum of electronic and thermal Energies= -1472.536623
Sum of electronic and thermal Enthalpies= -1472.535679
Sum of electronic and thermal Free Energies= -1472.590651

5-ClIII-CN (in Acetonitrile)

C	-2.74895	-2.21625	0.00005
C	-1.37163	-2.36081	0.00001
C	-0.55751	-1.23817	-0.00004
C	-1.15949	0.00324	-0.00005
C	-2.5227	0.1959	0.
C	-3.31431	-0.94831	0.00006
H	-3.38618	-3.0893	0.00009
H	-0.88755	-3.32836	0.00002
H	-2.97657	1.17407	0.
H	-4.38885	-0.83069	0.00012
C	0.93779	-1.33196	-0.00007
O	1.52157	-2.39396	-0.00001
C	-1.28649	2.68355	0.00005
N	-1.9745	3.60323	0.00013
N	1.41756	-0.07096	-0.00021
C	2.73015	0.37521	0.
O	2.93573	1.56854	-0.00001
C	3.82882	-0.65052	0.00021
H	3.73763	-1.29724	0.87156
H	3.73796	-1.29711	-0.87128
H	4.78082	-0.12891	0.0004
Cl	0.02405	1.38846	-0.00009

Sum of electronic and zero-point Energies= -1105.139112
Sum of electronic and thermal Energies= -1105.124852
Sum of electronic and thermal Enthalpies= -1105.123908
Sum of electronic and thermal Free Energies= -1105.182294

5-ClIII-F (in Acetonitrile)

C	-3.21449	-1.41814	0.00027
C	-1.91055	-1.88255	-0.00004
C	-0.87367	-0.96214	-0.00017
C	-1.16184	0.38546	-0.00002
C	-2.44493	0.88817	0.00035
C	-3.47109	-0.04993	0.00048
H	-4.0382	-2.11796	0.00041
H	-1.66779	-2.93648	-0.00017
H	-2.62535	1.94954	0.00055
H	-4.49291	0.30374	0.00072
C	0.56246	-1.31194	-0.00042
O	1.01099	-2.43156	-0.00062
F	-0.7581	2.89677	-0.00034
N	1.26977	-0.13034	-0.00041
C	2.65384	0.08212	0.00023
O	3.06313	1.21504	0.00034
C	3.5362	-1.13115	0.00067
H	3.32942	-1.7495	0.87225
H	3.33102	-1.74871	-0.87188
H	4.56565	-0.78748	0.00165
Cl	0.28731	1.41536	-0.0002

Sum of electronic and zero-point Energies= -1112.182382
Sum of electronic and thermal Energies= -1112.169777
Sum of electronic and thermal Enthalpies= -1112.168833
Sum of electronic and thermal Free Energies= -1112.222139

5-ClIII-N3 (in Acetonitrile)

C	-3.76213	0.02983	-0.00014
C	-2.84382	1.0656	-0.00031
C	-1.48964	0.7711	-0.00018
C	-1.08508	-0.54968	0.00011
C	-1.96832	-1.60846	0.0003
C	-3.32213	-1.28831	0.00016
H	-4.82155	0.24387	-0.00025
H	-3.13925	2.10616	-0.00055
H	-1.62918	-2.62932	0.00056
H	-4.04048	-2.09641	0.00028
C	-0.43161	1.81267	-0.00038
O	-0.64337	3.00248	-0.00041
N	0.77414	1.17272	-0.00056
C	2.07141	1.67965	0.00015
O	2.9976	0.9065	0.00059
C	2.22572	3.17388	0.0003
H	1.73565	3.60274	-0.8721
H	1.73485	3.60266	0.87228
H	3.28772	3.39825	0.00079
N	0.51943	-2.73412	0.00141
N	2.731	-3.50805	-0.00164
N	1.67142	-3.12543	-0.00023
Cl	0.69412	-0.74431	0.00027

Sum of electronic and zero-point Energies= -1176.499544
Sum of electronic and thermal Energies= -1176.484740
Sum of electronic and thermal Enthalpies= -1176.483796
Sum of electronic and thermal Free Energies= -1176.544494

5-ClIII-NH2 (in Acetonitrile)

C	-3.10529	-1.63159	0.00016
C	-1.7613	-1.96717	-0.00006
C	-0.79295	-0.97437	-0.0002
C	-1.21045	0.34434	-0.00014
C	-2.53559	0.72012	0.00013
C	-3.48485	-0.29669	0.00028
H	-3.8586	-2.40701	0.00025
H	-1.41818	-2.99335	-0.00012
H	-2.81966	1.75871	0.00017
H	-4.53236	-0.02864	0.00051
C	0.67431	-1.29994	-0.00027
O	1.07148	-2.44987	-0.00005
N	1.33911	-0.13317	-0.00064
C	2.69849	0.08589	-0.00001
O	3.11137	1.22954	0.00005
C	3.62433	-1.1024	0.00058
N	-0.96816	3.01971	0.00032
H	3.4309	-1.72779	0.87089
H	3.43173	-1.72796	-0.8698
H	4.64815	-0.74055	0.00103
H	-0.68703	3.5483	0.82023
H	-0.68785	3.54835	-0.81983
Cl	0.10549	1.59163	-0.00023

Sum of electronic and zero-point Energies= -1068.254561
Sum of electronic and thermal Energies= -1068.241811
Sum of electronic and thermal Enthalpies= -1068.240867
Sum of electronic and thermal Free Energies= -1068.296675

5-ClIII-NHAc (in Acetonitrile)

C	-0.01744	3.68042	-0.01545
C	1.08911	2.85844	-0.14087
C	0.94549	1.48562	-0.00937
C	-0.30938	0.96938	0.24774
C	-1.43413	1.75346	0.37222
C	-1.26563	3.12685	0.23918
H	0.08643	4.75143	-0.11844
H	2.08032	3.24263	-0.34149
H	-2.40387	1.32973	0.56415
H	-2.13329	3.76532	0.33073
C	2.09641	0.5418	-0.13299
O	3.23073	0.90988	-0.35477
C	-2.9954	-1.05398	-0.36849
O	-2.69714	-0.5864	-1.4416
C	-4.29437	-1.78177	-0.10957
H	-4.10262	-2.85559	-0.11485

H	-4.71989	-1.51556	0.85683
H	-4.99377	-1.54402	-0.90486
N	1.60857	-0.7052	0.05259
C	2.27126	-1.91807	0.04486
O	1.64399	-2.93097	0.27198
C	3.74681	-1.92029	-0.2478
H	3.9396	-1.45462	-1.21291
H	4.27579	-1.32037	0.49114
H	4.09389	-2.94889	-0.23415
N	-2.16393	-0.9267	0.72272
H	-2.34256	-1.51138	1.52826
Cl	-0.37115	-0.8298	0.39099

Sum of electronic and zero-point Energies= -1220.878215
Sum of electronic and thermal Energies= -1220.861305
Sum of electronic and thermal Enthalpies= -1220.860361
Sum of electronic and thermal Free Energies= -1220.924888

5-ClIII-OCOCH3 (in Acetonitrile)

C	-1.81664	3.44723	-0.00017
C	-2.41812	2.20159	-0.0006
C	-1.61797	1.06983	-0.00043
C	-0.24397	1.19932	0.00017
C	0.39025	2.42563	0.00069
C	-0.42963	3.54875	0.0005
H	-3.49193	2.07204	-0.00111
H	1.46234	2.50473	0.00123
H	0.03701	4.5242	0.00089
C	-2.14863	-0.31002	-0.00079
O	-3.31815	-0.60654	-0.00081
C	3.29223	-0.28737	-0.00069
O	3.12013	-1.48387	-0.00148
O	2.3148	0.59711	0.00031
C	4.66051	0.35528	-0.00001
H	4.7663	0.98965	-0.87891
H	4.7665	0.98721	0.88063
H	5.4231	-0.41706	-0.00113
N	-1.07329	-1.16505	0.00019
C	-1.03612	-2.56928	0.00123
O	0.03238	-3.11757	0.00105
C	-2.35665	-3.28343	0.00021
H	-2.94209	-2.99848	0.87235
H	-2.94077	-2.99856	-0.87286
H	-2.1505	-4.34902	0.00039
Cl	0.59765	-0.37648	0.00025
H	-2.42182	4.34255	-0.00034

Sum of electronic and zero-point Energies= -1240.764634
Sum of electronic and thermal Energies= -1240.747979
Sum of electronic and thermal Enthalpies= -1240.747034
Sum of electronic and thermal Free Energies= -1240.810866

5-ClIII-OH (in Acetonitrile)

C	-3.17793	-1.49575	0.00917
C	-1.85741	-1.91295	0.01655
C	-0.84472	-0.96684	0.00673
C	-1.17652	0.3731	-0.00444
C	-2.47764	0.82685	-0.02123
C	-3.4785	-0.13891	-0.01143
H	-3.97696	-2.22372	0.01581
H	-1.57755	-2.95782	0.02795
H	-2.6983	1.88023	-0.04824
H	-4.51005	0.185	-0.02408
C	0.60273	-1.31306	0.00459
O	1.02271	-2.44894	0.02324
O	-0.80523	2.96024	-0.05096
H	-0.66015	3.36897	0.80933
N	1.29227	-0.14188	-0.02064
C	2.66322	0.07247	-0.00447
O	3.07706	1.20941	0.01111
C	3.56421	-1.1309	-0.00759
H	3.36467	-1.75232	0.86387
H	3.36189	-1.75187	-0.8786
H	4.59152	-0.78009	-0.00861
Cl	0.22341	1.49265	-0.02396

Sum of electronic and zero-point Energies= -1088.133568
Sum of electronic and thermal Energies= -1088.120574
Sum of electronic and thermal Enthalpies= -1088.119630
Sum of electronic and thermal Free Energies= -1088.173508

5-ClIII-OTs (in Acetonitrile)

C	4.01226	2.90702	0.04666
C	4.39999	1.58576	0.18005
C	3.44615	0.59475	0.00483
C	2.1462	0.94571	-0.29177
C	1.71844	2.24467	-0.44273
C	2.69211	3.22229	-0.26121
H	4.73498	3.69909	0.18159
H	5.41495	1.29613	0.41553
H	0.69904	2.50472	-0.67763
H	2.39613	4.25719	-0.36207
C	3.71109	-0.84836	0.10624
O	4.76049	-1.37657	0.35598
N	2.51529	-1.51619	-0.15158
C	2.20723	-2.89197	-0.13866
O	1.07568	-3.22357	-0.36495
C	3.33727	-3.82865	0.15824
H	4.13845	-3.69791	-0.56661
H	3.76119	-3.6095	1.13653
H	2.93963	-4.83771	0.12313
O	-0.57985	0.67094	-0.87056
S	-1.34137	1.19959	0.33327
O	-1.50558	2.62447	0.2198
O	-0.72888	0.69589	1.53851
C	-2.93754	0.45803	0.17845
C	-4.01532	1.2182	-0.23695
C	-3.08788	-0.89213	0.47082
C	-5.26098	0.61607	-0.36364
H	-3.87316	2.2686	-0.44898
C	-4.33253	-1.47788	0.33773
H	-2.23607	-1.46815	0.8083
C	-5.43702	-0.73311	-0.0813
H	-6.10881	1.20753	-0.68559
H	-4.45655	-2.52967	0.56516
C	-6.78367	-1.38822	-0.21063
H	-7.13079	-1.75298	0.7571
H	-7.52635	-0.69276	-0.59674
H	-6.73568	-2.24519	-0.88333
Cl	1.10401	-0.50013	-0.45911

Sum of electronic and zero-point Energies= -1907.035663
Sum of electronic and thermal Energies= -1907.012841
Sum of electronic and thermal Enthalpies= -1907.011897
Sum of electronic and thermal Free Energies= -1907.092674

5-IIII-anion (in Acetonitrile)

C	-2.72012	2.48038	0.20676
C	-1.34688	2.47191	0.0375
C	-0.61455	1.28454	-0.01475
C	-1.32614	0.09246	0.06315
C	-2.71014	0.08242	0.21066
C	-3.40732	1.27681	0.29733
H	-3.25518	3.41999	0.2614
H	-0.78873	3.39273	-0.06909
H	-3.23753	-0.86035	0.25394
H	-4.48244	1.26181	0.4236
C	0.89765	1.42801	-0.17714
O	1.27119	2.29898	-0.9762
N	1.58752	0.65219	0.64295
C	2.93258	0.4838	0.49281
O	3.66334	0.17396	1.42376
C	3.54741	0.59941	-0.89918
H	2.84908	0.30077	-1.67915
H	3.80061	1.64225	-1.08586
H	4.44979	-0.00882	-0.92558
I	-0.40452	-1.78671	-0.12383

Sum of electronic and zero-point Energies= -850.013678
Sum of electronic and thermal Energies= -850.001549
Sum of electronic and thermal Enthalpies= -850.000605
Sum of electronic and thermal Free Energies= -850.054990

5-IIII-Br (in Acetonitrile)

C	-0.39352	3.96921	-0.00001
C	-1.44049	3.0634	-0.00002
C	-1.17312	1.70073	0.
C	0.14671	1.27612	0.00003
C	1.20907	2.15477	0.00003
C	0.91959	3.51483	0.00002
H	-0.59672	5.03092	-0.00002
H	-2.4758	3.37708	-0.00003
H	2.22917	1.80156	0.00005
H	1.73749	4.22225	0.00002
C	-2.28697	0.70725	0.
O	-3.45315	1.02686	-0.00004
N	-1.78547	-0.57003	0.00004
C	-2.47535	-1.77369	0.
O	-1.82208	-2.79595	0.00007
C	-3.97507	-1.75666	-0.00013
H	-4.34381	-1.21792	0.87088
H	-4.34367	-1.21818	-0.87136
H	-4.32168	-2.78504	-0.00001
Br	2.99879	-0.70125	-0.00006
I	0.35231	-0.82377	0.00004

Sum of electronic and zero-point Energies= -3424.050903
Sum of electronic and thermal Energies= -3424.037222
Sum of electronic and thermal Enthalpies= -3424.036278
Sum of electronic and thermal Free Energies= -3424.093990

5-IIII-CCH (in Acetonitrile)

C	-2.047	3.2556	-0.00001
C	-0.67999	3.03538	0.
C	-0.18289	1.73795	0.00001
C	-1.08283	0.68445	0.00001
C	-2.45008	0.87294	0.00001
C	-2.92503	2.17905	0.
H	-2.43234	4.26575	-0.00002
H	0.03478	3.84725	-0.00001
H	-3.13134	0.03519	0.00001
H	-3.99302	2.34897	-0.00001

C	1.30038	1.4963	0.00001
O	2.09078	2.4173	-0.00002
N	1.55275	0.16245	0.00001
C	2.78267	-0.46762	-0.00003
O	2.79866	-1.68396	0.
C	4.0392	0.35527	-0.00002
H	4.05921	1.00928	-0.87018
H	4.0592	1.00928	0.87014
H	4.88792	-0.3215	-0.00002
C	-2.12153	-2.1294	0.
C	-3.12645	-2.79168	-0.00007
H	-4.01751	-3.37471	-0.0001
I	-0.19793	-1.23005	0.00002

Sum of electronic and zero-point Energies= -926.528624
Sum of electronic and thermal Energies= -926.514059
Sum of electronic and thermal Enthalpies= -926.513115
Sum of electronic and thermal Free Energies= -926.572091

5-IIII-CF3 (in Acetonitrile)

C	0.23261	3.9545	0.00003
C	1.30394	3.07837	-0.00012
C	1.08386	1.7066	-0.00005
C	-0.22153	1.24022	0.00011
C	-1.30958	2.09366	0.00029
C	-1.06547	3.46154	0.00025
H	0.40484	5.02172	-0.00001
H	2.32975	3.42135	-0.00026
H	-2.32612	1.73775	0.00047
H	-1.90606	4.14172	0.00041
C	2.25387	0.76299	-0.00007
O	3.39563	1.17286	-0.00061
C	-2.63957	-0.7508	-0.00016
F	-3.03656	-2.02871	-0.00031
F	-3.17368	-0.1661	-1.07766
F	-3.174	-0.16628	1.07727
N	1.8171	-0.52162	0.00032
C	2.57101	-1.68128	0.00015
O	1.98035	-2.74353	0.00006
C	4.06983	-1.5877	0.00004
H	4.41039	-1.02961	-0.8705
H	4.41053	-1.02938	0.87038
H	4.47238	-2.59578	0.00012
I	-0.3913	-0.86547	0.00009

Sum of electronic and zero-point Energies= -1187.469046
Sum of electronic and thermal Energies= -1187.452914
Sum of electronic and thermal Enthalpies= -1187.451970
Sum of electronic and thermal Free Energies= -1187.514863

5-IIII-CH3 (in Acetonitrile)

C	-3.03151	2.32936	-0.00034
C	-1.66372	2.54519	-0.00035
C	-0.78001	1.47302	-0.00018
C	-1.2989	0.18647	-0.00003
C	-2.66114	-0.05427	-0.00003
C	-3.52688	1.03282	-0.00019
H	-3.71322	3.16855	-0.00047
H	-1.23788	3.53961	-0.00046
H	-3.06346	-1.05449	0.00009
H	-4.59384	0.85631	-0.00018
C	0.70704	1.73599	-0.00014
O	1.1292	2.87778	-0.00055
C	-1.44598	-2.90905	0.00044
N	1.37792	0.568	-0.00001
C	2.74331	0.38359	-0.00004
O	3.17348	-0.75587	-0.00002
C	3.6573	1.57902	-0.00011
H	3.45835	2.20306	-0.86991
H	3.45806	2.20343	0.86936
H	4.68368	1.2249	0.00011
H	-2.05191	-2.84278	-0.89792
H	-0.88733	-3.84282	0.00054
H	-2.05187	-2.84257	0.89882
I	0.10822	-1.38448	0.0002

Sum of electronic and zero-point Energies= -889.684158
Sum of electronic and thermal Energies= -889.670267
Sum of electronic and thermal Enthalpies= -889.669322
Sum of electronic and thermal Free Energies= -889.726756

5-IIII-CHCH2 (in Acetonitrile)

C	1.53158	3.55662	-0.05456
C	0.21152	3.13782	-0.05111
C	-0.09709	1.78349	-0.00861
C	0.94491	0.87059	0.03578
C	2.27067	1.26131	0.01961
C	2.55655	2.62038	-0.0241
H	1.76514	4.61177	-0.08852
H	-0.61396	3.83609	-0.08572
H	3.07229	0.53829	0.02558
H	3.58908	2.9418	-0.03809
C	-1.5403	1.3426	-0.03703
O	-2.43626	2.16592	-0.05485
C	2.44217	-1.79252	0.29102
N	-1.60831	-0.00439	-0.04572
C	-2.74124	-0.78883	-0.05888
O	-2.60607	-1.99893	-0.03165
C	-4.09762	-0.13965	-0.10811
H	-4.2326	0.51005	0.75522
H	-4.17508	0.50345	-0.9832
H	-4.8505	-0.92165	-0.12921
H	2.95807	-1.56194	1.21605
C	2.98424	-2.55501	-0.64337
H	3.97603	-2.97706	-0.5231
H	2.46292	-2.79425	-1.56326
I	0.39345	-1.16552	0.11816

Sum of electronic and zero-point Energies= -927.752371
Sum of electronic and thermal Energies= -927.737402
Sum of electronic and thermal Enthalpies= -927.736458
Sum of electronic and thermal Free Energies= -927.796995

5-IIII-Cl (in Acetonitrile)

C	2.26868	3.14616	0.00048
C	0.88869	3.03433	-0.00015
C	0.30024	1.77656	-0.00016
C	1.11446	0.65451	0.00024
C	2.49122	0.73521	0.00103
C	3.06031	2.00387	0.00115
H	2.73207	4.12281	0.00051
H	0.23918	3.89941	-0.00055
H	3.10323	-0.15369	0.00152
H	4.13807	2.09256	0.00182
C	-1.18476	1.62799	-0.00037
O	-1.94062	2.57168	-0.00113
Cl	2.14937	-2.47327	-0.00127
N	-1.52962	0.29888	0.00052
C	-2.79642	-0.26699	0.00039
O	-2.86921	-1.47826	0.00039
C	-3.99916	0.62854	0.00036
H	-3.98073	1.2814	0.87117
H	-3.98101	1.28077	-0.87094
H	-4.88429	0.00063	0.0007
I	0.04567	-1.15771	0.00004

Sum of electronic and zero-point Energies= -1310.051470
Sum of electronic and thermal Energies= -1310.038084
Sum of electronic and thermal Enthalpies= -1310.037140
Sum of electronic and thermal Free Energies= -1310.093297

5-IIII-CN (in Acetonitrile)

C	2.36303	3.0276	0.00002
C	0.98118	2.9428	0.00005
C	0.36338	1.69873	0.00001
C	1.15675	0.56267	-0.00008
C	2.53585	0.6144	-0.00011
C	3.1318	1.87053	-0.00005
H	2.84547	3.99498	0.00005
H	0.34898	3.82048	0.00009
H	3.1416	-0.27948	-0.00016
H	4.21114	1.93518	-0.00006
C	-1.13144	1.5951	0.00002
O	-1.84479	2.57285	-0.00002
C	1.96019	-2.31673	0.00014
N	2.9315	-2.93329	0.00012
N	-1.51227	0.28375	-0.0001
C	-2.79564	-0.23973	-0.00014
O	-2.90709	-1.44945	-0.00025
C	-3.97189	0.69051	0.00025
H	-3.93364	1.34252	0.87125
H	-3.93376	1.34312	-0.87032
H	-4.87658	0.091	0.00012
I	0.05128	-1.23327	0.00001

Sum of electronic and zero-point Energies= -942.646498
Sum of electronic and thermal Energies= -942.632129
Sum of electronic and thermal Enthalpies= -942.631185
Sum of electronic and thermal Free Energies= -942.689587

5-IIII-F (in Acetonitrile)

C	3.35167	1.84251	-0.0002
C	2.03558	2.27346	-0.00027
C	1.01027	1.33686	-0.00021
C	1.33059	-0.01032	-0.00002
C	2.63282	-0.47007	0.00003
C	3.64484	0.48247	-0.00007
H	4.15589	2.56494	-0.00025
H	1.77236	3.32261	-0.00037
H	2.84253	-1.52875	0.00016
H	4.67504	0.15341	-0.00002
C	-0.42353	1.75037	-0.00028
O	-0.77763	2.90652	-0.00018
F	0.99697	-2.77603	0.00029
N	-1.23802	0.64149	-0.00018
C	-2.6238	0.59164	0.00008
O	-3.14202	-0.50649	0.00035
C	-3.40747	1.86929	0.
H	-3.14787	2.46884	0.87064
H	-3.14859	2.46844	-0.87112
H	-4.46257	1.61526	0.00054
I	-0.31966	-1.27865	0.00006

Sum of electronic and zero-point Energies= -949.690130
Sum of electronic and thermal Energies= -949.677224
Sum of electronic and thermal Enthalpies= -949.676280
Sum of electronic and thermal Free Energies= -949.730733

5-IIII-N3 (in Acetonitrile)

C	-1.1307	3.70993	0.02136
C	0.14775	3.19345	0.1495
C	0.34824	1.82213	0.06019
C	-0.74421	0.99789	-0.15304
C	-2.02786	1.48501	-0.29254
C	-2.2082	2.86029	-0.20106
H	-1.29206	4.77658	0.09064
H	1.01053	3.82413	0.31706
H	-2.85899	0.82257	-0.48139
H	-3.20476	3.26637	-0.30747
C	1.71809	1.23569	0.1794
O	2.70293	1.91226	0.36982
N	1.6635	-0.12612	0.03781
C	2.70623	-1.03832	0.08642
O	2.4318	-2.2132	-0.0505
C	4.10634	-0.54517	0.30141
H	4.37938	0.1625	-0.47933
H	4.17156	-0.00127	1.24213
H	4.76903	-1.40465	0.3
N	-2.37097	-1.50832	-0.56594
N	-3.6107	-1.81394	1.40012
N	-2.99158	-1.66377	0.47353
I	-0.27129	-1.04866	-0.25594

Sum of electronic and zero-point Energies= -1014.002731
Sum of electronic and thermal Energies= -1013.987692
Sum of electronic and thermal Enthalpies= -1013.986748
Sum of electronic and thermal Free Energies= -1014.047507

5-IIII-NH2 (in Acetonitrile)

C	3.21823	2.07773	0.00003
C	1.87422	2.41227	-0.00001
C	0.90923	1.41301	-0.00002
C	1.32245	0.09007	0.
C	2.65425	-0.27283	0.00006
C	3.60343	0.74266	0.00008
H	3.96902	2.8558	0.00004
H	1.536	3.43976	-0.00005
H	2.93347	-1.31595	0.00013
H	4.65305	0.48154	0.00012
C	-0.55051	1.76447	-0.00002
O	-0.92579	2.91931	-0.00017
N	-1.29924	0.63036	0.00019
C	-2.67541	0.52778	0.00011
O	-3.16742	-0.5858	0.00007
C	-3.51479	1.77446	0.00007
N	1.19926	-2.86116	0.00011
H	-3.27947	2.38538	0.86989
H	-3.27953	2.38527	-0.86986
H	-4.55966	1.47996	0.00011
H	1.08671	-3.44352	0.82109
H	1.087	-3.44348	-0.82093
I	-0.22166	-1.34066	-0.00007

Sum of electronic and zero-point Energies= -905.744990
Sum of electronic and thermal Energies= -905.731163
Sum of electronic and thermal Enthalpies= -905.730219
Sum of electronic and thermal Free Energies= -905.787215

5-IIII-NHAc (in Acetonitrile)

C	0.13364	3.91497	0.02434
C	1.23528	3.08398	-0.08465
C	1.07174	1.70638	-0.01284
C	-0.20123	1.19185	0.17178
C	-1.31656	1.99695	0.27699
C	-1.13338	3.37203	0.20287
H	0.25692	4.98746	-0.03372
H	2.2361	3.46837	-0.22826
H	-2.29936	1.57349	0.40793
H	-1.99479	4.02133	0.27924
C	2.25014	0.79079	-0.1367
O	3.37782	1.20817	-0.28886
C	-3.24973	-0.69703	-0.43731
O	-2.88264	-0.32616	-1.53298
C	-4.6963	-1.02424	-0.13252
H	-4.80447	-2.10203	-0.00761
H	-5.02307	-0.54366	0.78927
H	-5.31518	-0.69609	-0.96141
N	1.84358	-0.50936	-0.04991
C	2.62412	-1.64972	-0.10818
O	2.06902	-2.72437	0.01641
C	4.10481	-1.52366	-0.32099
H	4.30725	-0.98465	-1.24479
H	4.54938	-0.93308	0.4782
H	4.52749	-2.523	-0.34995
N	-2.37203	-0.8364	0.60013
H	-2.73546	-1.15689	1.48557
I	-0.29159	-0.90426	0.25133

Sum of electronic and zero-point Energies= -1058.378158
Sum of electronic and thermal Energies= -1058.360824
Sum of electronic and thermal Enthalpies= -1058.359879
Sum of electronic and thermal Free Energies= -1058.426288

5-IIII-OCOCH3 (in Acetonitrile)

C	1.61262	3.77473	-0.00008
C	2.33028	2.59147	-0.00002
C	1.65485	1.37814	0.00002
C	0.26781	1.3705	0.
C	-0.47106	2.54042	-0.00004
C	0.22286	3.74442	-0.00008
H	2.13156	4.72308	-0.00012
H	3.41181	2.57212	-0.00002
H	-1.54822	2.51595	-0.00004
H	-0.33697	4.66981	-0.00011
C	2.40761	0.08899	0.00004
O	3.61601	0.03242	0.00012
C	-3.42428	-0.36198	0.00006
O	-3.24696	-1.56218	0.00003
O	-2.42876	0.49947	0.00021
C	-4.78631	0.28055	-0.00036
H	-4.8863	0.9176	0.87717
H	-4.88769	0.91245	-0.88149
H	-5.5532	-0.48685	0.00229
N	1.53408	-0.9694	0.0001
C	1.82468	-2.32656	0.
O	0.88826	-3.09762	0.00015
C	3.25636	-2.77223	-0.00046
H	3.77303	-2.37371	-0.87174
H	3.77399	-2.37344	0.87008
H	3.26917	-3.85742	-0.00043
I	-0.5639	-0.5521	0.0001

Sum of electronic and zero-point Energies= -1078.271472
Sum of electronic and thermal Energies= -1078.255212
Sum of electronic and thermal Enthalpies= -1078.254268
Sum of electronic and thermal Free Energies= -1078.317499

5-IIII-OH (in Acetonitrile)

C	3.30408	1.93489	-0.00193
C	1.97677	2.33039	0.01502
C	0.97303	1.37071	0.01212
C	1.32584	0.03153	-0.00317
C	2.64007	-0.39118	-0.02893
C	3.63094	0.5835	-0.02675
H	4.08924	2.6781	-0.00062
H	1.68592	3.37221	0.02823
H	2.87545	-1.44437	-0.06386
H	4.66857	0.27922	-0.04849
C	-0.47139	1.76127	0.01545
O	-0.83006	2.91846	0.03651
O	1.03467	-2.84887	-0.03429
H	1.17707	-3.18145	0.85637
N	-1.25901	0.64349	-0.0109
C	-2.64122	0.57718	-0.00918
O	-3.15453	-0.52471	-0.01457
C	-3.44393	1.84536	-0.00227
H	-3.19741	2.44074	0.87508
H	-3.188	2.45698	-0.86561
H	-4.49584	1.57792	-0.0104
I	-0.28241	-1.30764	-0.00788

Sum of electronic and zero-point Energies= -925.639782
Sum of electronic and thermal Energies= -925.626414
Sum of electronic and thermal Enthalpies= -925.625470
Sum of electronic and thermal Free Energies= -925.680885

5-IIII-OTs (in Acetonitrile)

C	3.67215	3.28256	0.11369
C	4.1609	1.99135	0.21336
C	3.29168	0.91861	0.06588
C	1.94973	1.16725	-0.17721
C	1.43406	2.44197	-0.28923
C	2.32231	3.50104	-0.13674
H	4.34085	4.12379	0.23048
H	5.20463	1.78143	0.40458
H	0.38906	2.62905	-0.48579
H	1.94226	4.51035	-0.21417
C	3.7814	-0.48451	0.15933
O	4.93398	-0.77809	0.36645
N	2.73996	-1.37151	-0.02837
C	2.76781	-2.75986	-0.01627
O	1.7171	-3.33761	-0.20179
C	4.06842	-3.46418	0.22049
H	4.79672	-3.17102	-0.53344
H	4.48559	-3.16673	1.18088
H	3.88043	-4.53247	0.1893
O	-0.91387	0.6425	-0.7518
S	-1.7551	1.27921	0.37343
O	-1.94878	2.6646	0.06363
O	-1.1692	0.92735	1.63833
C	-3.30811	0.45736	0.22841
C	-4.26615	0.95745	-0.64111
C	-3.5488	-0.68245	0.97923
C	-5.47546	0.29419	-0.76323

H -4.06317 1.86231 -1.19704
C -4.76503 -1.33407 0.84518
H -2.79844 -1.0356 1.67348
C -5.74063 -0.86034 -0.0282
H -6.23179 0.68253 -1.43382
H -4.96401 -2.21926 1.43595
C -7.04702 -1.58504 -0.19032
H -7.27952 -2.18184 0.68985
H -7.86521 -0.88683 -0.36106
H -7.00513 -2.25917 -1.04787
I 0.81759 -0.58493 -0.35332

R-Br-cation (in Acetonitrile)
Br 0. 0. 0.
1 Br 1 3.0600 1.000 0.000000
0.000000 0.000000
Br 0.000000000000 0.000000000000
0.000000000000
1 Br 1 3.0600 1.000 0.000000
0.000000 0.000000
Sum of electronic and zero-point Energies= -2573.736111
Sum of electronic and thermal Energies= -2573.734694
Sum of electronic and thermal Enthalpies= -2573.733750
Sum of electronic and thermal Free Energies= -2573.752286

R-CCH-cation (in Acetonitrile)
C 0. 0. 0.46467
H 0. 0. 1.556
C 0. 0. -0.72401
Sum of electronic and zero-point Energies= -76.181079
Sum of electronic and thermal Energies= -76.178643
Sum of electronic and thermal Enthalpies= -76.177699
Sum of electronic and thermal Free Energies= -76.200291

R-CF3-cation (in Acetonitrile)
C 0. 0.00002 0.00016
F 1.06797 0.60587 -0.00004
F -1.05872 0.62189 -0.00004
F -0.00925 -1.22776 -0.00004
Sum of electronic and zero-point Energies= -337.367604
Sum of electronic and thermal Energies= -337.364343
Sum of electronic and thermal Enthalpies= -337.363399
Sum of electronic and thermal Free Energies= -337.393467

R-CH3-cation (in Acetonitrile)
C 0. -0.00008 0.00005
H 0.94564 -0.54111 -0.00007
H -0.94183 -0.54771 -0.00007
H -0.00382 1.08932 -0.00019
Sum of electronic and zero-point Energies= -39.567030
Sum of electronic and thermal Energies= -39.564177
Sum of electronic and thermal Enthalpies= -39.563233
Sum of electronic and thermal Free Energies= -39.586098

R-CHCH2-cation (in Acetonitrile)
C 0.66964 0.00001 0.00001
H 1.75115 0.00003 0.00005
C -0.58051 0.00003 -0.00004
H -1.14309 0.94522 0.00005
H -1.14279 -0.94546 0.00008
Sum of electronic and zero-point Energies= -77.656207
Sum of electronic and thermal Energies= -77.652389
Sum of electronic and thermal Enthalpies= -77.651445
Sum of electronic and thermal Free Energies= -77.678925

R-Cl-cation (in Acetonitrile)
Cl 0. 0. 0.
Cl 0.000000000000 0.000000000000
0.000000000000
Sum of electronic and zero-point Energies= -459.694034
Sum of electronic and thermal Energies= -459.692618
Sum of electronic and thermal Enthalpies= -459.691673
Sum of electronic and thermal Free Energies= -459.709057

R-CN-cation (in Acetonitrile)
C 0. 0. -0.62907
N 0. 0. 0.53921
Sum of electronic and zero-point Energies= -92.265695
Sum of electronic and thermal Energies= -92.263334
Sum of electronic and thermal Enthalpies= -92.262390
Sum of electronic and thermal Free Energies= -92.284714

R-F-cation (in Acetonitrile)
F 0. 0. 0.
F 0.000000000000 0.000000000000
0.000000000000
Sum of electronic and zero-point Energies= -99.115496
Sum of electronic and thermal Energies= -99.114079
Sum of electronic and thermal Enthalpies= -99.113135
Sum of electronic and thermal Free Energies= -99.129654

R-N3-cation (in Acetonitrile)
N 0. 0. -1.17101
N 0. 0. 1.17114
N 0. 0. -0.00014
Sum of electronic and zero-point Energies= -163.772265
Sum of electronic and thermal Energies= -163.769339
Sum of electronic and thermal Enthalpies= -163.768395
Sum of electronic and thermal Free Energies= -163.793624

R-NH2-cation (in Acetonitrile)
N 0. 0.13818 0.
H 0.84849 -0.48362 0.
H -0.84849 -0.48362 0.
Sum of electronic and zero-point Energies= -55.533420
Sum of electronic and thermal Energies= -55.530581
Sum of electronic and thermal Enthalpies= -55.529637
Sum of electronic and thermal Free Energies= -55.551132

R-NHAc-cation (in Acetonitrile)
C -0.0918 -0.00644 -0.00036
O 0.81714 0.84437 -0.01471
C -1.53126 -0.06929 -0.01799
H -1.85169 -0.82378 -0.74095
H -1.82975 -0.43161 0.97611
H -1.95746 0.91112 -0.21512
N 1.05639 -0.70425 0.13831
H 1.44537 -1.02661 -0.76042
Sum of electronic and zero-point Energies= -208.217932
Sum of electronic and thermal Energies= -208.213334
Sum of electronic and thermal Enthalpies= -208.212390
Sum of electronic and thermal Free Energies= -208.244778

R-OCOCH3-cation (in Acetonitrile)
C 0.06626 0.00788 -0.00044
O -0.92937 -0.78092 0.00005
O -0.94153 0.775 0.00007
C 1.50377 0.01112 -0.00028
H 1.88135 1.03092 -0.01092
H 1.83169 -0.53804 0.89137
H 1.83403 -0.55949 -0.87707
Sum of electronic and zero-point Energies= -228.056627
Sum of electronic and thermal Energies= -228.052190
Sum of electronic and thermal Enthalpies= -228.051246
Sum of electronic and thermal Free Energies= -228.083447

R-OH-cation (in Acetonitrile)
O 0. 0. 0.11505
H 0. 0. -0.92043
Sum of electronic and zero-point Energies= -75.272886
Sum of electronic and thermal Energies= -75.270525
Sum of electronic and thermal Enthalpies= -75.269581
Sum of electronic and thermal Free Energies= -75.289310

R-OTs-cation (in Acetonitrile)
O 1.56281 -0.00069 1.26699
S 1.91753 -0.00043 -0.24009
O 2.31581 -1.24929 -0.751
O 2.31791 1.24816 -0.75002
C 0.21963 0.00004 0.61314
C -0.46299 -1.24388 0.49365
C -0.46117 1.24461 0.49302
C -1.76177 -1.22279 0.07818
H 0.07257 -2.16444 0.68669
C -1.76027 1.22539 0.07708
H 0.07555 2.16456 0.6858
C -2.43291 0.00192 -0.14048
H -2.29469 -2.15196 -0.0702
H -2.29137 2.15535 -0.07216
C -3.85868 0. -0.55278
H -4.13001 0.91153 -1.07958
H -4.09962 -0.87049 -1.15935
H -4.47619 -0.05476 0.35159
Sum of electronic and zero-point Energies= -894.389577
Sum of electronic and thermal Energies= -894.379709
Sum of electronic and thermal Enthalpies= -894.378765
Sum of electronic and thermal Free Energies= -894.425301
